# Visible-Light Photoredox Catalyzed Dehydrogenative Synthesis of Allylic Carboxylates from Styrenes

**DOI:** 10.1021/acs.orglett.1c01375

**Published:** 2021-05-25

**Authors:** Yang Liu, Simone Battaglioli, Lorenzo Lombardi, Arianna Menichetti, Giovanni Valenti, Marco Montalti, Marco Bandini

**Affiliations:** † Dipartimento di Chimica “Giacomo Ciamician”, Alma Mater Studiorum, Università di Bologna, via Selmi 2, 40126 Bologna, Italy; § Consorzio CINMPIS, via Selmi 2, 40126 Bologna, Italy

## Abstract

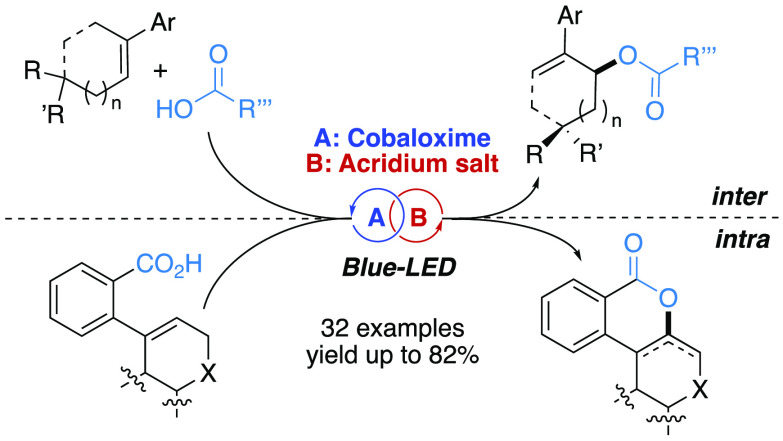

The visible-light photoredox/[Co(III)] cocatalyzed dehydrogenative functionalization of cyclic and acyclic styryl derivatives with carboxylic acids is documented. The methodology enables the chemo- and regioselective allylic functionalization of styryl compounds, leading to allylic carboxylates (32 examples) under stoichiometric acceptorless conditions. Intermolecular as well as intramolecular variants are documented in high yields (up to 82%). A mechanistic rationale is also proposed on the basis of a combined experimental and spectroscopic investigation.

Unactivated olefins are convenient feedstocks in organic synthesis due to their large availability and intrinsic wide chemical flexibility.^1^ Generally, the chemical manipulation of alkenes requires site-selective electrophilic activation of the π-system using noble transition metals or harsh Brønsted acidic conditions. Very recently, radical variants started flanking these approaches^2^ with the use of dedicated visible-light induced generation of radical cations that could evolve into chemical diversity/complexity via subsequent stoichiometric oxidant-free dehydrogenative couplings.

In this context, the combined use of Fukuzumi acridinium salts (visible-light photoredox abstractors of electrons from olefins)^3^ and [Co(II)/(III)] oximine proton acceptors (i.e., cobaloximes)^4^ has recently received extensive attention in the direct functionalization of unactivated alkenes under a catalytic hydrogen evolution regime (Figure 1a).^5,6^ In the realm of photoredox acceptorless dehydrogenation reactions, Lei documented the *anti*-Markovnikov oxidation of styrenes with water,^5a^ alcohols, and azoles.^5b^ In addition, an elegant [4 + 2]-type cycloaddition between alkenes and aromatic ketoimines to deliver dihydroisoquinolines was also reported.^5c^ Subsequently, Wu extended this approach to the formation of alkenylphosphines via a dehydrogenative C–P bond forming process under photosensitizer-free conditions.^5d^

**Figure 1:**
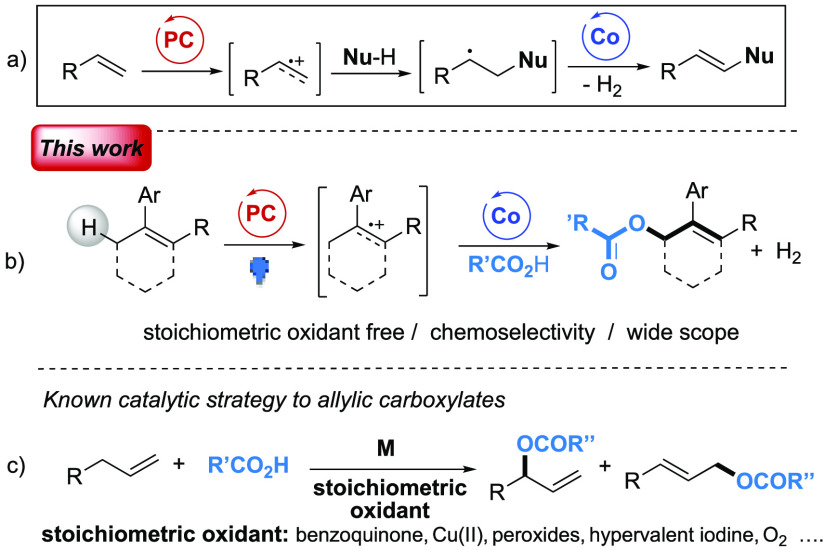
(a) Co/visible-light mediated functionalization of olefins via SET oxidation. (b) Present working plan. (c) Classic metal catalyzed oxidative synthesis of allylic carboxylates via C–H functionalization.

In a continuation of our ongoing interests toward the realization of visible-light photoredox promoted synthetic protocols^7^ and metal mediated allylic nucleophilic substitutions,^8^ we envisioned the opportunity to apply the visible-light induced cobaloxime/acridinium dual catalysis to the preparation of allylic esters via dehydrogenation of Csp^3^–H bonds under stoichiometric oxidant-free conditions (Figure 1b). Such an approach would represent a significant improvement with respect to the known oxidant-based synthesis of allylic carboxylates (Figure 1c).^9^

In this scenario, 1-phenyl-1-cyclohexene (**1a**) and butanoic acid (**2a**) were elected as model substrates in order to tackle the intrinsic regioselective issues of the protocol (see compounds **5aa**/**5aa′**/**5aa″** in the Table 1 graphic). In addition, rigorous base-free conditions were targeted in order to prevent undesired photoinduced decarboxylative events.^10^

**Table 1:**
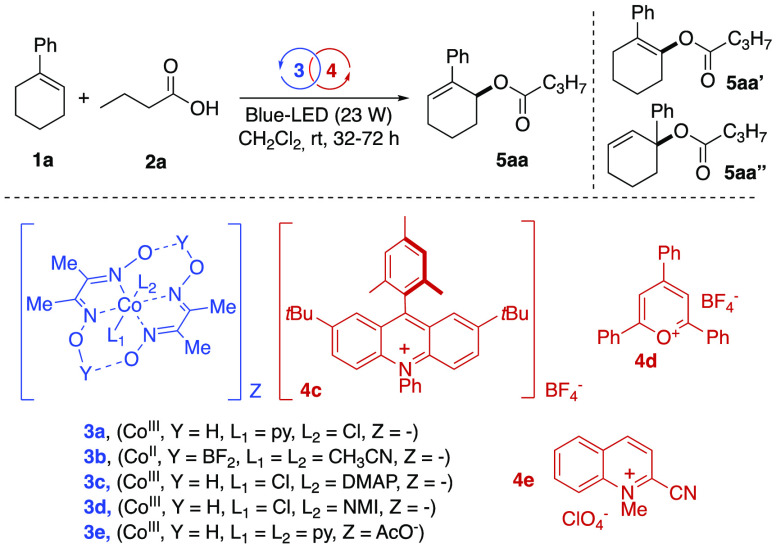
Optimization of the Reaction Conditions

run^a^	reaction conditions	yield **5aa** (%)^b^
1	**3a**/**4a**	63
2	**3b**/**4a**	traces
3	**3c**/**4a**	33
4	**3d**/**4a**	37
5	**3e**/**4a**	50
6	**3a**/**4b**	52
7	**3a**/**4c** (48 h)	60
8	**3a**/**4d**	nr
9	**3a**/**4e**	nr
10	**3a**/–	nr
11	**3a**/**4a** in reagent grade/undegassed DCM	nr
12	**3a**/**4a** in DMF	nr
13	**3a**/**4a** in (CH_2_Cl)_2_	41
14	**3a**/**4a** in CH_3_CN	nr
15	**3a:4a** blue LED (1 W)	20
16	**3a:4a** blue LED (40 W), 32 h	31
17	**3a:4a** dark	nr

a Reaction conditions: **1a** (0.1 mmol, 0.1 M), **2a** (10 equiv), **3** (5 mol %), **4** (2.5 mol %) under nitrogen conditions and degassed solvent (72 h), unless otherwise specified.

b Determined after flash chromatography. In many cases and more pronouncedly for yields <50%, discrete amounts of unreacted olefin **1a** were observed even upon prolonged irradiation times. DMAP: 4-(dimethylamino)-pyridine. NMI: *N*-methylimidazole. nr: no reaction.

At the outset of the optimization stage, we discovered that the combined use of [Co(III)(dmgH)_2_pyCl] (**3a**) (5 mol %, dmgH = dimethylglyoximate, py = pyridine) and Fukuzumi 9-mesityl-10-methylacridinium perchlorate (**4a**) (2.5 mol %) promoted the chemoselective *anti*-Markovnikov (**5aa** vs **5aa″**)^5a,11,12^ formation of the butyrate **5aa** in 63% yield (blue LED 23 W 465 nm, rt, DCM, entry 1, Table 1). Furthermore, high allylic ester (**5aa**) vs enolester (**5aa′**) chemoselectivity (generally >25:1) was observed as well (see mechanistic discussion for details).

Interestingly, among the cobaloximes tested (**3a**–**e**), while [Co(dmgBF_2_)_2_(ACN)_2_] (**3b**) demonstrated inertness in the coupling process (entry 2), the analogous Co(III) featured different ancillary ligands (i.e., DMAP, NMI, py: **3c**–**e**) which promoted the process but to lower extents with respect to **3a** (yield: 33–50%, entries 3–5). Perchlorate acridiunim **4a** proved to be far superior with respect to the corresponding 9-mesityl-10-methylacridinium tetrafluoroborate (**4b**, 52% yield) and to photosensitizers **4d**,**e** (entries 7 and 8). Additionally, removal of the photosensitizer (PC) (entry 10) caused the failure of the reaction. The reaction medium was then assessed (entries 11–14). Here, dry and degassed CH_2_Cl_2_ emerged as the optimal solvent, suggesting also the presence of radical intermediates during the reaction course. Then, different conditions for light exposure (i.e., dark and 1/40 W blue LEDs) were examined providing insights about the genuineness of the light-driven method (entry 17) but not improved chemical outcomes (entries 15 and 16).

With the aim of further improving the performance of the dehydrogenative cross-coupling process, we reasoned that the employment of a photosensitizer featuring a longer excited state lifetime (**4a** τ = 6.4 ns)^13^ and higher thermal stability (side dealkylation events have been documented with *N*-alkyl acridinuim derivatives)^14^ could provide a higher concentration of the key radical cation (Figure 1a). Therefore, in line with the recent discoveries by Nicewicz,^14^ the new *N*-phenyl dye **4c** was synthesized and fully characterized spectroscopically: (i) singlet excited state energy = 2.64 eV; (ii) singlet excited state lifetime = 17.6 ns; (iii) cyclic voltammetry revealed that two reversible one-electron reductions were observed at −0.59 and −1.65 V (vs SCE see Figure S1).^15^ Finally, the excited state reduction potential was estimated to be 2.05 V and hence comparable to that of **4a**.

Interestingly, the *N*-phenyl acridinium **4c** provided **5aa** in a similar extent to **4a** (60% yield) but with a shorter reaction time (48 h, entry 7 vs entry 1), enabling also the reactivity of several inert substrates with **4a** to be unlocked. As a partial explanation of the recorded outcomes, we compared the relative decrease of the singlet excited state lifetime of **4a** and **4c** in the presence of the same concentration of **1a** (21 mM) in CH_2_Cl_2_. The results revealed that the singlet excited state of **4a** was quenched only by 55%. However, in the case of **4c**, quenching was as high as 71%. Accompanied by the previous results, the Stern–Volmer quenching constants were found to be *k*_Q_ = 8.6 × 10^9^ M^–1^ s^–1^ and *k*_Q_ = 6.6 × 10^9^ M^–1^ s^–1^ for **4a** and **4c**, respectively.^16^

Having established the optimal reaction conditions, we faced the substrate scope of the methodology by subjecting to the model photoredox cross-coupling conditions a range of carboxylic acids **2b**–**m** and alkene **1a** (Scheme 1). Interestingly, good yields were obtained for linear (**5ac**,**d**), branched (**5ae**) and hydrocinnamic carboxylates **5af** (58%). Analogously, acetic acid proved competent in the dehydrogenative coupling, delivering the desired acetate **5ab** in 58% yield. α,β-Unsaturated carboxylic acids worked also satisfyingly, providing the carboxylates **5ag**–**h** in moderate yield (43%) but without appreciable erosion on the stereochemical information on the pristine carboxylic acid. Additionally, functionalized benzoic acids (**2i**–**m**) effectively participated to the oxidative coupling, delivering the products **5ai**–**am** in moderate to good yields (45–71%), regardless the position as well as electronic properties of the aryl substituents.

**Scheme 1:**
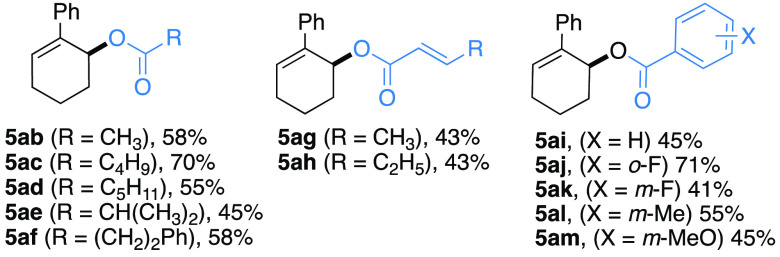
Generality of the Method towards Differently Substituted Carboxylic Acids Based on Table 1, Entry 1, Conditions

Optimal conditions were then applied to a series of cyclic as well as acyclic styryl derivatives in order to assess the generality of the protocol toward unsaturated hydrocarbons (Scheme 2). First, a range of functionalized 1-aryl-cyclohexenes (**1b**–**n**) were subjected to the oxidative photocatalyzed intermolecular derivatization. Substituents can be effectively accommodated at the C-4 position of the cyclohexenyl scaffold (i.e., *t*Bu, Me, and *gem*-dimethyl), generating the corresponding carboxylates **5** in a yield up to 71%.

**Scheme 2:**
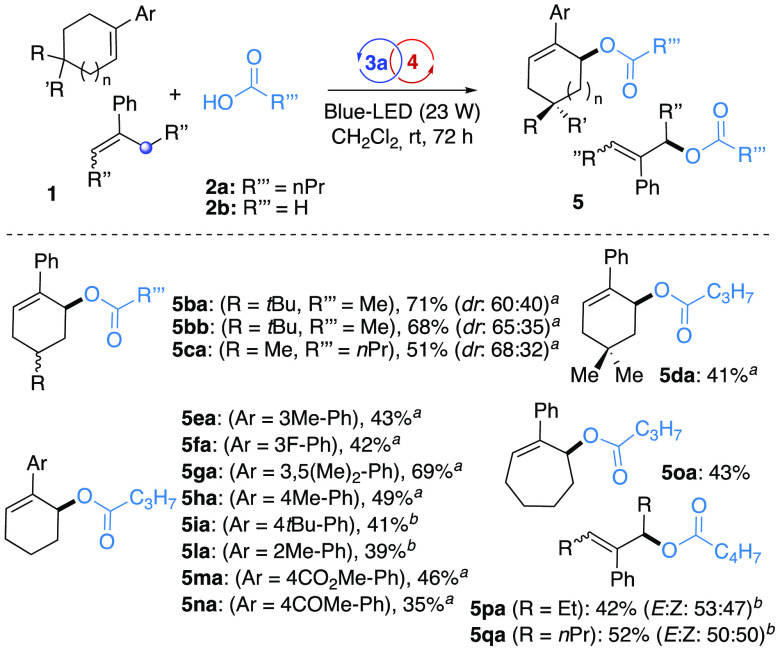
Scope of the Reaction: Alkenes^,^ Catalyst **4a**. Catalyst **4b**. The blue spot shows the dehydrogenation site.

In this direction, a library of carboxylic acids **6a–d** were readily obtained via Suzuki cross-coupling and directly subjected to optimal reaction parameters (Scheme 3). Additionally, the adoption of 1-aryl-cyclohexenyl units, carrying both EWGs (i.e., CO_2_Me, COMe, F) and EDGs (i.e., Me and di-Me, *t*Bu) at the *ortho*-, *meta*-, and *para*-positions of the arene, led to the allylic carboxylates **5** in moderate to good yields (up to 69%) via *anti*-Markovnikov condensation. The generality of the protocol was also ascertained for the cycloeptenyl compound **1o** that generated the desired benzoate **5oa** in 43% yield.^17^ Finally, C7, C9 and diphenyl-substituted C5 acyclic styryl compounds **1p**,**q** were conveniently synthesized as an *E:Z*-mixture via Suzuki cross-coupling of the corresponding enol triflates or Grignard addition/dehydration sequences (see SI) and subjected to the oxidative coupling in the presence of **4c**.^18^ Also, in these cases, the allylic esters were isolated in satisfying yields (up to 52%) and marked allylic ester vs enol ester selectivity.

**Scheme 3:**
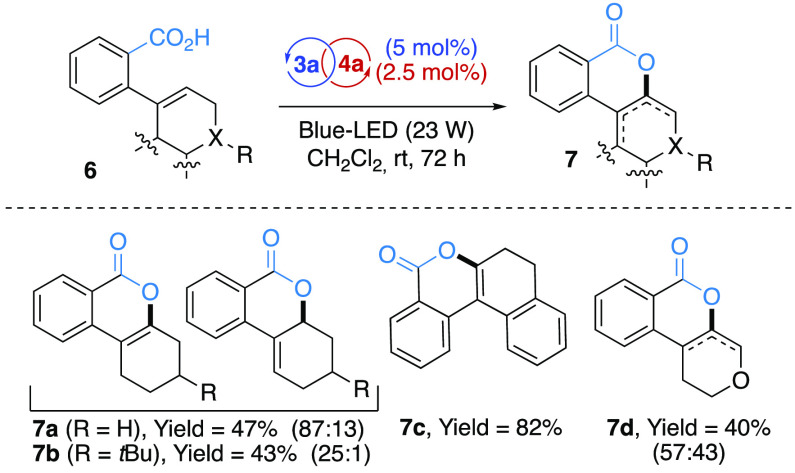
Synthesis of Functionalized Isocoumarin Derivatives **7** via Intramolecular Photoredox/Co Cocatalyzed Dehydrogenative Reaction

Moreover, the synthetic versatility of the procedure was further emphasized by implementing an intramolecular variant. In particular, the photoredox procedure was applied to the direct synthesis of isocoumarin scaffolds **7**(19) via an unprecedented intramolecular dehydrogenative formal Csp^2^–H functionalization.^20^ Interestingly, the desired isocoumarins **7** were obtained from moderate to excellent yields (up to 82%) accompanied by a high selectivity toward the 3,4-unsaturated scaffold (up to >25:1).^21^ Differently, the corresponding pyranyl core **7d** was isolated in 40% yield as a mixture (ca. 1:1) of the 1,4- and 1,10*b*-dihydropyranyl isomers. Therefore, the synthetic flexibility of the allylic carboxylates was examined (Figure 2a). First, the epoxidation of the cyclohexenyl core was carried out effectively (mCPBA, CH_2_Cl_2_, 0 °C, 16 h) delivering the cyclohexene oxide **8ab** in 88% yield. In addition, the acetyl group of **5ab** could be conveniently saponified (NaOH, MeOH, rt) to release the corresponding allylic alcohol **9ab** in 89% yield, proposing the present methodology as a catalytic indirect hydroxylation of allylic Csp^3^–H bonds.

**Figure 2:**
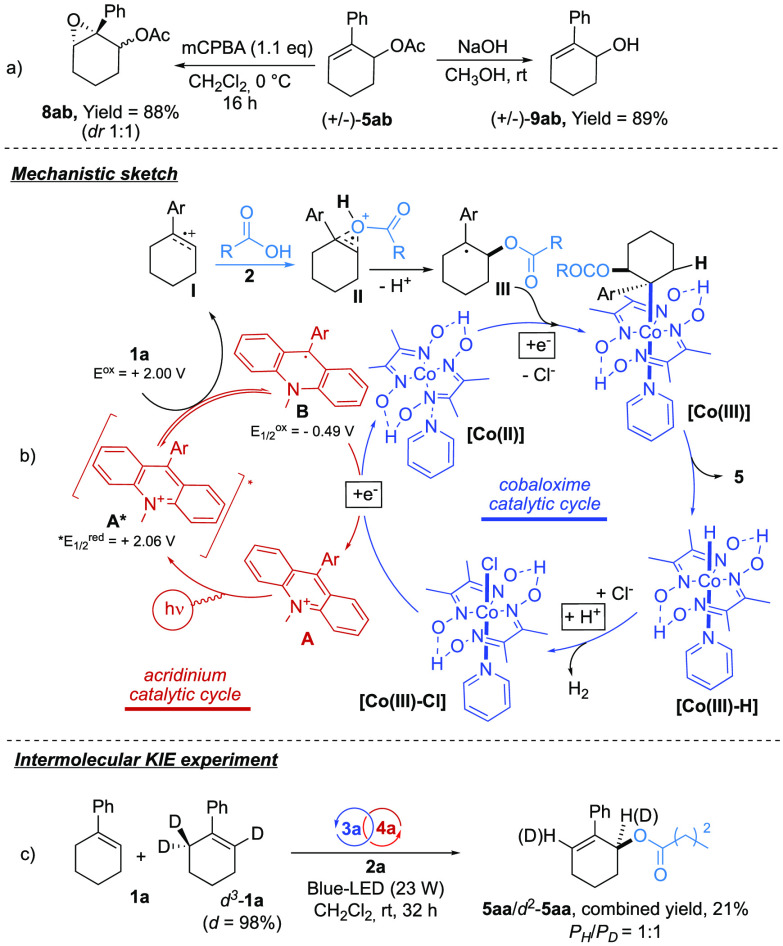
(a) Synthetic manipulations of the allyl acetate **5ab**. (b) Mechanistic hypothesis for the dual Co/acridinium dehydrogenative functionalization of olefins (the case for PC **4a** is depicted). (c) Parallel isotope labeling experiments.

In order to get some insight into the reaction machinery, several dedicated control experiments were carried out. First, the on/off irradiation experiment (Schemes S1 and S2) revealed that, upon a relatively short induction period, the reaction proceeded smoothly under blue LED irradiation, which showed negligible advancements during the light-free stages. Radical trap experiments with TEMPO led to contrasting results with respect to similar processes previously investigated.^3d,6,22^ In particular, attempts to replace the cobaloxime **3a** with stoichiometric amounts of TEMPO failed in promoting the photoredox condensation, and no overall inhibition was observed when the radical trap was added to optimal conditions.

Mechanistically, the schematic representation depicted in Figure 2b is proposed. Irradiation with a blue LED (465 nm, 23 W) promotes the [Acr^+^-Mes ClO_4_^–^] **A** into the corresponding excited state [Acr^+^-Mes ClO_4_^–^]* A* (*E*^red^[Acr^•^-Mes^•+^/Acr^•^-Mes] = +2.06 V vs SCE)^11a^ that could oxidize the olefin **1a** (*E*^ox^**1a**/**1a**^•+^ = +2.00 V vs SCE)^23^ via an SET process and deliver the aryl cation **I** and the reduced form of the PC **B**.^24^ Therefore, the reoxidation of **B** by [Co(III)(dmgH)_2_pyCl] (*E*_1/2_^red^ Co(III)/Co(II) = −0.67 V vs SCE)^25a^ would restore the [Acr^+^-Mes ClO_4_^–^] **A** with the concomitant reduction of the cobalt species. The radical cation **II** could undergo *anti*-Markovnikov condensation with the carboxylic acid^26^ releasing the α-carboxyl-benzyl radical **III** upon deprotonation. Therefore, the in situ formed [Co(II)] complex might trap the radical **III** to deliver a [Co(III)]–alkyl intermediate that would rapidly evolve into the final product **5** and the corresponding [Co(III)–H] via β-H elimination.^27,28^

It is worth mentioning that, as the β-H elimination of alkyl–Co species is subjected to rigid stereochemical constraints (i.e., *syn* periplanar conformations are required),^29^ we can speculate that the β-C*H*–OCO_2_R cannot arrange *syn* periplanar with respect to the C–Co linkage, making the formation of the enolester **5′** unlikely. Last, protonation of [Co(III)–H] would restore the catalytically active [Co(III)] adduct via a hydrogen evolution reaction (HER).^30^

Finally, a kinetic isotope effect (KIE) experiment was carried out with deuterated phenyl-cyclohexene *d*^3^-**1a** (Figure 2c).^31^ In the intermolecular competition experiment, a **1a**/*d*^3^-**1a** 1:1 mixture was utilized under optimal conditions (32 h, yield = 21%). Interestingly, no isotopic effect was observed (**5aa**:*d*^2^-**5aa** = 1:1), excluding the β-elimination from the rate-determining step of the catalytic cycle.

In conclusion, in this study, we have documented an unprecedented dual visible-light/cobalt catalyzed redox protocol for the preparation of cyclic and acyclic allylic carboxylates via direct Csp^3^–H oxidation of styryl compounds with carboxylic acids. The oxidant-free methodology showed peculiar *anti*-Markovnikov regiochemistry. An intramolecular variant was also realized, resulting in the direct preparation of isocoumarin scaffolds in up to 82% yield . Studies toward the extension of the present methodology to the realization of direct allylic C–H activation protocols are underway in our laboratories.

## Supporting Information Available

Synthetic and catalytic procedures, analytic characterization of unknown compounds, mechanistic experiments, and NMR spectra (PDF)
