# Supplementary material for: Visible-Light Photoredox Catalyzed Dehydrogenative Synthesis of Allylic Carboxylates from Styrenes
Source: Org Lett. 2021 May 25;23(11):4441–6. doi: 10.1021/acs.orglett.1c01375 (PMC8289305; doi:10.1021/acs.orglett.1c01375)
Supplement: Supplementary file 1 — ol1c01375_si_001.pdf [file ol1c01375_si_001.pdf]

Visible-light photoredox catalyzed dehydrogenative synthesis of allylic carboxylates from styrenes

Yang Liu,[a] Simone Battaglioli,[a] Lorenzo Lombardi,[a] Arianna Menichetti,[a] Giovanni Valenti,[a] Marco Montalti[a] and Marco Bandini*[a,b]

[a] Dipartimento di Chimica "Giacomo Ciamician", Alma Mater Studiorum – Università di Bologna, via Selmi 2, 40126 Bologna, Italy

[b] Consorzio CINMPIS, via Selmi 2, 40126 Bologna, Italy

Table of Contents

General methods S2
Synthesis of olefins for intermolecular reactions S3
Table S1. Optimization intermolecular reaction conditions S6
General procedure for intermolecular process S9
Synthesis of substrates **6a-d** S15
General procedure for intramolecular process S18
Functionalization of **5ab** S20
Monitoring of the reaction conversion S22
Kinetic isotope effect experiment S24
Synthesis of the photocatalyst **4c** S27
NMR Spectra S32
References S105

General Methods.

^{1}H-NMR spectra were recorded on Varian 400 (400 MHz) spectrometers. Chemical shifts are reported in ppm from TMS with the solvent resonance as the internal standard (deuterochloroform: 7.24 ppm). Data are reported as follows: chemical shift, multiplicity (s = singlet, d = doublet, dd= doublet doublet, t = triplet, td = triple doublet, dt = double triplet, q = quartet, sext = sextet, sept = septet, p = pseudo, b = broad, m = multiplet), coupling constants (Hz). ^{13}C-NMR spectra were recorded on a Varian 400 (100 MHz) spectrometers with complete proton decoupling. Chemical shifts are reported in ppm from TMS with the solvent as the internal standard (deuterochloroform: 77.0 ppm).

GC-MS spectra were taken by EI ionization at 70 eV on a Hewlett-Packard 5971 with GC injection. They are reported as: *m/z* (rel. intense). LC-electrospray ionization mass spectra were obtained with Agilent Technologies MSD1100 single-quadrupole mass spectrometer.

Chromatographic purification was done with 240-400 mesh silica gel. Other anhydrous solvents were supplied by Sigma Aldrich in Sureseal® bottles and used without any further purification. Reactions requiring temperatures higher than r.t. were carried out in an oil bath on a heated stirring plate. Commercially available chemicals were purchased from Sigma Aldrich, Stream and TCI and used without any further purification. Melting points were determined with Bibby Stuart Scientific Melting Point Apparatus SMP 3 and are not corrected. Agilent Technologies LC/MSD Trap 1100 series (nebulizer: 15.0 PSI, dry Gas: 5.0 L/min, dry Temperature: 325 °C, capillary voltage positive scan: 4000 mA, capillary voltage negative scan: 3500 mA). Compounds **1a**, **2a** – **2m** are commercially available.

UV−vis absorption spectra were recorded at room temperature by means of Perkin-Elmer Lambda 45 spectrophotometer. Quartz cuvettes (Hellma) with optical path length of 1 cm were used. The fluorescence spectra were recorded with an Edinburgh FLS920 equipped photomultiplier Hamamatsu R928P. The same instrument connected to a PCS900 PC card was used for the Time Correlates Single Photon Counting (TCSPC) experiments. Luminescence quantum yields (uncertainty, ± 15%) were determined using rhodamine 101 solution in ethanol as a reference (Φ = 1.0). Fluorescence intensities were corrected for inner filter effects according to standard methods.[1]

Synthesis of olefins for intermolecular reactions

Substrates of enol triflates

The enol triflate of the cyclohexanone was obtained in 63% following the procedure described in the literature.[2]

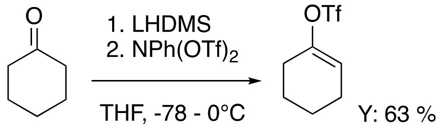

The enol triflate deriving from 4-substituted cyclohexanones were obtained following the procedure described in the literature.[2]

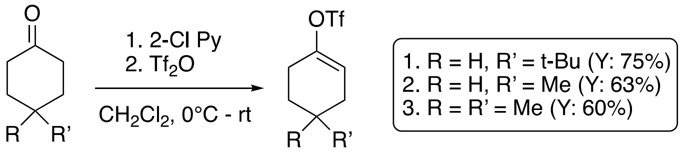

The enol triflates were subsequently converted into the corresponding phenylcyclohexenes **1b-d** by using the following procedure.

Synthesis of the 1-phenylcyclohexenes 1b-h/l-n

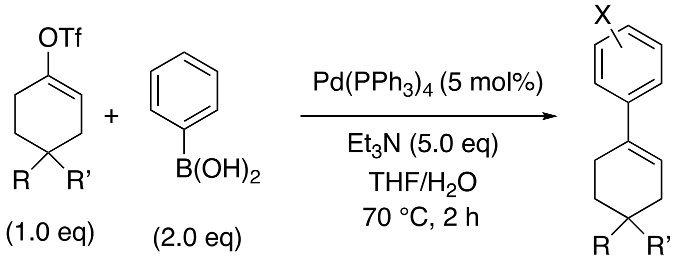

1b (R = *t*Bu, R' = H, X = H), Y: 77%
1c (R = Me, R' = X = H), Y: 72%
1d (R = R' = Me, X = H), Y: 73%
1e (R = R' = H, X = 3-Me), Y: 73%
1f (R = R' = H, X = 3-F), Y: 72%
1g (R = R' = H, X = 3,5-Me_2), Y: 65%
1h (R = R' = H, X = 4-Me), Y: 72%
1l (R = R' = H, X = 2-Me), Y: 75%
1m (R = R' = H, X = 4-CO_2Me), Y: 70%
1n (R = R' = H, X = 4-COMe), Y: 75%

Under N_2, a dry Schlenk tube was charged with THF (5 ml), deionized H_2O (1 mL) and Et_3N (5.0 mmol). Then phenylboronic acid (2.0 mmol) and the triflate (1.0 mmol) were added. The reaction was stirred at 70 °C for 2 hours or until completion (TLC). Then H_2O (5 mL) was added and the resulting mixture was extracted with EtOAc (2 x 15 mL), then the organic phase was dried over Na_2SO_4 and concentrated under vacuum. The residue was purified with gel flash chromatography. The NMR spectra of compounds **1b-d** and **1e-h/l,m** were in agreement with those reported in the literature.[3,4]

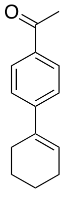

1n. White solid. M.p. = 73.9 – 75.1 °C; **^{1}H NMR** (400 MHz, $CDCl_3$) δ 7.88 (d, *J* = 8.4 Hz, 2H), 7.44 (d, *J* = 8.5 Hz, 2H), 6.27 – 6.24 (m, 1H), 2.57 (s, 3H), 2.43 – 2.38 (m, 2H), 2.25 – 2.20 (m, 2H), 1.81 – 1.75 (m, 2H), 1.69 – 1.63 (m, 2H). **^{13}C NMR** (100 MHz, $CDCl_3$) δ 179.69, 147.23, 135.80, 135.18, 128.39, 127.41, 124.83, 27.11, 26.51, 25.97, 22.84, 21.92. **GC-MS**: 185 (100), 200 (75), 129 (48), 157 (45); **Anal. Calc.** for ($C_{14}H_{16}O$: 200.12): C, 83.96; H, 8.05; O, 7.99; found: C, 84.24; H, 8.19; O, 8.23.

Synthesis of the 1-phenylcyclohexene 1i

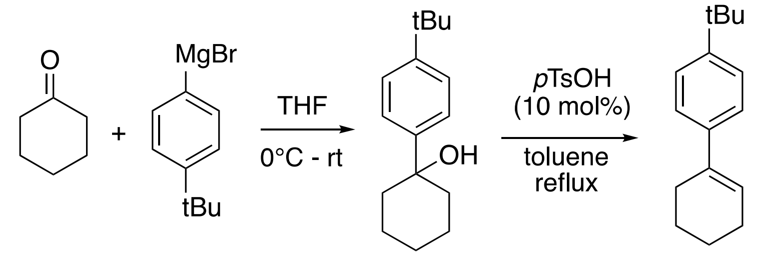

1i, Y: 65%

In a dry three-necked flask equipped with condenser the cyclohexanone (3 mmol) was dissolved in 1 ml of THF and added dropwise at 0 °C to a solution of freshly prepared 4-tert-butylphenylmagnesium bromide (3.6 mmol, ca. 0.5 mM). The reaction was stirred at rt for 2 h when the TLC showed the reaction completed. Then, a saturated aqueous solution of NH_4Cl was added (5 mL) at 0 °C and the resulting mixture was extracted with AcOEt (3 x 5 mL). The organic phase was dried over Na_2SO_4 and concentrated under vacuum. The reaction crude was used directly in the next step without further purification. A Schlenk tube was charged with 5 mL of reagent grade toluene, the tertiary alcohols (crude product, ca. 3 mmol) and *p*TsOH (10 mol%). The reaction was stirred at reflux until completion (TLC). Then H_2O (5 mL) was added, and the resulting mixture was extracted with EtOAc (2 x 15 mL), then the organic phase was dried over Na_2SO_4 and concentrated under vacuum. The residue was purified with flash chromatography (*n*Hex 100%) to give **1i** as a colorless oil (380 mg, 65%). The NMR spectra of **1i** is in agreement with that reported in literature.[8]

Synthesis of substrates 1o-q

The first step for the synthesis of the compounds **1o-q** involves the formation of enol triflates. Enol triflate precursors were obtained through the following procedure.[5] The spectra of the enol triflates are in agreement with those reported in literature.[6,7]

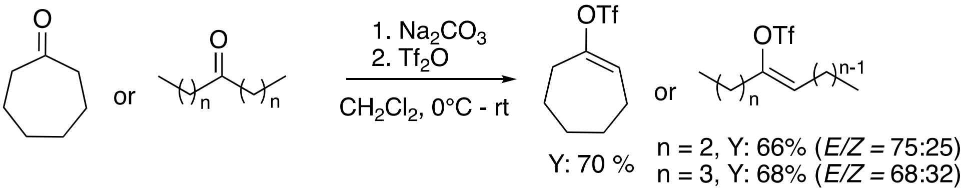

The enol triflate was subsequently used in the Suzuki-cross coupling as previously described for substates **1b**-**n**. The spectra of olefin **1o** is in agreement with that reported in literature.[8]

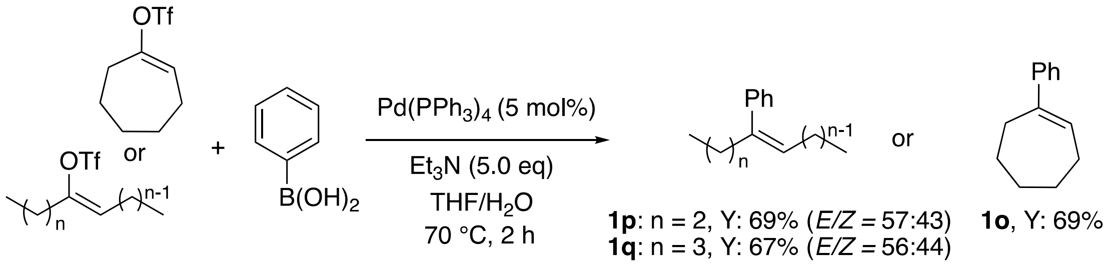

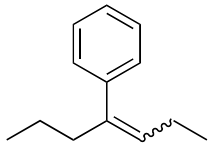

1p. Colorless oil. **^{1}H NMR** (400 MHz, $CDCl_3$) Signal of isomer *Z*-**1p** δ 7.34 – 7.18 (m, 4H), 7.13 – 7.11 (m, 1H), 5.41 (t, *J* = 7.3 Hz, 1H), 2.28 (td, *J* = 7.5 Hz, *J* = 0.8 Hz, 2H), 1.92 (p, *J* = 7.4 Hz, 2H), 1.40 – 1.25 (m, 2H), 0.91 (t, *J* = 7.5 Hz, 3H), 0.89 – 0.83 (m, 3H). Signal of isomer *E*-**1p** δ 7.34 – 7.18 (m, 4H), 7.13 – 7.11 (m, 1H), 5.64 (t, *J* = 7.2 Hz, 1H), 2.48 – 2.44 (m, 2H), 2.20 (p, *J* = 7.5 Hz, 2H), 1.40 – 1.25 (m, 2H), 1.04 (t, *J* = 7.5 Hz, 3H), 0.89 – 0.83 (m, 3H). **^{13}C NMR** (100 MHz, $CDCl_3$) δ 143.40, 141.47, 140.16, 130.87, 129.02, 128.37, 128.06, 127.86, 126.32, 126.20, 41.27, 31.60, 22.19, 21.84, 21.82, 21.18, 14.72, 14.41, 13.91, 13.57. **GC-MS**: 174 (6), 145 (22), 131 (100), 117 (32); The signals of the two stereoisomers were assigned by the 1D-NOESY experiment.

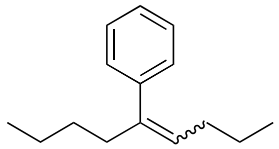

1q. Colorless oil. **^{1}H NMR** (400 MHz, $CDCl_3$) Sum of the signals of the two isomers δ 7.33 – 7.13 (m, 8H), 7.12 - 7.09 (m, 2H), 5.62 (t, *J* = 7.2 Hz, 1H), 5.41 (t, *J* = 7.3 Hz, 1H), 2.49 - 2.45 (m, 2H), 2.31 – 2.29 (m, 2H), 2.18 – 2.13 (m, 2H), 1.90 – 1.85 (m, 2H),1.50 – 1.41 (m, 2H), 1.35 - 1.24 (m, 10H), 0.95 (t, *J* = 7.4 Hz, 3H), 0.87 – 0-80 (m, 9H). **^{13}C NMR** (100 MHz, $CDCl_3$) δ 143.51, 141.63, 141.05, 140.20, 128.88, 128.42, 128.05, 127.84, 127.02, 126.30, 126.28, 126.13, 39.01, 30.91, 30.87, 30.60, 30.35, 29.45, 23.24, 23.04, 22.66, 22.22, 13.94, 13.93, 13.90, 13.74. **GC-MS**: 202 (14), 173 (13), 145 (60), 118 (100);

Table S1. Optimization of the intermolecular reaction conditions.

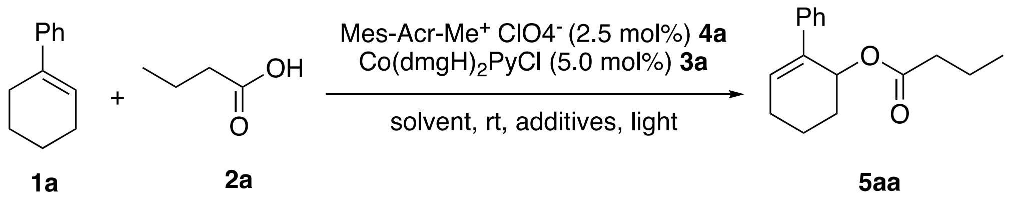

Entry	Reaction condtions	Yield (%)[a]
1	Toluene	17
2	DMSO	Traces
3	CH_3CN	46
4	CH_2Cl_2/CH_3CN (20:1)	54
5	CH_2Cl_2/CH_3CN (10:1)	46
6	CH_2Cl_2/HFIP (1:1)	0
7	chlorobenzene	19
8	AcOEt	Traces
9	*c*Hex/CH_2Cl_2 (5:1)	Traces
10	**3a**:**4a** (1:1, 5 mol%)	59
11	**3a**:**4a** (1:2, 1.25/2.5 mol%)	25
12	0.5 M in CH_2Cl_2	48
13	0.2 M in CH_2Cl_2	44
14	0.025 M in CH_2Cl_2	17
15	ZnF_2 (1 eq.) as an additive	42
16	BLUE LED 23 W (20 h)	< 10
17	BLUE LED 40 W (20 h)	38

[a] Isolated yields after flash chromatography.

Optimized general procedure for the intermolecular process with PC 4a.

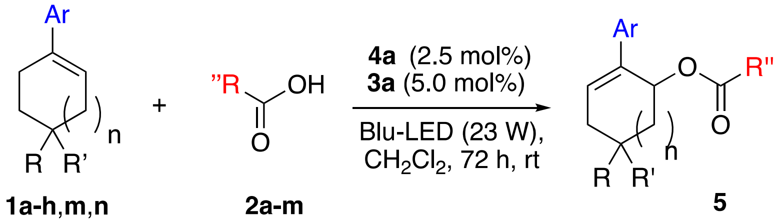

A 5 mL dry vial equipped with a stirring bar was charged with: acridinium **4a** (1 mg, 2.5 mol%), [Co(dmgH)$_2$(Py)(Cl)] (2.0 mg, 5 mol%), dry DCM (1 mL), styryl derivative **1** (0.1 mmol) and the desired carboxylic acid (1.0 mmol). The solution was degassed with N_2 then stirred under 23 W blue LED irradiation (465 nm) for 72 h. Then, the solvent was removed under vacuum and the residue purified via flash chromatography.

1-mmol Procedure:

A 20 mL dry schlenk tube, equipped with a stirring bar was charged with: acridinium **4a** (10 mg, 2.5 mol%), [Co(dmgH)$_2$(Py)(Cl)] (20.0 mg, 5 mol%), dry DCM (10 mL), 1 phenylcyclohexene **1a** (158.0 mg, 1.0 mmol) and butyric acid (880.0 mg, 10.0 mmol). The solution was degassed with N_2 then stirred under 23 W blue LED irradiation (465 nm) for 72 h. Then, the solvent was removed under vacuum and the residue purified via flash chromatography to obtain **5aa** in 40% yield (97.6 mg).

Optimized general procedure for the intermolecular process with PC 4c.

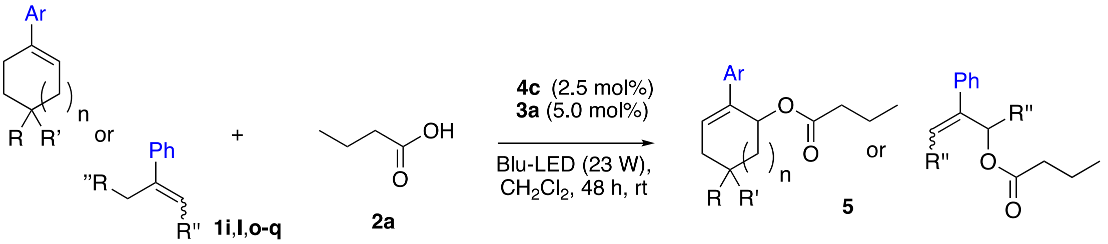

A 5 mL dry vial equipped with a stirring bar was charged with: acridinium **4c** (1.4 mg, 2.5 mol%), [Co(dmgH)$_2$(Py)(Cl)] (2.0 mg, 5 mol%), dry DCM (1 mL), styryl derivative **1** (0.1 mmol) and the desired carboxylic acid (1.0 mmol). The solution was degassed with N_2 then stirred under 23 W blue LED irradiation (465 nm) for 48 h. Then, the solvent was removed under vacuum and the residue purified via flash chromatography.

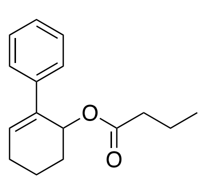

5aa. Viscous oil. *c*Hex:EtOAc: 100:1. Yield = 63% (15.2 mg). **^{1}H NMR** (400 MHz, $CDCl_3$) δ 7.32 – 7.25 (m, 4H), 7.22 – 7.18 (m, 1H), 6.31 (dd, *J* = 4.6 Hz, *J* = 3.4 Hz, 1H), 5.94 (t, *J* = 3.9 Hz, 1H), 2.35 – 2.27 (m, 1H), 2.23 – 2.19 (m, 1H), 2.17 – 2.12 (m, 2H), 2.00 – 1.94 (m, 1H), 1.90 – 1.82 (m, 1H), 1.76 – 1.67 (m, 2H), 1.49 (sext, *J* = 7.4 Hz, 2H), 0.79 (t, *J* = 7.4 Hz, 3H); **^{13}C NMR** (100 MHz, $CDCl_3$) δ 173.32, 139.61, 135.70, 130.78, 128.25, 126.98, 125.58, 67.16, 36.52, 29.08, 25.85, 18.50, 17.63, 13.41; **GC-MS**: 156 (100), 141 (18), 128 (19), 115 (21), 91 (23); **Anal. Calc.** for ($C_{16}H_{20}O_2$: 244.15): C, 78.65; H, 8.25; found: C, 78.76; H, 8.37.

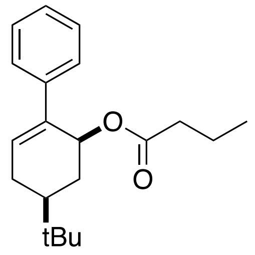

5ba. *dr* = 60:40. Viscous oil. *c*Hex:EtOAc: 100:1. Yield = 71% (21.3 mg). **^{1}H NMR** (400 MHz, $CDCl_3$) δ 7.35 - 7.34 (m,1H), 7.30 - 7.14 (m,4H), 6.40 (dd, *J* = 5.7 Hz, *J* = 2.4 Hz, 1H), 6.00 (m, 1H), 2.38 – 2.31 (m, 1H), 2.29 – 2.21 (m, 1H), 2.20 – 2.16 (m, 2H), 2.12 – 1.97 (m, 1H), 1.61-1.34 (m, 4H), 0.89 (s, 9H), 0.83 (t, *J* = 7.4 Hz, 3H); **^{13}C NMR** (100 MHz, $CDCl_3$) δ 173.44, 173.36, 139.42, 139.14, 138.05, 134.47, 131.31, 129.95, 128.35, 128.04, 127.07, 126.68, 125.94, 125.43, 70.94, 68.05, 42.81, 38.00, 36.63, 36.41, 32.20, 31.64, 30.85, 30.48, 27.76, 27.10, 27.03, 18.69, 18.28, 13.47. **GC-MS**: 155 (100), 212 (17), 115 (14), 128 (11), 91 (11); **Anal. Calc.** for ($C_{20}H_{28}O_2$: 300,44): C, 79.96; H, 9.39; found: C, 80.15; H, 9.56. Diagnostic signal of the minor diastereoisomer: **^{1}H NMR** (401 MHz, $CDCl_3$) δ 6.09 – 6.07 (m, 2H), 0.90 (s, 9H) 0.73 (t, *J* = 7.4 Hz, 3H).

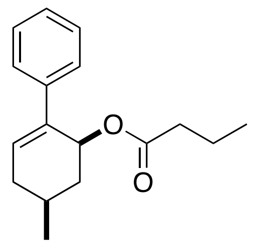

5ca. *dr* = 68:32. Viscous oil. *c*Hex:EtOAc: 100:1. Yield = 51% (13.2 mg); **^{1}H NMR** (400 MHz, $CDCl_3$) δ 7.34 – 7.32 (m, 1H), 7.29 – 7.19 (m, 4H), 6.34 (dd, *J* = 5.5 Hz, *J* = 2.4 Hz, 1H), 5.97 – 5.94 (m, 1H), 2.41 – 2.34 (m, 1H), 2.30 – 2.21 (m, 1H), 2.19 – 2.15 (m, 2H), 2.06 – 1.78 (m, 1H),1.63 - 1.37 (m, 4H), 1.02 (d, *J* = 4.3 Hz, 3H), 0.79 (t, *J* = 7.4 Hz, 3H); **^{13}C NMR** (100 MHz, $CDCl_3$) δ 173.32, 139.48, 139.30, 137.65, 134.85, 130.70, 129.50, 128.31, 128.05, 127.06, 126.74, 125.94, 125.53, 69.57, 67.67, 37.43, 37.38, 36.55, 36.40, 34.65, 34.18, 27.78, 23.39, 21.51, 21.34, 18.54, 18.32, 13.45, 13.37; **GC-MS**: 170 (100), 155 (74), 91 (5), 115 (21); **Anal. Calc.** for ($C_{17}H_{22}O_2$: 258,16): C, 79.03; H, 8.58; found: C, 79.26; H, 8.67. Diagnostic signal of the minor diastereoisomer: **^{1}H NMR** (401 MHz, $CDCl_3$) δ 6.09 – 6.05 (m, 2H), 1.07(d, *J* = 4.3 Hz, 3H), 0.74 (t, *J* = 7.4 Hz, 3H).

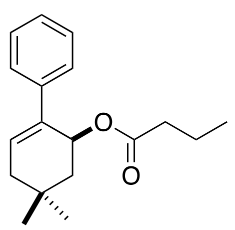

5da. Viscous oil. *c*Hex:EtOAc: 100:1. Yield = 41% (11.2 mg). **^{1}H NMR** (400 MHz, $CDCl_3$) δ 7.27 – 7.26 (m, 4H), 7.23 – 7.17 (m, 1H), 6.12 (dd, *J* = 5.8 Hz, *J* = 2.4 Hz, 1H), 6.05 – 6.02 (t, *J* = 5.6 Hz, 1H), 2.11 – 2.03 (m, 4H), 1.93 (dd, *J* = 13.4 Hz, *J* = 6.0 Hz, 1H), 1.62 (dd, *J* = 13.4 Hz, *J* = 6.7 Hz, 1H), 1.43 (sext, *J* = 7.4 Hz, 2H), 1.04 (s, 3H), 1.02 (s, 3H), 0.75 (t, *J* = 7.4 Hz, 3H); **^{13}C NMR** (100 MHz, $CDCl_3$) δ 173.42, 139.31, 135.58, 128.98, 128.16, 126.86, 125.84, 68.04, 41.70, 39.81, 36.46, 29.95, 29.45, 27.69, 18.36, 13.46; **GC-MS**: 184 (100), 154 (65), 91 (14): **Anal. Calc.** for ($C_{18}H_{24}O_2$: 272,18): C, 79.37; H, 8.88; found: C, 79.51; H, 9.17.

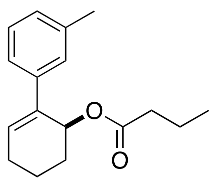

5ea. Viscous oil. *c*Hex:EtOAc: 100:1. Yield = 43% (11.1 mg). **^{1}H NMR** (400 MHz, $CDCl_3$) δ 7.18 – 7.09 (m, 3H), 7.02 (d, *J* = 7.3 Hz, 1H), 6.31 - 6.29 (dd, *J* = 4.7 Hz, *J* = 3.5 Hz, 1H), 5.92 – 5.90 (t, *J* = 3.9 Hz, 1H), 2.31 (s, 3H), 2.29 - 2.26 (m, 1H), 2.22 – 2.18 (m, 1H), 2.17 – 2.13 (m, 2H), 2.00 - 1.94 (m, 1H), 1.89 – 1.81 (m, 1H), 1.75 - 1.66 (m, 2H), 1.50 (sext, *J* = 7.4 Hz, 2H), 0.80 (t, *J* = 7.4 Hz, 3H); **^{13}C NMR** (100 MHz, $CDCl_3$) δ 173.34, 139.60, 137.72, 135.71, 130.62, 128.14, 127.74, 126.83, 122.66, 67.27, 36.53, 29.06, 25.84, 21.45, 18.52, 17.62, 13.42; **GC-MS**: 170 (100), 155 (36), 105 (23); **Anal. Calc.** for ($C_{17}H_{22}O_2$: 258.16): C, 79.03; H, 8.58; found: C, 79.27; H, 8.73.

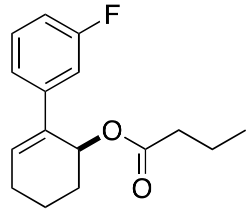

5fa. Yellow viscous oil. *c*Hex:EtOAc: 100:1. Yield = 42% (11.0 mg). **^{1}H NMR** (400 MHz, $CDCl_3$) δ 7.25 – 7.20 (m, 1H), 7.08 – 7.06 (m, 1H), 7.02 – 7.00 (m, 1H), 6.92 - 6.87 (m, 1H), 6.33 (dd, *J* = 4.7 Hz, *J* = 3.3 Hz, 1H), 5.87 (t, *J* = 4.0 Hz,1H), 2.35 - 2.27 (m, 1H), 2.23 – 2.19 (m, 1H), 2.18 – 2.14 (m, 2H), 2.00 - 1.94 (m, 1H), 1.89 – 1.81 (m, 1H), 1.74 - 1.67 (m, 2H), 1.49 (sext, *J* = 7.4 Hz, 2H), 0.80 (t, *J* = 7.4 Hz, 3H); **^{13}C NMR** (100 MHz, $CDCl_3$) δ 173.26, 162.89 (d, *J* = 245 Hz, 1C), 142.03 (d, *J* = 7.7 Hz, 1C), 134.86 (d, *J* = 2.0 Hz, 1C), 131.91, 129.65 (d, *J* = 8.4 Hz, 1C), 121.15 (d, *J* = 2.7 Hz, 1C), 113.78 (d, *J* = 21.1 Hz, 1C), 112.62 (d, *J* = 22.1 Hz, 1C), 67.00, 36.49, 28.97, 25.82, 18.50, 17.51, 13.40; **^{19}F NMR** (377 MHz, $CDCl_3$) δ -113.58 - 113.65 (m, 1F) ; **GC-MS**: 174 (100), 146 (18), 109 (23); **Anal. Calc.** for ($C_{16}H_{19}FO_2$: 262.14): C, 73.26; H, 7.30; found: C, 73.44; H, 7.48.

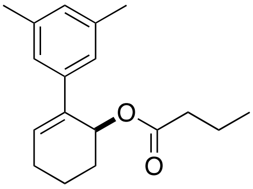

5ga. Viscous oil. *c*Hex:EtOAc: 100:1. Yield = 69% (18.8 mg). **^{1}H NMR** (400 MHz, $CDCl_3$) δ 6.93 (m, 2H), 6.86 (m, 1H), 6.28 (dd, *J* = 4.6 Hz, *J* = 3.4 Hz, 1H), 5.89 (t, *J* = 4.0 Hz, 1H), 2.34 – 2.29 (m, 1H), 2.27 (s, 6H), 2.22 - 2.19 (m,1H), 2.18 – 2.13 (m, 2H), 2.00 – 1.94 (m, 1H), 1.88 – 1.80 (m, 1H), 1.73 – 1.65 (m, 2H), 1.51 (sext, *J* = 7.2 Hz, 2H), 0.80 (t, *J* = 7.2 Hz, 3H); **^{13}C NMR** (100 MHz, $CDCl_3$) δ 173.37, 139.62, 137.60, 135.74, 130.49, 128.66, 123.50, 67.40, 36.55, 29.05, 25.84, 21.33, 18.56, 17.63, 13.43; **GC-MS**: 184 (100), 169 (39), 119 (18); **Anal. Calc.** for ($C_{18}H_{24}O_2$: 272,18): C, 79.37; H, 8.88; found: C, 80.21; H, 9.17.

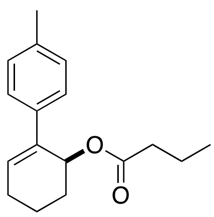

5ha. Viscous oil. *c*Hex:EtOAc: 100:1. Yield = 49% (12.6 mg). **^{1}H NMR** (400 MHz, $CDCl_3$) δ 7.20 (d, *J* = 8.0 Hz, 2H), 7.08 (d, *J* = 8.0 Hz, 2H), 6.28 (dd, *J* = 4.7 Hz, *J* = 3.5 Hz,1H), 5.91 (m, 1H), 2.30 (s, 3H), 2.32 – 2.25 (m, 1H), 2.22 – 2.19 (m, 1H), 2.17 – 2.13 (m, 2H), 2.00 – 1.94 (m, 1H), 1.88 – 1.79 (m, 1H), 1.74 – 1.67 (m, 2H), 1.51 (sext, *J* = 7.4 Hz, 2H), 0.80 (t, *J* = 7.2 Hz, 3H); **^{13}C NMR** (100 MHz, $CDCl_3$) δ 173.35, 136.69, 136.64, 135.35, 129.97, 128.96, 125.37, 67.14, 36.53, 29.08, 25.83, 20.99, 18.51, 17.56, 13.43; **GC-MS**: 170 (100), 155 (34), 105 (21); **Anal. Calc.** for ($C_{17}H_{22}O_2$: 258.16): C, 79.03; H, 8.58; found: C, 79.23; H, 8.75.

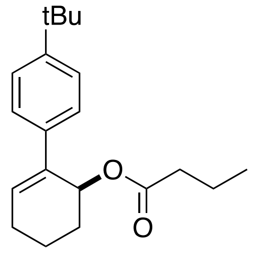

5ia. Viscous oil. *c*Hex:EtOAc: 100:1. Yield = 41% (12.3 mg). **^{1}H NMR** (400 MHz, $CDCl_3$) δ 7.30 – 7.28 (m, 2H), 7.25 – 7.23 (m, 2H), 6.30 (dd,

J = 4.7 Hz, *J* = 3.3 Hz,1H), 5.92 (t, *J* = 3.7 Hz ,1H), 2.34 – 2.26 (m, 1H), 2.22 – 2.14 (m, 3H), 2.02 – 1.94 (m, 1H), 1.76 – 1.68 (m, 1H), 1.49 (sext, *J* = 7.4 Hz, 2H), 1.28 (s, 9H), 0.77 (t, *J* = 7.4 Hz, 3H); **^{13}C NMR** (100 MHz, $CDCl_3$) δ 173.37, 149.90, 136.64, 135.29, 129.98, 125.16, 125.15, 67.19, 36.58, 34.38, 31.25, 29.08, 25.83, 18.53, 17.57, 13.38; **GC-MS**: 212 (97), 197 (100), 155 (48); **Anal. Calc.** for ($C_{20}H_{28}O_2$: 300.21): C, 79.96; H, 9.39; found: C, 80.45; H, 9.77.

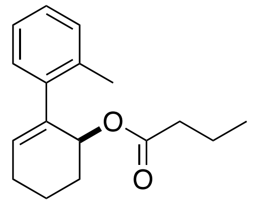

5la Viscous oil. *c*Hex:EtOAc: 100:1. Yield = 39% (10.1 mg). **^{1}H NMR** (400 MHz, $CDCl_3$) δ 7.12 - 7.10 (m, 2H), 7.09 – 7.05 (m, 1H), 7.05 – 7.04 (m, 1H), 5.79 (t, *J* = 3.8 Hz, 1H), 5.77 (t, *J* = 4.5 Hz, 1H), 2.25 – 2.21 (m, 1H), 2.26 (s, 3H), 2.19 – 2.12 (m, 1H), 2.11 – 2.02 (m, 2H), 1.94 – 1.88 (m, 2H), 1.80 – 1.70 (m, 2H), 1.46 – 1.37 (m, 2H), 0.71 (t, *J* = 7.4 Hz, 3H); **^{13}C NMR** (100 MHz, $CDCl_3$) δ 173.00, 140.68, 137.02, 135.76, 131.54, 129.76, 129.06, 126.84, 125.15, 69.46, 36.46, 29.01, 25.30, 19.85, 18.35, 18.19, 13.37; **GC-MS**: 187 (8), 170 (100), 155 (40), 142 (78); **Anal. Calc.** for ($C_{17}H_{22}O_2$: 258.16): C, 79.03; H, 8.58; found: C, 79.23; H, 8.94.

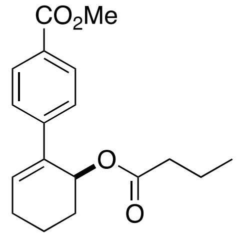

5ma. Yellow viscous oil. *c*Hex:EtOAc: 80:1. Yield = 46% (13.9 mg). **^{1}H NMR** (400 MHz, $CDCl_3$) δ 7.94 (m, 2H), 7.39 (m, 2H), 6.42 (dd, *J* = 4.6 Hz, *J* = 3.5 Hz, 1H), 5.95 (t, *J* = 4.1 Hz , 1H), 3.88 (s, 3H), 2.38 – 2.29 (m, 1H), 2.26 – 2.20 (m, 1H), 2.16 – 2.11 (m, 2H), 2.00 – 1.94 (m, 1H), 1.91 - 1.84 (m, 1H), 1.75 – 1.68 (m, 2H), 1.49 (sext, *J* = 7.4 Hz, 2H), 0.79 (t, *J* = 7.4 Hz, 3H); **^{13}C NMR** (100 MHz, $CDCl_3$) δ 173.25, 166.93, 144.12, 135.15, 132.92, 129.64, 128.62, 125.46, 66.82, 51.98, 36.44, 28.97, 25.96, 18.47, 17.55, 13.44: **GC-MS**: 214 (100), 155 (44), 115 (13); **Anal. Calc.** for ($C_{18}H_{22}O_4$: 302.15): C, 71.50; H, 7.33; found: C, 71.76; H, 7.48.

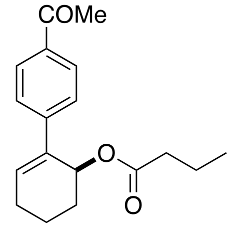

5na. Yellow viscous oil. *c*Hex:EtOAc: 80:1. Yield = 35% (10.0 mg). **^{1}H NMR** (400 MHz, $CDCl_3$) δ 7.87 (m, 2H), 7.41 (m, 2H), 6.44 (dd, *J* = 4.7 Hz, *J* = 3.4 Hz, 1H), 5.96 (t, *J* = 4.0 Hz, 1H), 2.57 (s, 3H), 2.39 – 2.30 (m, 1H), 2.27 – 2.19 (m, 1H), 2.16 - 2.12 (m, 2H), 2.00 – 1.95 (m, 1H), 1.92 - 1.84 (m, 1H), 1.78 – 1.69 (m, 2H), 1.50 (sext, *J* = 7.4 Hz, 2H), 0.81 (t, *J* = 7.4 Hz, 3H); **^{13}C NMR** (100 MHz, $CDCl_3$) δ 197.83, 173.44, 144.49, 135.88, 135.28, 133.33, 128.66, 125.83, 66.95, 36.63, 29.16, 26.72, 26.18, 18.66, 17.70, 13.63; **GC-MS**: 198 (100), 183 (48), 155 (34), 115 (15); **Anal. Calc.** for ($C_{18}H_{22}O_3$: 286.16): C, 75.50; H, 7.74; found: C, 75.65; H, 7.92.

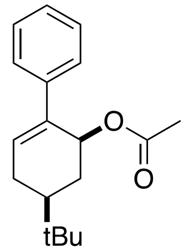

5bb. *dr* = 65:35. Viscous oil. *c*Hex:EtOAc: 100:1. Yield = 68% (18.5 mg). **^{1}H NMR** (400 MHz, $CDCl_3$) δ 7.35 – 7.34 (m, 1H), 7.31 – 7.20 (m, 4H), 6.40 (dd, *J* = 5.6 Hz, *J* = 2.3 Hz, 1H), 5.98 (m, 1H), 2.38 – 2.23 (m, 2H), 2.15 – 1.94 (m, 1H), 1.96 (s, 3H), 1.64 - 1.36 (m, 2H), 0.89 (s, 9H); **^{13}C NMR** (100 MHz, $CDCl_3$) δ 170.93, 170.74, 139.33, 139.13, 137.81, 134.36, 131.55, 130.10, 128.40, 128.08, 127.11, 127.71, 125.83, 125.40, 71.26, 68.38, 42.78, 37.95, 32.20, 31.62, 30.82, 30.37, 27.75, 27.09, 27.02, 26.68, 21.31, 21.09: **GC-MS**: 155 (100), 212 (14), 115 (12), 128 (9), 91 (10): **Anal. Calc.** for ($C_{18}H_{24}O_2$: 272.18): C, 79.37; H, 8.88;

found: C, 79.52; H, 9.07. Diagnostic signal of the minor diastereoisomer: **^{1}H NMR** (401 MHz, $CDCl_3$) δ 6.10 - 6.04 (m, 2H), 1.82 (s, 3H), 0.90 (s, 9H)

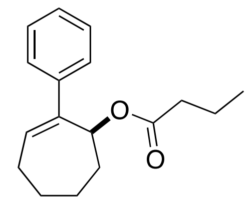

5oa. Viscous oil. *c*Hex:EtOAc: 100:1. Yield = 43% (11.1 mg). **^{1}H NMR** (400 MHz, $CDCl_3$) δ 7.28 – 7.25 (m, 4H), 7.21 – 7.17 (m, 1H), 6.12 (t, *J* = 6.7 Hz, 1H), 5.84 (d, *J* = 7.8 Hz, 1H), 2.52 – 2.44 (m, 1H), 2.30 – 2.24 (m, 1H), 2.23 – 2.17 (m, 2H), 2.15 – 2.07 (m, 1H), 1.96 – 1.91 (m, 1H), 1.84 – 1.77 (m, 2H), 1.74 – 1.67 (m, 1H), 1.64 – 1.59 (m, 1H), 1.58 – 1.50 (m, 2H), 0.85 (t, , *J* = 7.4 Hz, 3H). **^{13}C NMR** (100 MHz, $CDCl_3$) δ 172.79, 143.18, 142.34, 134.03, 127.98, 126.65, 126.55, 74.33, 36.43, 30.77, 27.50, 26.55, 25.47, 18.41, 13.58. **GC-MS**: 170 (91), 155 (50), 142 (100), 129 (38); **Anal. Calc.** for ($C_{17}H_{22}O_2$: 258.16): C, 79.03; H, 8.58; found: C, 79.48; H, 8.79.

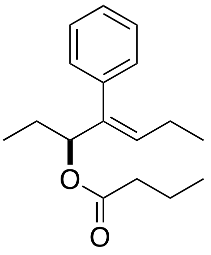

5pa. Viscous oil. *c*Hex:EtOAc: 100:1. *E*:*Z* = 53:47. Yield = 42% (10.9 mg). **^{1}H NMR** (400 MHz, $CDCl_3$) diagnostic signal for *Z*-**5pa** δ 5.77 (t, *J* = 7.2 Hz, 1H); diagnostic signal isomer *E*-**5pa** δ 5.33 (t, *J* = 6.6 Hz, 1H); sum of the other signals of the two isomers δ 7.37 - 7.25 (m, 8H), 7.17 – 7.15 (m, 2H), 5.67 (t, *J* = 6.9 Hz, 1H), 5.64 (t, *J* = 7.2 Hz, 1H), 2.43 -2.26 (m, 6H), 1.92 - 1.85 (m, 2H), 1.71 – 1.60 (m, 4H) 1.60 – 1.50 (m, 4H), 1.04 (t, *J* = 8.4 Hz, 3H), 0.96 – 0.80 (m, 15H); **^{13}C NMR** (100 MHz, $CDCl_3$) δ 172.99, 172.93, 141.26, 138.94, 138.19, 138.10, 136.56, 131.99, 129.20, 128.20, 127.94, 127.83, 126.85, 126.72, 78.99, 73.90, 36.61, 36.52, 26.36, 26.24, 21.86, 21.52, 18.50, 18.43, 14.28, 14.24, 13.67, 13.63, 10.07, 9.82; **GC-MS**: 190 (23), 172 (70), 157 (100), 143 (74); **Anal. Calc.** for ($C_{17}H_{24}O_2$: 260.18): C, 78.42; H, 9.29; found: C, 79.56; H, 9.41. The signals of the two isomers were assigned by the 1D-NOESY experiment.

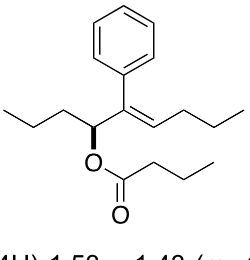

5qa. Viscous oil. *c*Hex:EtOAc: 100:1. *E*:*Z* = 50:50. Yield = 52% (15.0 mg). **^{1}H NMR** (400 MHz, $CDCl_3$) diagnostic signal isomer *Z*-**5qa** δ 5.87 (dd, *J* = 8.1 Hz, *J* = 6.5 Hz, 1H); diagnostic signal isomer *E*-**5qa** δ 5.40 (t, *J* = 6.7 Hz, 1H); sum of the other signals of the two isomers δ 7.37 - 7.22 (m, 8H), 7.17 – 7.15 (m, 2H), 5.67 (t, *J* = 7.5 Hz, 1H), 5.62 (t, *J* = 7.5 Hz, 1H), 2.36 -2.26 (m, 6H), 1.88 - 1.82 (m, 2H), 1.69 – 1.62 (m, 4H) 1.53 – 1.43 (m, 4H), 1.40 – 1.19 (m, 8H), 0.97 – 0.90 (m, 9H), 0.85 – 0.77 (m, 9H); **^{13}C NMR** (100 MHz, $CDCl_3$) δ 172.98, 172.91, 141.40, 140.03, 139.07, 138.26, 134.65, 130.20, 129.29, 128.19, 127.92, 127.83, 126.81, 126.71, 77.74, 72.35, 36.62, 36.52, 35.50, 35.50, 30.41, 30.15, 22.92, 22.75, 18.94, 18.81, 18.49, 18.43, 13.84, 13.74, 13.69, 13.64, 13.63. **GC-MS**: 218 (18), 200 (51), 171 (100), 129 (68); **Anal. Calc.** for ($C_{19}H_{28}O_2$: 288.21): C, 79.12; H, 9.79; found: C, 79.43; H, 10.07. The signals of the two isomers were assigned by the 1D-NOESY experiment.

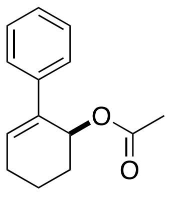

5ab. Viscous oil. *c*Hex:EtOAc: 100:1. Yield = 58% (12.5 mg). **^{1}H NMR** (400 MHz, $CDCl_3$) δ 7.33 – 7.26 (m, 4H), 7.24 - 7.19 (m,1H), 6.32 (dd, *J* = 4.8 Hz, *J* = 3.3 Hz , 1H), 5.93 – 5.91 (t, *J* = 4.0 Hz, 1H), 2.35 – 2.27 (m, 1H), 2.24 – 2.19 (m, 1H), 2.03 – 1.96 (m, 1H), 1.93 (s, 3H), 1.89 - 1.81 (m,1H), 1.79 -

1.66 (m, 2H): **^{13}C NMR** (100 MHz, $CDCl_3$) δ 170.76, 139.55, 135.45, 131.04, 128.32, 127.03, 125.50, 67.03, 28.97, 25.86, 21.28, 17.52; **GC-MS**: 156 (100), 141 (19), 128 (22), 115 (24), 91 (19): **Anal. Calc.** for ($C_{14}H_{16}O_2$: 216.12): C, 77.75; H, 7.46; found: C, 77.90; H, 7.62.

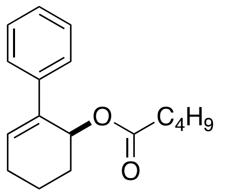

5ac. Viscous oil. *c*Hex:EtOAc: 100:1. Yield = 70% (18.1 mg). **^{1}H NMR** (400 MHz, $CDCl_3$) δ 7.32 – 7.29 (m, 2H), 7.29 – 7.24 (m, 2H), 7.23 – 7.18 (m, 1H), 6.31 (dd, *J* = 4.7 Hz, *J* = 3.4 Hz, 1H), 5.94 (t, *J* = 3.9 Hz , 1H), 2.35 – 2.27 (m, 1H), 2.23 – 2.20 (m, 1H), 2.19 – 2.14 (m, 2H), 2.00 – 1.94 (m, 1H), 1.90 – 1.82 (m, 1H), 1.78 – 1.67 (m, 2H), 1.44 (p, *J* = 7.5 Hz, 2H), 1.21 – 1.12 (m, 2H), 0.78 (t, *J* = 7.3 Hz, 3H); **^{13}C NMR** (100 MHz, $CDCl_3$) 173.52, 139.61, 135.70, 130.80, 128.26, 126.99, 125.99, 67.15, 34.37, 29.06, 27.08, 25.85, 22.03, 17.63, 13.61; **GC-MS**: 156 (100), 141 (19), 128 (23), 115 (27), 91 (29); **Anal. Calc.** for ($C_{17}H_{22}O_2$: 258.16): C, 79.03; H, 8.58; found: C, 79.24; H, 8.67.

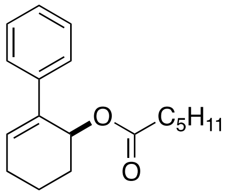

5ad. Viscous oil. *c*Hex:EtOAc: 100:1. Yield = 55% (15.0 mg). **^{1}H NMR** (400 MHz, $CDCl_3$) δ 7.32 – 7.25 (m, 4H), 7.22 – 7.19 (m, 1H), 6.31 (dd, *J* = 4.4 Hz, *J* = 3.7 Hz, 1H), 5.94 (t, *J* = 3. Hz, 1H), 2.35 – 2.27 (m, 1H), 2.22 – 2.19 (m, 1H), 2.18 – 2.13 (m, 2H), 2.00 – 1.94 (m, 1H), 1.90 – 1.82 (m, 1H), 1.76 – 1.68 (m, 2H), 1.50 - 1.42 (m, 2H), 1.22 - 1.08 (m, 4H), 0.80 (t, *J* = 7.1 Hz, 3H); **^{13}C NMR** (100 MHz, $CDCl_3$) δ 173.53, 139.60, 135.69, 130.80, 128.26, 126.99, 125.58, 67.13, 34.62, 31.07, 29.06, 25.85, 24.72, 22.21, 17.62, 13.80; **GC-MS**: 156 (100), 141 (19), 128 (21), 115 (26), 91 (27). **Anal. Calc.** for ($C_{18}H_{24}O_2$: 272.39): C, 79.37; H, 8.88; found: C, 79.54; H, 9.02.

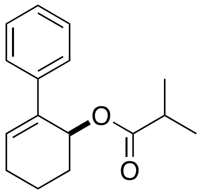

5ae. Viscous oil. *c*Hex:EtOAc: 100:1. Yield = 45% (11.0 mg). **^{1}H NMR** (400 MHz, $CDCl_3$) δ 7.31 – 7.25 (m, 4H), 7.22 – 7.18 (m, 1H), 6.30 (dd, *J* = 4.5 Hz, *J* = 3.5 Hz, 1H), 5.92 (t, *J* = 4.2 Hz, 1H), 2.39 (sept, *J* = 6.8 Hz , 1H), 2.33 – 2.26 (m, 1H), 2.23 – 2.17 (m, 1H), 1.98 – 1.91 (m, 1H), 1.91 – 1.83 (m, 1H), 1.74 – 1.68 (m, 2H), 1.02 (d, *J* = 6.8 Hz, 3H), 0.91 (d, *J* = 6.8 Hz, 3H); **^{13}C NMR** (100 MHz, $CDCl_3$) δ 176.78, 139.62, 135.88, 130.55, 128.21, 126.96, 125.61, 67.13, 34.08, 29.03, 25.83, 18.79, 18.75, 17.78; **GC-MS**: 156 (100), 141 (19), 128 (25), 115 (36), 91 (34); **Anal. Calc.** for ($C_{16}H_{20}O_2$: 244.33): C, 78.65; H, 8.25; found: C, 78.79; H, 8.37.

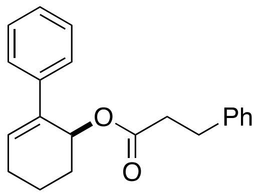

5af. Viscous oil. *c*Hex:EtOAc: 100:1. Yield = 58% (17.8 mg). **^{1}H NMR** (400 MHz, $CDCl_3$) δ 7.31 – 7.25 (m, 4H), 7.24 – 7.13 (m, 4H), 7.08 (d, *J* = 7.6 Hz, 2H), 6.32 (m, 1H), 5.94 (m, 1H), 2.81 (t, *J* = 7.8 Hz, 2H), 2.50 (t, *J* = 7.8 Hz, 2H), 2.35 – 2.26 (m, 1H), 2.22 – 2.14 (m, 1H), 1.96 – 1.89 (m, 1H), 1.79 - 1.87 (m, 1H), 1.69 – 1.63 (m, 2H); **^{13}C NMR** (100 MHz, $CDCl_3$) δ 172.57, 140.39, 139.58, 135.52, 130.99, 128.36, 128.32, 128.16, 127.03, 126.09, 125.56, 67.55, 36.03, 30.91, 28.99, 25.84, 17.53; **GC-MS**: 156 (100), 141 (16), 128 (18), 115 (22), 91 (57); **Anal. Calc.** for ($C_{21}H_{22}O_2$: 306.16): C, 82.32; H, 7.24; found: C, 82.49; H, 7.38.

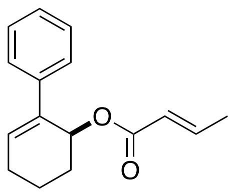

5ag. Viscous oil. *c*Hex:EtOAc: 100:1. Yield = 43% (10.4 mg). **^{1}H NMR** (400 MHz, $CDCl_3$) δ 7.34 – 7.31 (m, 2H), 7.29 – 7.25 (m, 2H), 7.22 - 7.18 (m, 1H), 6.87 (dq, *J* = 15.5 Hz, *J* = 6.9 Hz, 1H), 6.34 (dd, *J* = 4.8 Hz, *J* = 3.2 Hz, 1H), 5.96 (t, *J* = 3.8 Hz, 1H), 5.74 (dq, *J* = 15.5 Hz, *J* = 1.7 Hz, 1H), 2.36 – 2.28 (m, 1H), 2.24 – 2.14 (m, 1H), 2.05 – 1.99 (m, 1H), 1.90 – 1.82 (m, 1H), 1.79 (dd, *J* = 6.9 Hz, *J* = 1.7 Hz, 3H), 1.77 – 1.66 (m, 2H); **^{13}C NMR** (100 MHz, $CDCl_3$) δ 166.18, 144.54, 139.63, 135.45, 130.94, 128.30, 126.96, 125.46, 122.94, 67.12, 29.02, 25.91, 17.88, 17.50; **GC-MS**: 156 (100), 141 (20), 128 (27), 115 (31), 91 (29); **Anal. Calc.** for ($C_{16}H_{18}O_2$: 242.13): C, 79.31; H, 7.49; 13.20 found: C, 79.58; H, 7.74.

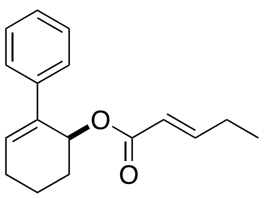

5ah. Viscous oil. *c*Hex:EtOAc: 100:1. Yield = 43% (11.0 mg). **^{1}H NMR** (400 MHz, $CDCl_3$) δ 7.34 – 7.25 (m, 4H), 7.22 – 7-18 (m, 1H), 6.93 (dt, *J* = 15.7 Hz, *J* = 6.3 Hz, 1H), 6.35 (dd, *J* = 4.4 Hz, *J* = 3.6 Hz, 1H), 5.97 (t, *J* = 3.6 Hz, 1H), 5.71 (d, *J* = 15.7 Hz,1H), 2.37 – 2.29 (m, 1H), 2.24 – 2.20 (m, 1H), 2.18 – 2.11 (m, 2H), 2.07 – 2.00 (m, 1H), 1.90 – 1.82 (m, 1H), 1.77 – 1.66 (m, 2H), 1.00 (t, *J* = 7.4 Hz, 3H); **^{13}C NMR** (100 MHz, $CDCl_3$) δ 166.48, 150.77, 139.63, 135.44, 130.95, 128.30, 126.96, 125.46, 120.48, 67.13, 29.01, 25.92, 25.20, 17.50, 11.97; **GC-MS**: 156 (100), 141 (19), 128 (25), 115 (30), 91 (28); **Anal. Calc.** for ($C_{17}H_{20}O_2$: 256.35): C, 79.65; H, 7.86; found: C, 79.89; H, 8.02.

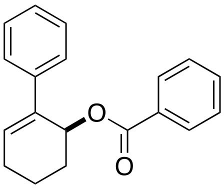

5ai. Viscous oil. *c*Hex:EtOAc: 100:1. Yield = 45% (12.5 mg). **^{1}H NMR** (400 MHz, $CDCl_3$) δ 7.92 (d, *J* = 7.2 Hz, 2H), 7.51 – 7.46 (m, 1H), 7.39 – 7.33 (m, 4H), 7.27 – 7.24 (m, 2H), 7.20 - 7.16 (m, 1H), 6.40 (dd, *J* = 4.7 Hz, *J* = 3.3 Hz, 1H), 6.14 (t, *J* = 3.6 Hz, 1H), 2.42 – 2.34 (m, 1H), 2.29 – 2.20 (m, 1H), 2.17 – 2.11 (m, 1H), 2.00 – 1,91 (m, 1H), 1.88 – 1.79 (m, 1H), 1.79 – 1.70 (m, 1H); **^{13}C NMR** (100 MHz, $CDCl_3$) δ 166.22, 139.76, 135.51, 132.70, 131.15, 130.61, 129.57, 128.34, 128.19, 127.0, 125.54, 68.26, 29.09, 25.95, 17.61; **GC-MS**: 156 (100), 141 (19), 128 (22), 115 (24), 91 (22), 77 (53). **Anal. Calc.** for ($C_{19}H_{18}O_2$: 278.13): C, 81.99; H, 6.52; found: C, 82.12; H, 6.67.

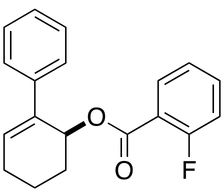

5aj. Viscous oil. *c*Hex:EtOAc: 100:1. Yield = 71% (21.0 mg). **^{1}H NMR** (400 MHz, $CDCl_3$) δ 7.70 (td, *J* = 7.6 Hz, *J* = 1.8 Hz ,1H), 7.45 – 7.41 (m, 1H), 7.39 – 7.37 (m, 2H), 7.29 – 7.25 (m, 2H), 7.21 – 7.18 (m, 1H), 7.10 – 7.02 (m, 2H), 6.38 (dd, *J* = 4.8 Hz, *J* = 3.3 Hz, 1H), 6.17 (t, *J* = 3.7 Hz, 1H), 2.41 – 2.33 (m, 1H), 2.29 – 2.24 (m, 1H), 2.21 – 2.13 (m, 1H), 2.00 – 1.92 (m, 1H), 1.89 – 1.80 (m, 1H), 1,80 – 1.71 (m, 1H); **^{13}C NMR** (100 MHz, $CDCl_3$) δ 163.91, 161.87 (d, *J* = 260 Hz, 1C) , 139.70, 135.37, 134.08 (d, *J* = 9.0 Hz, 1C), 131.83 (d, *J* = 1.0 Hz, 1C), 131.33, 128.33, 127.02, 125.61, 123.72 (d, *J* = 4.0 Hz, 1C), 119.25 (d, *J* = 10.0 Hz, 1C), 116.80 (d, *J* = 22.2 Hz, 1C), 68.62, 29.07, 25.92, 17.56; **^{19}F NMR** (376 MHz, $CDCl_3$) δ -109.95 - 110.01 (m, 1F); **GC-MS**: 156 (100), 141 (20), 123 (42), 91 (27); **Anal. Calc.** for ($C_{19}H_{17}FO_2$: 296.34): C, 77.01; H, 5.78; found: C, 77.35; H, 5.84.

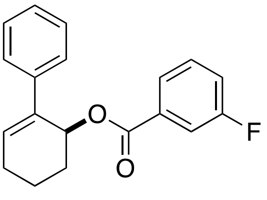

5ak. Viscous oil. *c*Hex:EtOAc: 100:1. Yield = 41% (12.1 mg). **^{1}H NMR** (400 MHz, $CDCl_3$) δ 7.71 – 7.69 (m, 1H), 7.59 – 7.56 (m, 1H),

7.36 – 7.25 (m, 5H), 7.20 – 7.15 (m, 2H), 6.39 (dd, *J* = 4.8 Hz, *J* = 3.3 Hz, 1H), 6.13 (t, *J* = 3.8 Hz, 1H), 2.42 – 2.34 (m, 1H), 2.29 – 2.20 (m, 1H), 2.16 – 2.10 (m, 1H), 2.00 – 1.92 (m, 1H), 1.83 – 1.70 (m, 2H); **^{13}C NMR** (100 MHz, $CDCl_3$) δ 165.10, 163.19 (d, *J* = 247 Hz, 1C), 139.64, 135.34, 132.76 (d, *J* = 7.4 Hz, 1C), 131.36, 129.79 (d, *J* = 7.8 Hz, 1C), 128.36, 127.07, 125.54, 125.28 (d, *J* = 3.0 Hz, 1C), 119.76 (d, *J* = 21.3 Hz, 1C), 116.43 (d, *J* = 22.9 Hz, 1C), 68.76, 29.04, 25.90, 17.59; **^{19}F NMR** (376 MHz, $CDCl_3$) δ -112.62 – 112.69 (m, 1F); **GC-MS**: 156 (100), 141 (21), 91 (26); **Anal. Calc.** for ($C_{19}H_{17}FO_2$: 296.12): C, 77.01; H, 5.78; found: C, 77.34; H, 5.86.

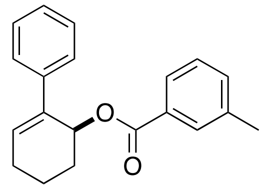

5al. Viscous oil. *c*Hex:EtOAc: 100:1. Yield = 55% (16.1 mg). **^{1}H NMR** (400 MHz, $CDCl_3$) δ 7.71 (d, *J* = 7.3 Hz, 2H), 7.38 – 7.35 (m, 2H), 7.30 – 7.22 (m, 4H), 7.21 – 7.15 (m, 1H), 6.40 (dd, *J* = 4.8 Hz, *J* = 3.3 Hz, 1H), 6.13 (t, *J* = 3.7 Hz,1H), 2.42 – 2.34 (m, 1H), 2.33 (s, 3H), 2.29 – 2.19 (m, 1H), 2.18 – 2.10 (m, 1H), 1.98 – 1.90 (m, 1H), 1.88 – 1.60 (m, 2H); **^{13}C NMR** (100 MHz, $CDCl_3$) δ 166.40, 139.77, 137.97, 135.52, 133.46, 131.09, 130.54, 130.07, 128.32, 128.08, 126.97, 126.71, 125.52, 68.17, 29.10, 25.96, 21.18, 17.62; **GC-MS**: 156 (100), 141 (19), 128 (20), 115 (25), 91 (59); **Anal. Calc.** for ($C_{20}H_{20}O_2$: 292.15): C, 82.16; H, 6.90; found: C, 82.32; H, 7.21.

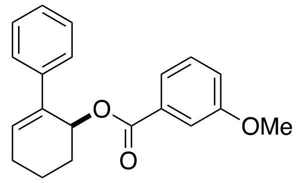

5am.Viscous oil. *c*Hex:EtOAc: 80:1. Yield = 45% (13.9 mg). **^{1}H NMR** (400 MHz, $CDCl_3$) δ 7.50 (dd, *J* = 7.6 Hz, *J* = 1.0 Hz, 1H), 7.42 – 7.41 (m, 1H), 7.36 (d, *J* = 7.6 Hz, 2H), 7.26 (d, *J* = 8.0 Hz, 3H), 7.24 – 7.23 (m, 1H), 7.03 – 7.00 (m, 1H) 6.38 – 6.36 (m, 1H), 6.12 – 6.10 (m, 1H), 3.77 (s, 3H), 2.41 – 2.33 (m, 1H), 2.28 – 2.18 (m, 1H), 2.15 – 2.09 (m, 1H), 2.00 – 1.92 (m, 1H), 1.87 – 1.70 (m, 2H); **^{13}C NMR** (100 MHz, $CDCl_3$) δ 166.11, 159.40, 139.81, 135.58, 131.94, 131.13, 129.20, 128.32, 126.98, 125.58, 121.97, 119.18, 114.05, 68.49, 55.34, 29.08, 25.93, 17.68; **GC-MS**: 156 (100), 141 (20), 128 (20), 115 (23), 91 (24); **Anal. Calc.** for ($C_{20}H_{20}O_3$: 308.38): C, 77.90; H, 6.54; found: C, 78.13; H, 6.73.

Synthesis of substrates 6a-d

Synthesis of the enol triflates through the procedures reported in the literatures.[2]

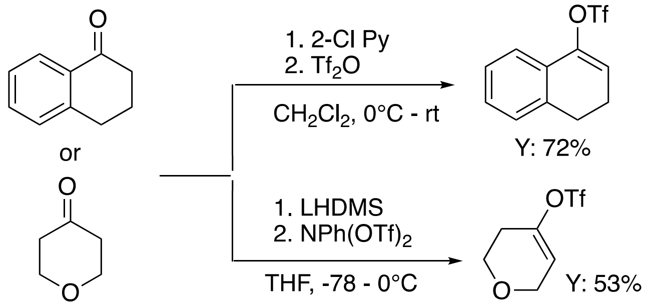

The enol triflates were subsequently converted into the corresponding acids **6** using the following procedure.

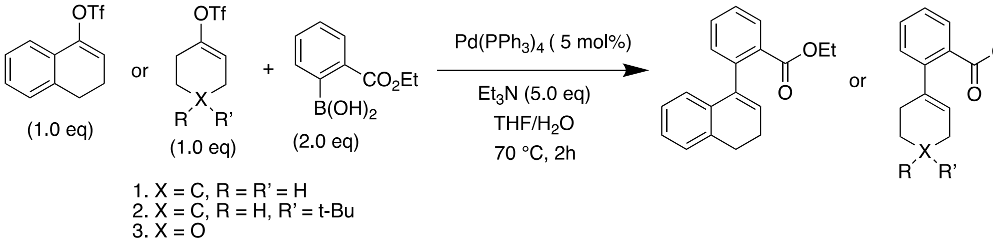

Under N_2, a dry Schlenk tube was charged with reagent grade THF (5 mL), deionized H_2O (1 mL) and Et_3N (5.0 eq). Then (2-(ethoxycarbonyl) phenylboronic acid (2.0 eq, 2.0 mmol, 386 mg) and triflate (1 mmol, 1.0 eq) was added. The reaction was stirred at 70 °C for 2 hours or until completion (TLC). Then H_2O was added, and the resulting mixture was extracted with EtOAc (2 x 15 mL). The organic phase was dried over Na_2SO_4 and concentrated under vacuum. The residue was purified with gel chromatography.

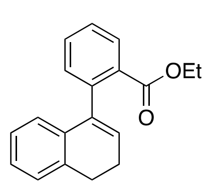

Viscous oil. *c*Hex:EtOAc: 40:1. Yield = 95% (264 mg). **^{1}H NMR** (400 MHz, $CDCl_3$) δ 7.91 (dd, dd, *J* = 7.2 Hz, *J* = 1.2 Hz, 1H), 7.52 (td, *J* = 7.5 Hz, *J* = 1.4 Hz ,1H), 7.41 (td, *J* = 7.8 Hz, *J* = 1.3 Hz ,1H), 7.34 (dd, *J* = 7.6 Hz, *J* = 1.0 Hz, 1H), 7.18 (dd, *J* = 7.3 Hz, *J* = 0.8 Hz ,1H), 7.12 (td, *J* = 7.4 Hz, *J* = 1.3 Hz ,1H), 7.04 (td, *J* = 7.5 Hz, *J* = 1.4 Hz, 1H), 6.68 (d, *J* = 7.5 Hz, 1H), 3.99 (bs, 3H), 2.94 - 2.88 (m, 2H), 2.44 (d, *J* = 5.2 Hz, 2H), 0.99 (t, *J* = 7.2 Hz, 3H); **^{13}C NMR** (100 MHz, $CDCl_3$) δ 167.93, 141.17, 139.98, 135.85, 135.64, 131.78, 131.41, 131.25, 129.94, 127.40, 127.31, 126.79, 126.69, 126.32, 124.31, 60.78, 28.16, 23.54, 13.72. **GC-MS**: 231 (100), 202 (44), 215 (15), 278 (13).

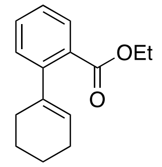

Viscous oil. *c*Hex:EtOAc: 40:1. Yield = 78% (179 mg). **^{1}H NMR** (400 MHz, $CDCl_3$) δ 7.71 (dd, *J* = 7.7 Hz, *J* = 1.1 Hz, 1H), 7.39 (td, *J* = 7.5 Hz, *J* = 1.4 Hz, 1H), 7.25 (td, *J* = 7.6 Hz, *J* = 1.2 Hz, 1H), 7.18 (dd, *J* = 7.7 Hz, *J* = 0.9 Hz, 1H), 5.55 – 5.52 (m, 1H), 4.29 (q, *J* = 7.1 Hz, 2H), 2.27 - 2.22 (m, 2H), 2.15 - 2.10 (m, 2H), 1.78 - 1.72 (m, 2H),), 1.69 - 1.63 (m, 2H), 1.34 (t, *J* = 7.2 Hz, 3H); **^{13}C NMR** (100 MHz, $CDCl_3$) δ 168.74, 145.32, 139.08, 131.01, 130.45, 129.44, 129.27, 126.33, 125.01, 60.84, 30.05, 25.47, 23.04, 21.97, 14.27; **GC-MS**: 184 (100), 165 (51), 128 (37), 115 (35), 230 (33).

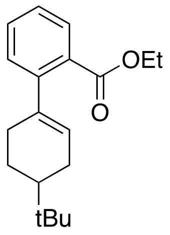

Viscous oil. *c*Hex:EtOAc: 40:1. Yield = 72% (206 mg). δ **^{1}H NMR** (400 MHz, $CDCl_3$) δ 7.71 (dd, *J* = 7.7 Hz, *J* = 0.8 Hz, 1H), 7.38 (td, *J* = 7.5 Hz, *J* = 1.4 Hz, 1H), 7.27- 7.23 (m ,1H), 7.18 (dd, *J* = 7.6 Hz, *J* = 0.7 Hz, 1H), 7.18 (dd, *J* = 7.3 Hz, *J* = 0.8 Hz ,1H), 5.56 - 5-54 (m, 1H), 4.33 - 4.25 (m, 2H), 2.36 – 2.29 (m, 2H), 2.19 – 2.14 (m, 1H), 1.94 - 1.86 (m, 2H), 1.42 – 1.36 (m, 1H), 1.34 (t, *J* = 7.1 Hz, 3H), 0.90 (s, 9H); **^{13}C NMR** (100 MHz, $CDCl_3$) δ 168.65, 145.07, 138.89, 131.04, 130.40, 129.46, 129.34, 126.34, 125.32, 60.83, 43.62, 32.22, 31.56, 27.20, 27.18, 24.44, 14.26; **GC-MS**: 165 (100), 183 (72), 57 (57), 286 (31).

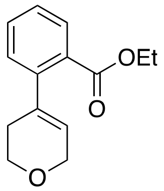

Viscous oil. *c*Hex:EtOAc: 10:1 Yield = 65% (151 mg). **^{1}H NMR** (400 MHz, $CDCl_3$) δ 7.79 (dd, *J* = 7.8 Hz, *J* = 1.2 Hz, 1H), 7.43 (td, *J* = 7.5 Hz, *J* = 1.3 Hz, 1H), 7.31 (td, *J* = 7.6 Hz, *J* = 1.2 Hz, 1H), 7.20 (dd, *J* = 7.8 Hz, *J* = 0.9 Hz, 1H), 5.56 – 5.55 (m, 1H), 4.30 (q, *J* = 7.1 Hz, 2H), 4.25 (q, *J* = 2.6 Hz, 2H), 2.37 - 2.34 (m, 2H) 1.35 (t, *J* = 7.1 Hz, 3H); **^{13}C NMR** (100 MHz, $CDCl_3$) δ 167.95, 144.48, 137.07, 131.49, 129.95, 129.87, 129.37, 127.02, 123.03, 65.55, 64.48, 61.01, 30.02, 14.25; **GC-MS**: 157 (100), 129 (89), 186 (87), 232 (4).

Hydrolysis of the ethyl esters

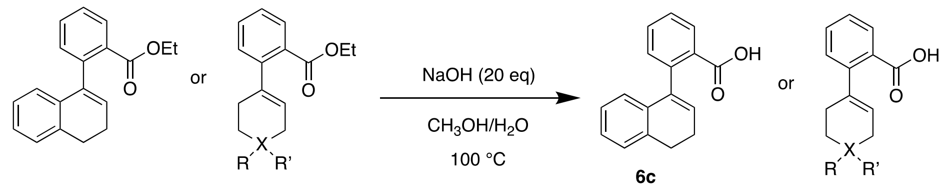

6c

1. X = C, R = R' = H
2. X = C, R = H, R' = t-Bu
3. X = O

6a, 6b, 6d

A one-necked flask was charged with MeOH (9 mL), H_2O (1 mL), the substrates obtained in the previous step and finally with NaOH (20 eq) finely pounded. The reaction was refluxed for 1 hours or until completion (TLC). The solution obtained was extracted with CH_2Cl_2 (2 x 15 mL). The aqueous layer was collected and HCl (6 M) was added to acidify the solution. Then EtOAc (2 x 15 mL) was used to extract the product. The organic layers were combined, dried over Na_2SO_4 and concentrated under vacuum.

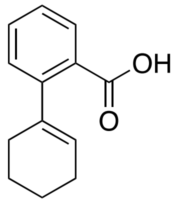

6a. White solid. Yield = 70% (125 mg). M.p. = 76 - 80 °C. **^{1}H NMR** (400 MHz, $CDCl_3$) δ 7.93 – 7.91 (m, 1H), 7.49 – 7.45 (m, 1H), 7.33 – 7.22 (m, 2H), 5.58 (m, 1H), 2.26 (m, 2H), 2.15 (m, 2H), 1.76 (m, 2H), 1.68 (m, 2H). **^{13}C NMR** (100 MHz, $CDCl_3$) δ 172.91, 146.68, 139.90, 132.30, 130.55, 129.87, 128.34, 126.54, 125.16, 30.34, 25.50, 23.04, 21.91. **GC-MS**: 184 (100), 128 (57), 115 (57), 202 (56), 91 (27). **Anal. Calc.** for ($C_{13}H_{14}O_2$: 202.25): C, 77.20; H, 6.98; found: C, 77.42; H, 7.14.

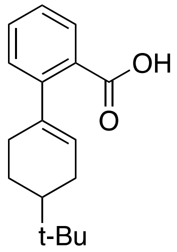

6b. Pale grey solid. Yield = 62% (128 mg). M.p. = 134 – 136 °C. **^{1}H NMR** (400 MHz, $CDCl_3$) δ 7.92 (dd, *J* = 7.8 Hz, *J* = 1.1 Hz, 1H), 7.46 (td, *J* = 7.5 Hz, *J* = 1.4 Hz, 1H), 7.30 (td, *J* = 7.7 Hz, *J* = 1.3 Hz, 1H), 7.20 (dd, *J* = 7.6 Hz, *J* = 1.0 Hz, 1H), 5.56 (m, 1H), 2.36 – 2.30 (m, 2H), 2.24 – 2.14 (m, 1H), 1.97 – 1.87 (m, 2H), 1.43 – 1.29 (m, 2H), 0.90 (s, 9H). **^{13}C NMR** (100 MHz, $CDCl_3$) δ 172.52, 144.26, 139.31, 132.28, 130.59, 129.44, 128.34, 126.56, 125.66, 43.57, 33.23, 31.81, 27.21, 24.45. **GC-MS**: 165 (100), 184 (94), 202 (58), 258 (42); **Anal. Calc.** for ($C_{17}H_{22}O_2$: 258.36): C, 79.03; H, 8.58; found: C, 79.31; H, 8.80.

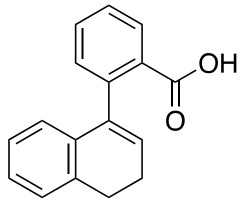

6c. White solid. Yield = 80% (211 mg). M.p. = 137 – 139 °C. **^{1}H NMR** (400 MHz, $CDCl_3$) δ 8.56 (bs, 1H), 7.94 (dd, *J* = 7.8 Hz, *J* = 1.2 Hz, 1H), 7.54 (td, *J* = 7.5 Hz, *J* = 1.3 Hz, 1H), 7.39 (td, *J* = 7.6 Hz, *J* = 1.1 Hz, 1H), 7.30 (dd, *J* = 7.6 Hz, *J* = 1.0 Hz, 1H), 7.13 (d, *J* = 7.2 Hz, 1H), 7.07 (td, *J* = 7.4 Hz, *J* = 1.1 Hz, 1H), 7.01 – 6.95 (m, 1H), 6.56 (d, *J* = 7.5 Hz, 1H), 5.88 (t, *J* = 4.6 Hz, 1H), 2.86 – 2.80 (m, 2H), 2.39 – 2.35 (m, 2H). **^{13}C NMR** (100 MHz, $CDCl_3$) δ 172.20, 142.32, 139.56, 135.87, 132.46, 131.42, 130.68, 129.87, 128.34, 127.28, 127.25, 126.70, 126.53, 126.18, 124.09, 27.99, 23.43. **GC-MS**: 178 (100), 231 (88), 250 (78); **Anal. Calc.** for ($C_{17}H_{14}O_2$: 250.10): C, 81.58; H, 5.64; found: C, 81.72; H, 5.82.

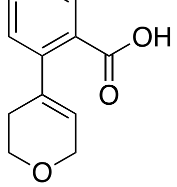

6d. Viscous oil. Yield = 59% (89.1 mg). **^{1}H NMR** (400 MHz, $CDCl_3$) δ 7.97 (dd, *J* = 7.8 Hz, *J* = 1.2 Hz, 1H), 7.50 (td, *J* = 7.5 Hz, *J* = 1.4 Hz, 1H), 7.35 (td, *J* = 7.6 Hz, *J* = 1.3 Hz, 1H), 7.23 (dd, *J* = 7.6 Hz, *J* = 1.2 Hz, 1H), 5.58 (m, 1H), 4.30 – 4.28 (m, 2H), 3.92 (t, *J* = 5.3 Hz, 2H), 2.40 – 2.37 (m, 2H). **^{13}C NMR** (100 MHz, $CDCl_3$) δ 172.28, 144.66, 137.33, 132.64, 130.91, 129.90, 128.19, 127.19, 122.81, 65.54, 64.51, 30.12. **GC-MS**: 157 (100), 129 (80), 146 (62), 105 (53) 202 (31). **Anal. Calc.** for ($C_{12}H_{12}O_3$: 204.08): C, 70.58; H, 5.92; found: C, 70.74; H, 6.25.

General procedure for intramolecular process.

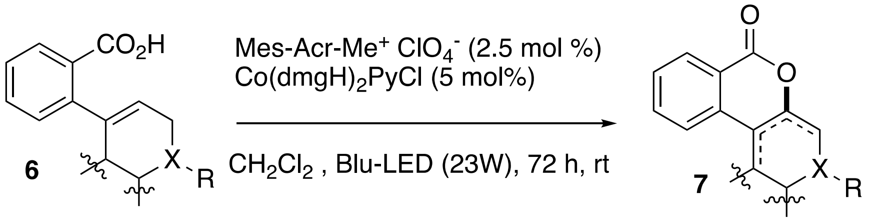

A 5 ml dry vial equipped with a stirring bar was charged with: Mes-Acr-$Me^+ClO_4^-$ (1.0 mg, 2.5 mol%), $[Co(dmgH)_2(Py)(Cl)]$ (2.0 mg, 5 mol%), dry DCM (1 ml) and acid (0.1 mmol). The solution was degassed with N_2 then stirred under 23 W blue LED irradiation (465 nm) for 72 h. The solvent was removed under vacuum and the residue purified by flash chromatography.

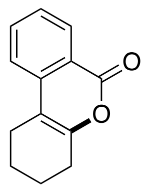

7a. White solid. *c*Hex:EtOAc: 20:1. Yield = 47% (9.4 mg). M.p. = 106 – 109 °C. **^{1}H NMR** (400 MHz, $CDCl_3$) δ 8.28 (dd, *J* = 8.3 Hz, *J* = 1.5 Hz,1H), 7.72 (ddd, *J* = 8.3 Hz, *J* = 7.2 Hz, *J* = 1.5 Hz, 1H), 7.46 – 7.42 (m, 2H), 2.61 – 2.55 (m, 4H), 1.91 - 1.78 (m, 4H). **^{13}C NMR** (100 MHz, $CDCl_3$) δ 162.7, 152.42, 138.03, 134.54, 129.73, 127.11, 121.33, 120.55, 109.30, 27.36, 22.65, 22.03. **GC-MS**: 144 (100), 200 (95), 115 (56), 102 (31). **Anal. Calc.** for ($C_{13}H_{12}O_2$: 200.08): C, 77.98; H, 6.04; found: C, 78.18; H, 6.28. Diagnostic signal of the minor isomer: **^{1}H NMR** (400 MHz, $CDCl_3$) δ 7.87 – 7.85 (m, 1H), 7.65- 7.61 (m, 1H), 7.51 – 7.47 (m, 1H), 7.37 – 7.35 (m, 1H), 6.21 - 6.16 (m, 1H), 5.46 – 5.44 (m, 1H).

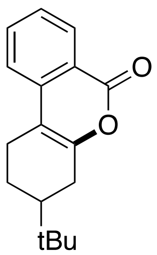

7b. Pale grey solid. *c*Hex:EtOAc: 50:1. Yield = 43% (11.0 mg). M.p. = 150 – 153 °C. **^{1}H NMR** (400 MHz, $CDCl_3$) δ 8.28 (dd, *J* = 7.8 Hz, *J* = 1.4 Hz, 1H), 7.70 (td, *J* = 7.7 Hz, *J* = 1.4 Hz, 1H), 7.46 – 7.42 (m, 2H), 2.79 – 2.74 (m, 1H), 2.59 – 2.52 (m, 1H), 2.52 – 2.44 (m, 1H), 2.44 – 2.34 (m, 1H), 2.11 – 2.05 (m,1H), 1.58 – 1.51 (m, 1H), 1.38 – 1.29 (m, 1H), 0.94 (s, 9H). **^{13}C NMR** (100 MHz, $CDCl_3$) δ 162.84, 152.72, 137.85, 134.53, 129.74, 127.07, 121.47, 120.43, 109.10, 44.09, 32.20, 29.17, 27.07, 23.53, 23.46. **GC-MS**: 256 (100), 200 (42), 144 (19), 115 (24). **Anal. Calc.** for ($C_{17}H_{20}O_2$: 256.35): C, 79.65; H, 7.86; found: C, 79.83; H, 7.99.

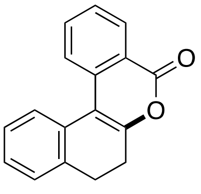

7c. White solid. *c*Hex:EtOAc: 20:1. Yield = 82% (20.3 mg). M.p. = 158 – 159 °C. **^{1}H NMR** (400 MHz, $CDCl_3$) δ 8.38 – 8.36 (m, 1H), 8.10 (d, *J* = 8.2 Hz, 1H), 7.77 – 7.71 (m, 2H), 7.53 – 7.49 (m, 1H), 7.32 – 7.29 (m, 2H), 7.24 - 7.20 (m, 1H), 2.93 (t, *J* = 7.4 Hz, 2H), 2.76 (t, *J* = 7.6 Hz, 2H). **^{13}C NMR** (100 MHz, $CDCl_3$) δ 161.96, 155.69, 135.76, 135.28, 134.45, 130.96, 130.39, 128.10, 127.39, 126.78, 126.43, 125.63, 123.67, 121.59, 110.98, 28.37, 27.34. **GC-MS**: 248 (100), 219 (48), 189 (38), 165 (22). **Anal. Calc.** for ($C_{17}H_{12}O_2$: 248.08): C, 82.24; H, 4.87; found: C, 82.42; H, 5.02.

Diagnostic signal of minor the isomer: ^{1}**H NMR** (401 MHz, $CDCl_3$) δ 8.77 (d, *J* = 8.7 Hz, 1H), 8.65 (d, *J* = 8.2 Hz, 1H), 8.49 (dd, *J* = 7.9 Hz, *J* = 1.1 Hz, 1H), 7.95 – 7.93 (m, 1H), 7.90 – 7.96 (m, 1H), 7.68 – 7.66 (m,1H), 7.64 - 7.60 (m, 1H), 7.56 – 7.54 (m, 1H).

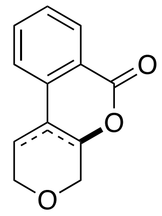

7d. Viscous oil. *c*Hex:EtOAc: 20:1. Yield = 40% (8.1 mg), as a 57:43 regioisomeric mixture. ^{1}**H NMR** (400 MHz, $CDCl_3$) δ 8.30 (dd, *J* = 7.9 Hz, *J* = 1.0 Hz, 1H), 7.75 (m, 1H), 7.49 (m, 1H), 7.42 (d, *J* = 8.0 Hz, 1H), 4.43 (t, *J* = 2.0 Hz, 2H), 4.01 (t, *J* = 5.5 Hz, 2H), 2.72 – 2.68 (m, 2H); ^{13}**C NMR** (100 MHz, $CDCl_3$) δ 149.75, 134.88, 134.24, 130.12, 129.37, 127.81, 125.68, 121.57, 121.10, 100.21, 64.69, 64.08, 63.12, 35.55, 22.61; **GC-MS**: 155 (100), 202 (88), 145 (42), 102 (34); **Anal. Calc.** for ($C_{12}H_{10}O_3$: 202.06): C, 71.28; H, 4.98; found: C, 71.43; H, 5.24. Diagnostic signal of the minor isomer: ^{1}**H NMR** (401 MHz, $CDCl_3$) δ 7.87 (dd, *J* = 7.6 Hz, *J* = 1.0 Hz, 1H), 7.66 (td, *J* = 7.5 Hz, *J* = 1.1 Hz, 1H), 7.53 (m, 1H), 7.38 (m, 1H), 6.75 (d, *J* = 6.1 Hz, 1H), 4.68 (dd, *J* = 6.1 Hz, *J* = 2.1 Hz, 1H), 4.35 - 4.31 (m, 1H), 4,22 - 4.29 (m,1H), 2.40 - 2.32 (m, 1H), 2.06 – 2.00 (m, 1H); **GC-MS:** 157 (100), 202 (24), 129 (81), 146 (58).

Functionalization of 5ab

Epoxidation reaction

The epoxidation reaction of (+/-)-**5ab** was performed using the following procedure.[9]

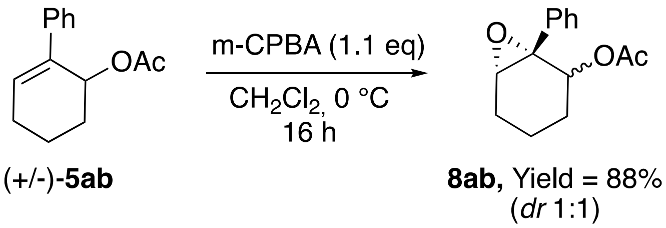

A dry two-necked flask was charged with DCM (1 mL) and (+/-)-**5ab** (94 μmol, 21.8 mg). The solution was cooled to 0 °C and *m*CPBA (17.2 mg, 0.1 mmol) was added. The reaction mixture was stirred 16 h at rt or until completion (TLC). Then H_2O was added and the resulting mixture was extracted with DCM (3 x 5 mL), then the organic phase was dried over Na_2SO_4 and concentrated under vacuum. The residue was purified with gel chromatography (cHex/AcOEt = 40/1) to give **8ab** as a viscous oil (19.2 mg, 88%). The two diastereomers were effectively separated by flash chromatography.

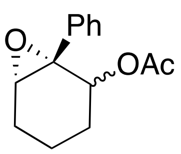

8ab: **^{1}H NMR** (400 MHz,$CDCl_3$) δ 7.39-7.37 (m, 1H), 7.36- 7.35 (m, 1H), 7.31 – 7.29 (m, 1H), 7.29 – 7.25 (m, 2H), 5.41 (t, *J* = 4.1 Hz, 1H), 3.36 - 3.35 (m, 1H), 2.07 - 2.03 (m, 2H), 1.93 - 1.86 (m, 1H), 1.81 (s, 3H), 1.64 - 1.55 (m, 2H), 1.49 – 1.41 (m, 1H). **^{13}C NMR** (100 MHz, $CDCl_3$) δ 169.66, 138.18, 127.90, 127.87, 127.49, 70.18, 61.49, 59.48, 25.70, 23.33, 20.85, 14.67. **GC-MS**: 133 (100), 105 (96), 77 (51). **Anal. Calc.** for ($C_{14}H_{16}O_3$: 232.28): C, 72.39; H, 6.94; found: C, 72.58; H, 7.21.

8ab': **^{1}H NMR** (400 MHz,$CDCl_3$) δ 7.36-7.35 (m, 1H), 7.33- 7.30 (m, 3H), 7.30 – 7.25 (m, 1H), 5.74 (dd, *J* = 9.4 Hz, *J* = 5.5 Hz, 1H), 3.13 (d, *J* = 4.3 Hz, 1H), 2.04 - 1.92 (m, 2H), 1.90 (s, 3H), 1.88 - 1.81 (m, 1H), 1.70 - 1.61 (m, 2H), 1.51 – 1.43 (m, 1H). **^{13}C NMR** (100 MHz, $CDCl_3$) δ 138.21, 128.33, 127.59, 125.48, 71.49, 64.55, 61.36, 25.49, 23.35, 20.90, 19.77. **GC-MS**: 133 (100), 105 (89), 77 (44). **Anal. Calc.** for ($C_{14}H_{16}O_3$: 232.28): C, 72.39; H, 6.94; found: C, 72.57; H, 7.24.

Hydrolysis

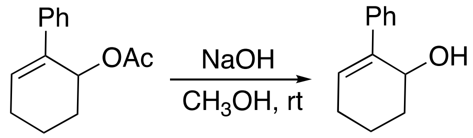

(+/-)-**5ab** (+/-)-**9ab**
89%

A flask was charged with MeOH (1 mL) and (+/-)-**5ab** (0.12 mmol, 26 mg). Then a solution of NaOH (3 mL, 1 M) was added. The reaction was checked over time until the SM disappeared. Then H_2O was added, and the resulting mixture was extracted with AcOEt (3 x 5 mL), then the organic phase was dried over Na_2SO_4 and concentrated under vacuum. The residue was purified with gel chromatography (cHex/AcOEt = 15/1) to give (+/-)-**9ab** as a viscous oil (18.5 mg, 89%).

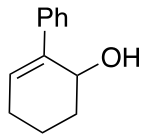

(+/-)-**9ab.** ^{1}**H NMR** (400 MHz, $CDCl_3$) δ 7.47-7.44 (m, 2H), 7.34- 7.30 (m, 2H), 7.26 – 7.22 (m, 1H), 6.15 (dd, *J* = 4.6 Hz, *J* = 3.5 Hz , 1H), 4.71 (m, 1H), 2.30 - 2.22 (m, 1H), 2.20 - 2.17 (m, 1H), 1.99 – 1.92 (m, 1H), 1.89 - 1.82 (m, 1H), 1.81 – 1.73 (m, 1H), 1.71 - 1.63 (m, 1H), 1.61 (bs, 1H,). ^{13}**C NMR** (100 MHz, $CDCl_3$) δ 140.14, 139.07, 128.65, 128.49, 127.06, 125.96, 65.44, 31.53, 26.03, 17.32. **GC-MS**: 174 (100), 115 (99), 91 (92), 156 (52). **Anal. Calc.** for ($C_{12}H_{14}O$: 174.10): C, 82.72; H, 8.10; found: C, 82.97; H, 8.23.

Monitoring of the reaction conversion.

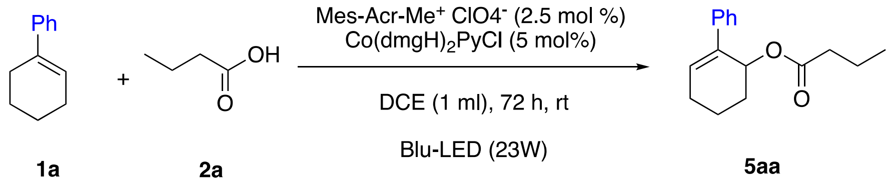

Two different dry vials equipped with a stirring bar were charged with: Mes-Acr-Me$^+$ClO$_4^-$ (1.0 mg, 2.5 mol%), [Co(dmgH)$_2$(Py)(Cl)] (2.0 mg, 5 mol%), dry DCE (1 ml), **1a** (0.1 mmol) and **2a** (1.0 mmol). The solutions were degassed with N_2 then stirred under 23 W blue LED irradiation. Samples injected into HPLC were prepared by taking 50 μL from the vial reaction and subsequently diluted. In the case of the first graph (**Scheme S1**) showed below, one of the two catalysis was monitored by taking samples during 72 h. In the second graph (**Scheme S2**) the second catalysis was always monitored over 72 h but exposing the reaction alternatively to the dark and to irradiation.

Scheme S1. Experiment with continuous irradiation.

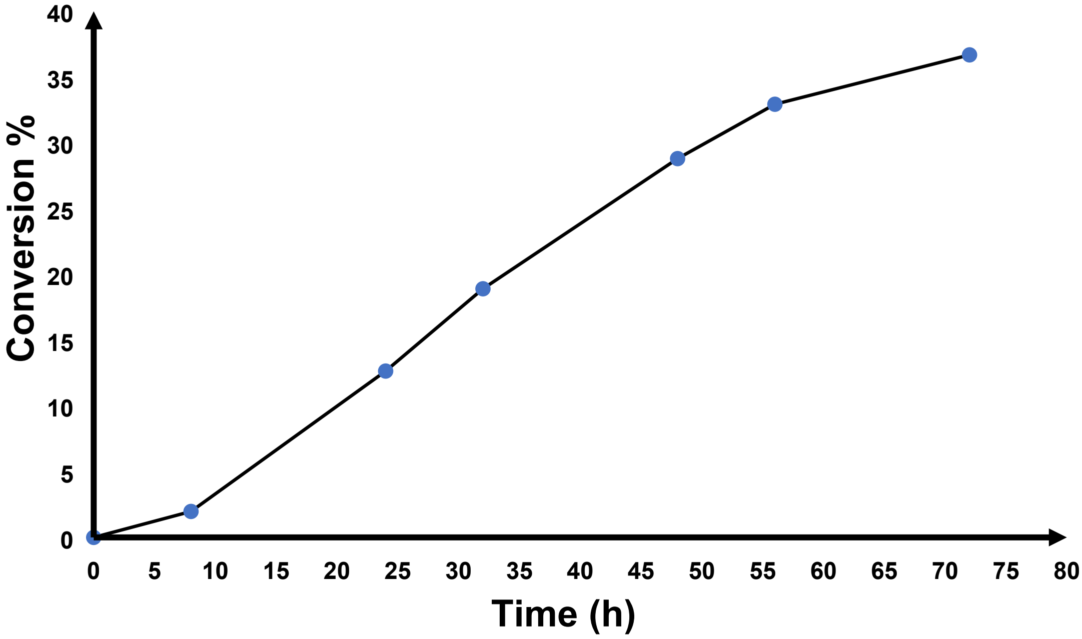

Scheme S2. On-off experiment

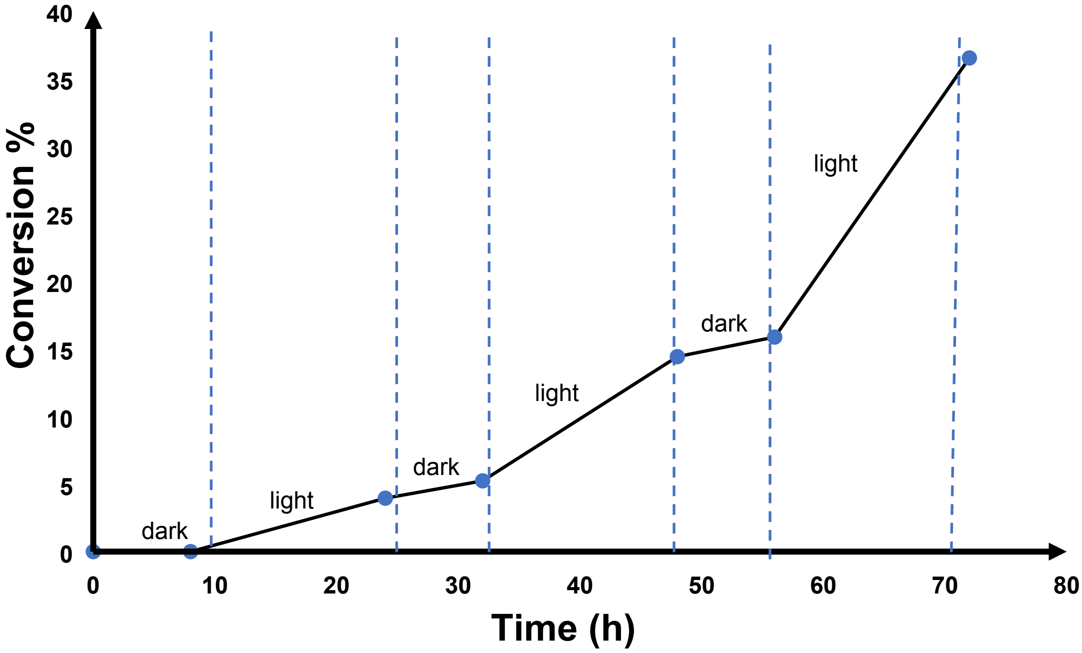

Kinetic isotope effect

Synthesis of d^3-1a.[10]

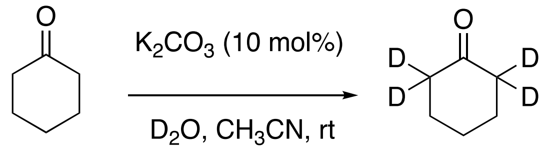

A dry two-necked flask was charged with D_2O (9 ml), CH_3CN (0.3 ml), cyclohexanone (309 μl, 3.0 mmol) and potassium carbonate (41 mg, 10 mol%). The reaction mixture was stirred at room temperature and controlled by **^{1}H NMR** analysis until an almost total conversion of cyclohexanone into the corresponding d^4 analogous was observed. Then the mixture was extracted with DCM (3 x 5 mL) and the organic phase was dried over Na_2SO_4. Upon removal of the solvent under reduced pressure, a reaction crude was obtained and used directly in the next step.

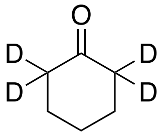

d^4-cyclohexanone: **^{1}H NMR** (401 MHz, $CDCl_3$) δ 1.84 - 1.81 (m, 4H), 1.72 – 1.67 (m, 2H).

In the second step in a dry three-necked flask equipped with condenser the d^4-cyclohexanone (ca. 3 mmol) was dissolved in 2 ml of THF was added dropwise at 0 °C to a solution of freshly prepared PhMgBr (3.6 mmol ca. 0.5 M). The reaction was stirred at rt for 7 h when the TLC showed the reaction completed. Then, a sat. NH_4Cl solution was added (5 mL) at 0 °C and the resulting mixture was extracted with AcOEt (3 x 5 mL). The organic phase was dried over Na_2SO_4 and concentrated under vacuum. The reaction crude was used directly in the next step.

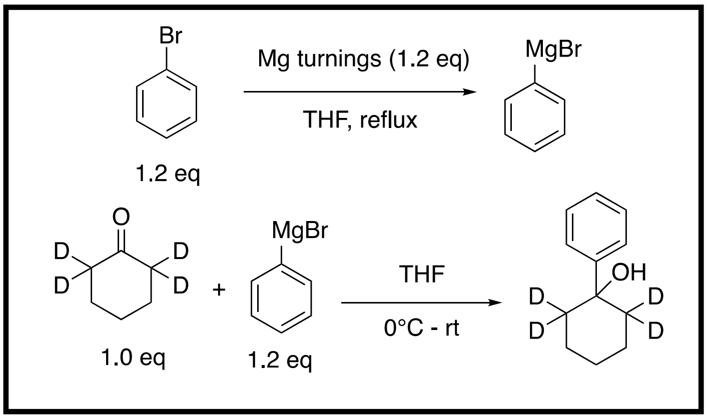

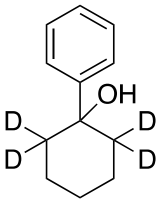

d^4-1-Phenylcyclohexan-1-ol: **^{1}H NMR** (401 MHz, $CDCl_3$) δ 7.51 – 7.41 (m, 2H), 7.37 – 7.33 (m, 2H), 7.26 – 7.24 (m, 1H), 1.77 - 1.74 (m, 2H), 1.77 - 1.74 (m, 2H), 1.73

– 1.70 (m, 2H), 1.64 – 1.62 (m, 2H). **GC-MS**: 134 (100), 105 (59), 77 (39), 180 (37), 56 (29)

In the final step a dry two-necked flask a solution of deuterated hydrochloric acid (DCl) 7.6 N (12.0 mmol, 1.6 ml) was added drop wise at 0 °C to the reaction crude of the previous step dissolved in 2 ml of THF. The reaction was stirred at rt until the SM disappeared. Then, the resulting mixture was extracted with Et_2O (3 x 5 mL). The organic phase was dried over Na_2SO_4 and concentrated under vacuum. The residue was purified with gel chromatography (*n*Hex = 100%) to give d^3-**1a** as a colorless oil (106 mg, 22%, unoptimized).

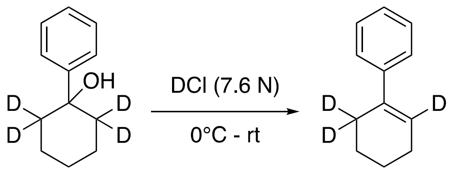

d^3-**1a**

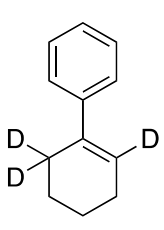

d^3-**1a**: **^{1}H NMR** (400 MHz, $CDCl_3$) δ 7.38-7.35 (m, 2H), 7.31- 7.27 (m, 2H), 7.21 – 7.17 (m, 1H), 2.19 (t, *J* = 6.1 Hz, 2H), 1.77 – 1.74 (m, 2H), 1.68 – 1.62 (m, 2H). **^{13}C NMR** (100 MHz, $CDCl_3$) δ 142.61, 136.64, 128.13, 126.43, 124.86, 124.45 (C-D, t, *J* = 18.0 Hz,1C), 26.54 (C-D_2, quintet, *J* = 20.5 Hz, 1C), 25.71, 22.83, 22.07. **GC-MS**: 181 (100), 131 (80), 145 (46), 116 (39). **Anal. Calc.** for ($C_{12}H_{11}D_3$: 161.13): C, 89.38; H, 10.62; found: C, 89.53; H, 10.83

KIE investigation from intermolecular competition

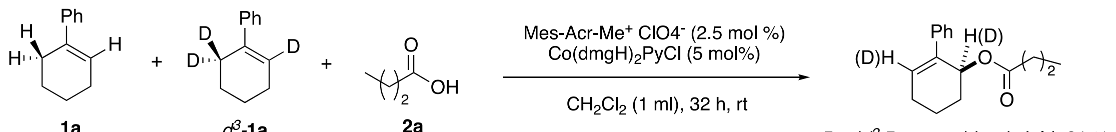

5aa/d^2-5aa, combined yield, 21 %
(P_H/P_D) = 1.0

For this type of experiment a 5 mL dry vial equipped with a stirring bar was charged with: Mes-Acr-Me$^+$ClO$_4^-$ (1.0 mg, 2.5 mol%), [Co(dmgH)$_2$(Py)(Cl)] (2.0 mg, 5 mol%), dry DCM (1 mL), **1a** (50 μmol), d^3-**1a** (50 μmol) and **2a** (1.0 mmol). The solution was degassed with N_2 then stirred under 23 W blue LED irradiation (465 nm) for 32 h. Then, the solvent was removed under vacuum and the residue purified via flash chromatography (*c*Hex/AcOEt = 100/1, Yield: 21%). The KIE value was given after **^{1}H NMR** analysis.

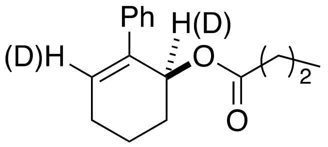

Signal of d^2-**5aa** + **5aa**: **^{1}H NMR** (400 MHz, $CDCl_3$) δ 7.32-7.25 (m, 8H), 7.22 - 7.18 (m, 2H,), 2.34 – 2.30 (m, 1H), 2.30 – 2.27 (m, 1H), 2.22 – 2.18 (m, 2H), 2.16 - 2.12 (m, 4H), 1.99 – 1.94 (m, 2H), 1.89 – 1.84 (m, 2H), 1.74 – 1.68 (m, 4H), 1.54 – 1.45 (sext, J = 7.2 Hz , 4H), 0.79 (t, J = 7.5 Hz, 6H).

Diagnostic signals of **5aa:** 6.31 (m, 1H), 5.94 (m, 1H).

Synthesis of the photocatalyst 4c

The photocatalyst **4c** was synthesized following the procedure described in the literature with slight modification.[11]

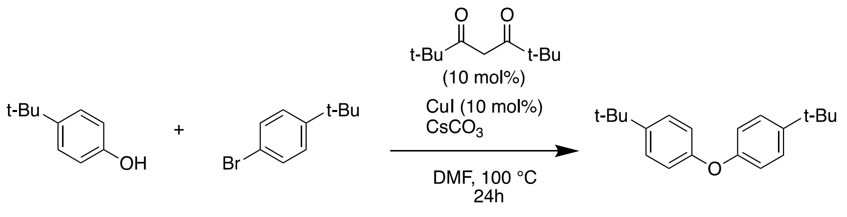

In the first step a Schlenk tube was charged with 1-bromo-4-(tert-butyl)benzene (1.06 g, 5.0 mmol), 4-(tert-butyl)phenol (1.13 mg, 7.5 mmol), Cs_2CO_3 (3.25 g, 10 mmol), CuI (95 mg, 10 mol%), 2,2,6,6-tetramethylheptane-3,5,-dione (92 mg, 10 mol%) and anhydrous DMF (1.5 ml). The reaction was stirred at 100 °C for 24 h, then cooled at room temperature and the DMF removed under vacuum. Et_2O was added and the reaction crude was filtered through celite until the washing layers became colorless. The filtrate was washed with water (2 x 10 ml) followed by brine (1 x 10 ml). The organic phase was dried over Na_2SO_4 and concentrated under vacuum. The residue was purified with gel chromatography (*c*Hex/AcOEt = 98/2) to give 4,4'-oxybis(tert-butylbenzene) as a viscous oil (630 mg, 45%).

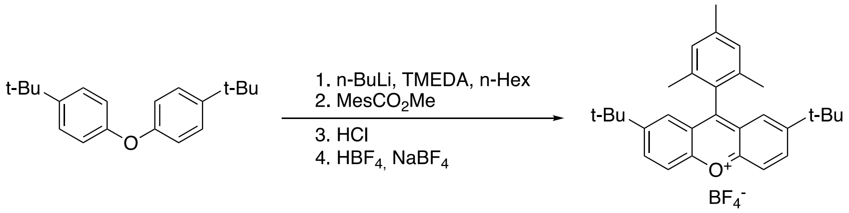

In the second step a flame-dried 3-necked 50 mL round bottom flask was charged under nitrogen with 4,4'-oxybis(tert-butylbenzene) (630 mg, 2.23 mmol), TMEDA (680 μl, 4.57 mmol) and anhydrous *n*Hex (3 mL). The resulting solution was cooled to -78°C and n-butyllithium (2.5 M in *n*Hex, 1.83 ml, 4.57 mmol) was added dropwise. The reaction mixture was allowed to warm to room temperature and was stirred at room temperature for 4 h.

The reaction was cooled to - 78 °C and a solution of methyl 2,4,6- trimethylbenzoate (401 mg, 2.25 mmol) in anhydrous n-hexane (3 ml) was added dropwise. After the addition, the reaction was allowed to slowly warm to room temperature and stirred overnight. The reaction was quenched with water and the biphasic mixture was stirred vigorously for 30 min. The mixture was diluted with Et_2O and the layers were separated. The organic layer was washed with water and brine. The organic layer was transferred to a 50 mL round bottom flask equipped with a stir bar. To the vigorously stirred solution was added conc. HCl (1 mL) resulting in a bright yellow precipitate that slowly turned brown over the course of addition. The brown suspension was stirred vigorously for 30 min then diluted with water. The layers were separated, and the organic

layer was extracted with water until the washings become colorless. To the combined aqueous layers was added solid $NaBF_4$ (736 mg, 6.69 mmol) resulting in a bright yellow precipitate. The resulting suspension was extracted with dichloromethane until the washings become colorless. To the combined organic layers was added HBF_4 (48 wt% in water, 0.29 mL, 2.23 mmol). Water was added, the phases were separated, and the organic layer was washed once with water and then once with aq. $NaBF_4$ (1 M, 7.85 ml). The organic layer was dried over solid NaBF4, filtered, and concentrated to dryness. The residue was purified by trituration with n-hexane and filtered. The solid was rinsed with n-pentane and dried in vacuo to give 2,7-di-tert-butyl-9-mesitylxanthylium tetrafluoroborate (264 mg, 0.53 mmol, 24% yield, unoptimized) as a yellow-orange solid.

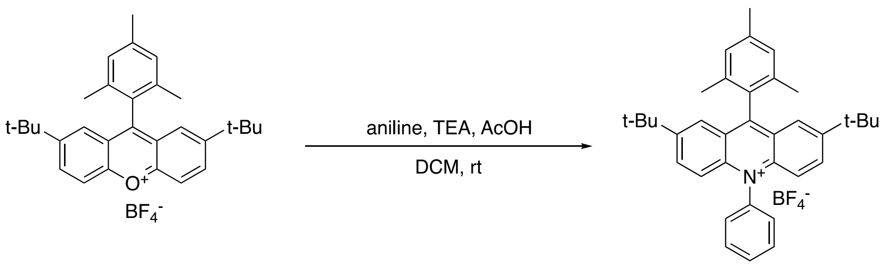

4c

To an oven-dried Schlenk tube under nitrogene were added 2,7-di-tert-butyl-9-mesitylxanthylium tetrafluoroborate (263 mg, 0.53 mmol) and dry, degassed dichloromethane (1.5 mL). To the resulting solution were added acetic acid (0.091 mL, 95 mg, 1.59 mmol) followed by NEt_3 (0.11 ml, 80mg, 0.795 mmol). Aniline (0.059 mL, 60 mg, 0.64 mmol) was then added dropwise. The flask was covered with aluminum foil and stirred at room temperature for 12 h. The reaction was transferred to a separatory funnel and washed with water followed by sat. aq. $NaHCO_3$. To the organic layer was added HBF_4 (48 wt% in water, 0.096 ml, 0.53 mmol). Water was added, the phases were separated and the organic layer was washed once with water and then once with aq. $NaBF_4$ (1M, 1.85 ml). The organic layer was dried over solid $NaBF_4$, filtered, and concentrated to dryness. The residue was purified by trituration with 1:2 Et_2O/hexanes and filtered. The solid was rinsed with n-pentane and dried in vacuo to give 2,7-di-tert-butyl-9-mesityl-10-phenylacridin-10-ium tetrafluoroborate **4c** (237 mg, 0.41 mmol, 78% yield) as a bright yellow solid.

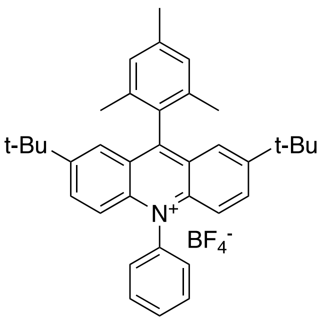

4c

4c. Bright yellow solid. M.p. = decomposition; **^{1}H NMR** (400 MHz, $CDCl_3$) δ 8.16 (dd, *J* = 9.5 Hz, *J* = 2.1 Hz, 2H), 7.92 – 7.89 (m, 2H), 7.87 – 7.83 (m, 1H), 7.70 (d, *J* = 1.9 Hz, 2H), 7.68 – 7.66 (m, 2H), 7.54 (d, *J* = 9.4 Hz, 2H), 7.17 (s, 2H), 2.50 (s, 3H), 1.82 (s, 6H), 1.28 (s, 18H). **^{13}C NMR** (100 MHz, $CDCl_3$) δ 162.70, 162.65, 151.94, 151.90, 140.24, 140.20, 140.06, 140.03, 137.90, 137.86, 136.73, 136.70, 135.89, 135.79, 131.92, 131.89, 131.62, 131.60, 129.18, 129.15, 129.00, 128.98, 127.87, 127.84, 125.84, 125.83, 122.69, 119.87, 119.86, 35.25, 30.57, 21.36, 20.15. All aromatic

carbons except one (122.69 ppm) appear splitted into two slightly broad signals. **^{19}F NMR** (377 MHz, $CDCl_3$) δ -154.31 (s, 1 F) -154.36 (s, 4 F). Two signals in 4:1 intensity ratio are observed due to the natural abundance of ^{10}B (20%) and ^{11}B (80%) **HRMS:** 486.31608 [M-BF_4]

Stern-Volmer experiments

Quenching of the excited state of **4a** and **4c** was investigated by measuring the excited state lifetime (by TCSPC) in the presence of increasing amount of quencher Q (**1a**). The data were analyzed according to the Stern-Volmer equation in order to determine the quenching constant k_q:

$$\frac{\tau_0}{\tau} = 1 + \tau_0 k_q [Q]$$

In order to elucidate the reaction mechanism and to rule out the possible reaction of the excited **4c** with butyric acid the lifetime of **4c** in the presence of butyric acid up to 80 mM concentration was measured. No effect of the carboxylic acid on **4c** excited state lifetime was detected.

Figure S1: Cyclic Voltammetry curves

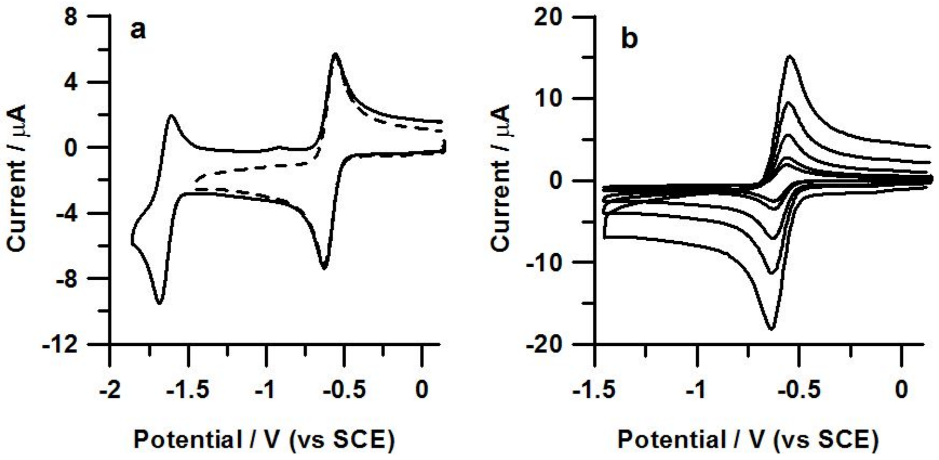

Experimental details: 1 mM solution of **4c** in a 0.07 M TBAH/CH_3CN electrolyte working electrode, Glassy carbon disk, 1mm diameter at a) $1Vs^{-1}$ and b) 0.1, 0.2, 1, 2 and 5 Vs^{-1}

Table S2. Half-wave ($E_{½}$) redox potentials (*vs.* SCE)

Species	$E_{½}$ / V (ΔE/mV) [a]	
**4c**	**-0.59**	**-1.65**
	**(69)**	**(75)**

[a] Reduction potentials measured in CH_3CN solution.

Figure S2: Absorption and fluorescence spectra in CH_3CN

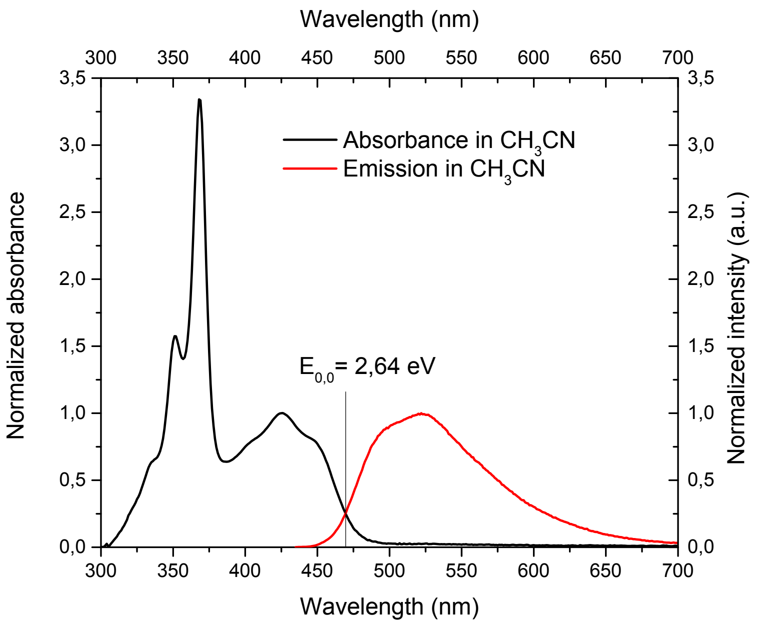

Experimental details: λ_{exc} = 420 nm, **4c** 5x10^{-5} M in CH_3CN at room temperature. Fluorescence quantum yields and excited state lifetimes were 29% (τ = 10.9 ns) and 38% (τ = 13.7 ns) in aerated and de-aerated conditions respectively.

Figure S3: Absorption and fluorescence spectra in CH_2Cl

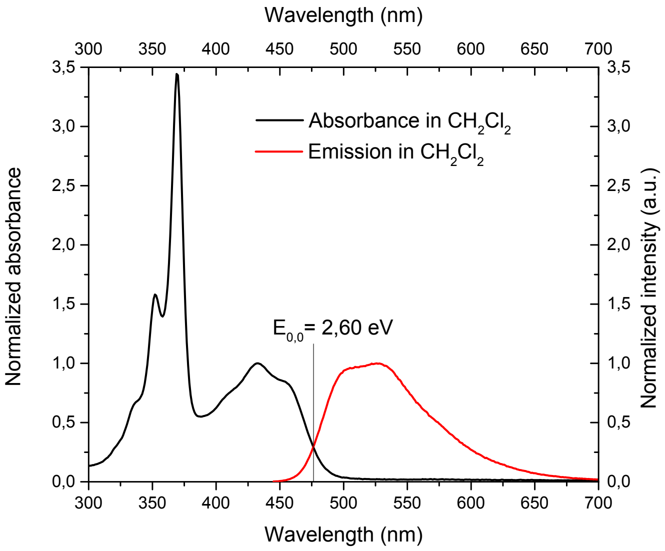

Experimental details: (λ_{exc} = 420 nm), **4c** 5 x 10^{-5} M in CH_2Cl_2 at room temperature. Fluorescence quantum yields and excited state lifetimes were 84% (τ = 17.0 ns) and 90% (τ = 17.6 ns) in aerated and de-aerated conditions respectively.

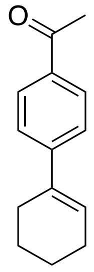

1n

^{1}H NMR (400 MHz, $CDCl_3$)

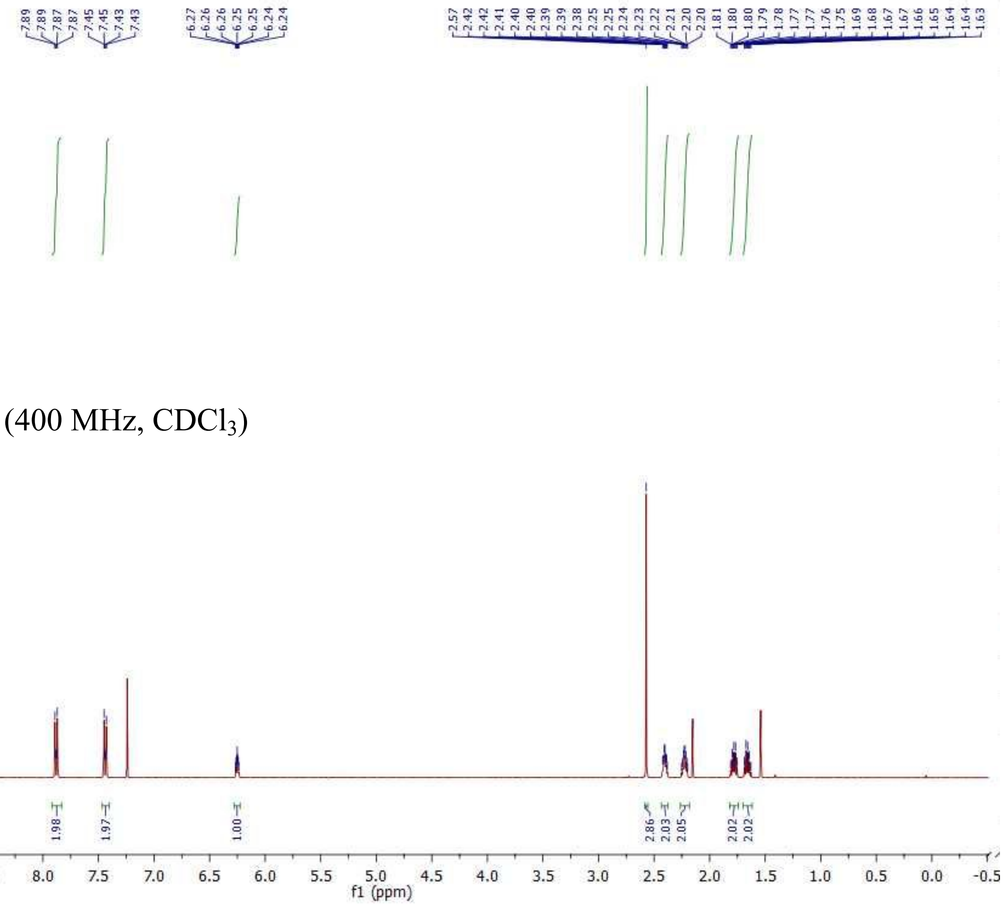

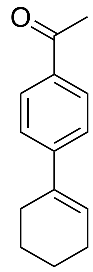

1n

^{13}C NMR (100 MHz, $CDCl_3$)

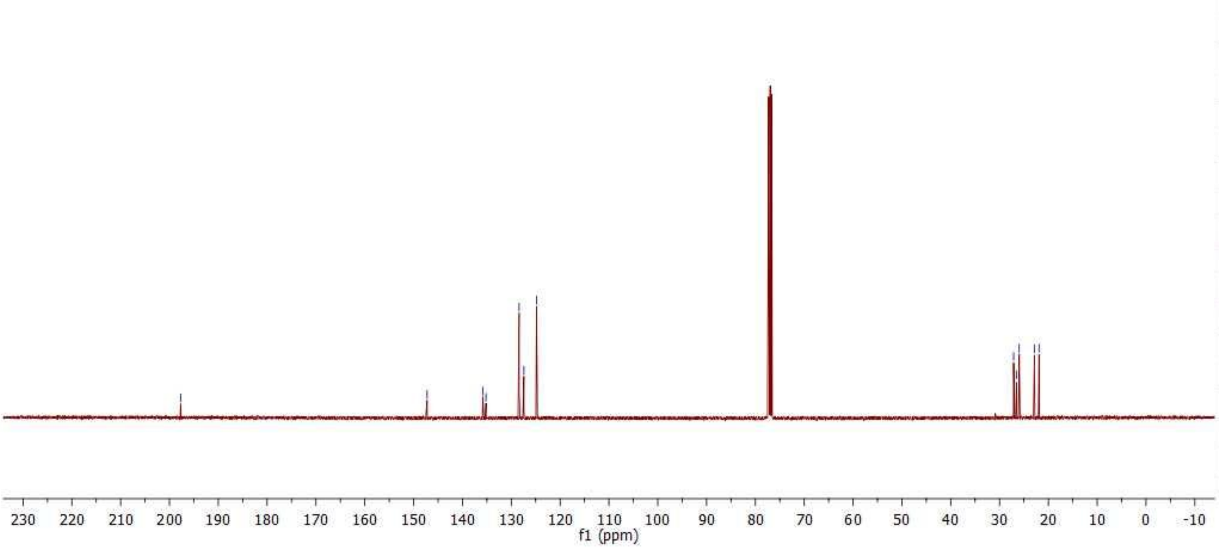

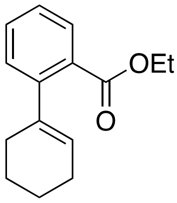

^{1}H NMR (400 MHz, $CDCl_3$)

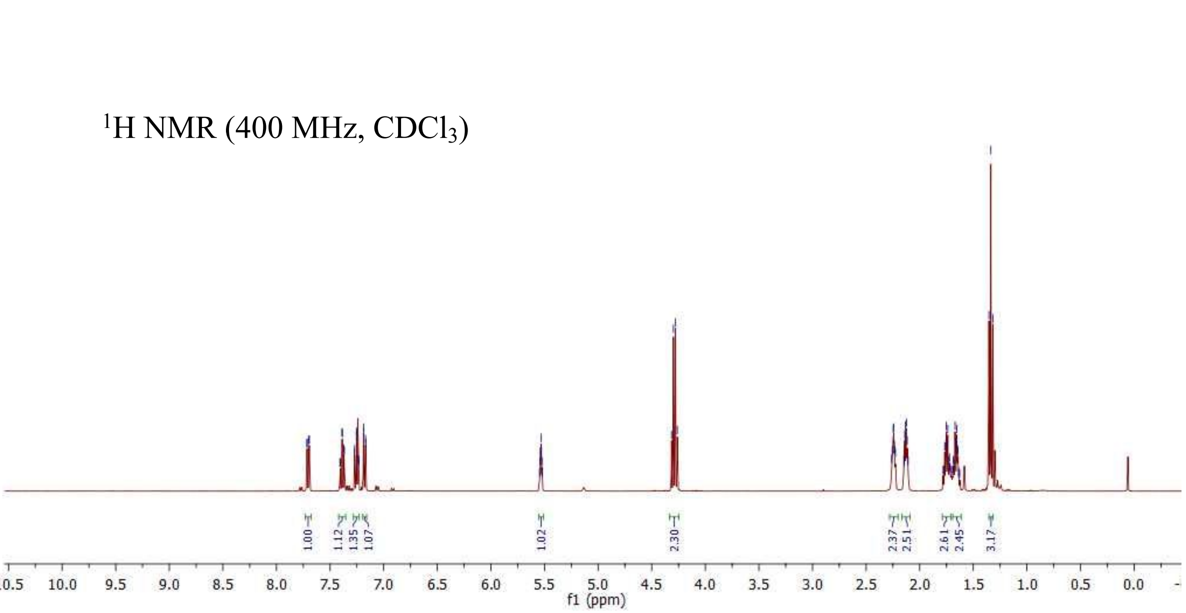

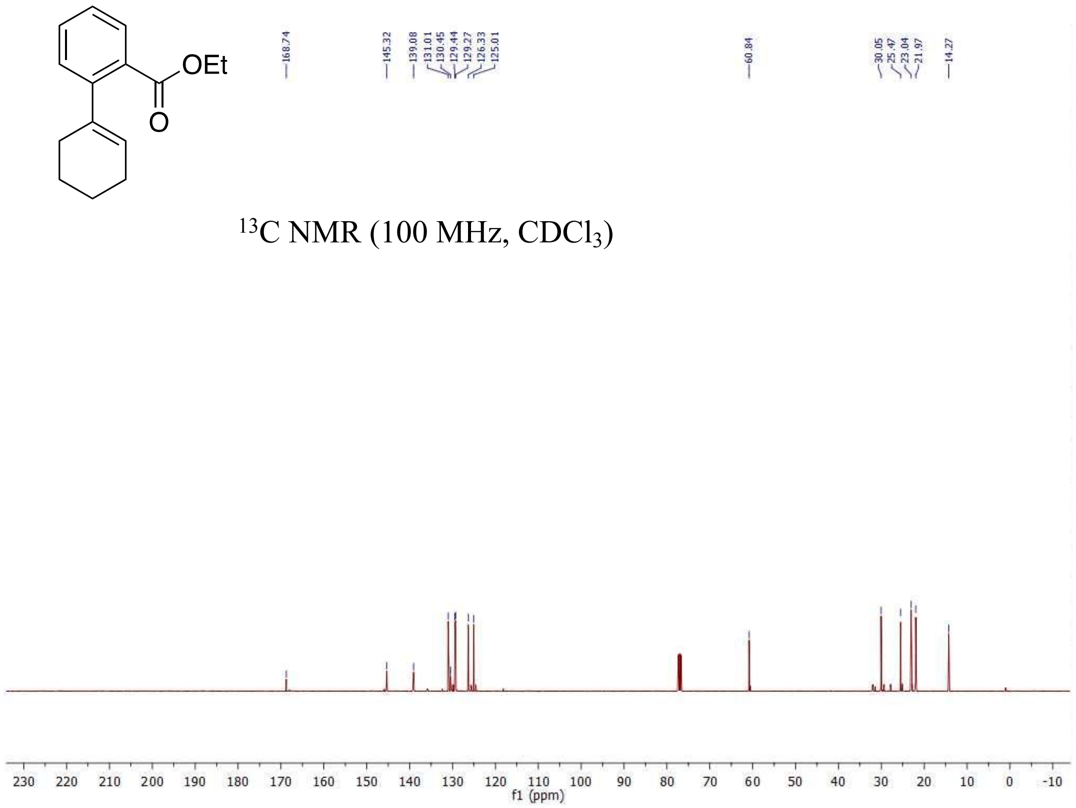

^{13}C NMR (100 MHz, $CDCl_3$)

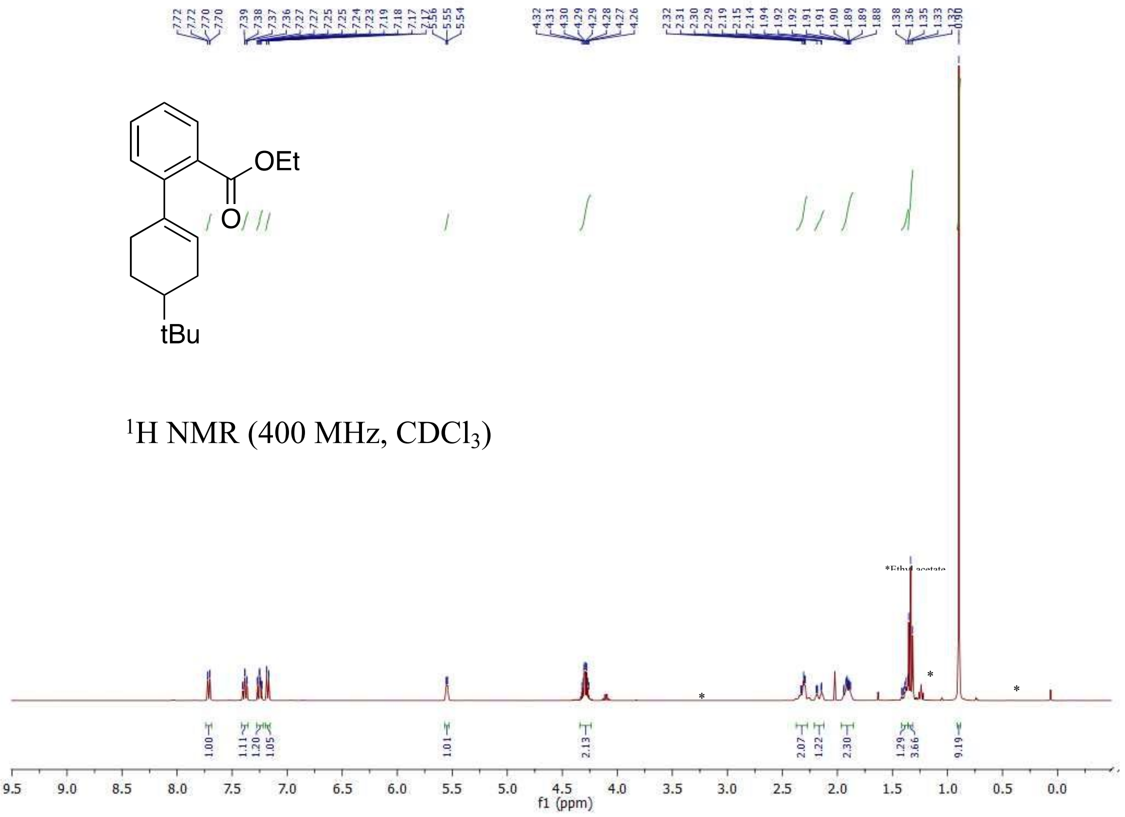

^{1}H NMR (400 MHz, $CDCl_3$)

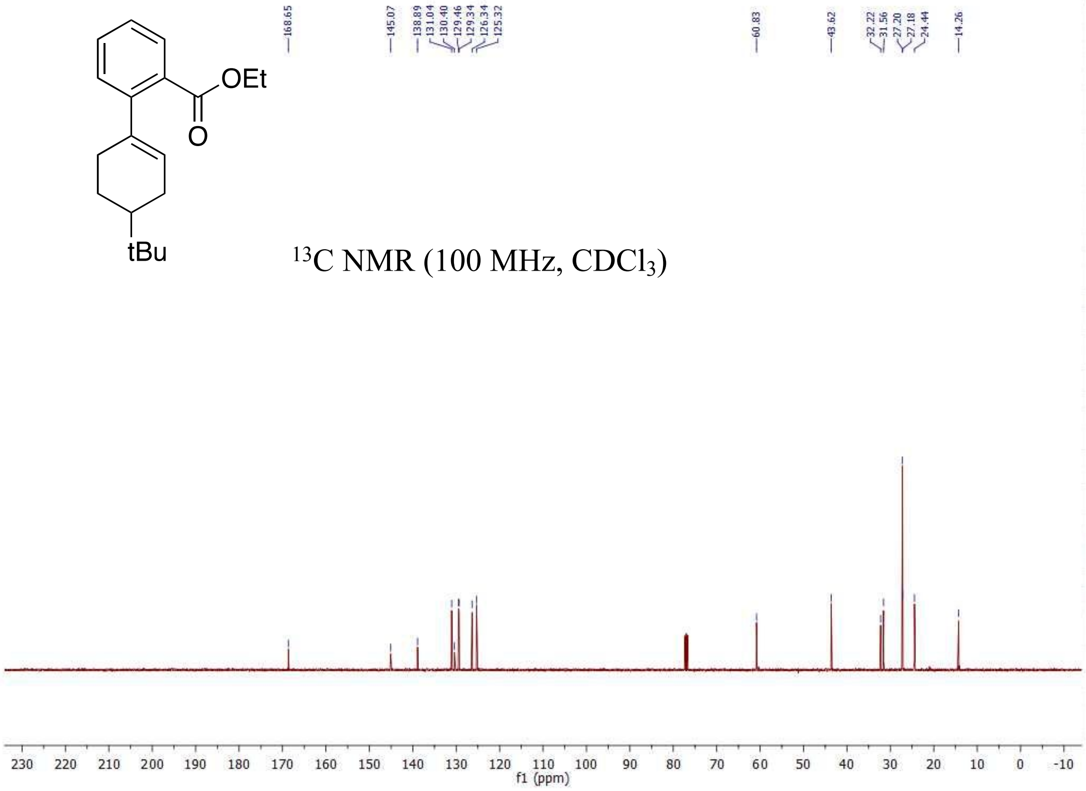

^{13}C NMR (100 MHz, $CDCl_3$)

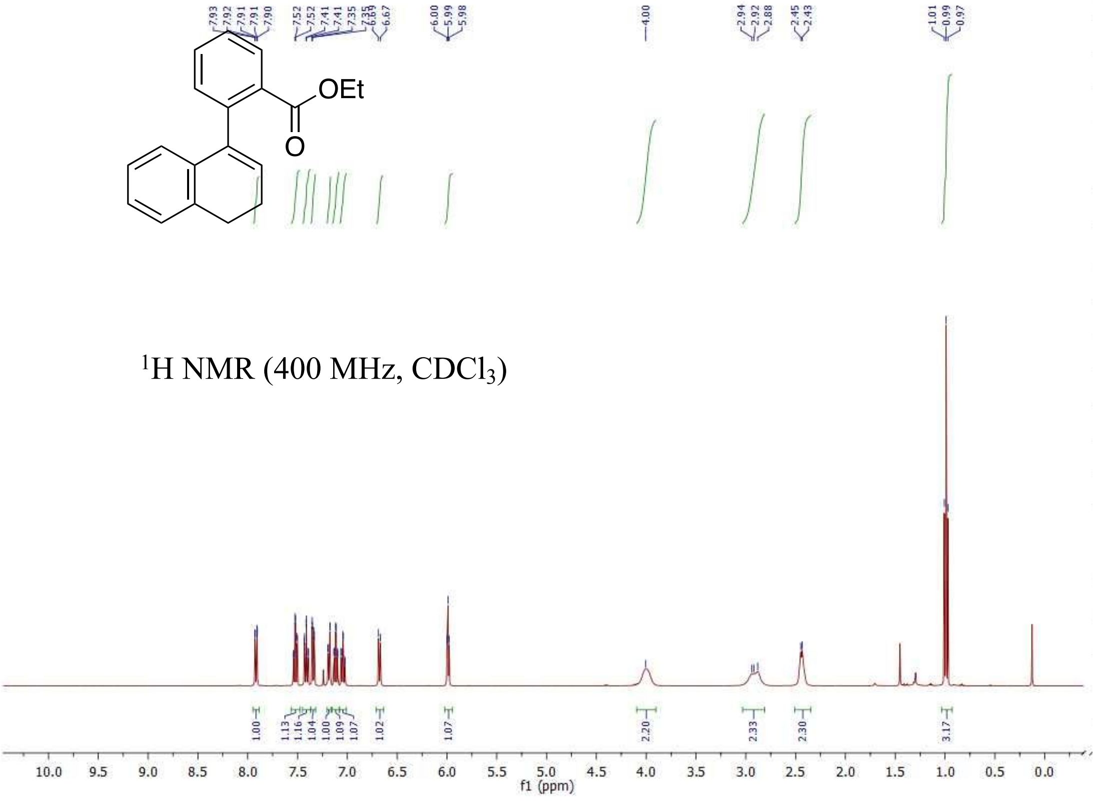

^{1}H NMR (400 MHz, $CDCl_3$)

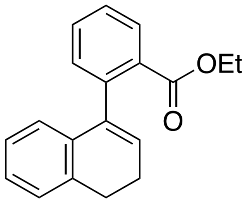

^{13}C NMR (100 MHz, $CDCl_3$)

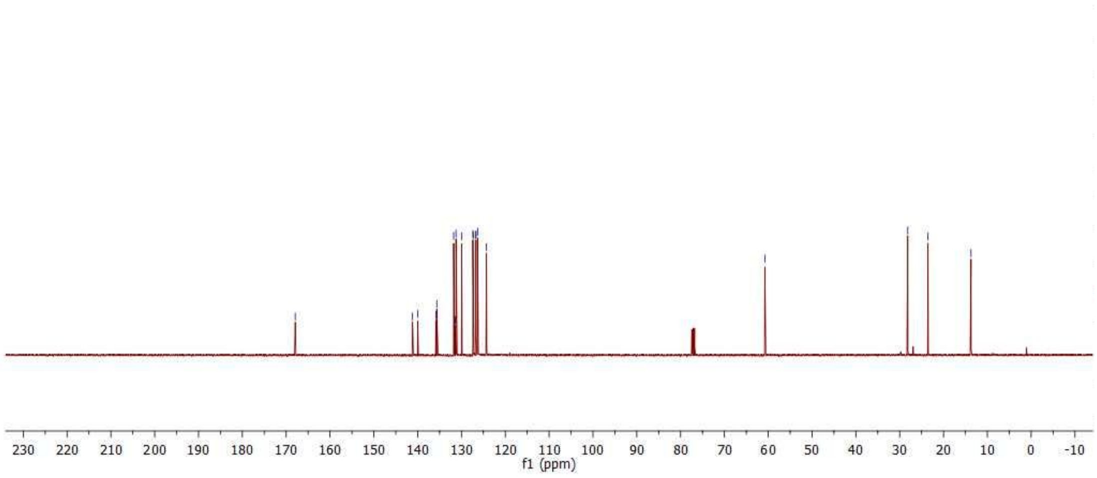

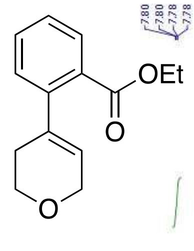

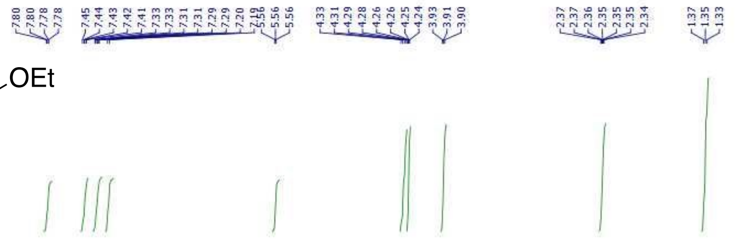

^{1}H NMR (400 MHz, $CDCl_3$)

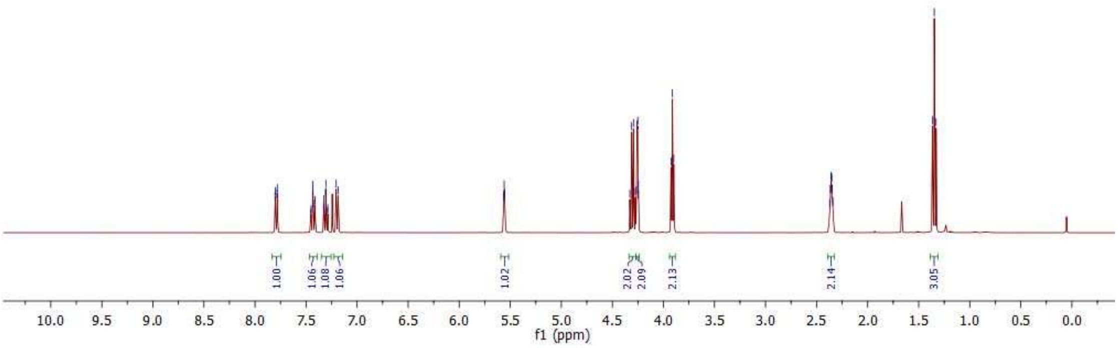

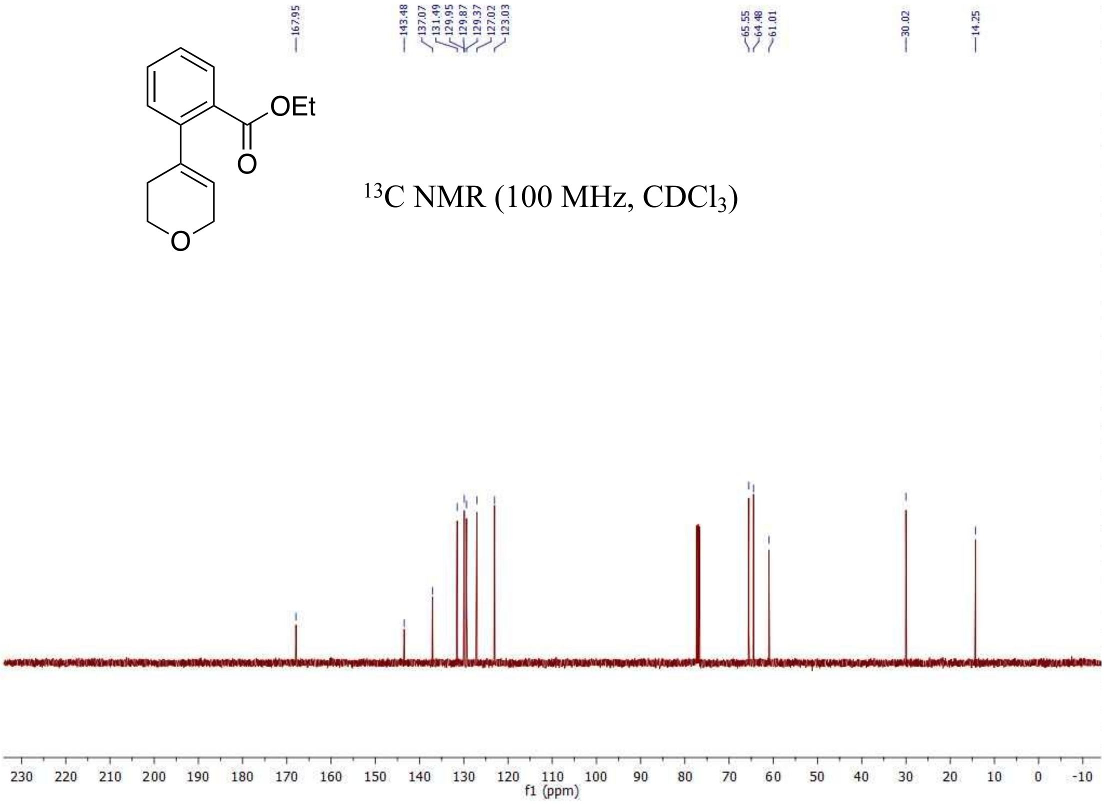

^{13}C NMR (100 MHz, $CDCl_3$)

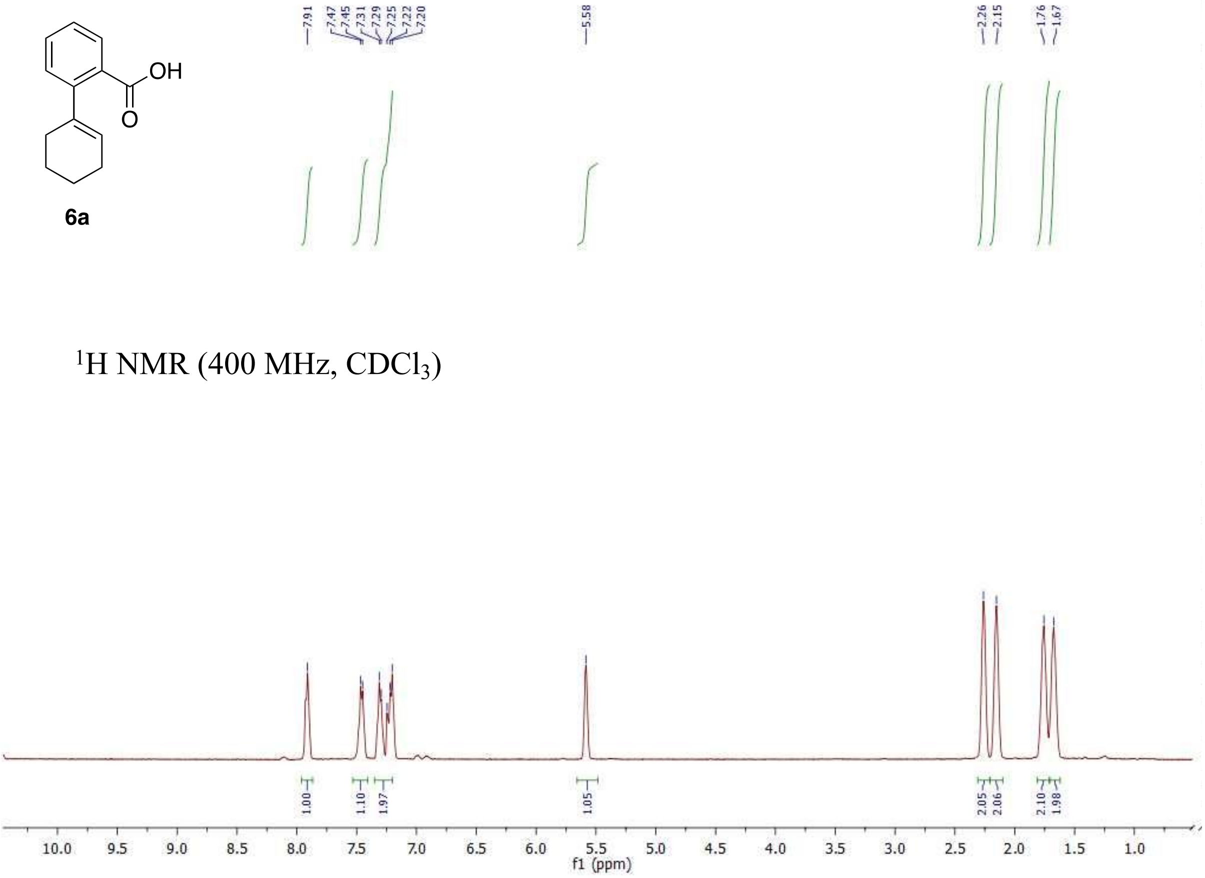

^{1}H NMR (400 MHz, $CDCl_3$)

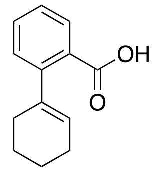

6a

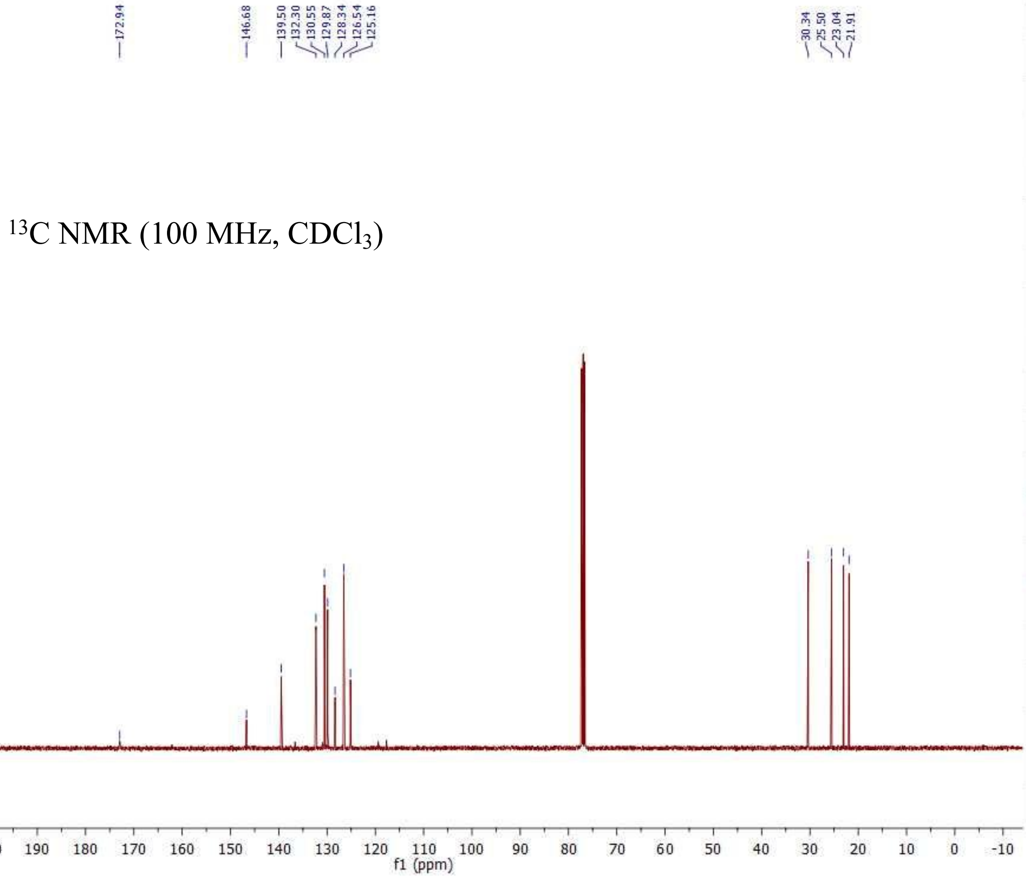

^{13}C NMR (100 MHz, $CDCl_3$)

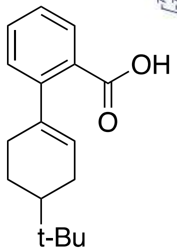

6b

^{1}H NMR (400 MHz, $CDCl_3$)

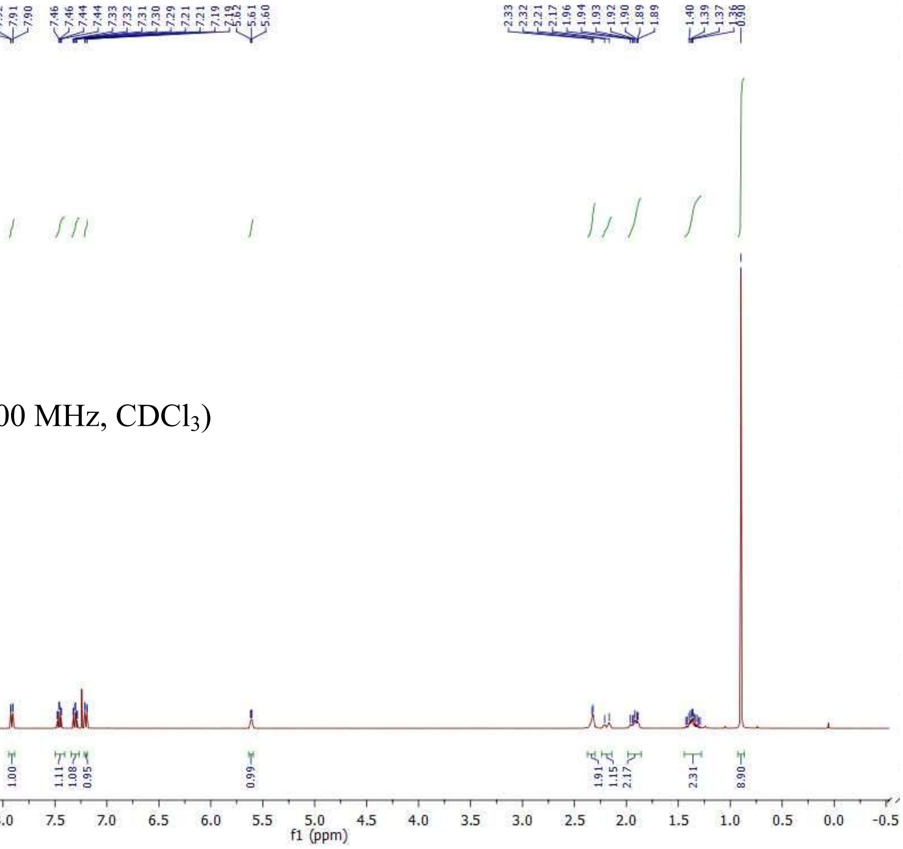

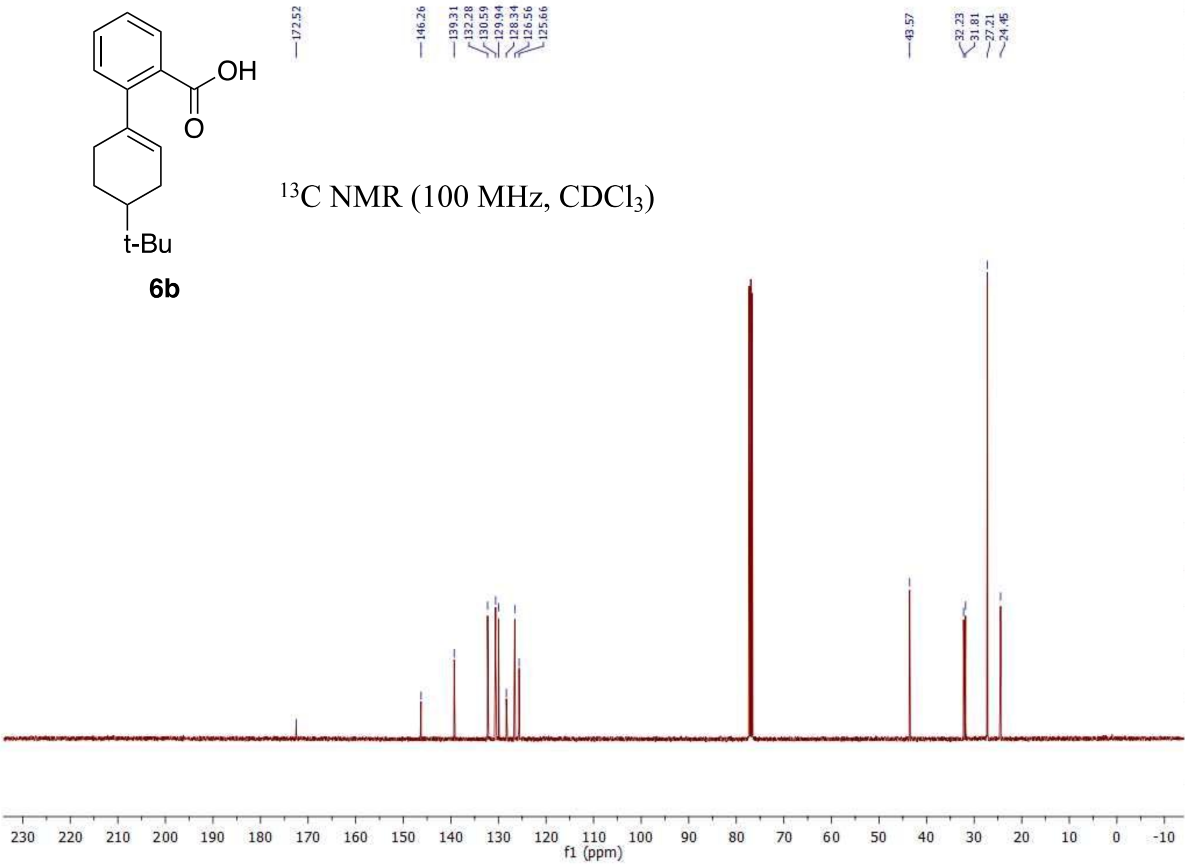

^{13}C NMR (100 MHz, $CDCl_3$)

6b

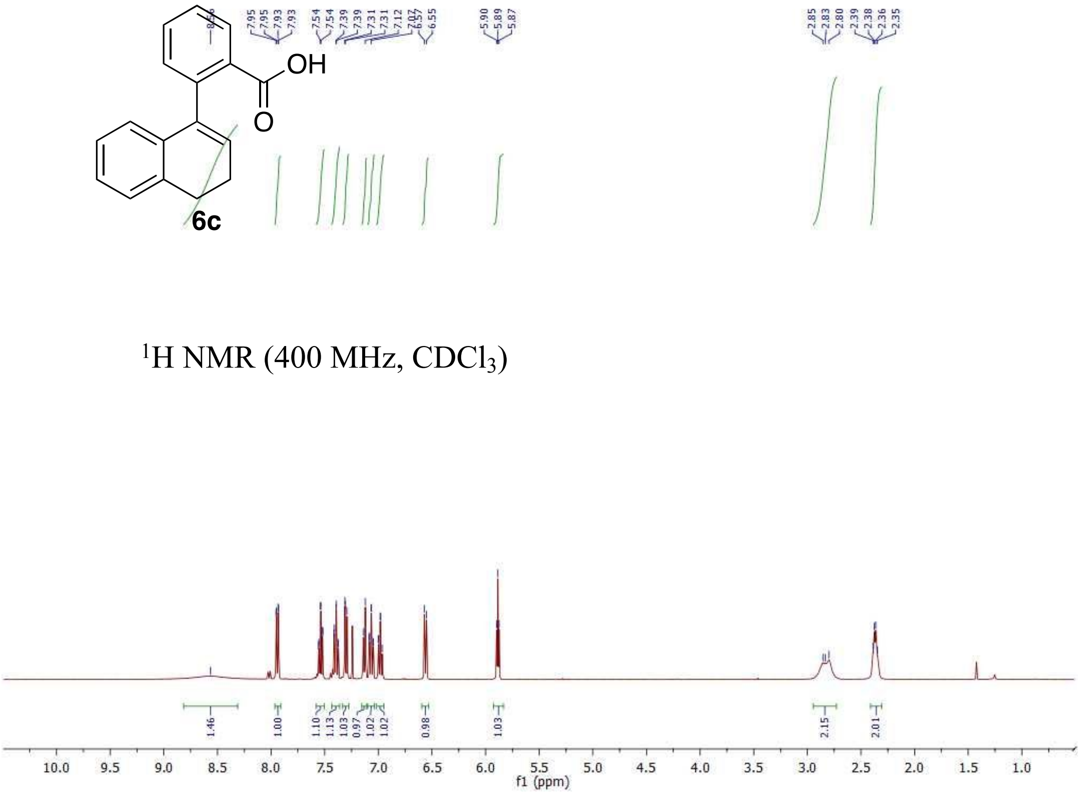

6c

^{1}H NMR (400 MHz, $CDCl_3$)

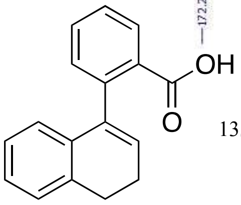

6c

^{13}C NMR (100 MHz, $CDCl_3$)

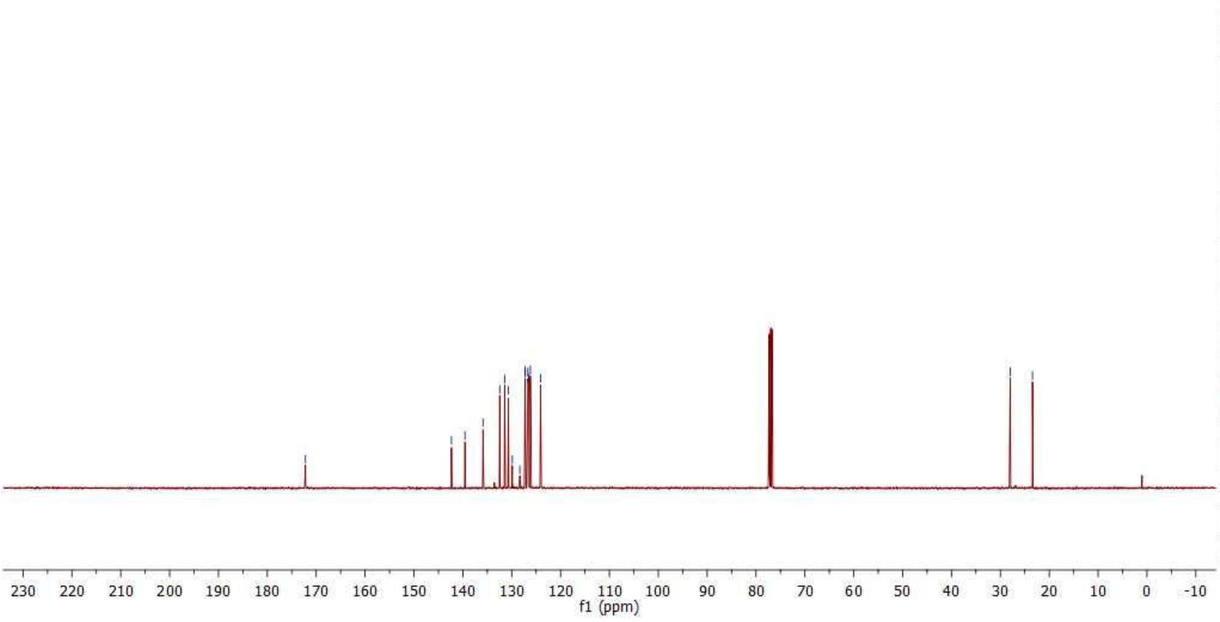

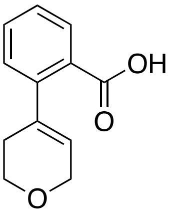

6d

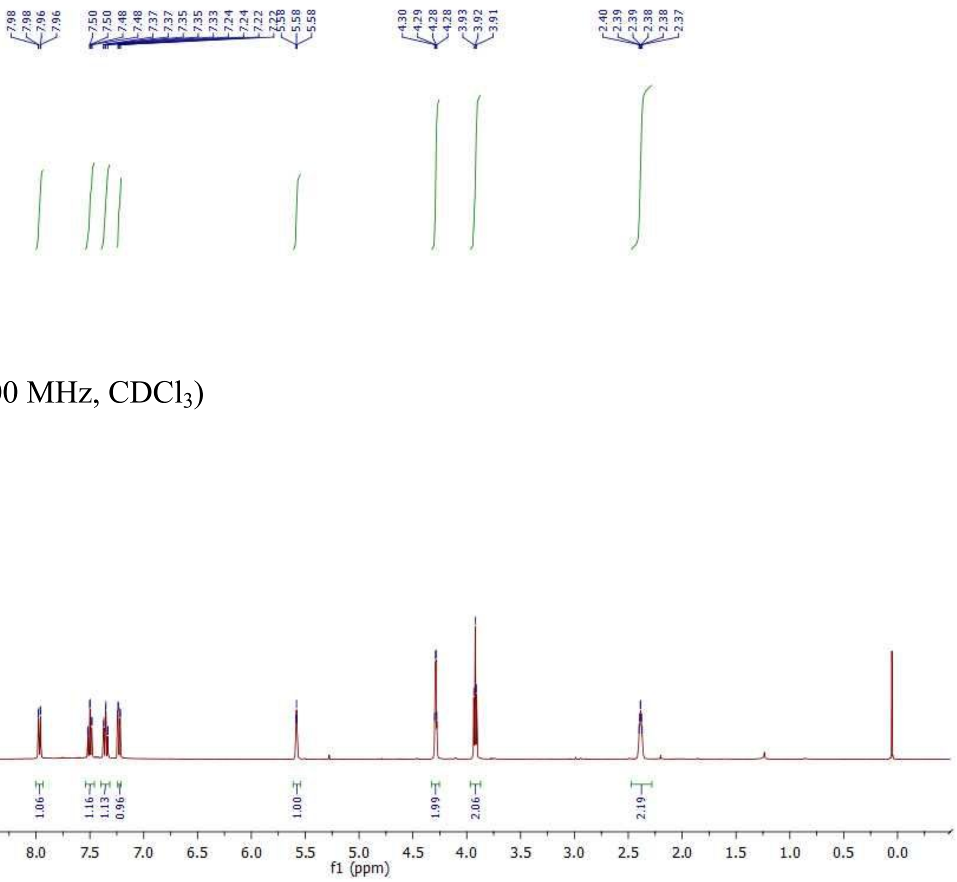

^{1}H NMR (400 MHz, $CDCl_3$)

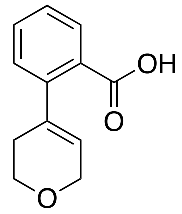

6d

^{13}C NMR (100 MHz, $CDCl_3$)

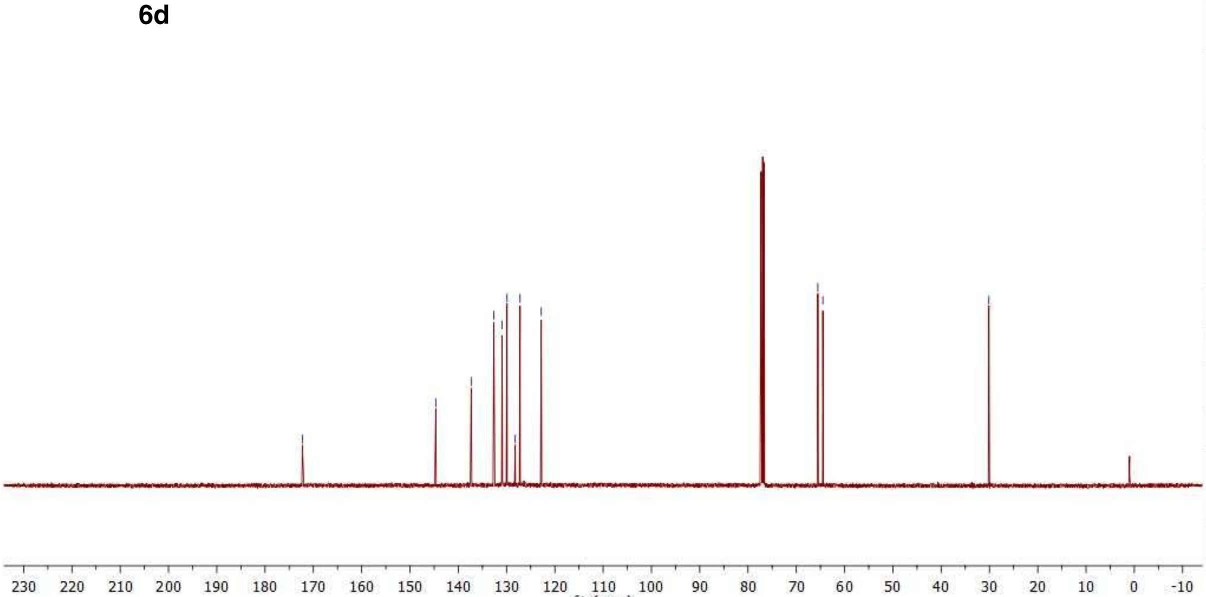

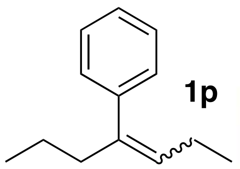

1p

^{1}H NMR (400 MHz, $CDCl_3$)

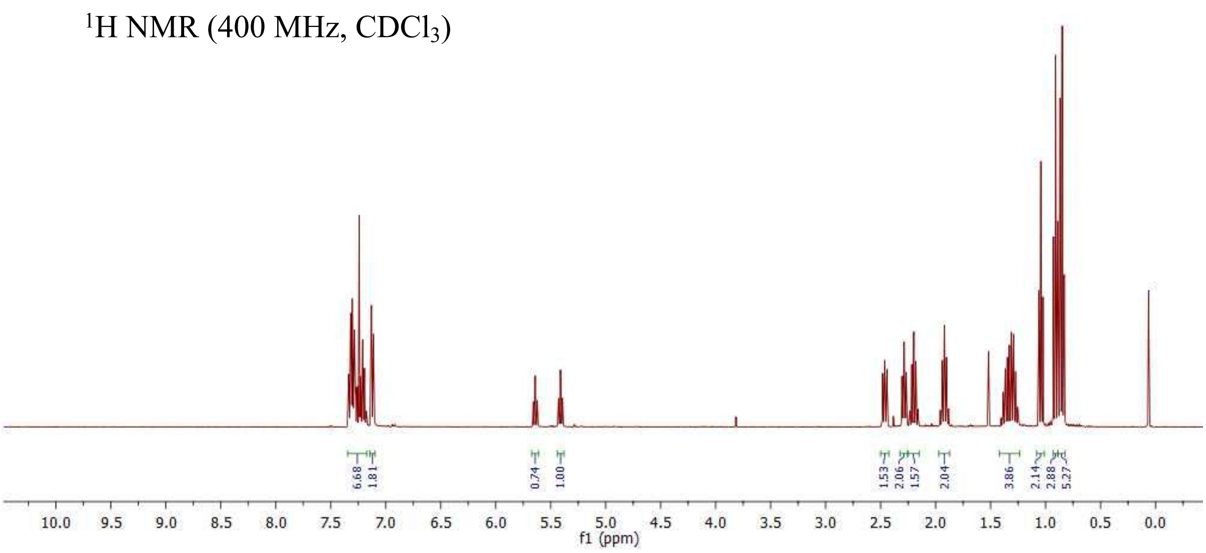

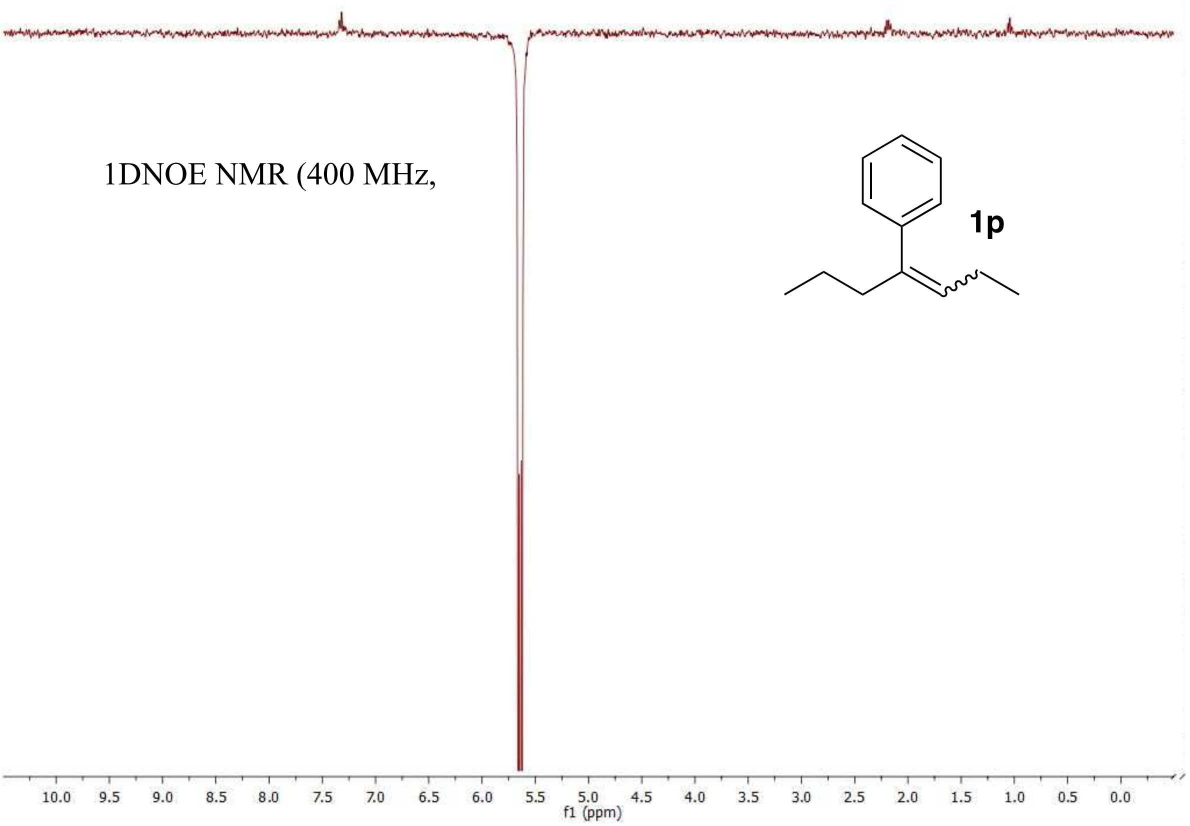
1DNOE NMR (400 MHz,
1p
10.0 9.5 9.0 8.5 8.0 7.5 7.0 6.5 6.0 5.5 5.0 4.5 4.0 3.5 3.0 2.5 2.0 1.5 1.0 0.5 0.0
f1 (ppm)

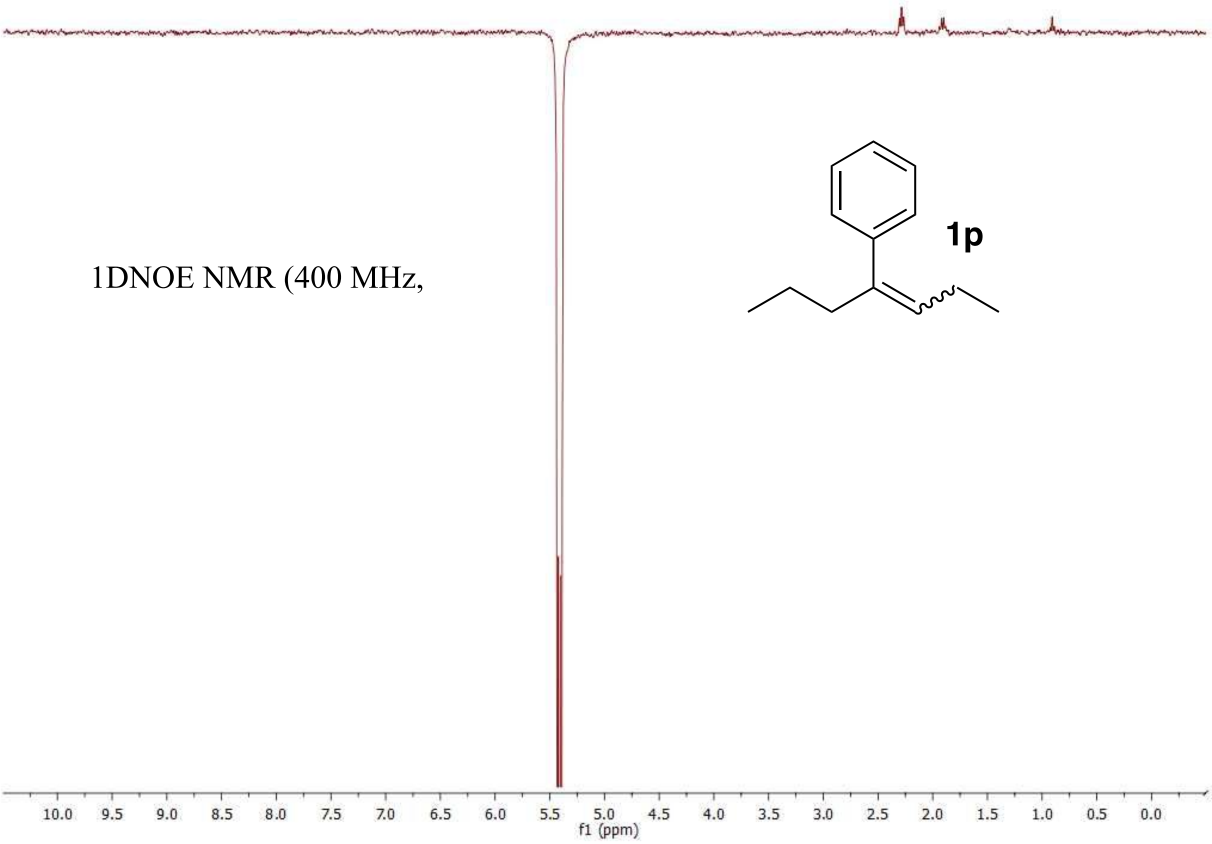
1DNOE NMR (400 MHz,
1p
10.0 9.5 9.0 8.5 8.0 7.5 7.0 6.5 6.0 5.5 5.0 4.5 4.0 3.5 3.0 2.5 2.0 1.5 1.0 0.5 0.0
f1 (ppm)

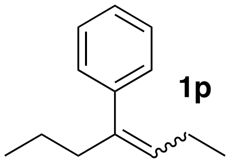

1p

^{13}C NMR (100 MHz, $CDCl_3$)

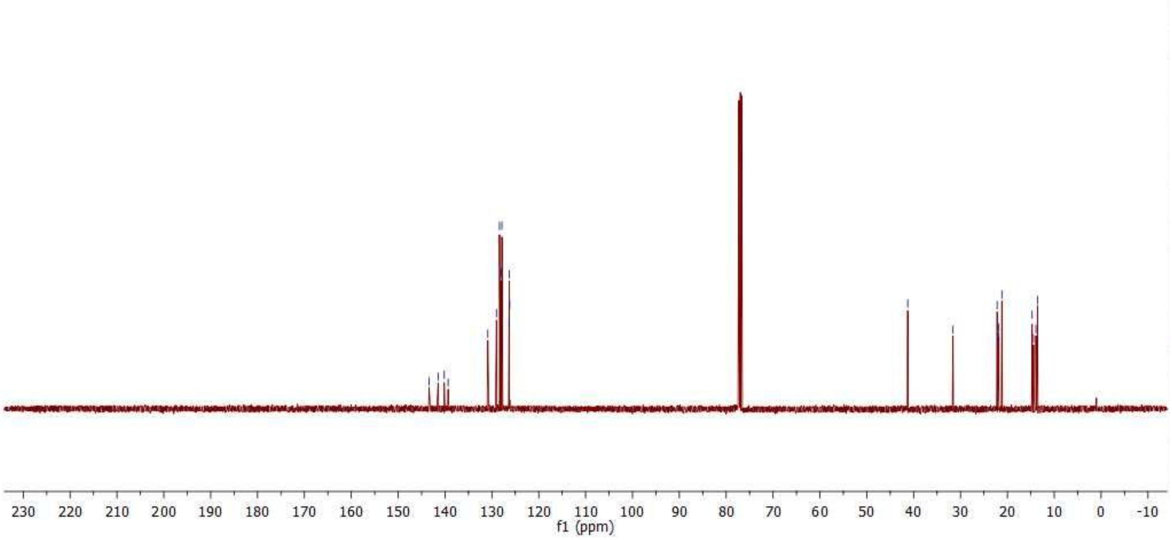

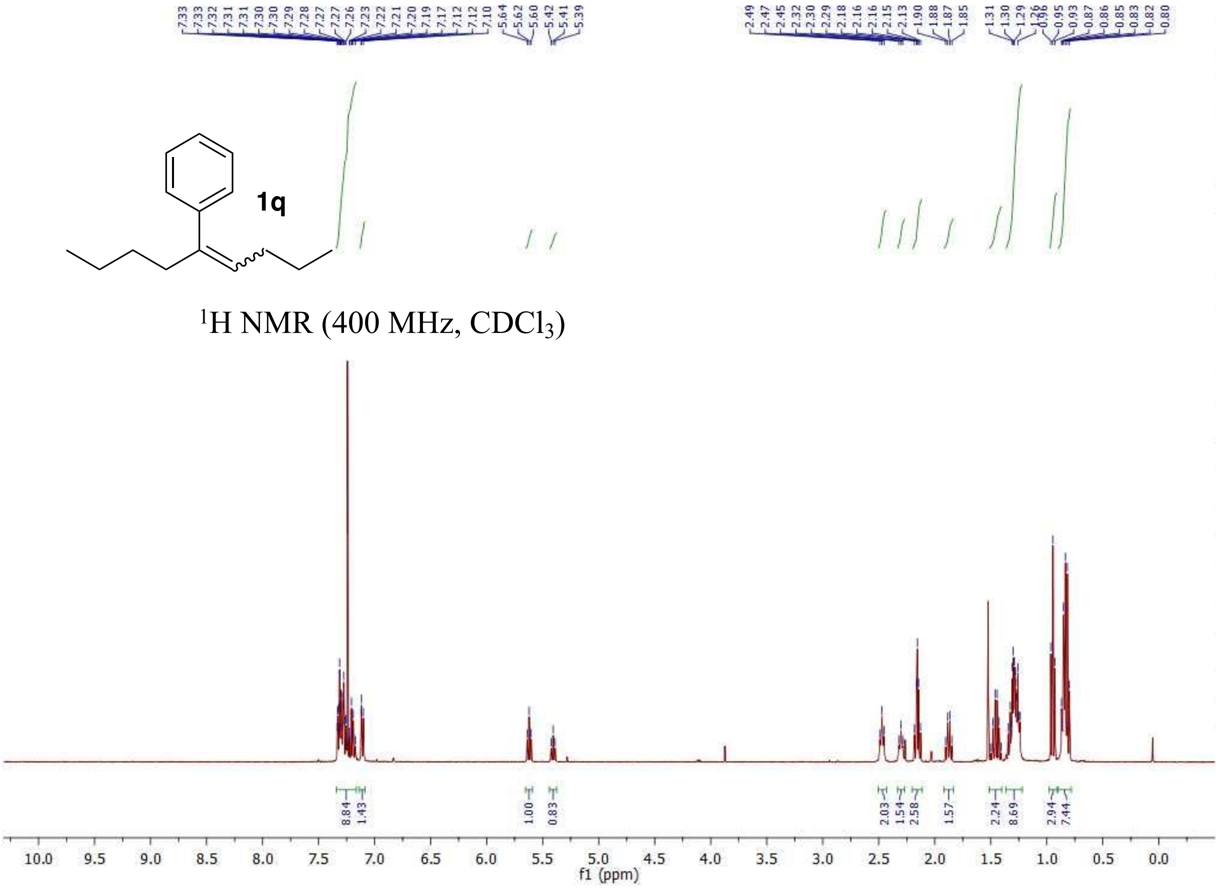

^{1}H NMR (400 MHz, $CDCl_3$)

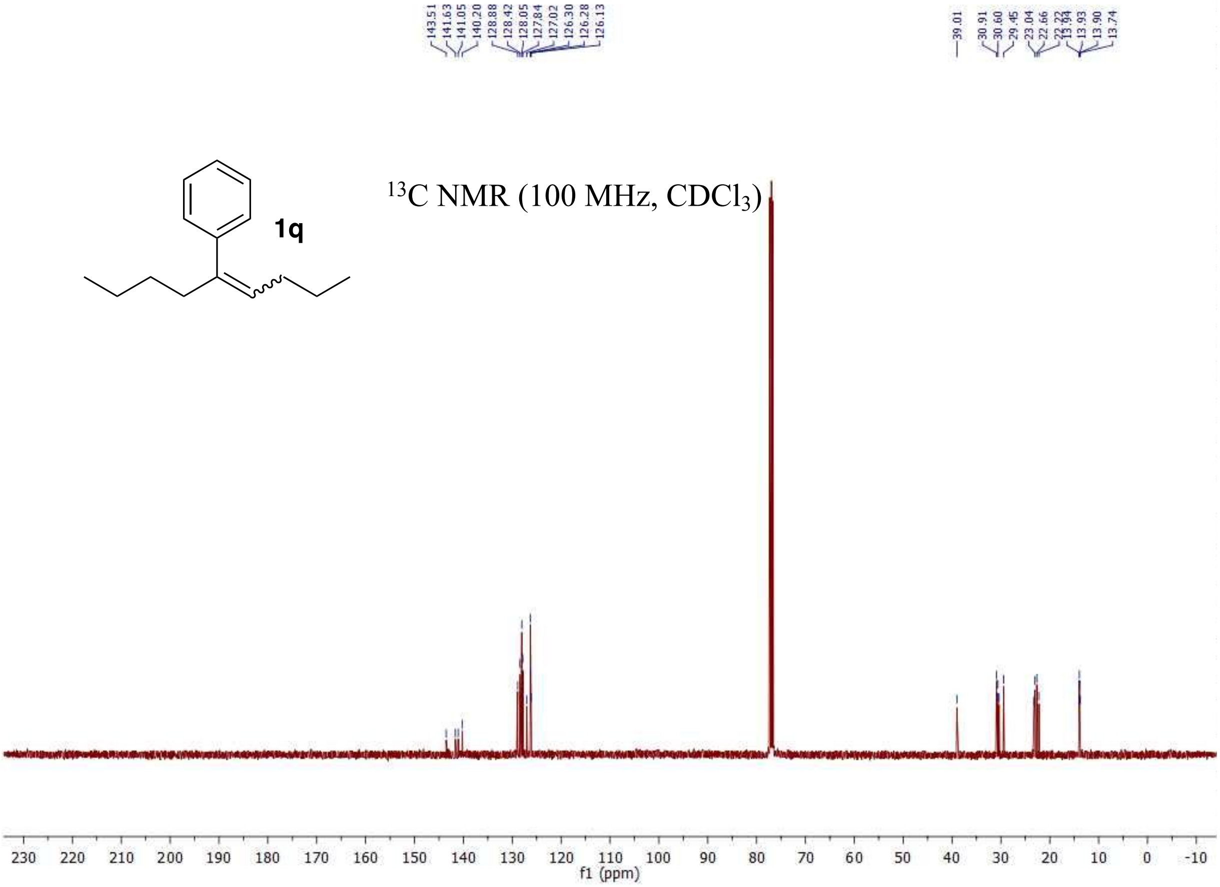

^{13}C NMR (100 MHz, $CDCl_3$)

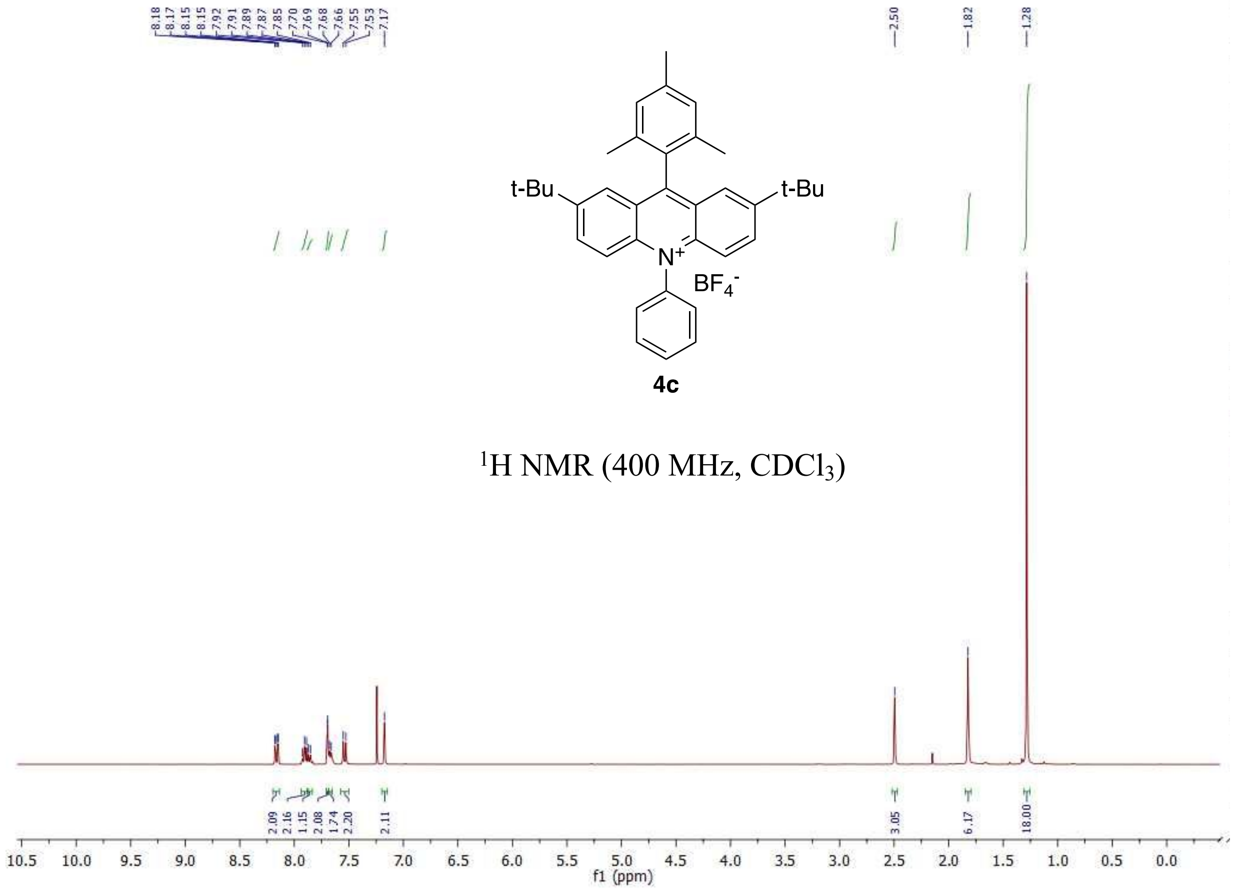

^{1}H NMR (400 MHz, $CDCl_3$)

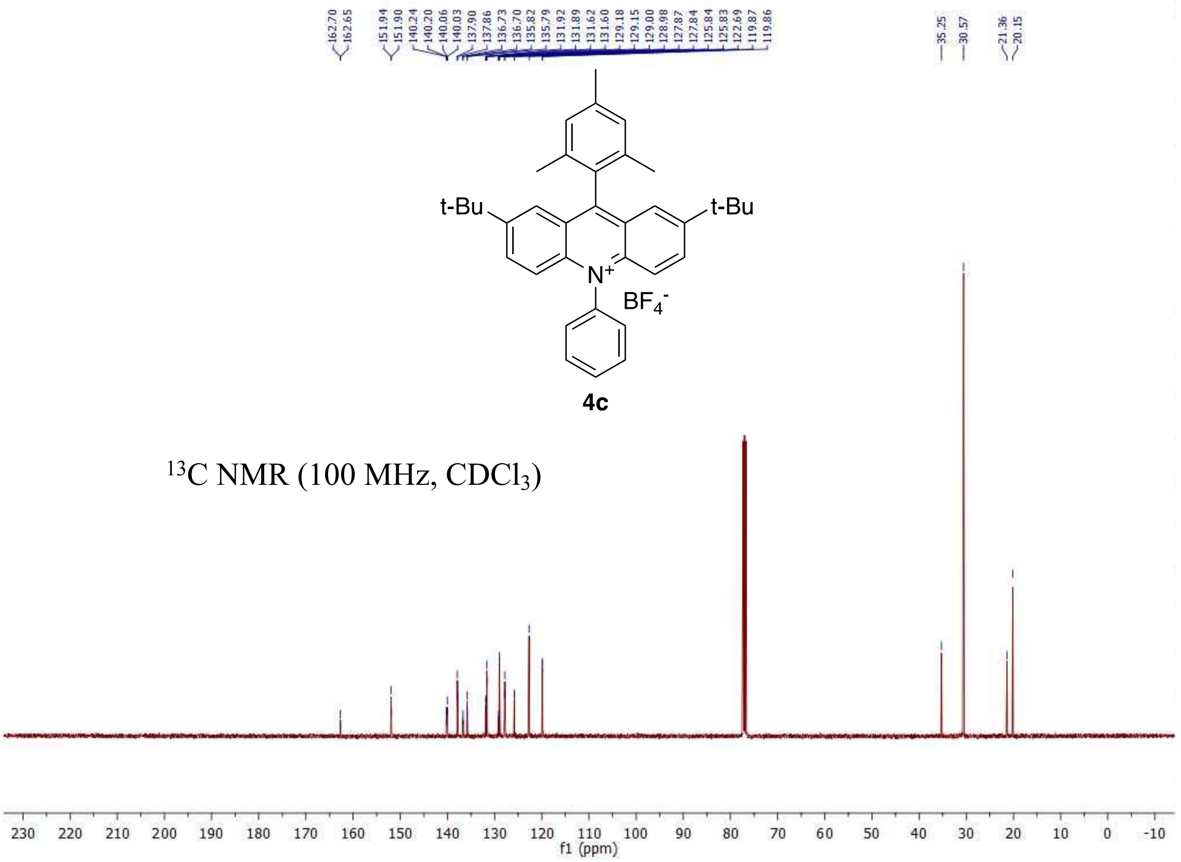

4c

^{13}C NMR (100 MHz, $CDCl_3$)

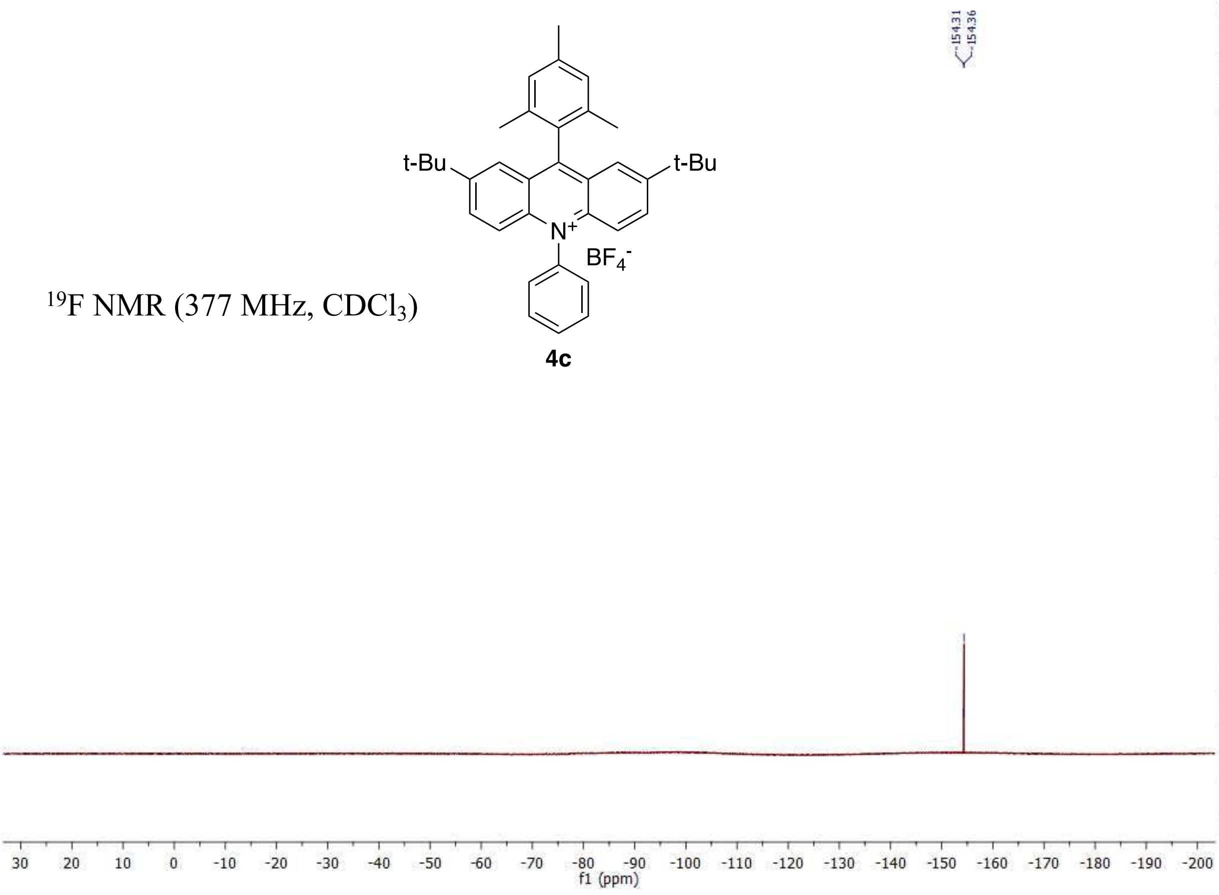

4c

^{19}F NMR (377 MHz, $CDCl_3$)

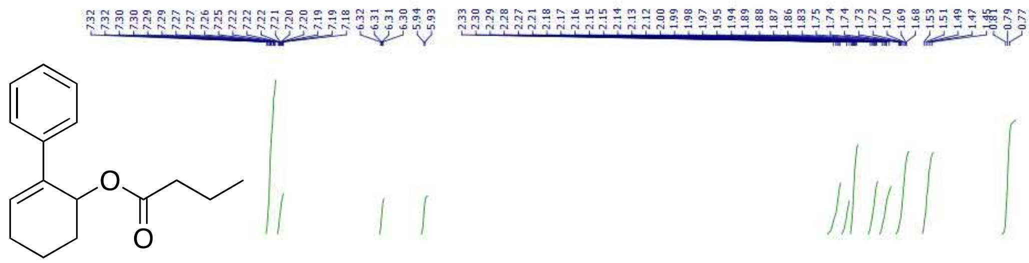

5aa

^1H NMR (400 MHz, $CDCl_3$)

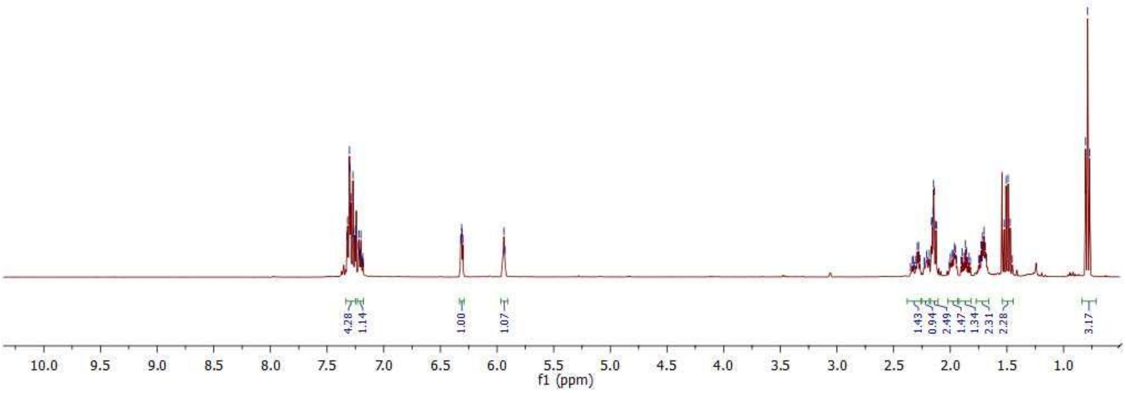

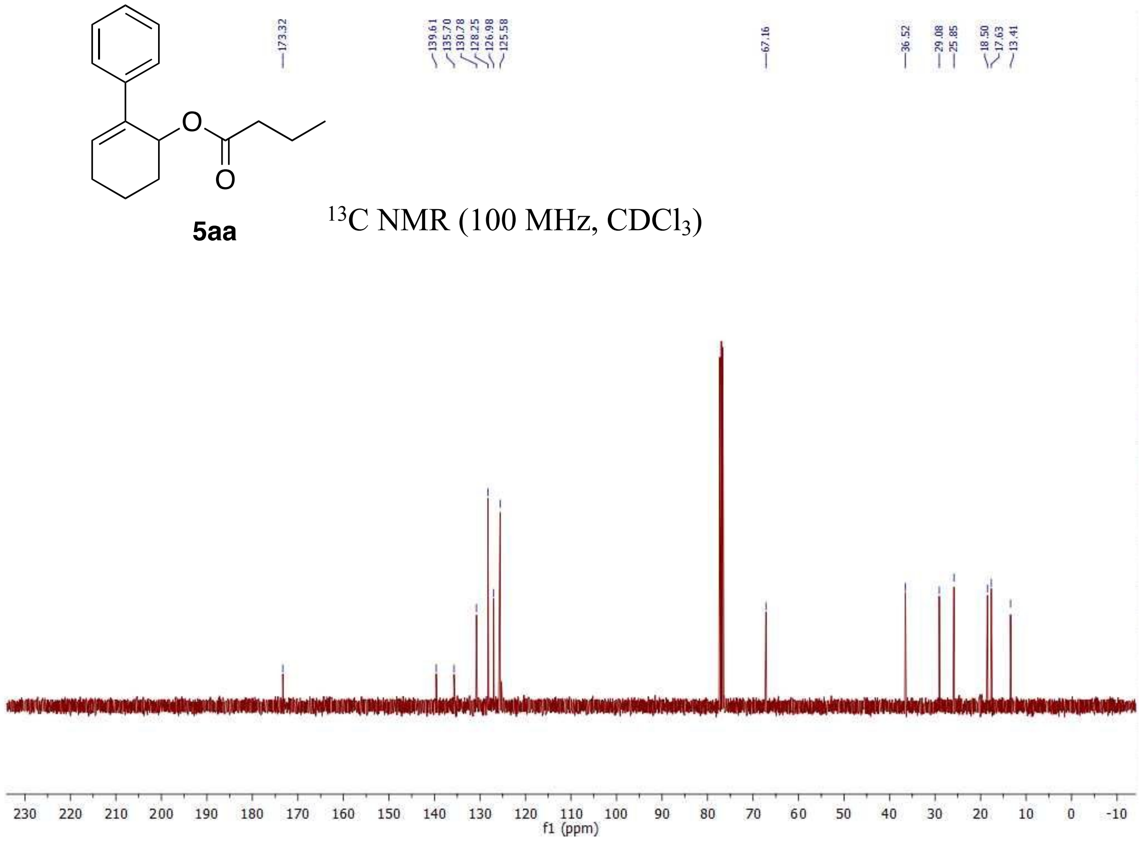

5aa

^{13}C NMR (100 MHz, $CDCl_3$)

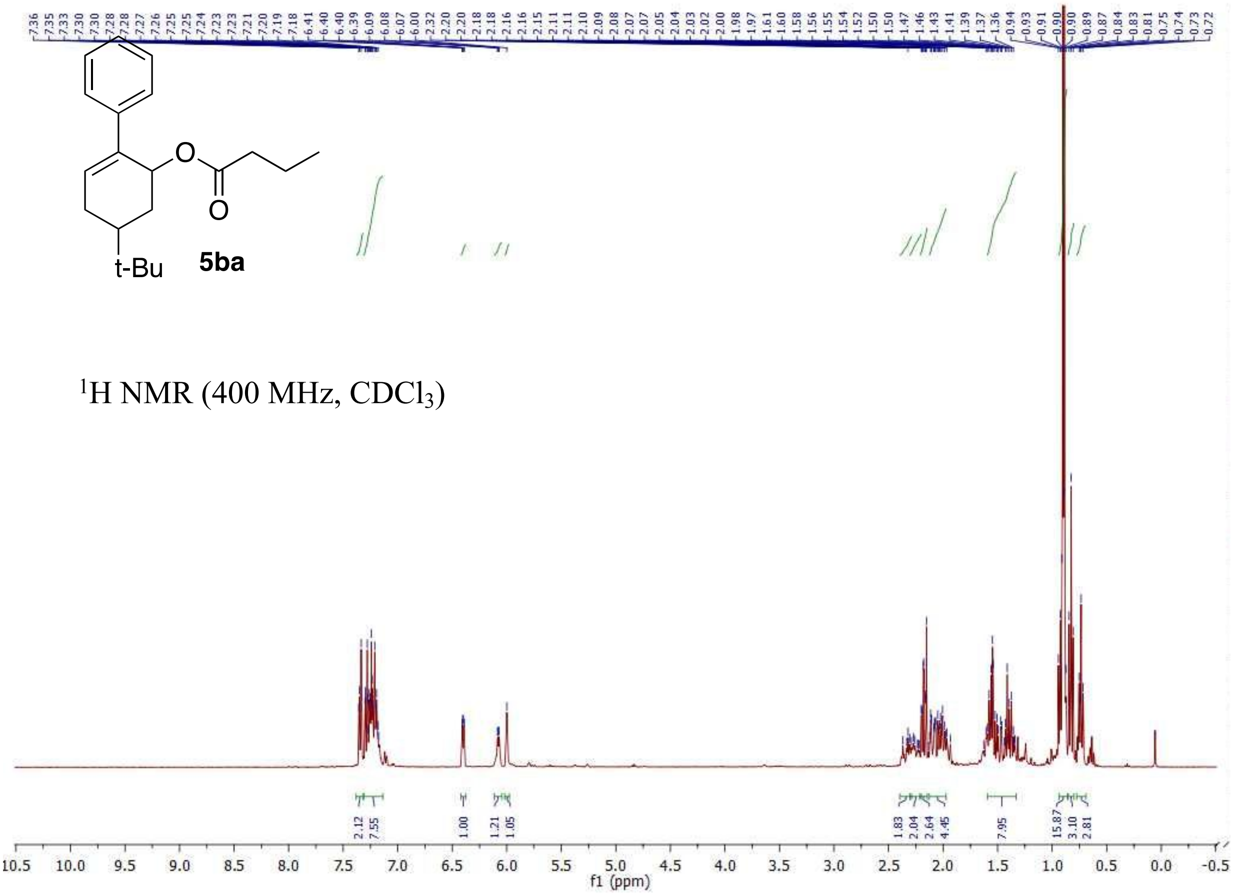

5ba

^{1}H NMR (400 MHz, $CDCl_3$)

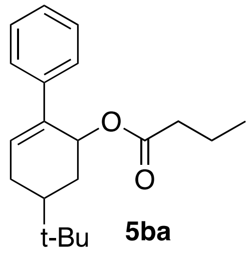

5ba

^{13}C NMR (100 MHz, $CDCl_3$)

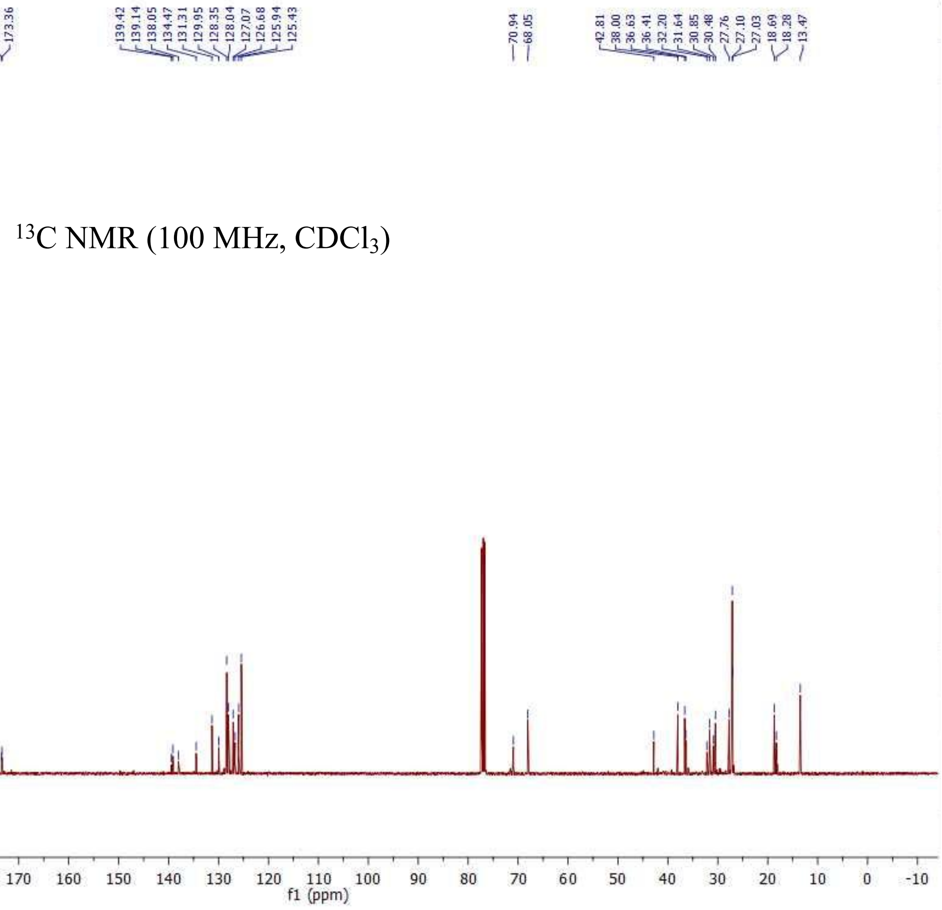

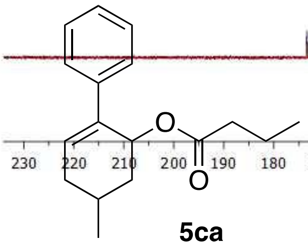

5ca

^{1}H NMR (400 MHz, $CDCl_3$)

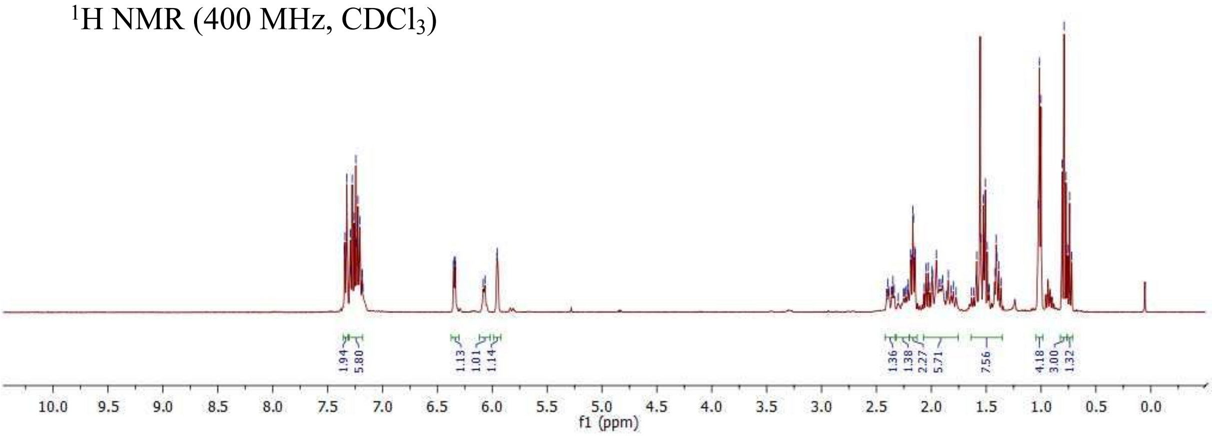

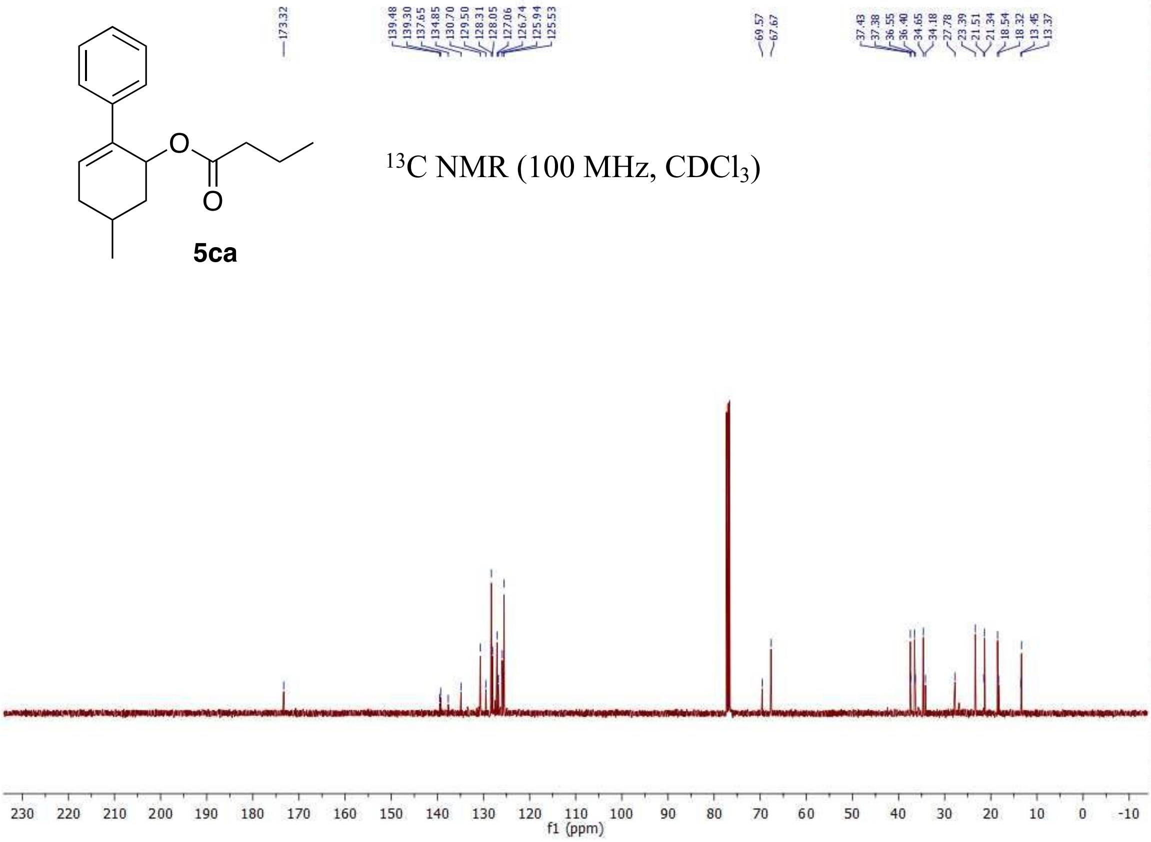
5ca
^{13}C NMR (100 MHz, $CDCl_3$)
f1 (ppm)

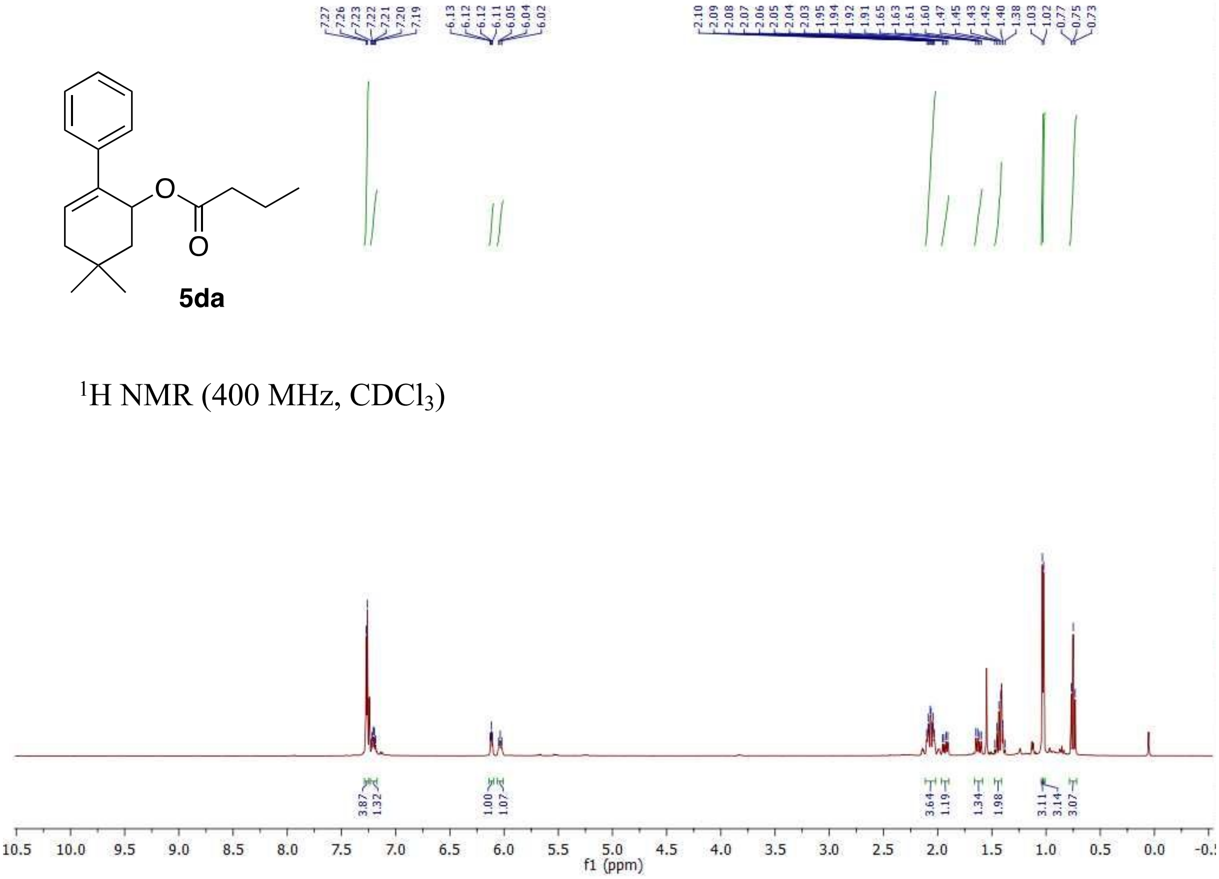
5da
^{1}H NMR (400 MHz, $CDCl_3$)
f1 (ppm)

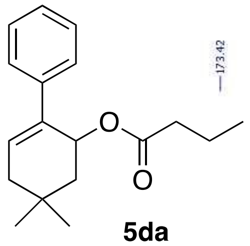

5da

^{13}C NMR (100 MHz, $CDCl_3$)

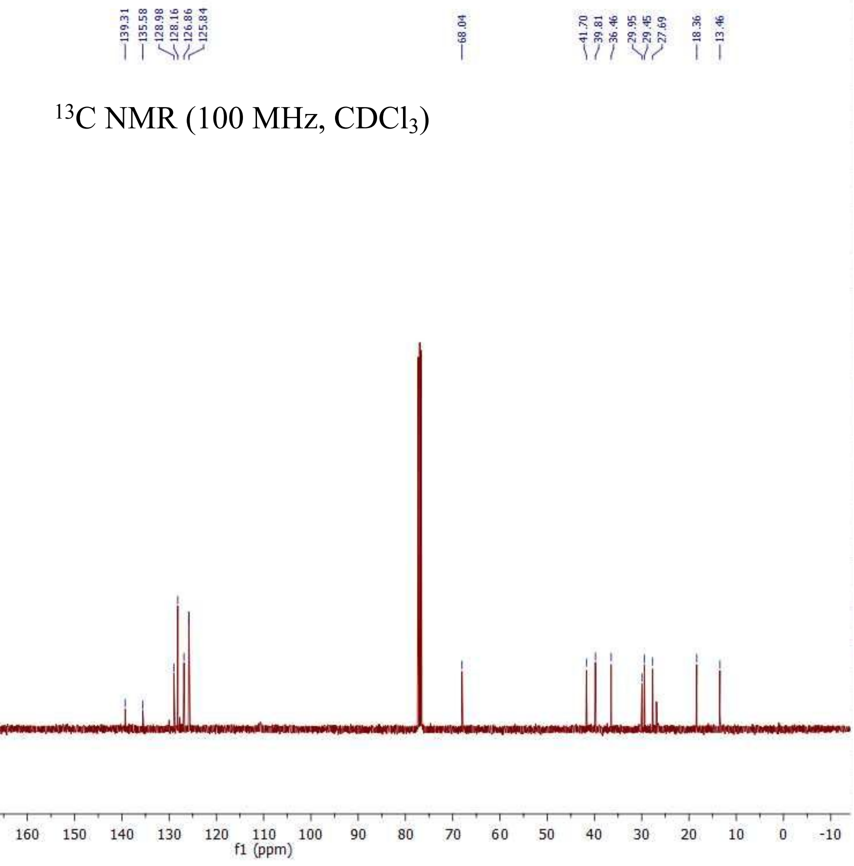

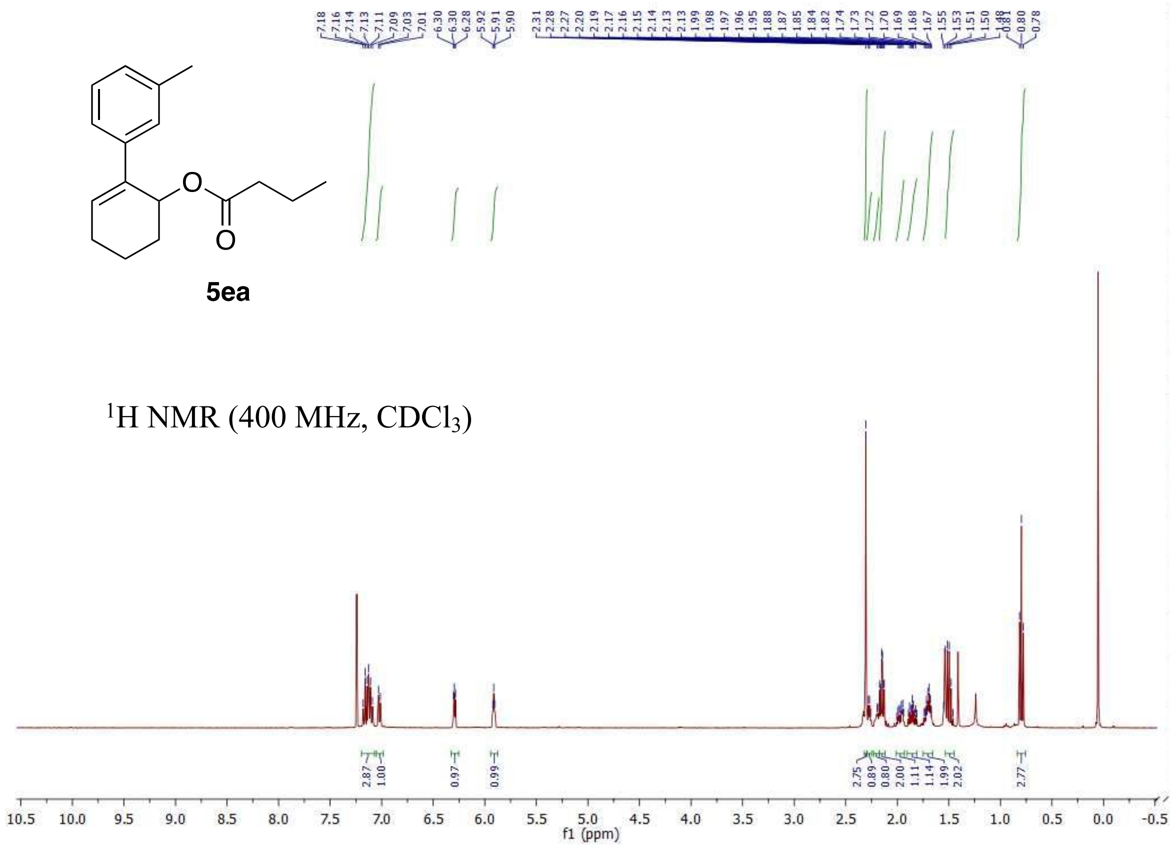

5ea

^{1}H NMR (400 MHz, $CDCl_3$)

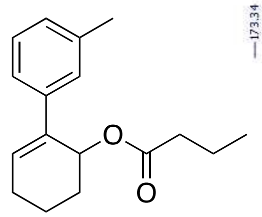

5ea

^{13}C NMR (100 MHz, $CDCl_3$)

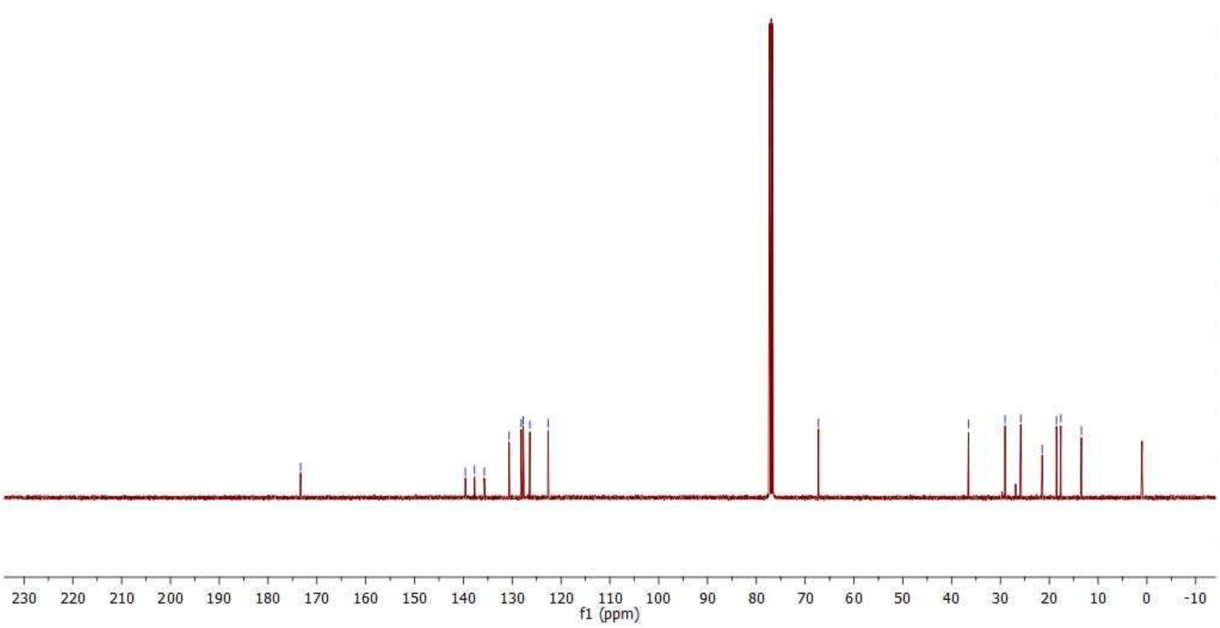

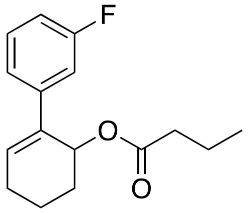

5fa

^{1}H NMR (400 MHz, $CDCl_3$)

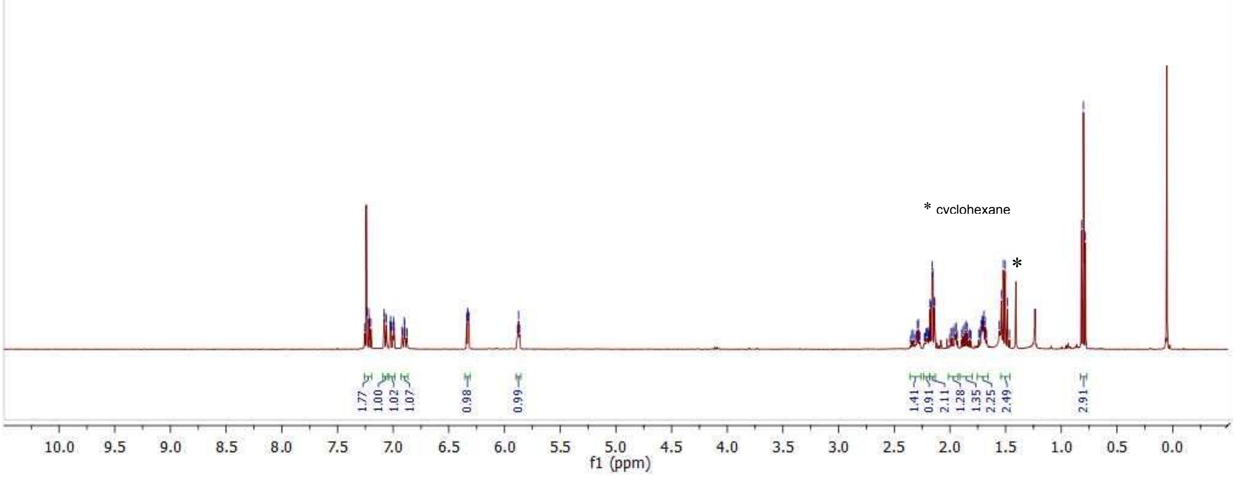

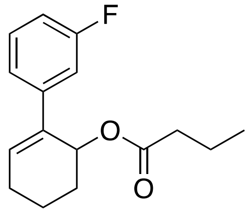

5fa

^{13}C NMR (100 MHz, $CDCl_3$)

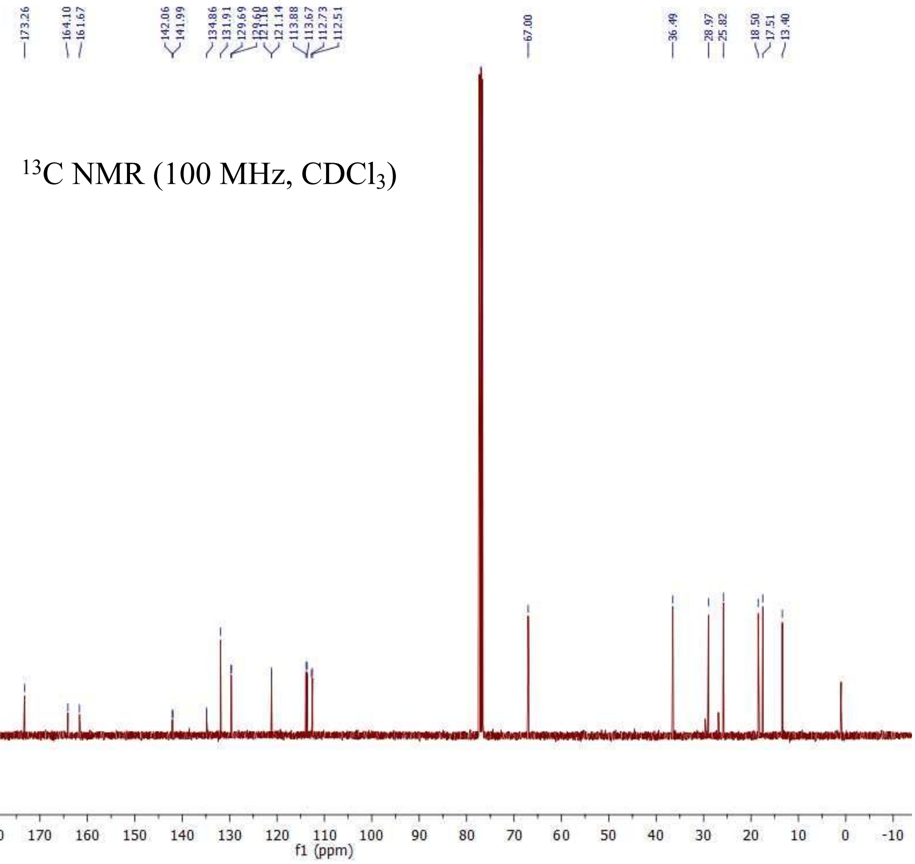

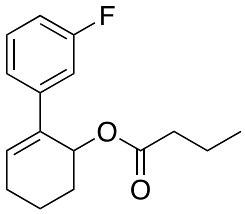

5fa

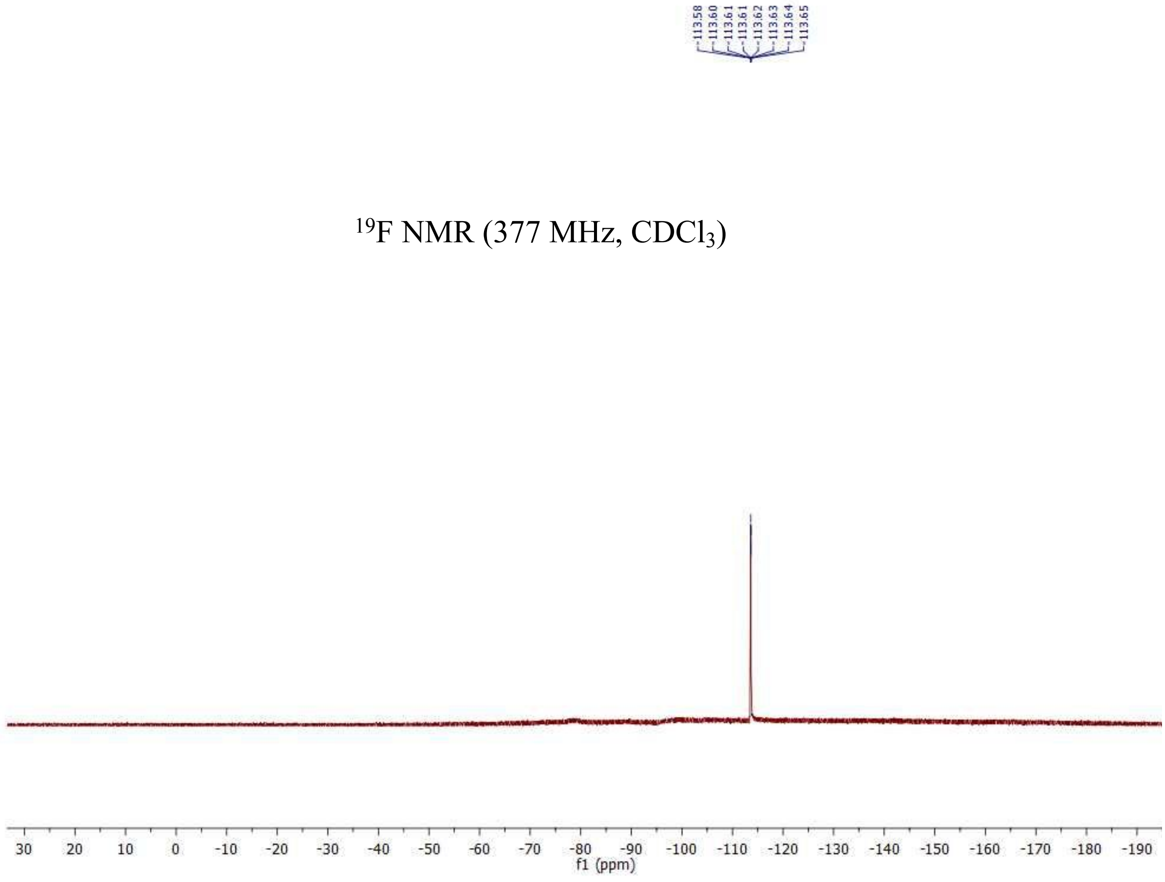

^{19}F NMR (377 MHz, $CDCl_3$)

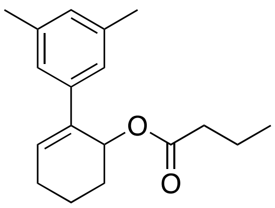

5ga

^{1}H NMR (400 MHz, $CDCl_3$)

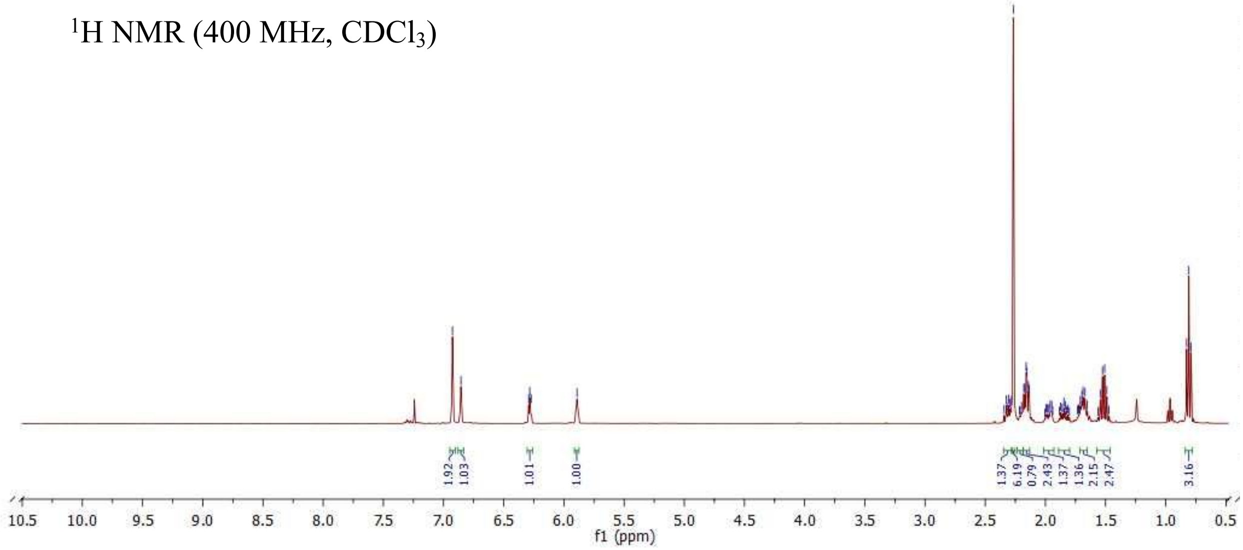

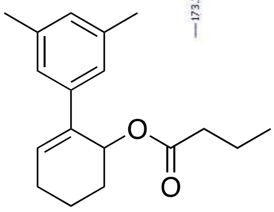

5ga

^{13}C NMR (100 MHz, $CDCl_3$)

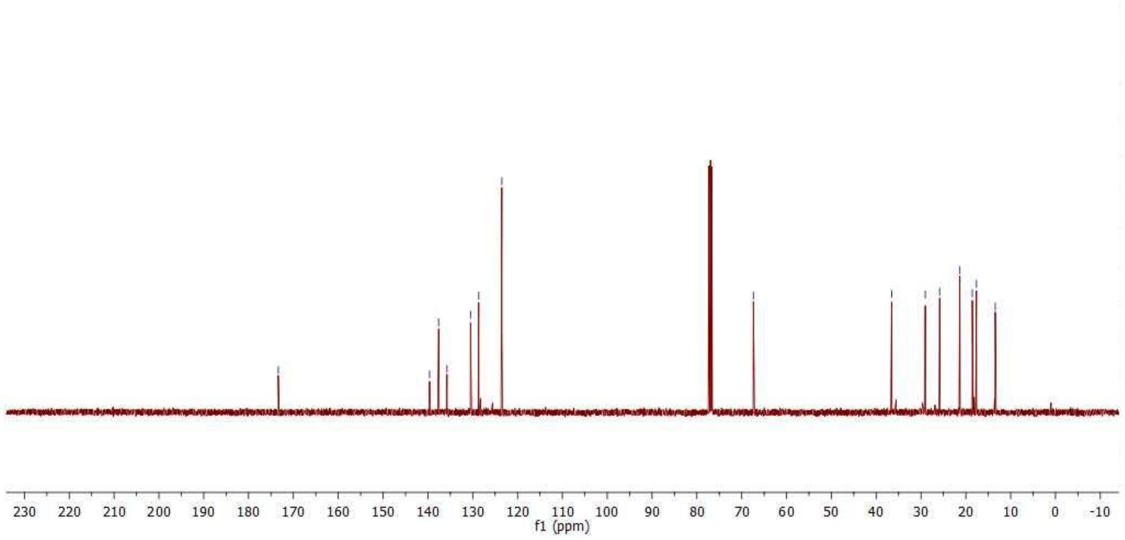

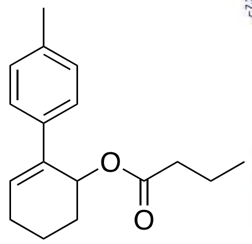

5ha

^{1}H NMR (400 MHz, $CDCl_3$)

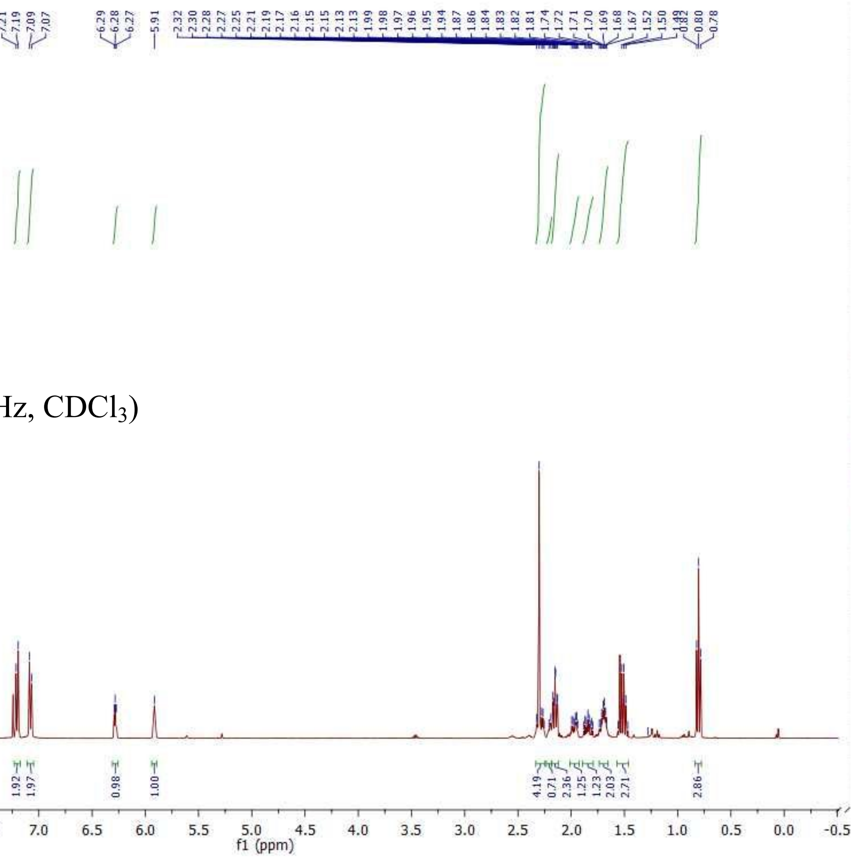

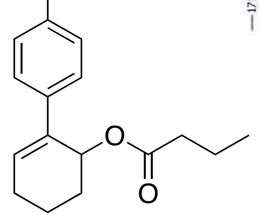

5ha

^{13}C NMR (100 MHz, $CDCl_3$)

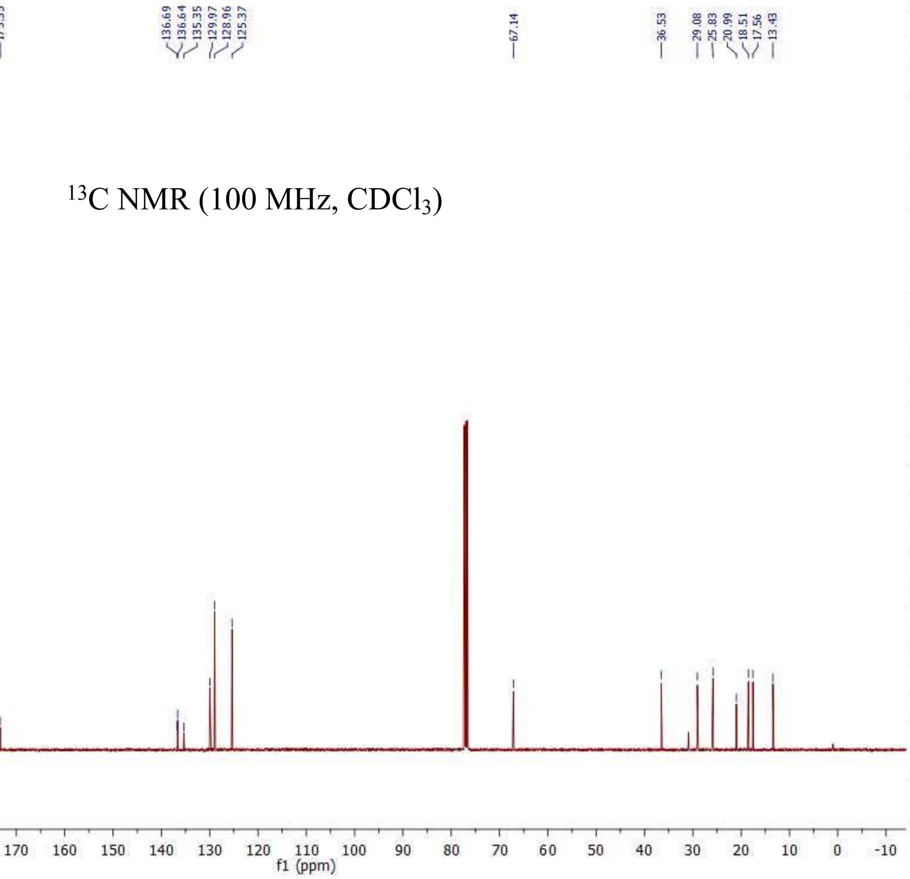

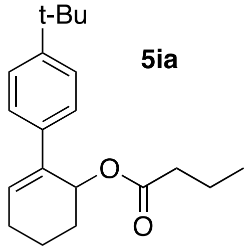

5ia

^1H NMR (400 MHz, $CDCl_3$)

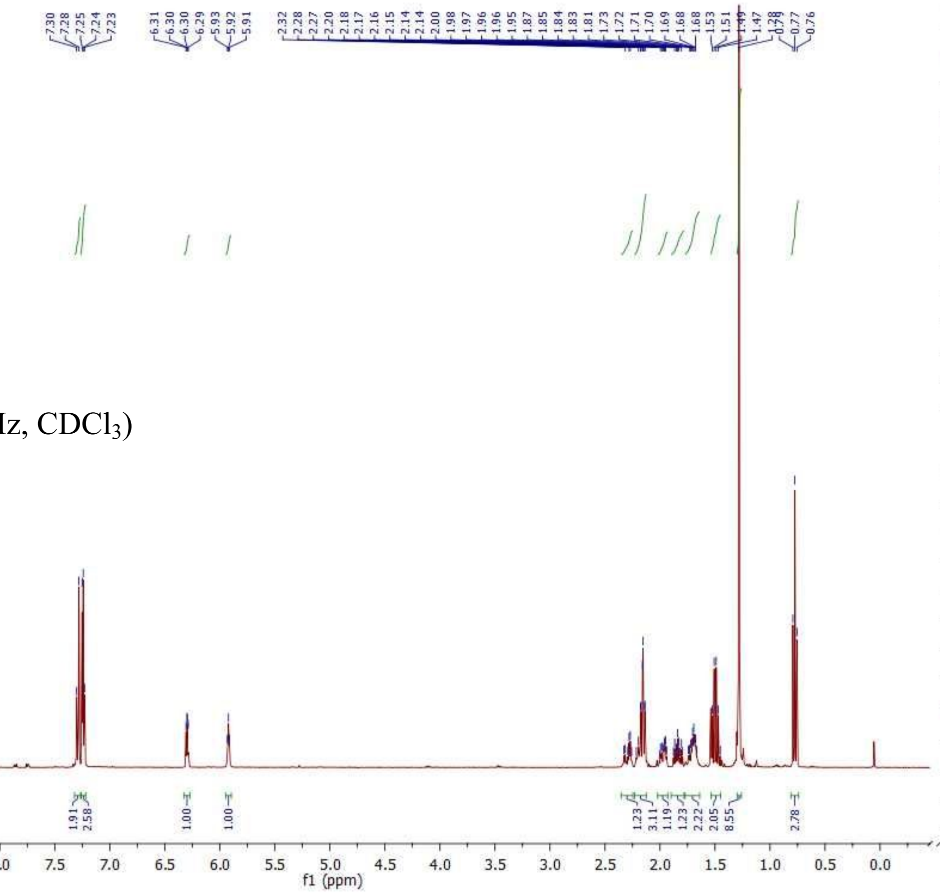

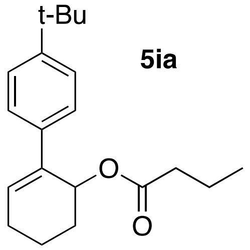

5ia

^{13}C NMR (100 MHz, $CDCl_3$)

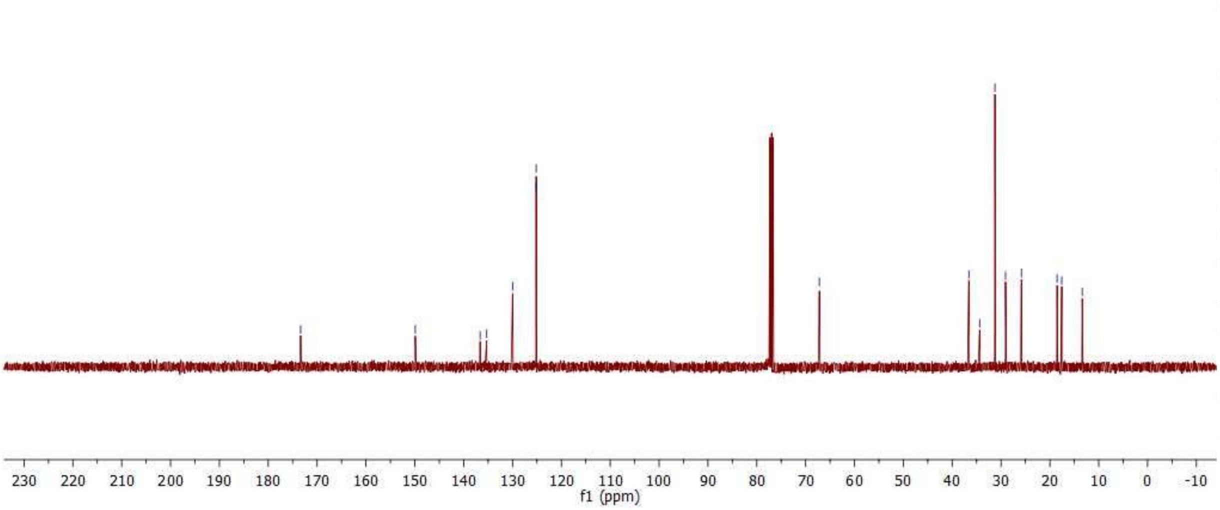

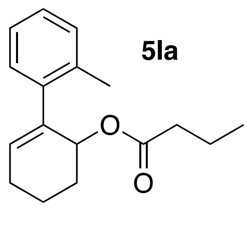

5la

^{1}H NMR (400 MHz, $CDCl_3$)

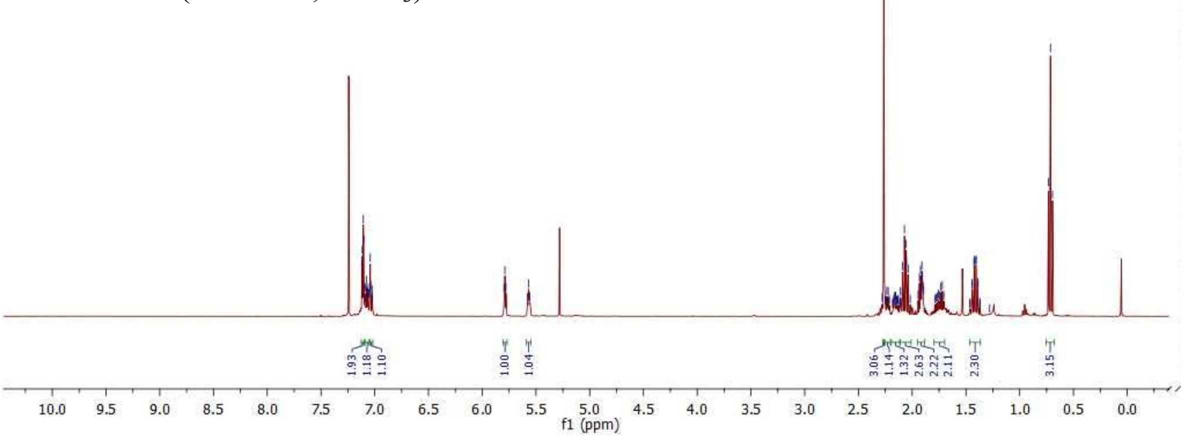

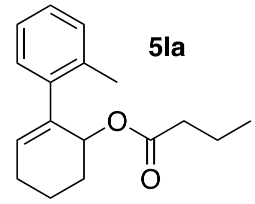

5la

^{13}C NMR (100 MHz, $CDCl_3$)

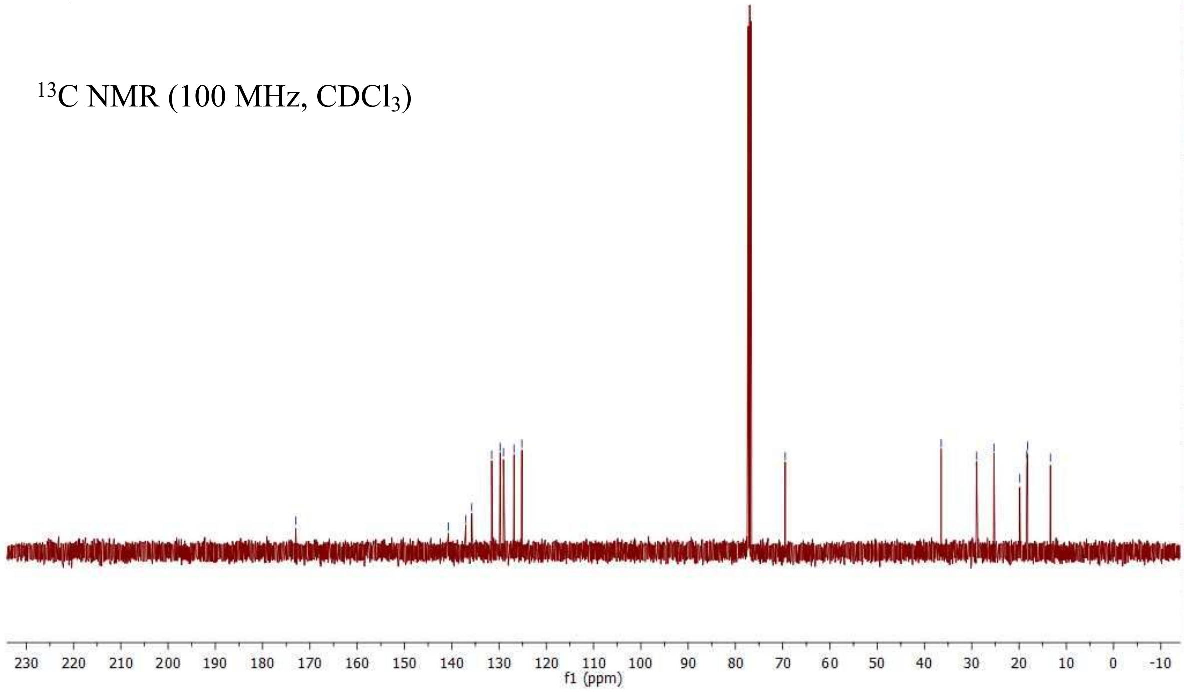

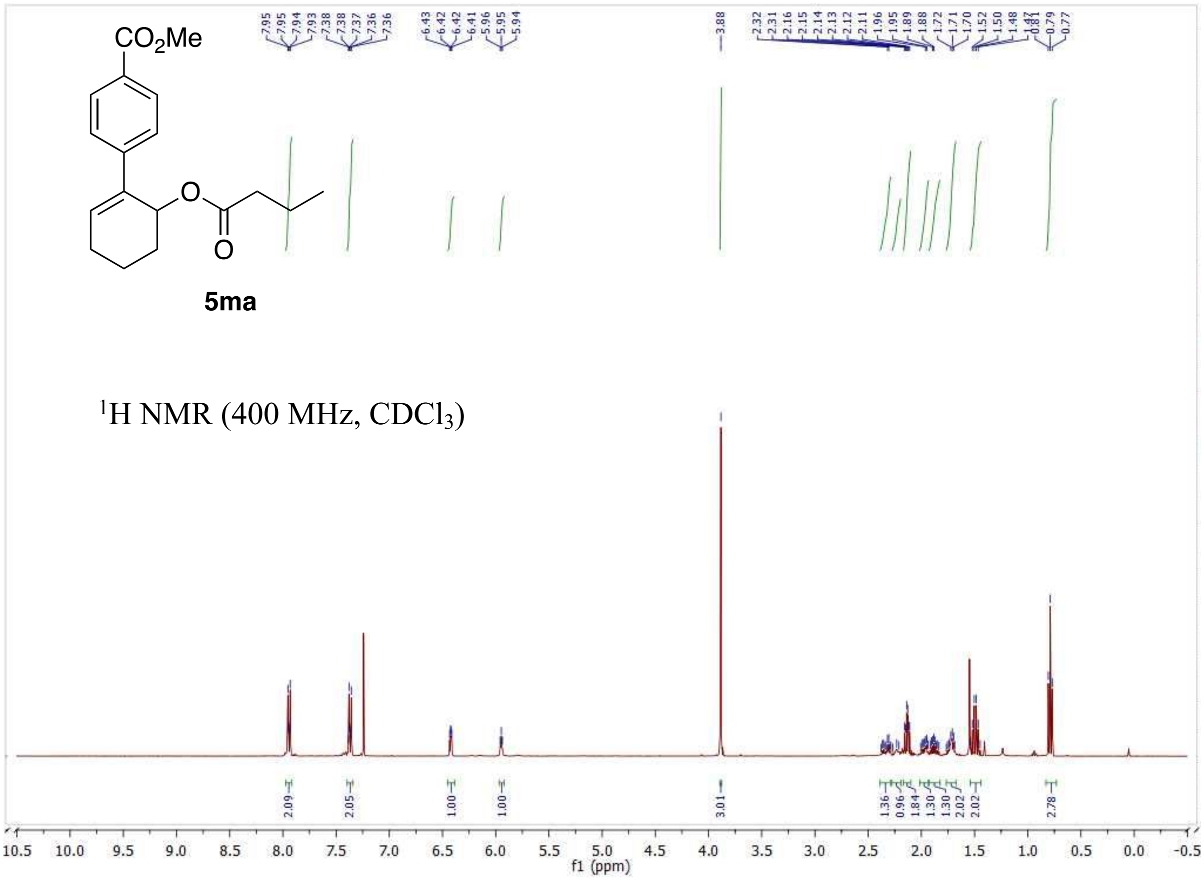

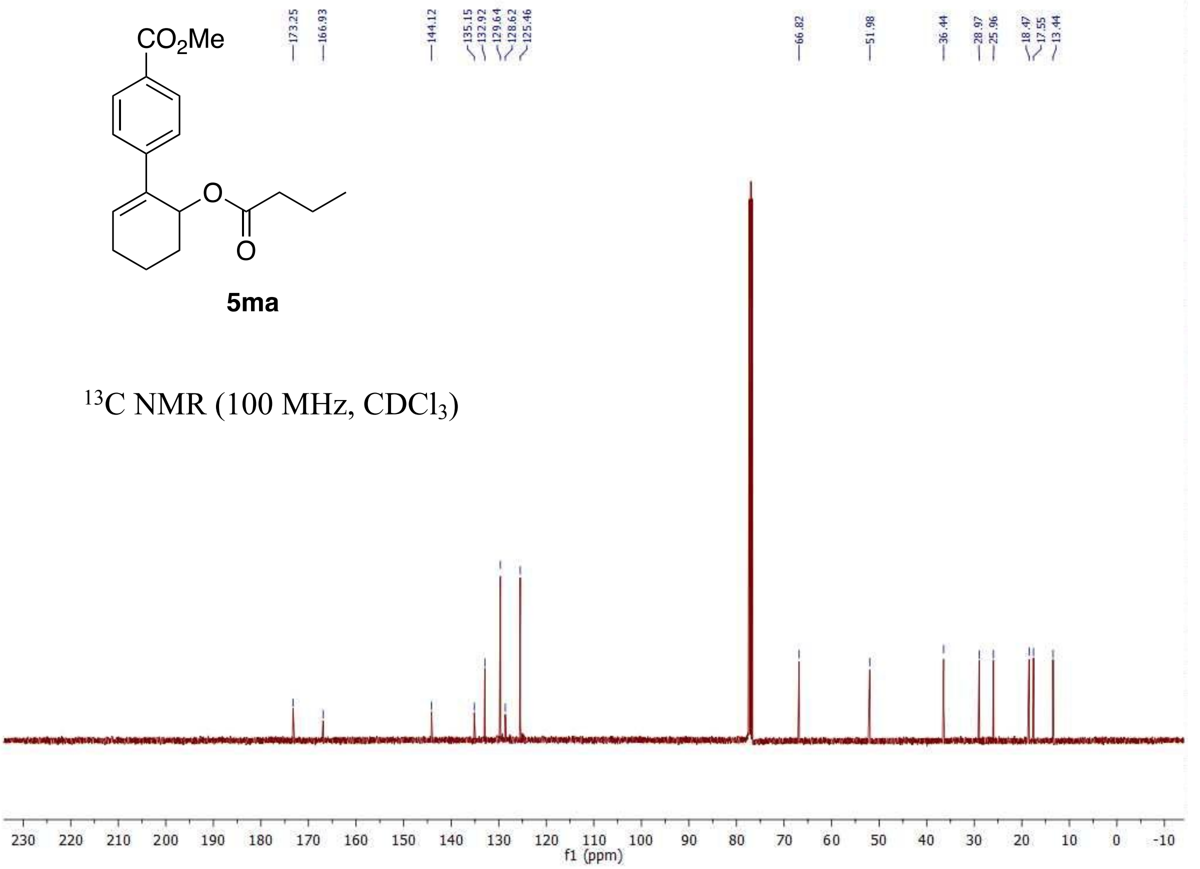

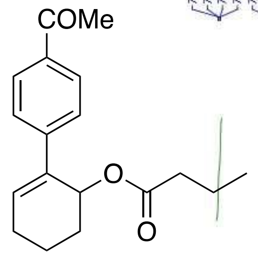

5na

^{1}H NMR (400 MHz, $CDCl_3$)

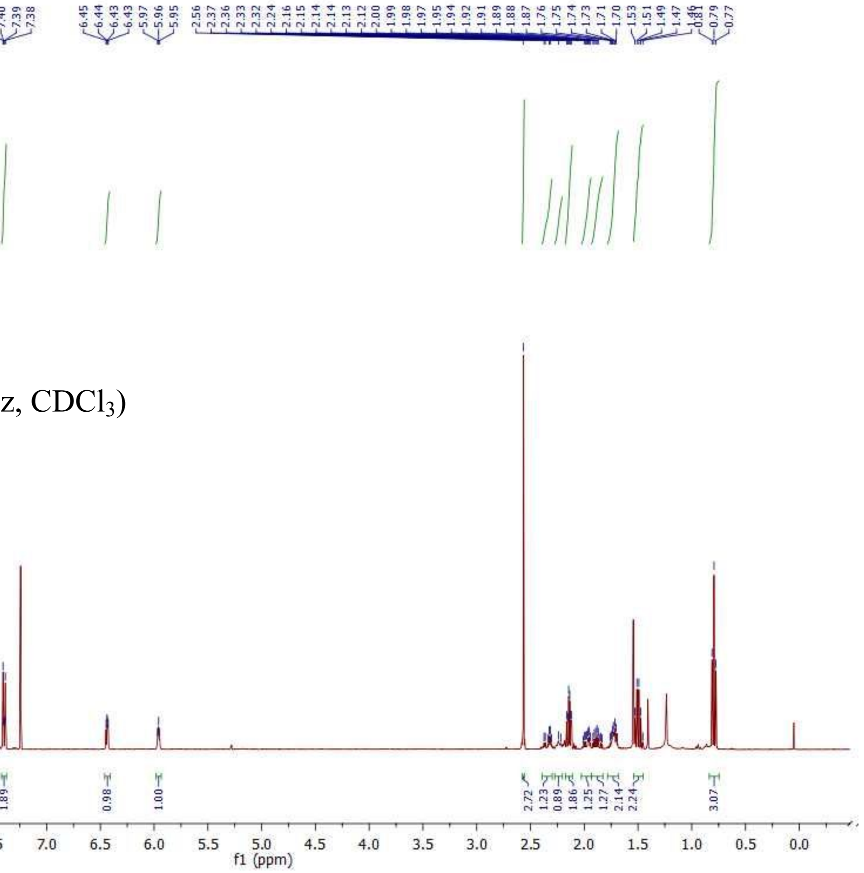

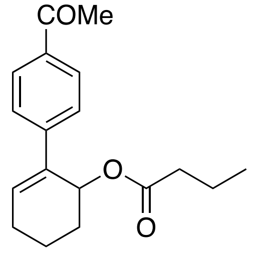

5na

^{13}C NMR (100 MHz, $CDCl_3$)

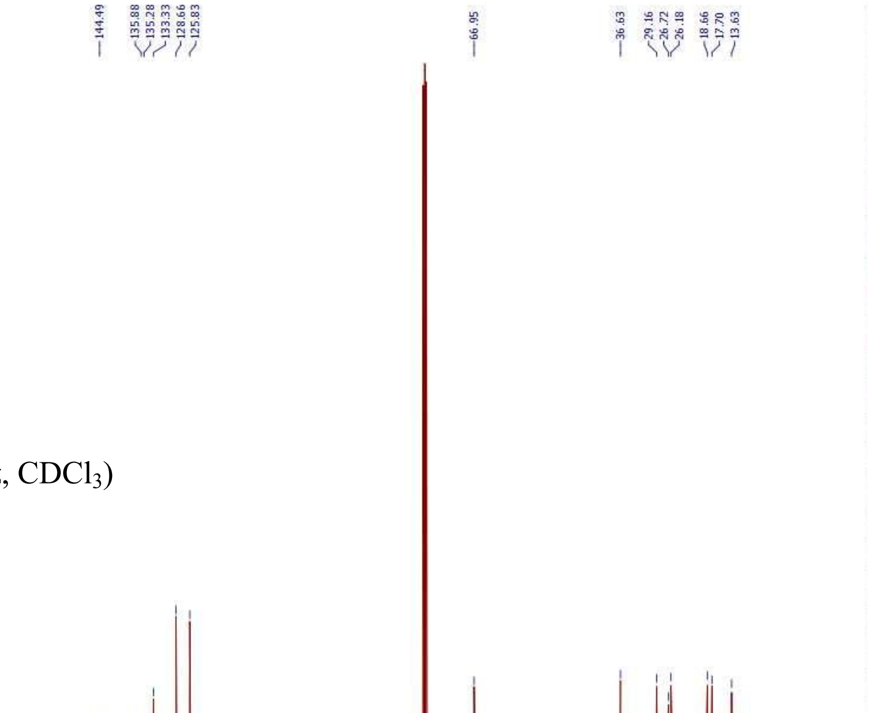

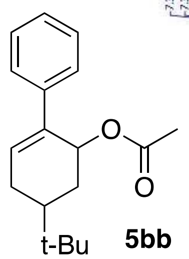

5bb

^{1}H NMR (400 MHz, $CDCl_3$)

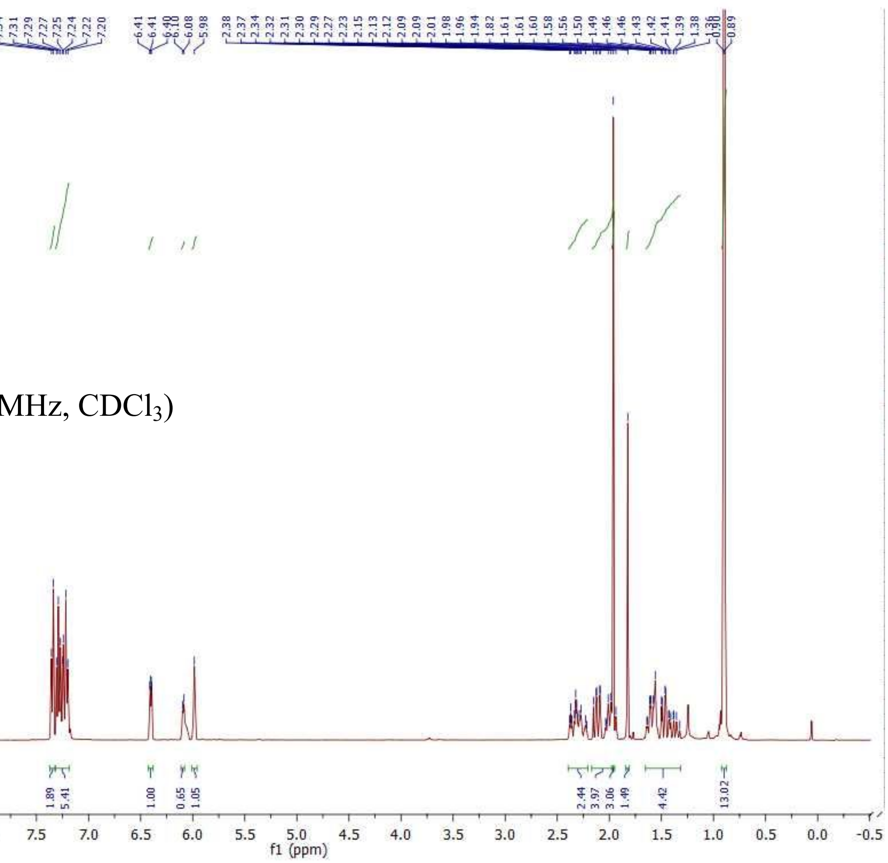

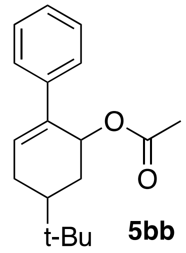

5bb

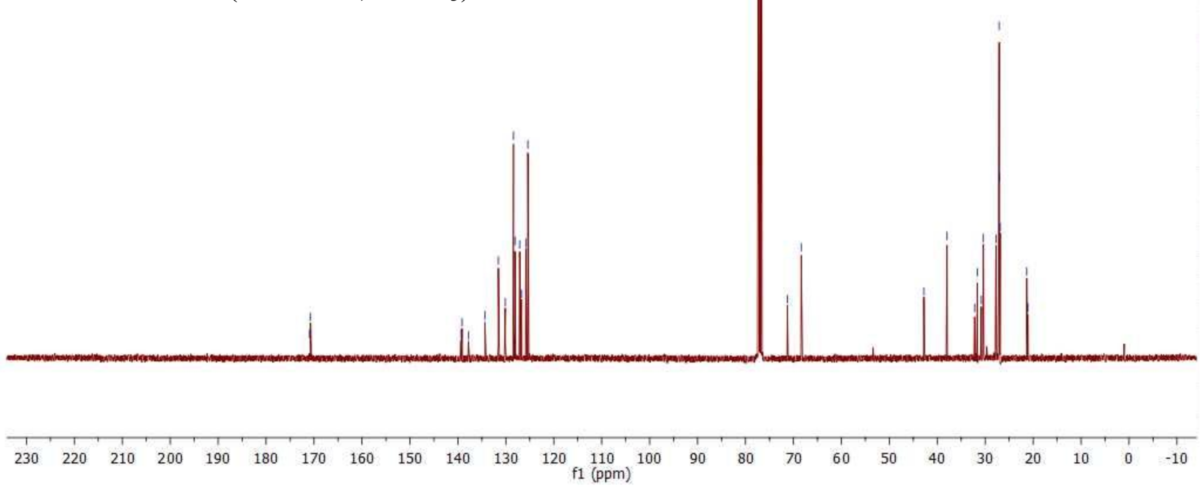

^{13}C NMR (100 MHz, $CDCl_3$)

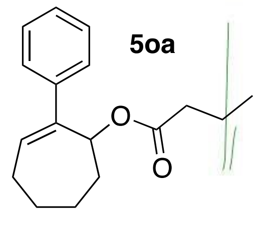

5oa

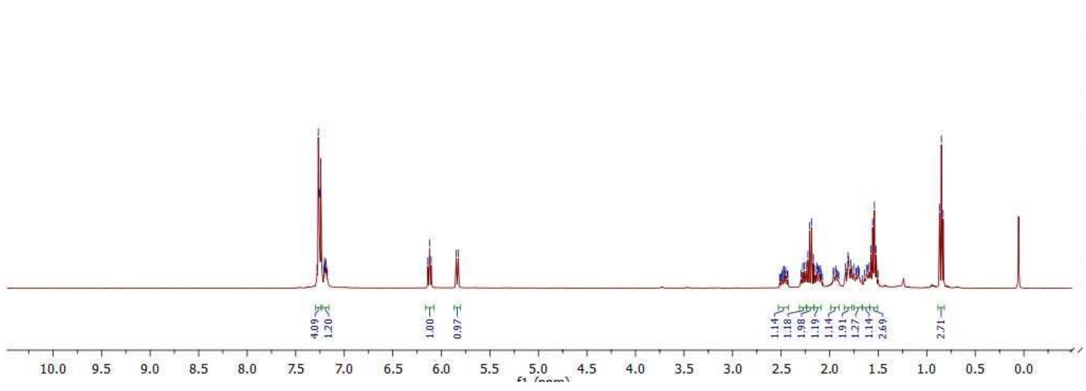

^{1}H NMR (400 MHz, $CDCl_3$)

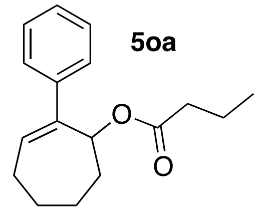

5oa

^{13}C NMR (100 MHz, $CDCl_3$)

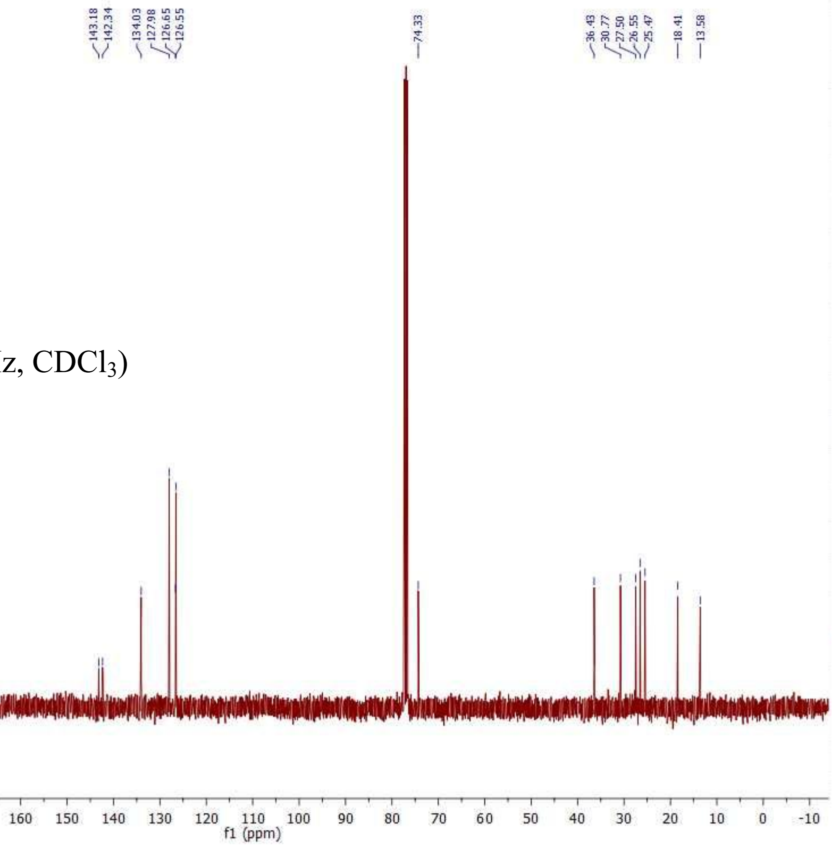

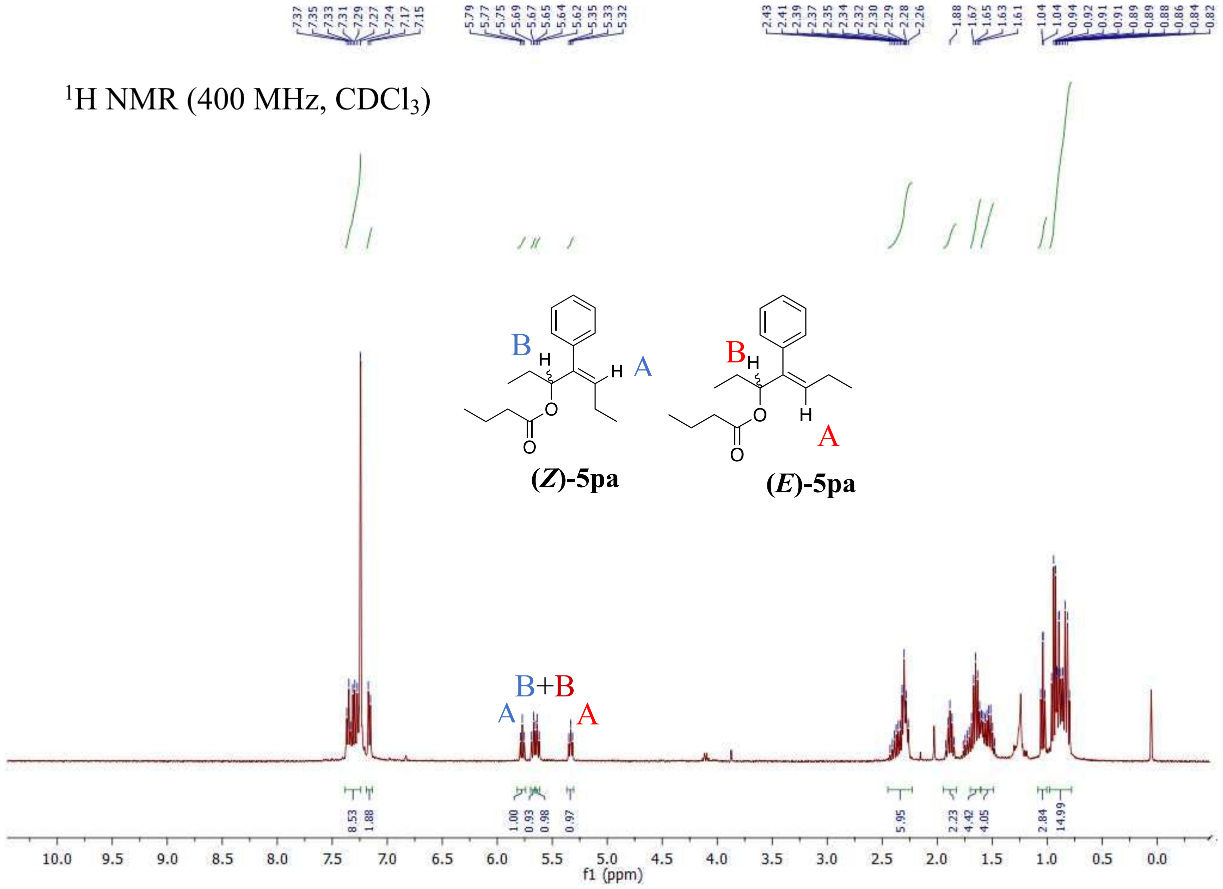

^{1}H NMR (400 MHz, CDCl3)
(Z)-5pa
(E)-5pa
B+B
A
A
f1 (ppm)

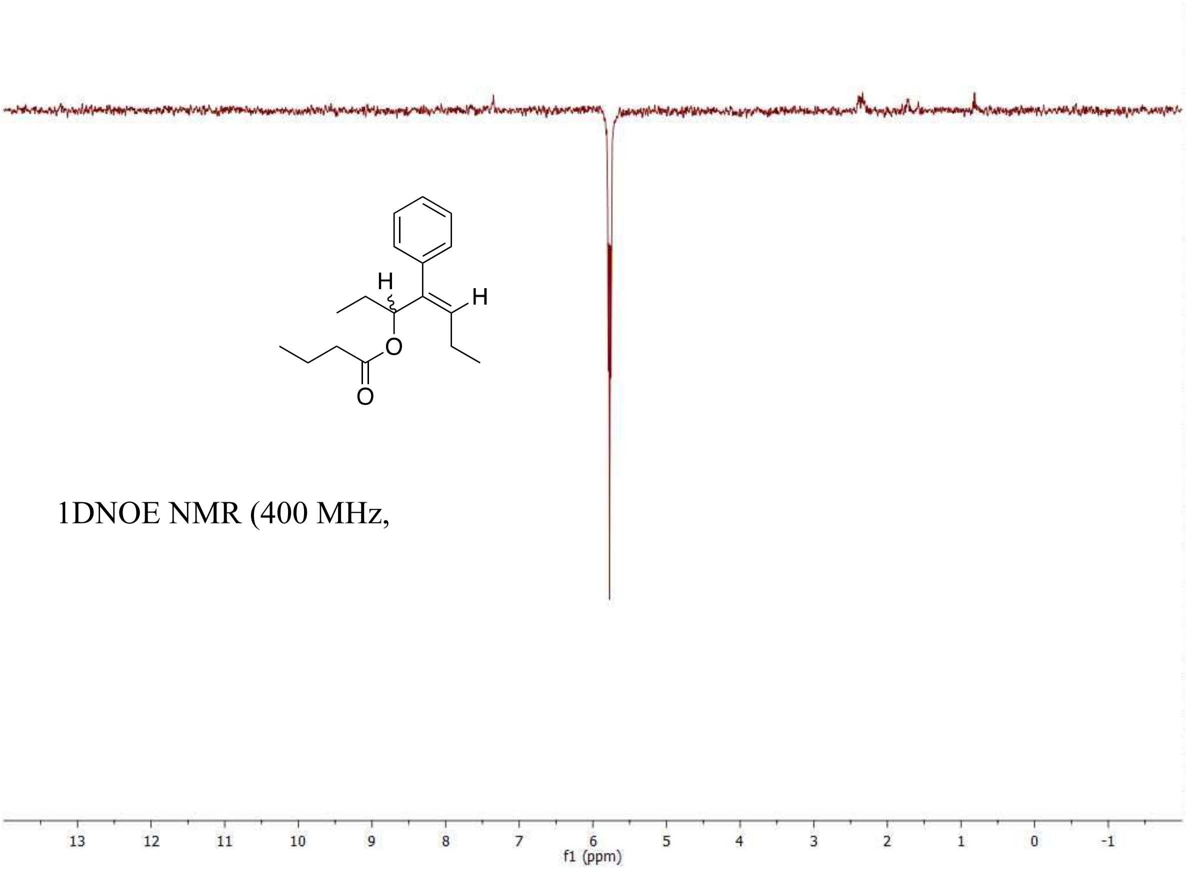
1DNOE NMR (400 MHz,
H
H
O
O
f1 (ppm)

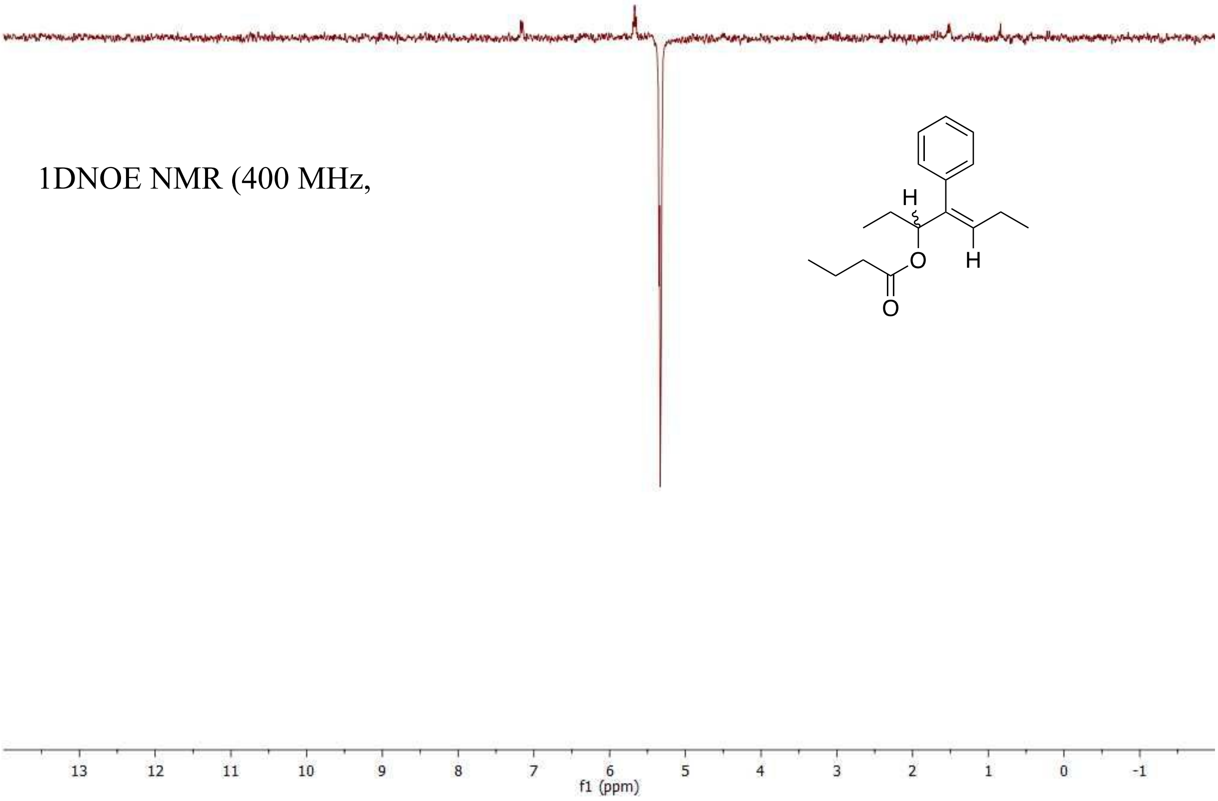
1DNOE NMR (400 MHz,
H
H
O
O
f1 (ppm)

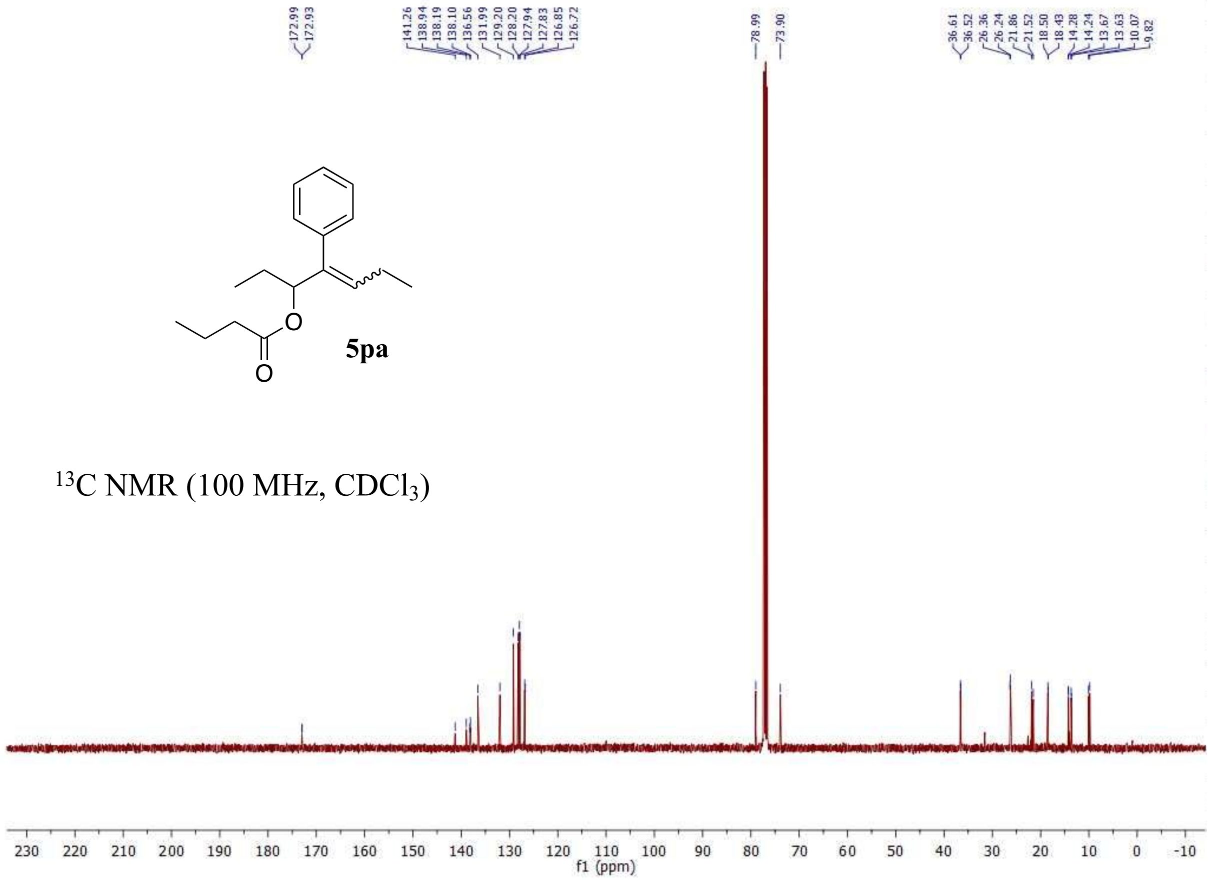

5pa
^{13}C NMR (100 MHz, CDCl$_3$)
f1 (ppm)

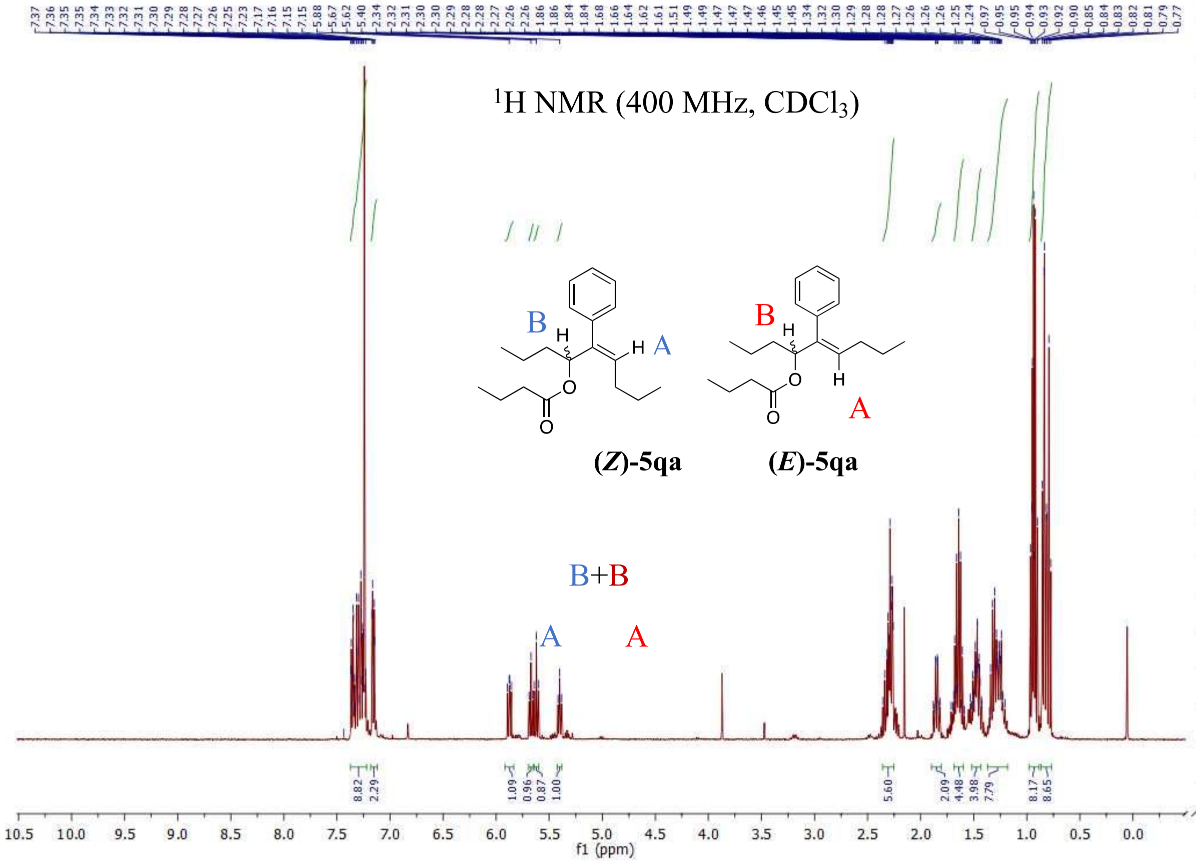

^{1}H NMR (400 MHz, CDCl$_3$)
(Z)-5qa
(E)-5qa
B+B
A
A
f1 (ppm)

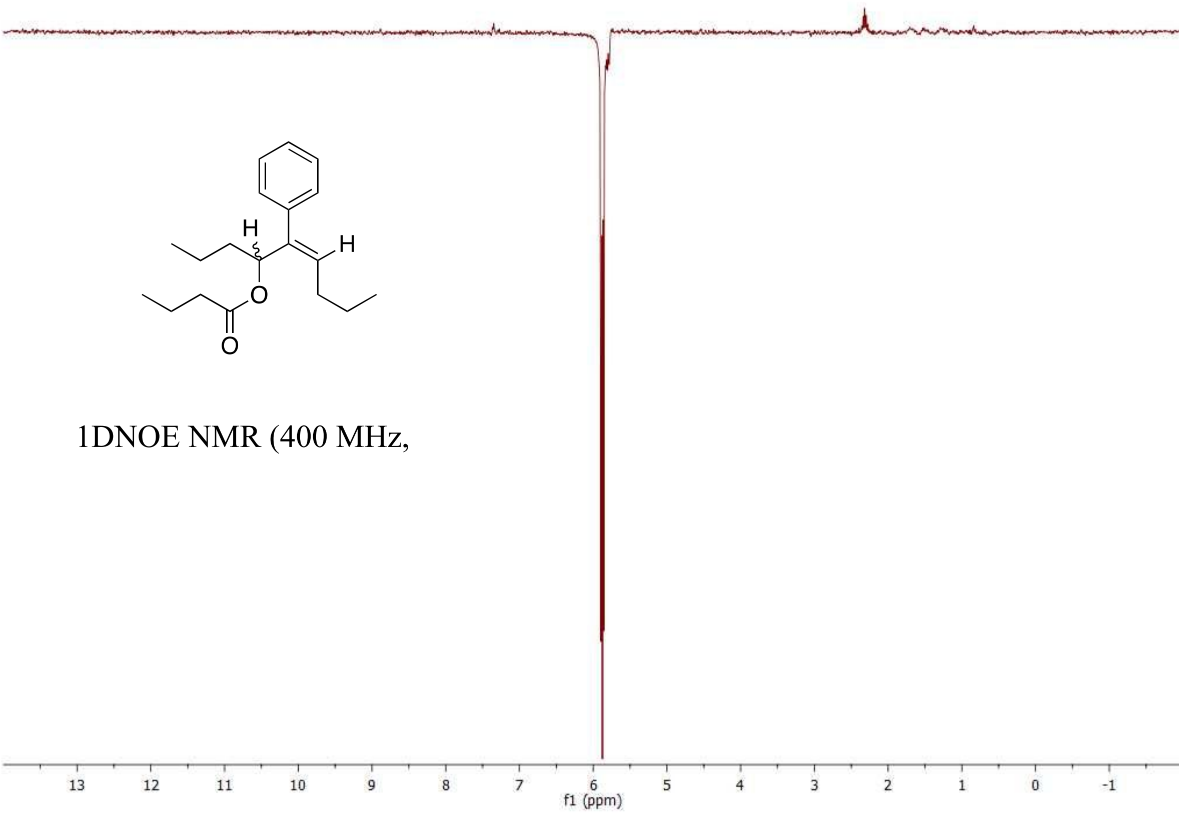

1DNOE NMR (400 MHz,

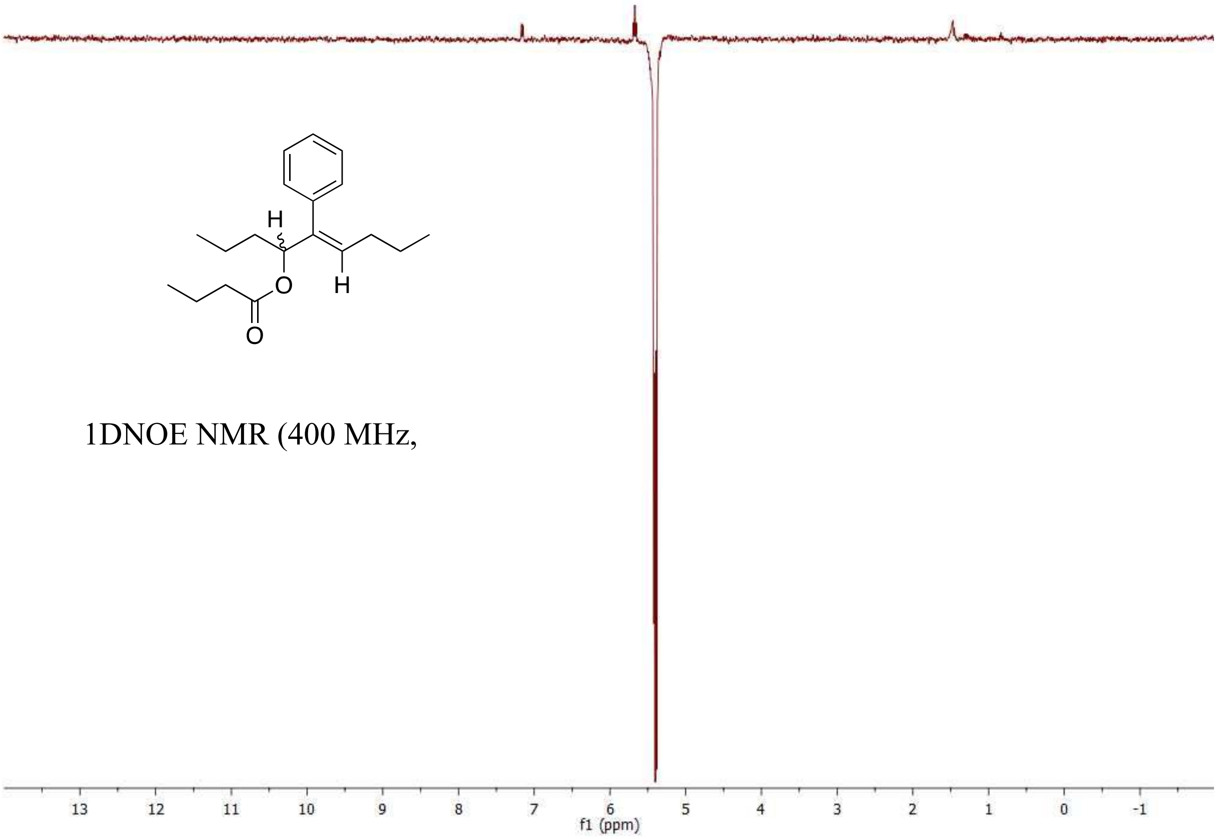
H
O
H
O
1DNOE NMR (400 MHz,
13 12 11 10 9 8 7 6 5 4 3 2 1 0 -1
f1 (ppm)

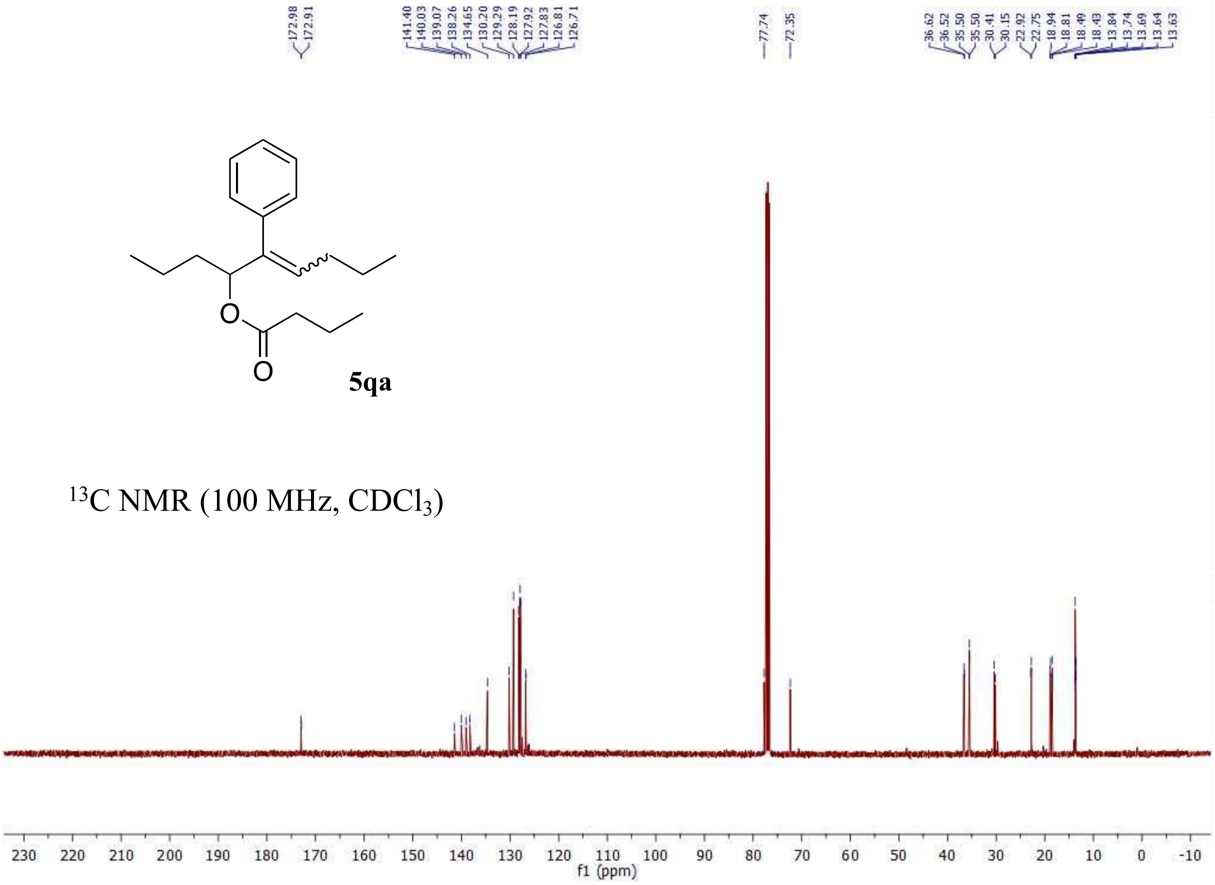
5qa
13C NMR (100 MHz, CDCl3)
f1 (ppm)

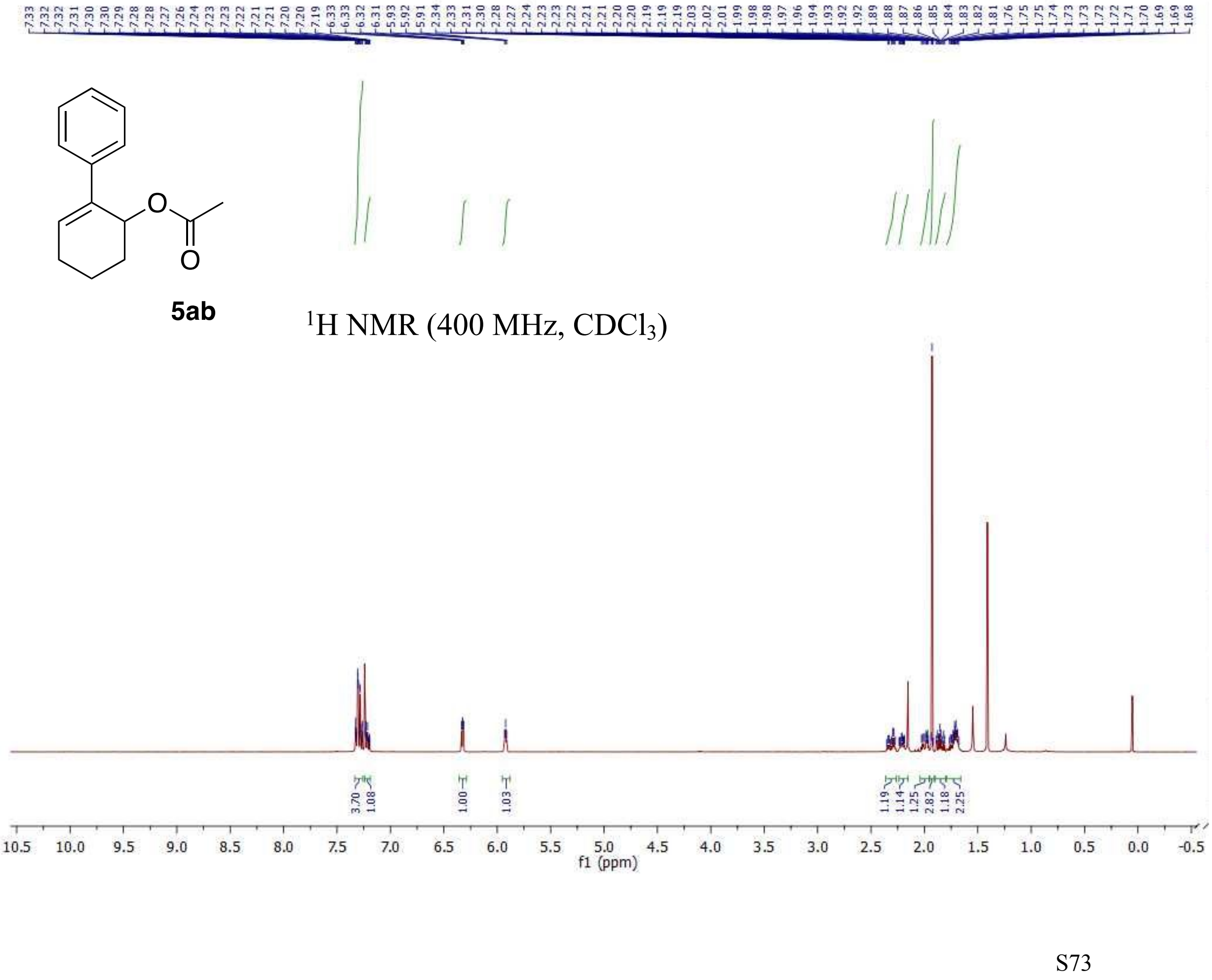
5ab
1H NMR (400 MHz, CDCl3)
f1 (ppm)

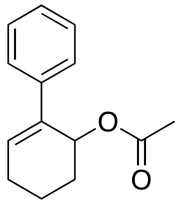

5ab

^{13}C NMR (100 MHz, $CDCl_3$)

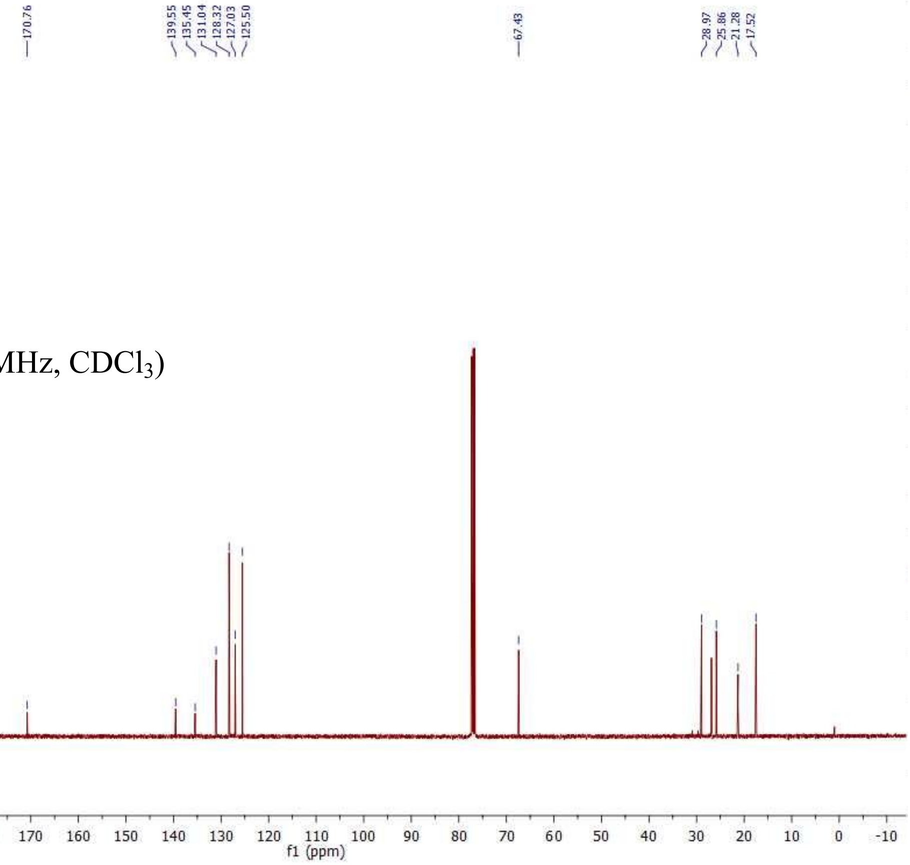

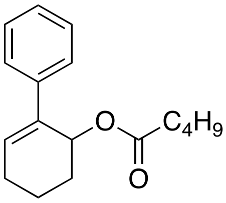

5ac

^{1}H NMR (400 MHz, $CDCl_3$)

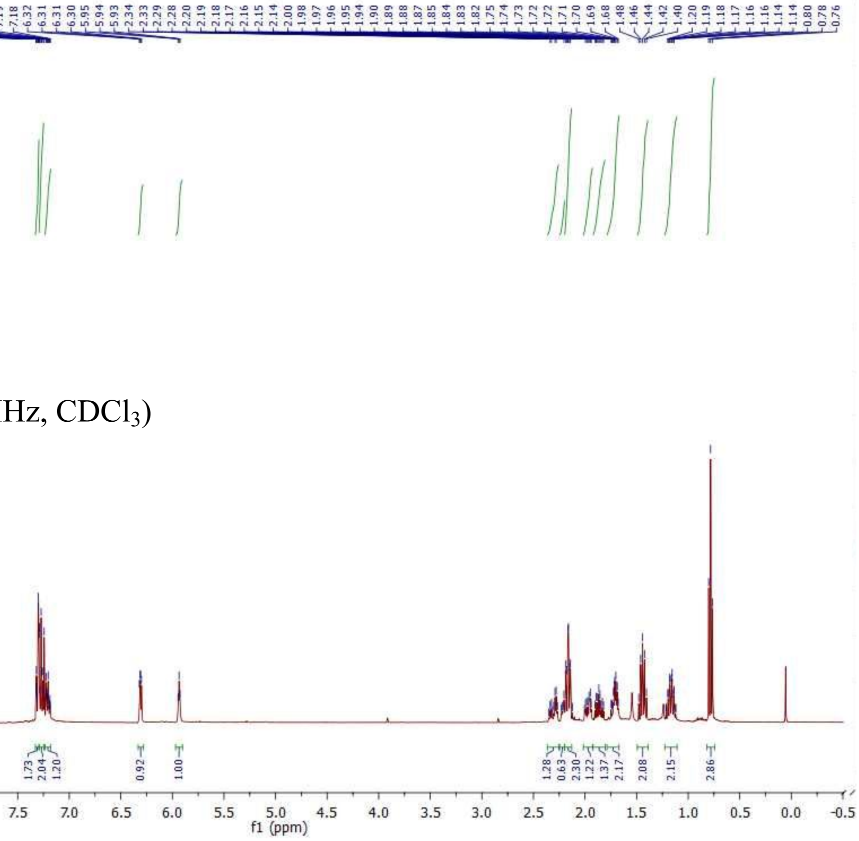

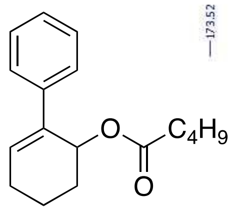

5ac

^{13}C NMR (100 MHz, $CDCl_3$)

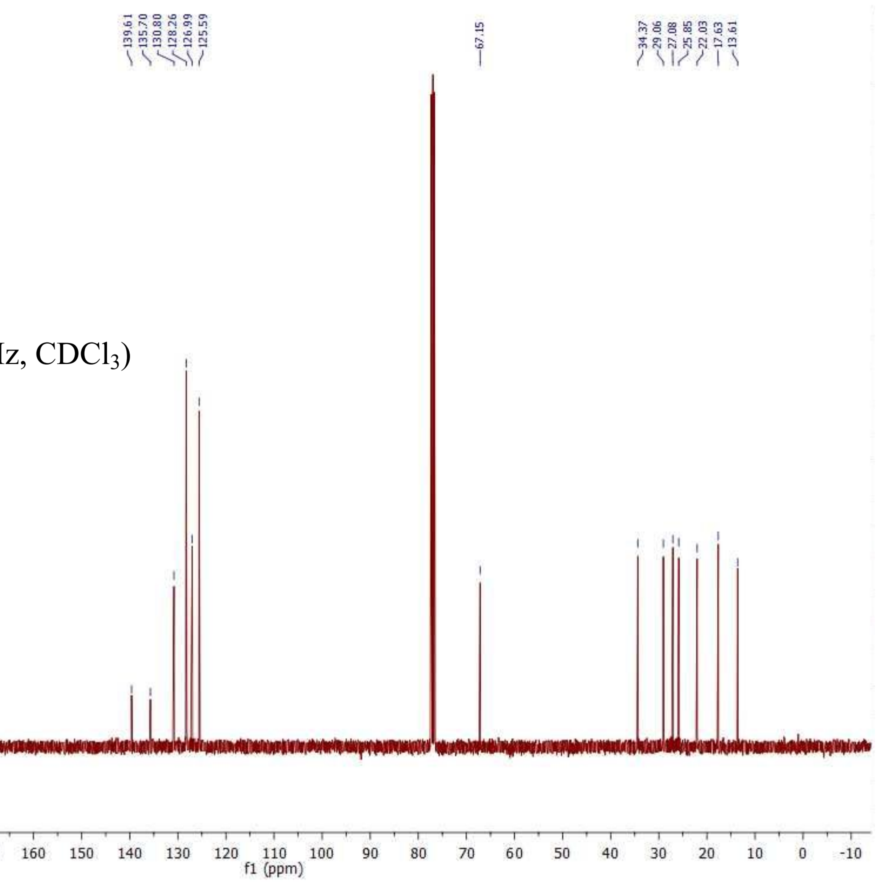

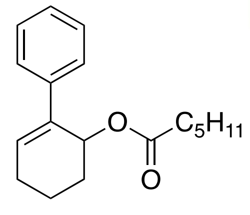

5ad

^{1}H NMR (400 MHz, $CDCl_3$)

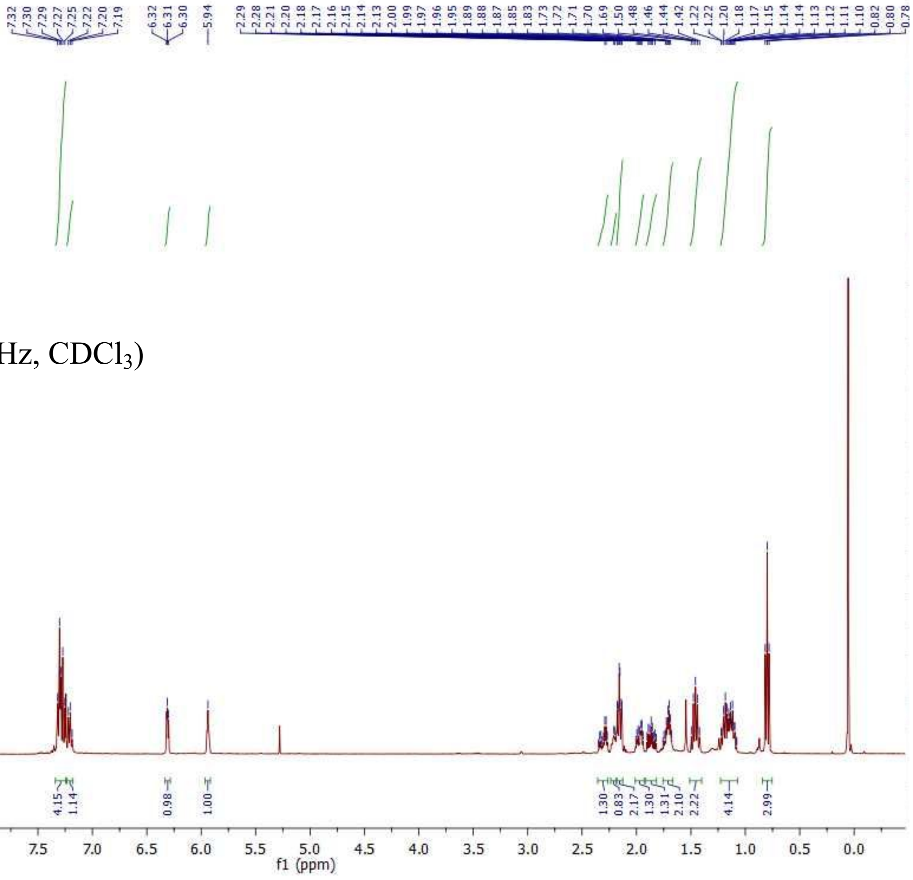

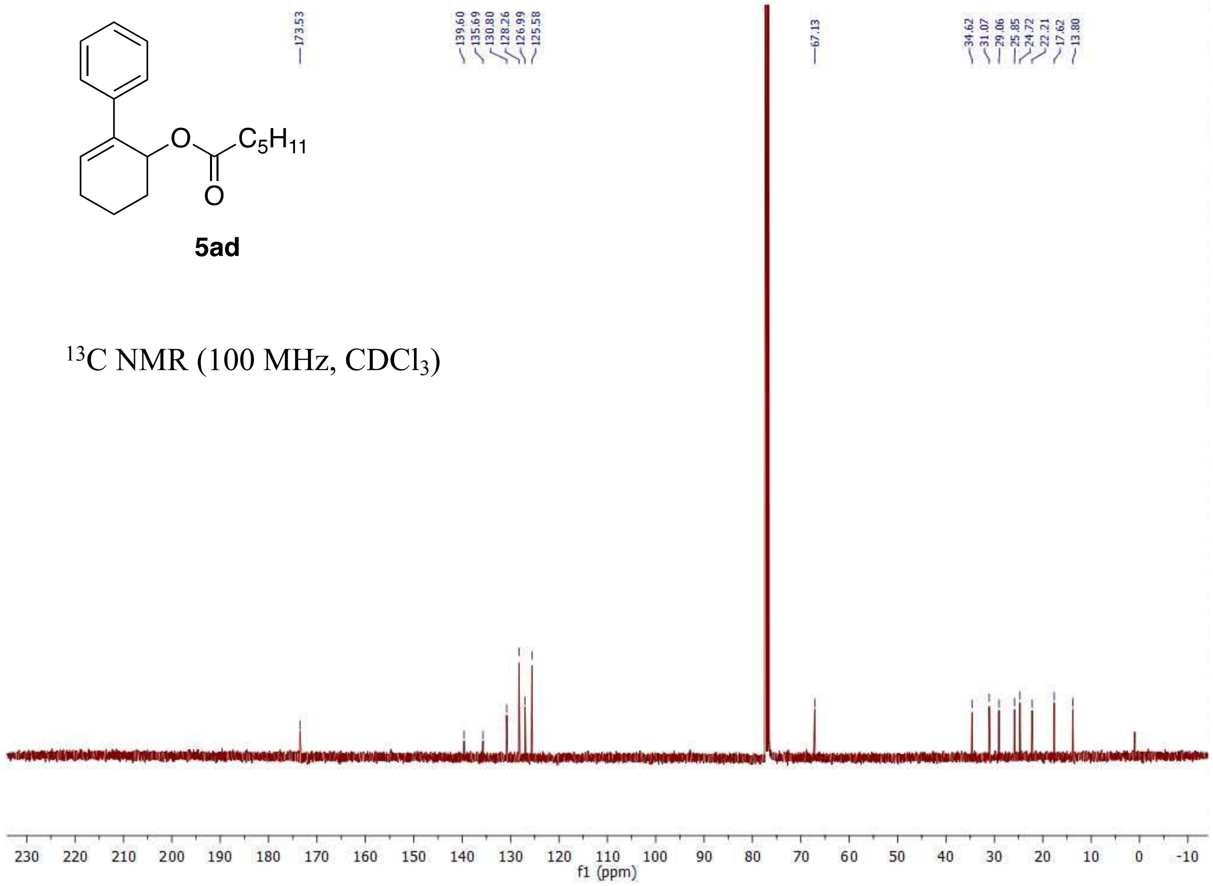

5ad

^{13}C NMR (100 MHz, $CDCl_3$)

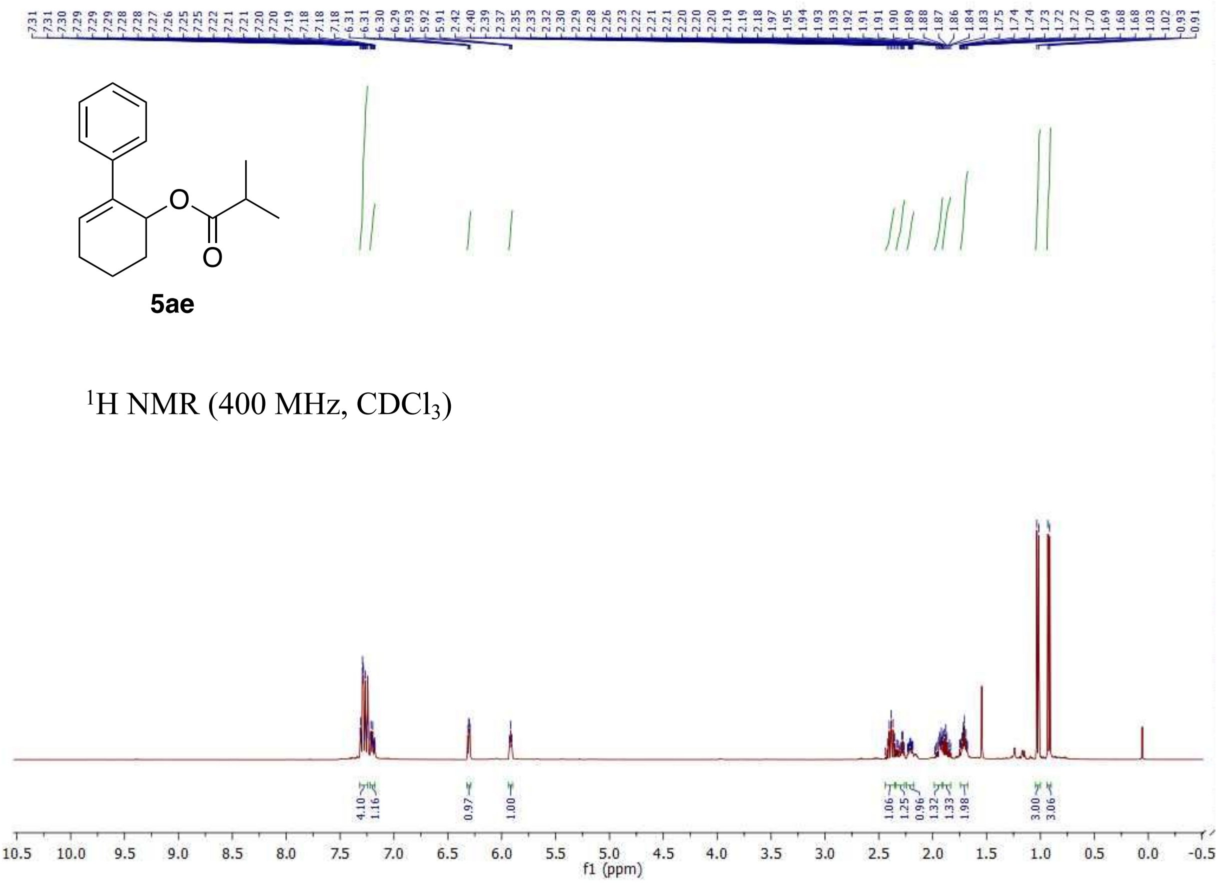

5ae

^{1}H NMR (400 MHz, $CDCl_3$)

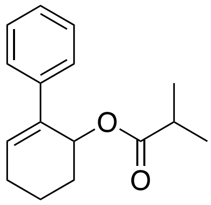

5ae

^{13}C NMR (100 MHz, $CDCl_3$)

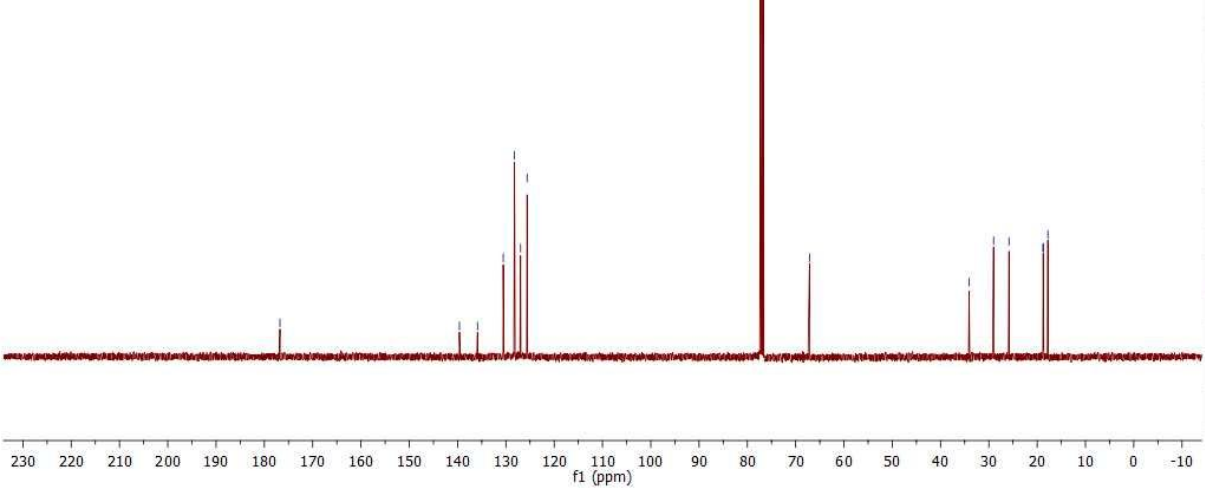

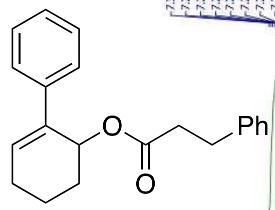

5af

^{1}H NMR (400 MHz, $CDCl_3$)

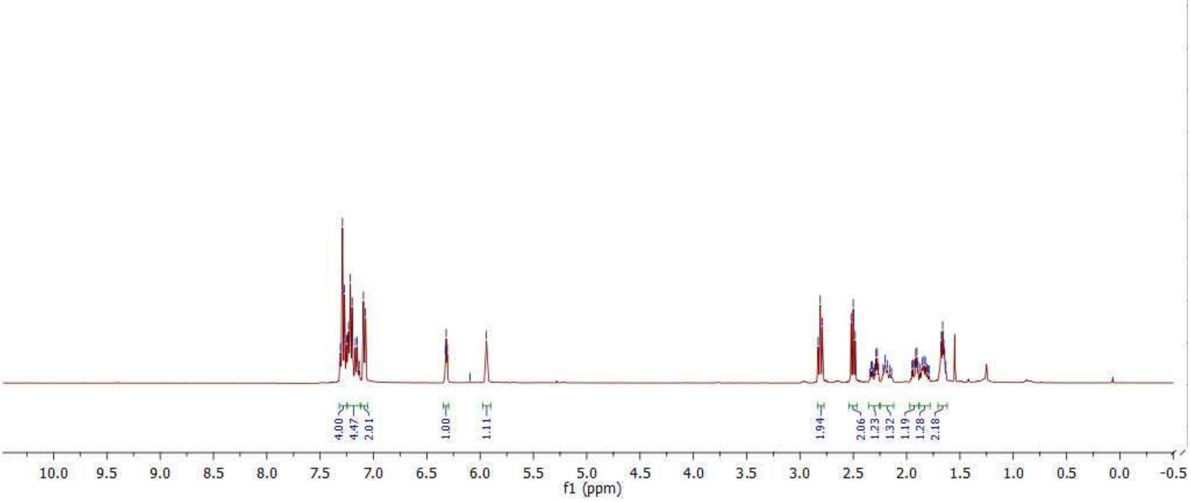

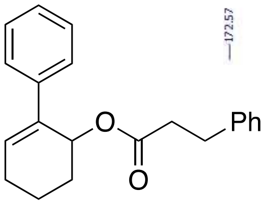

5af

^{13}C NMR (100 MHz, $CDCl_3$)

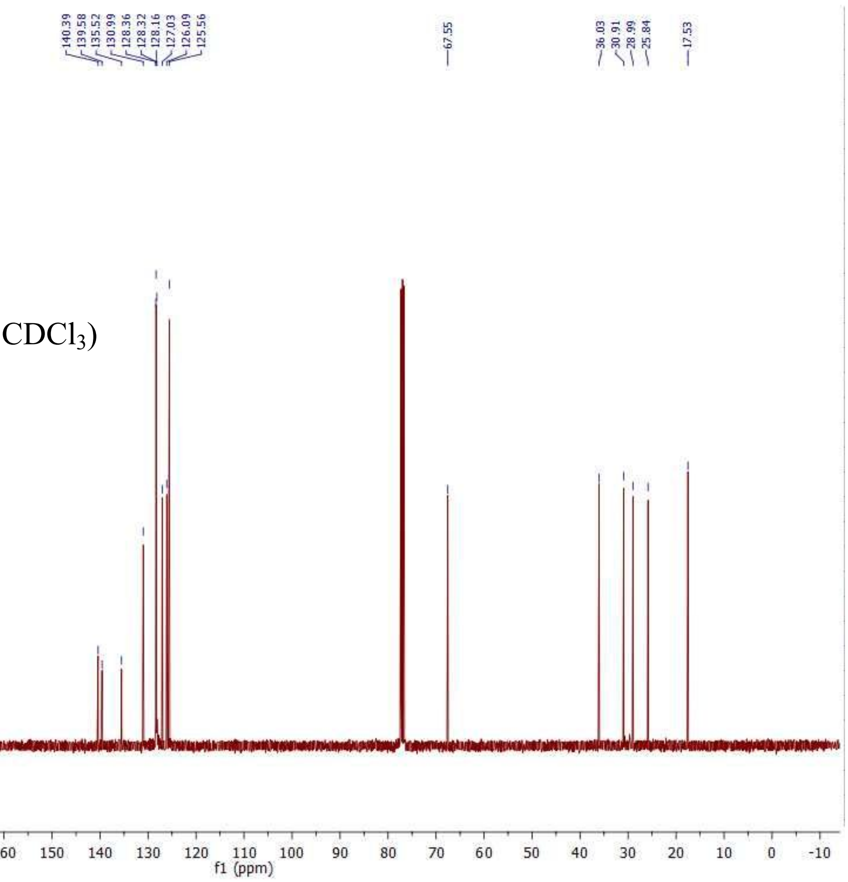

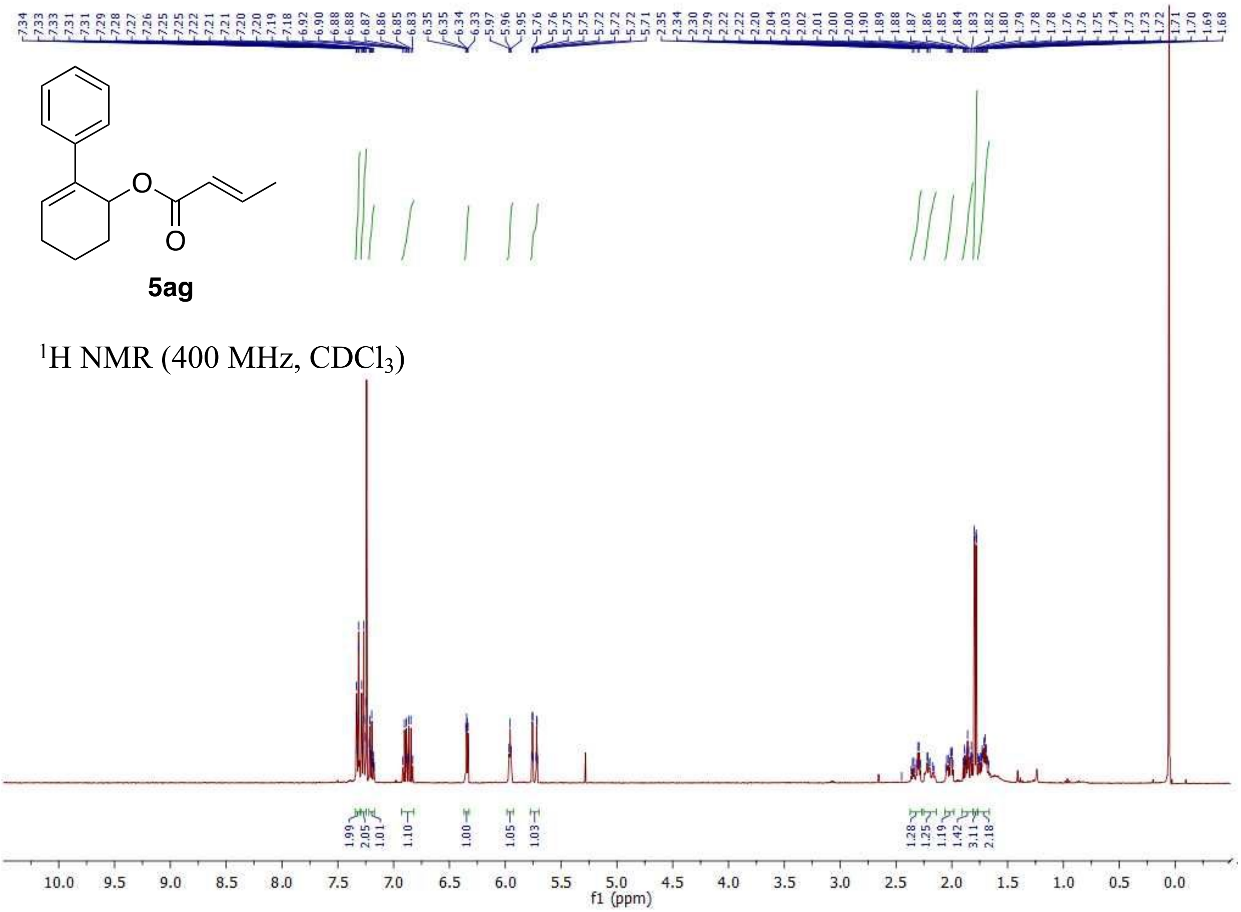

5ag

^{1}H NMR (400 MHz, $CDCl_3$)

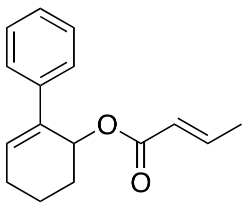

5ag

^{13}C NMR (100 MHz, $CDCl_3$)

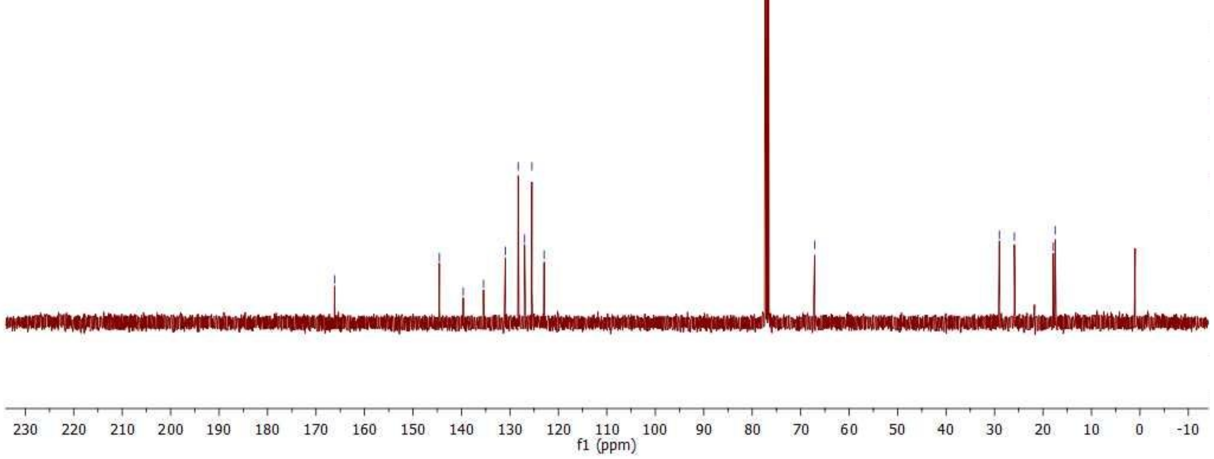

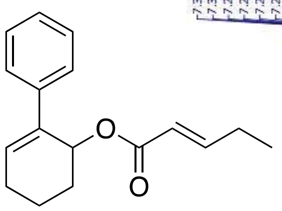

5ah

^{1}H NMR (400 MHz, $CDCl_3$)

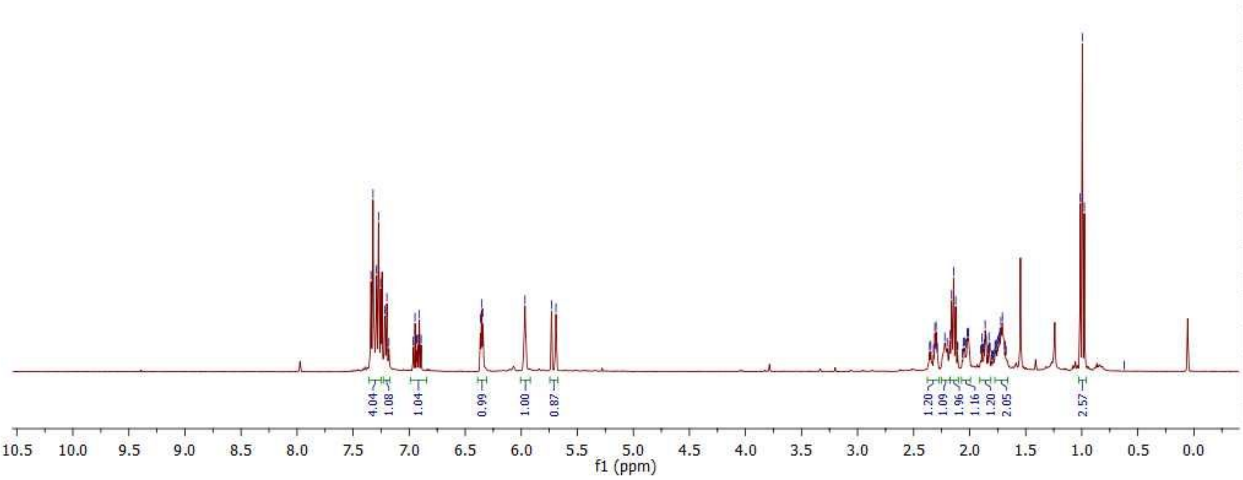

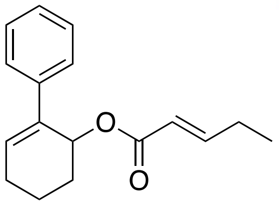

5ah

^{13}C NMR (100 MHz, $CDCl_3$)

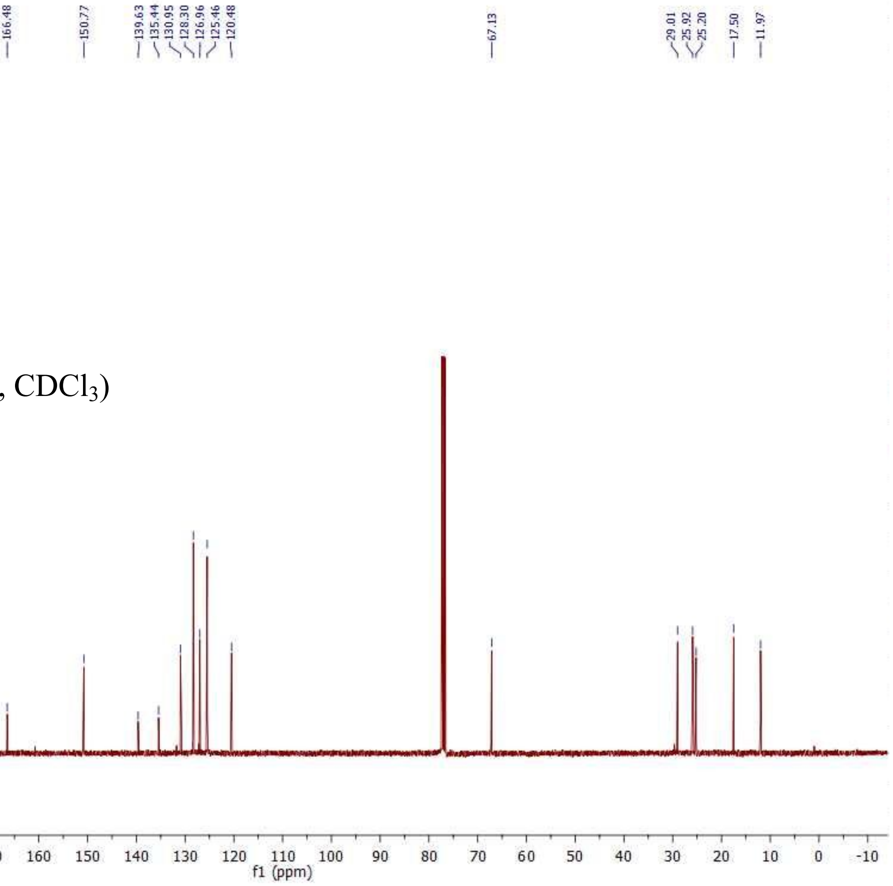

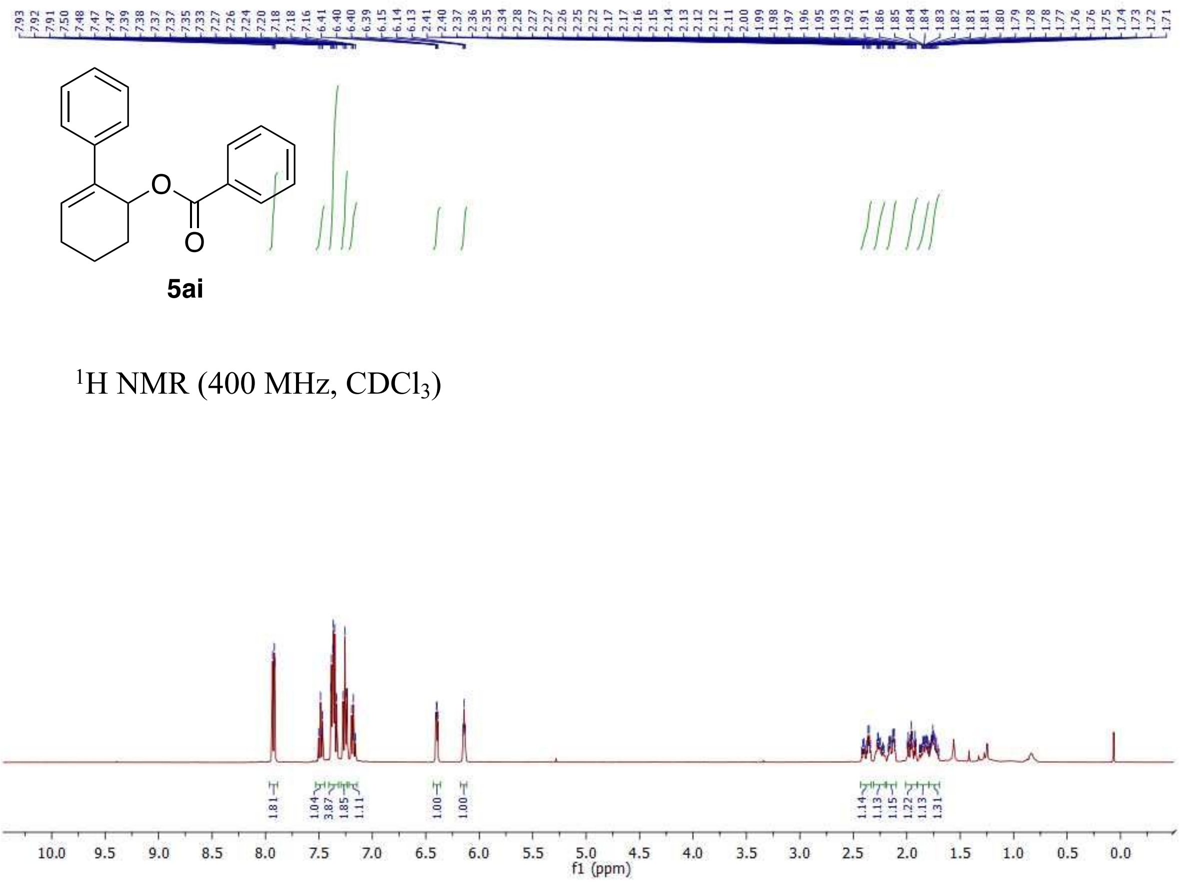

5ai

^{1}H NMR (400 MHz, $CDCl_3$)

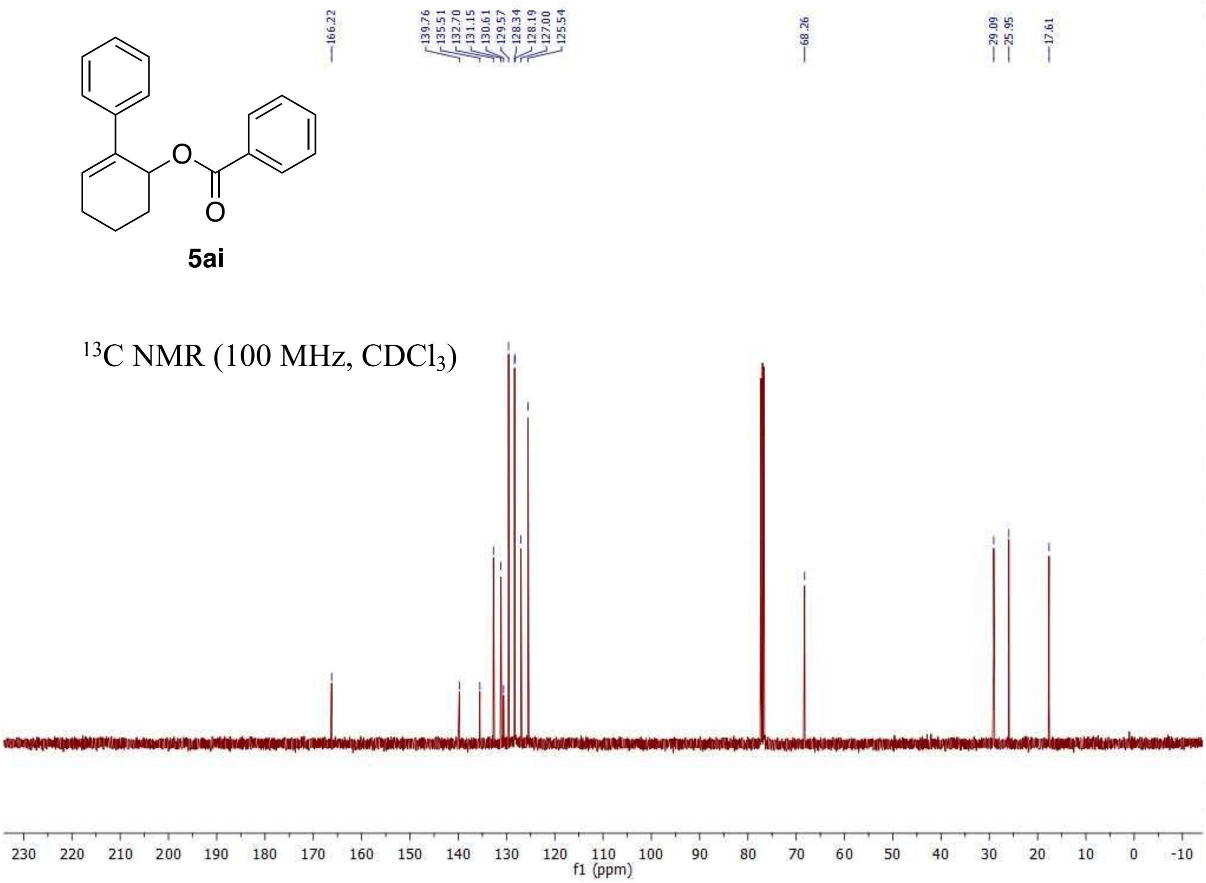

5ai

^{13}C NMR (100 MHz, $CDCl_3$)

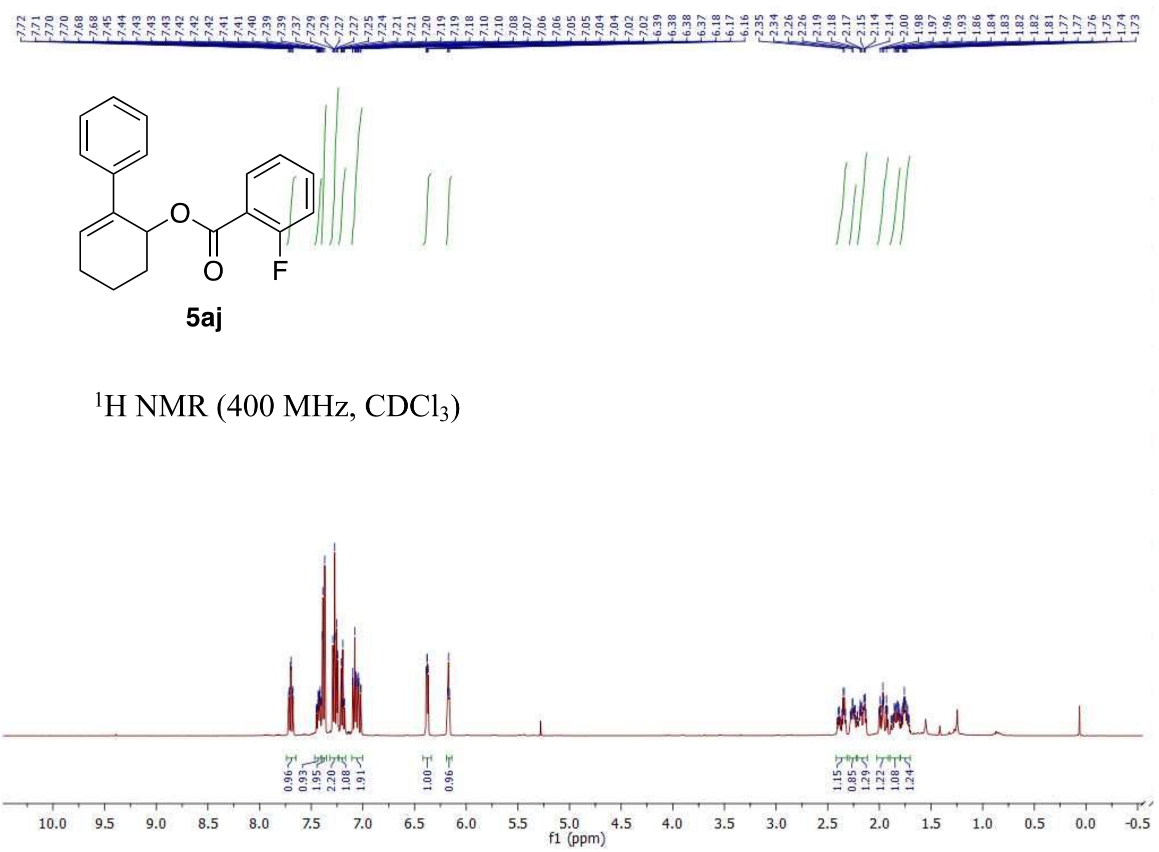

5aj

^{1}H NMR (400 MHz, $CDCl_3$)

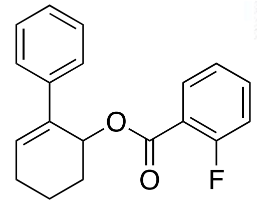

5aj

^{13}C NMR (100 MHz, $CDCl_3$)

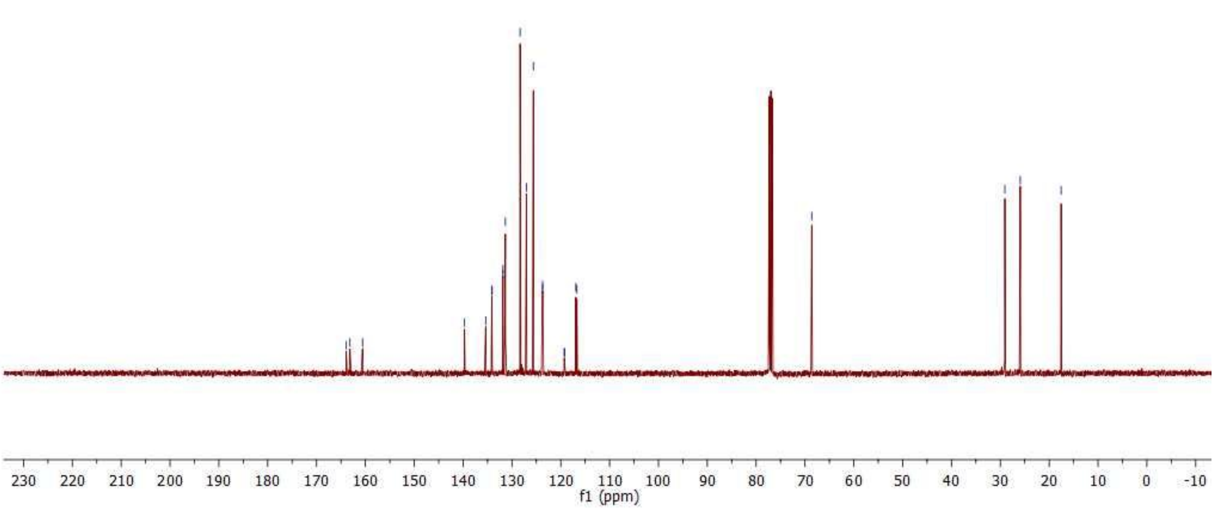

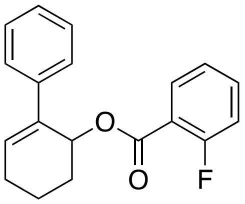

5aj

^{19}F NMR (377 MHz, $CDCl_3$)

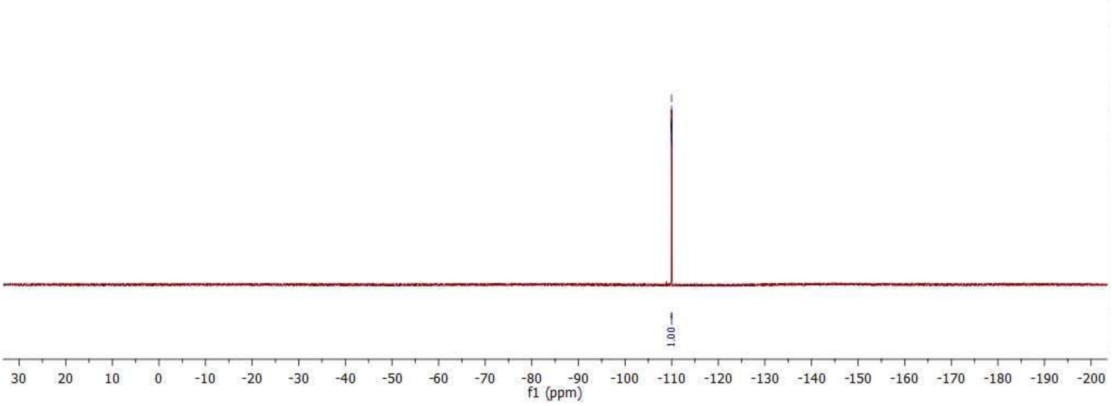

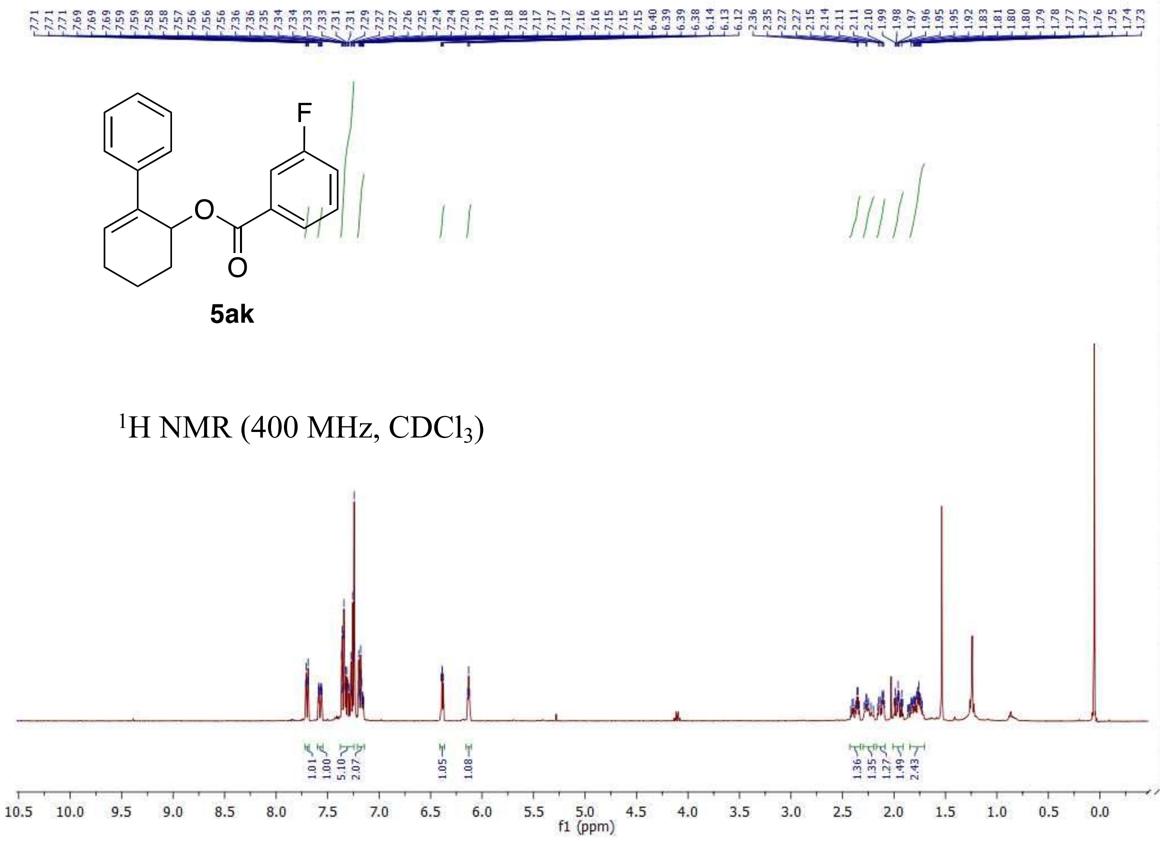
5ak
^{1}H NMR (400 MHz, CDCl$_3$)
f1 (ppm)
10.5 10.0 9.5 9.0 8.5 8.0 7.5 7.0 6.5 6.0 5.5 5.0 4.5 4.0 3.5 3.0 2.5 2.0 1.5 1.0 0.5 0.0

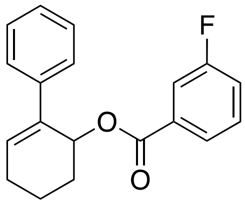

5ak

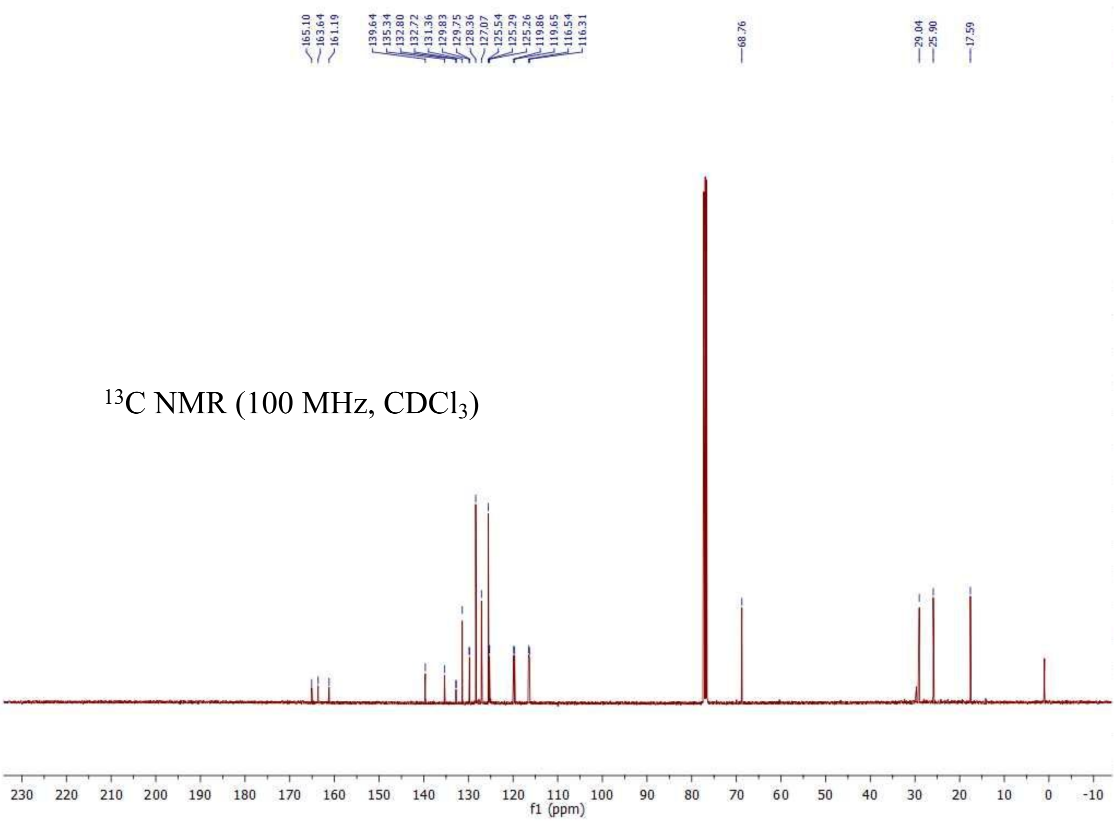

^{13}C NMR (100 MHz, $CDCl_3$)

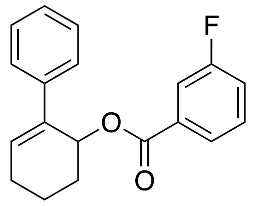

5ak

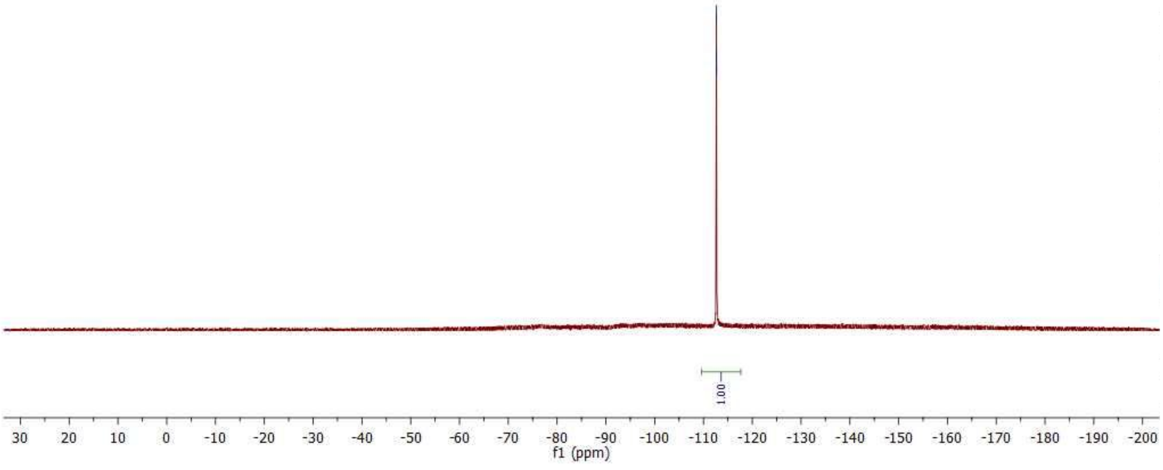

^{19}F NMR (377 MHz, $CDCl_3$)

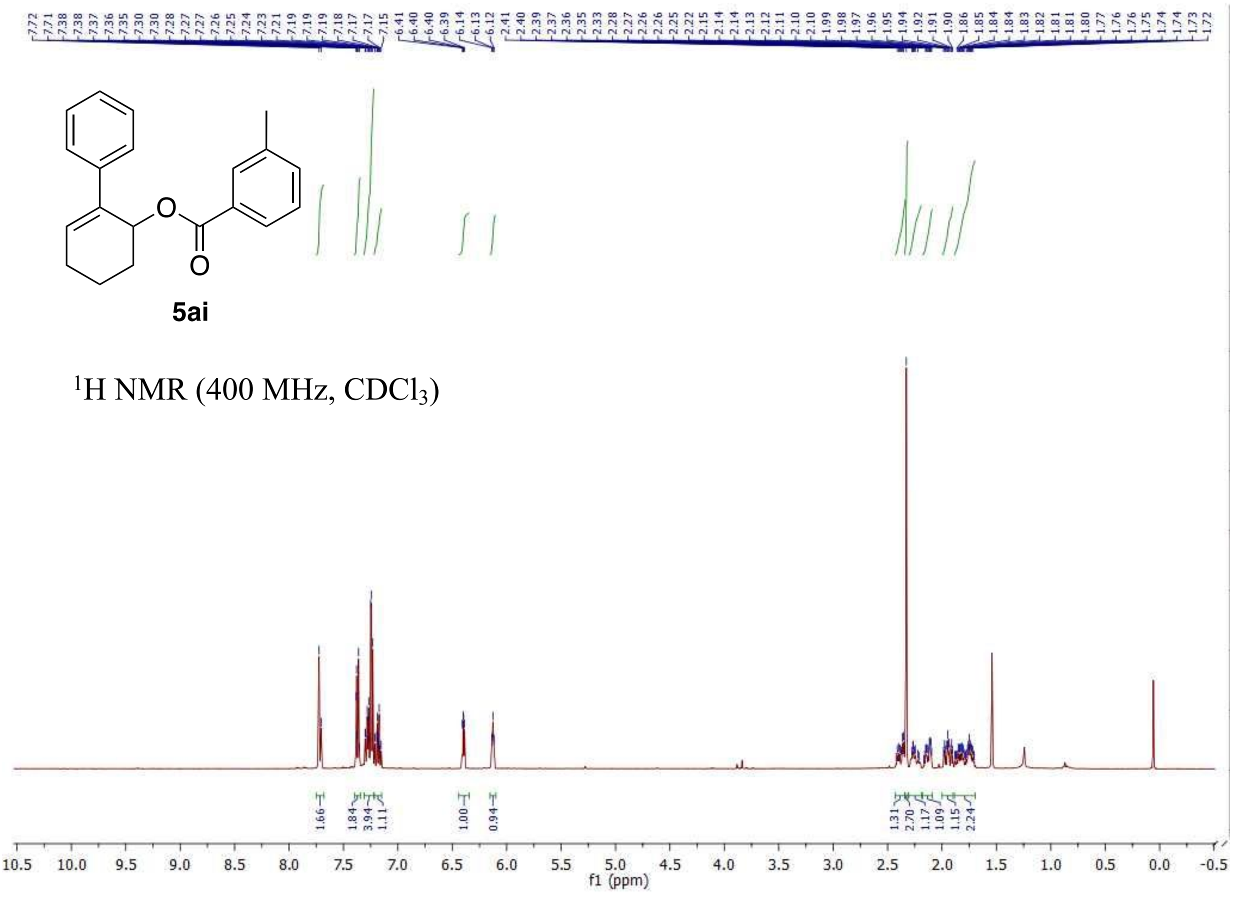
5ai
1H NMR (400 MHz, CDCl3)
f1 (ppm)

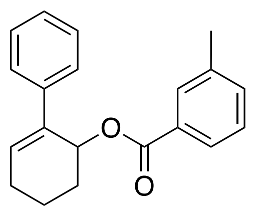

5ai

^{13}C NMR (100 MHz, CDCl$_3$)

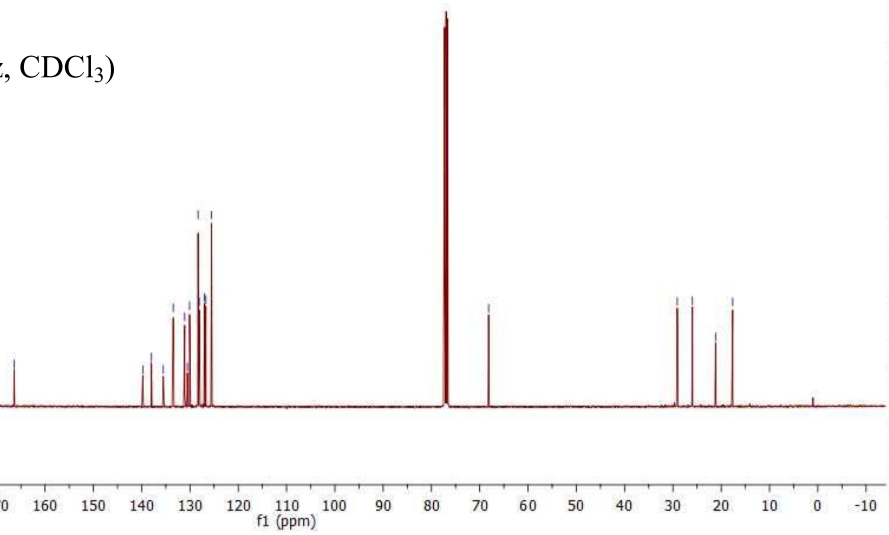

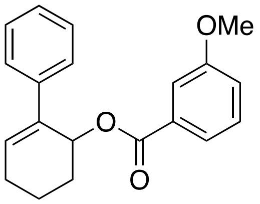

5am

^{1}H NMR (400 MHz, $CDCl_3$)

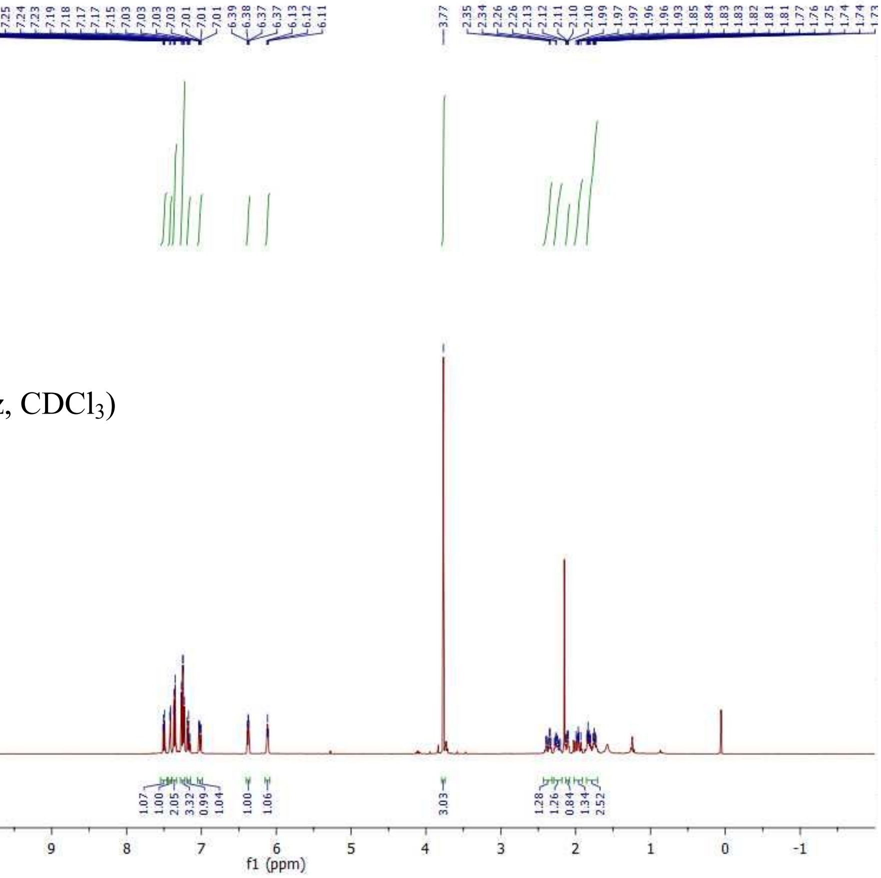

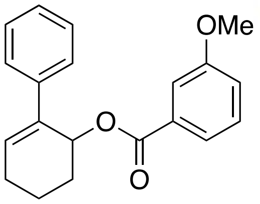

5am

^{13}C NMR (100 MHz, $CDCl_3$)

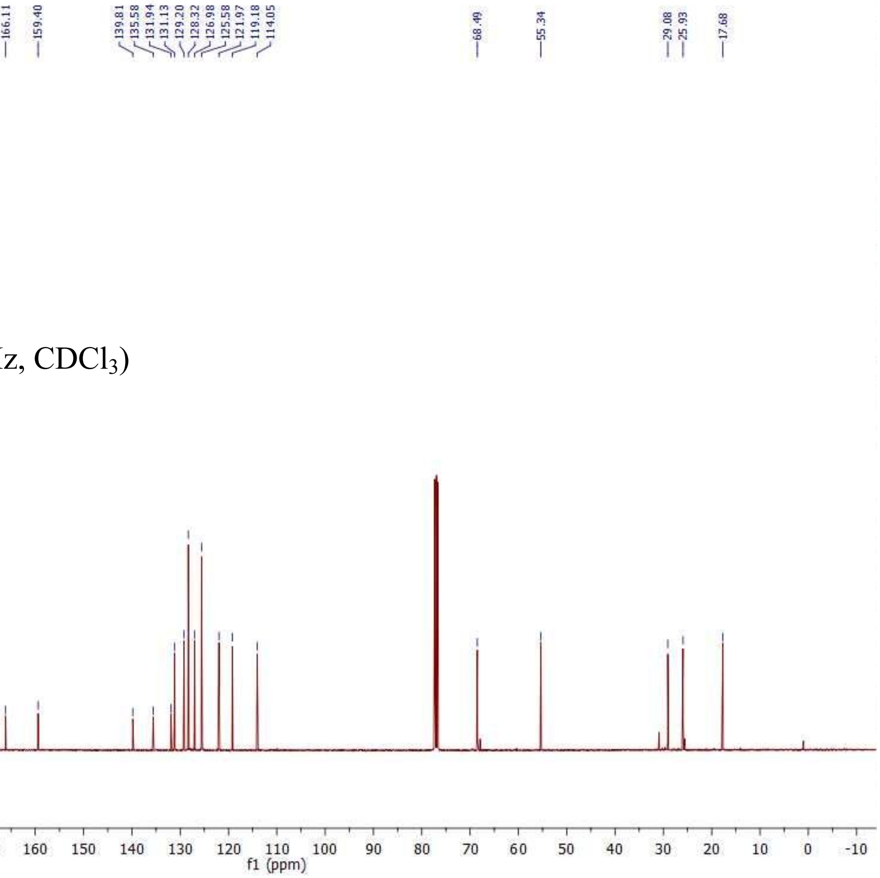

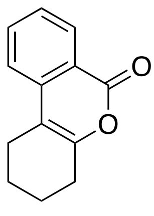

7a

^{1}H NMR (400 MHz, $CDCl_3$)

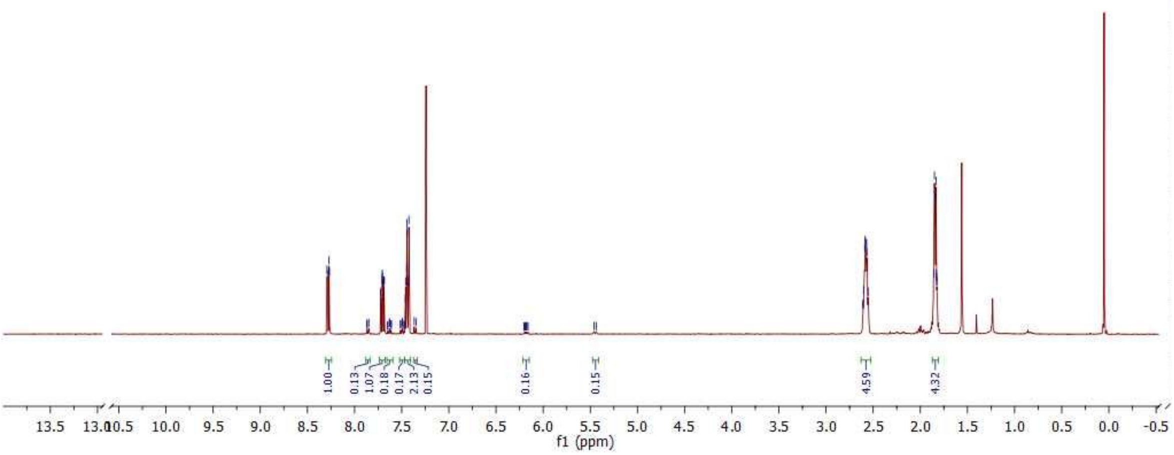

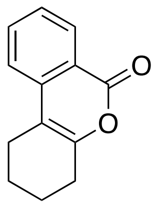

7a

^{13}C NMR (100 MHz, $CDCl_3$)

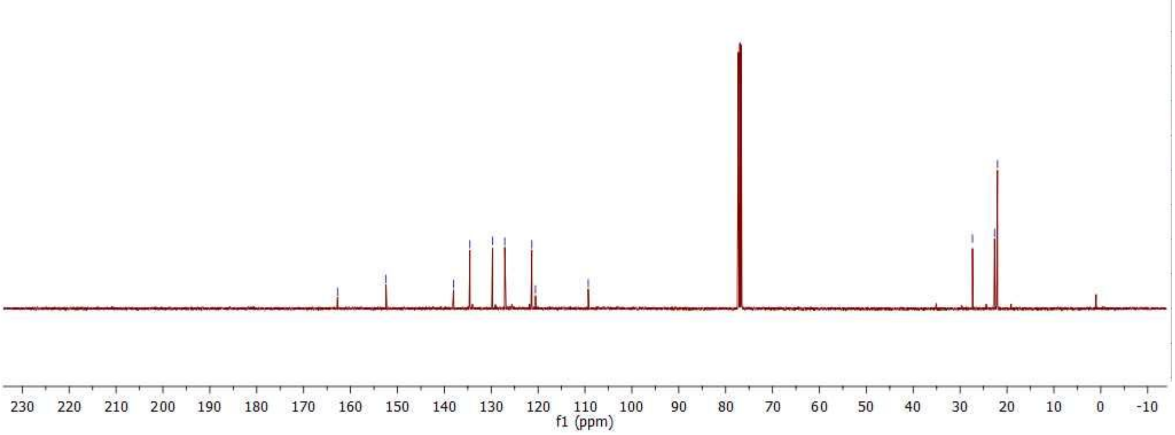

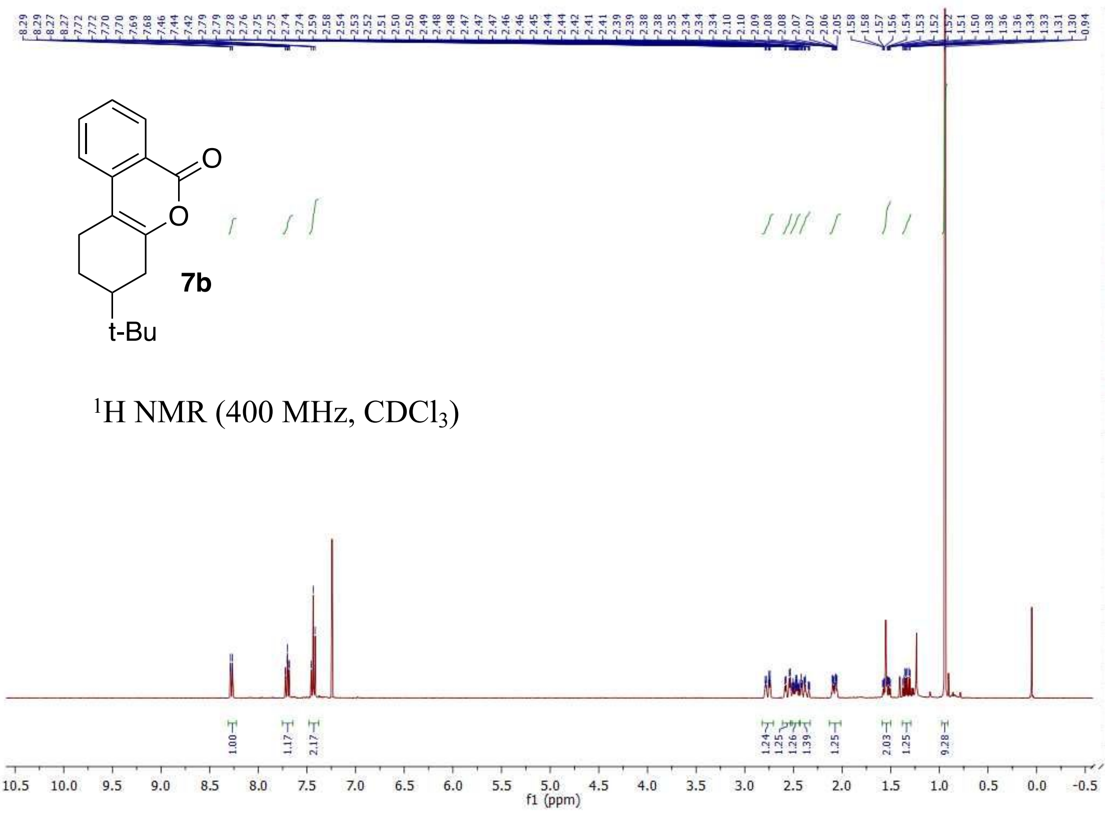

^{1}H NMR (400 MHz, $CDCl_3$)

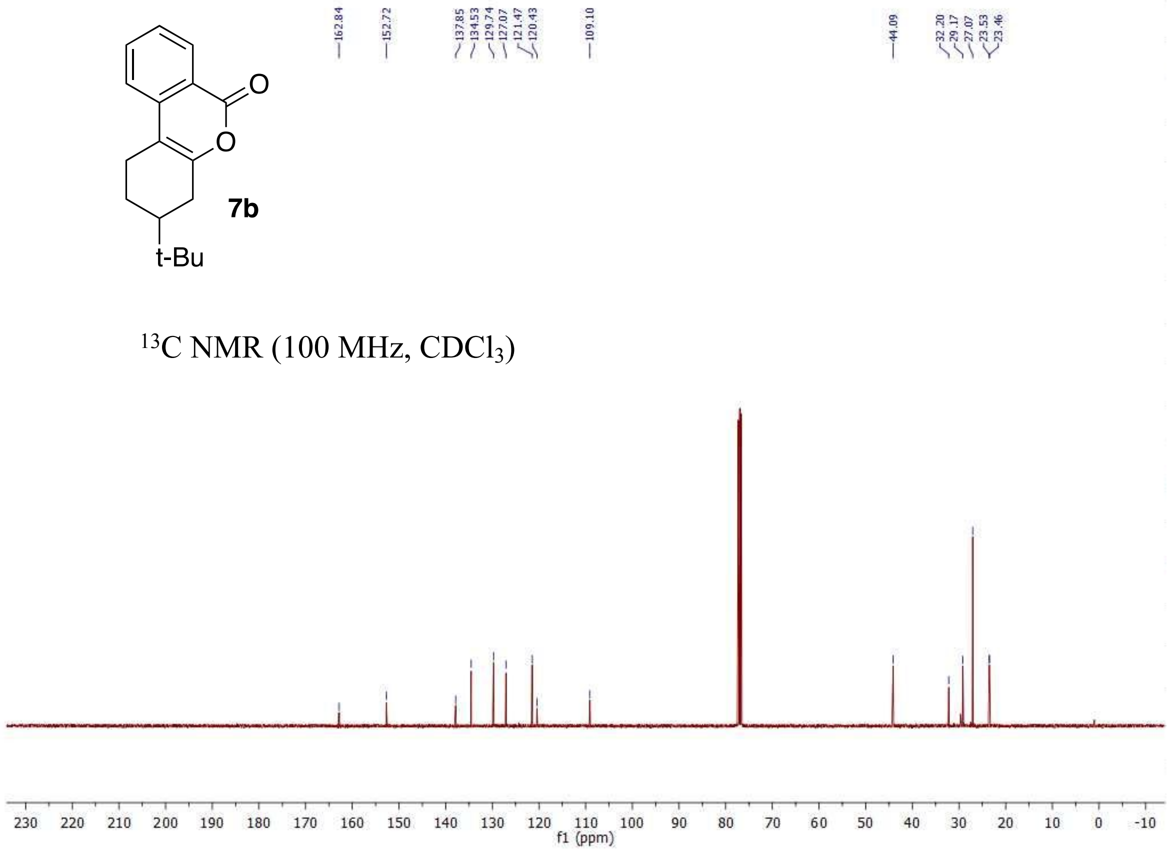

^{13}C NMR (100 MHz, $CDCl_3$)

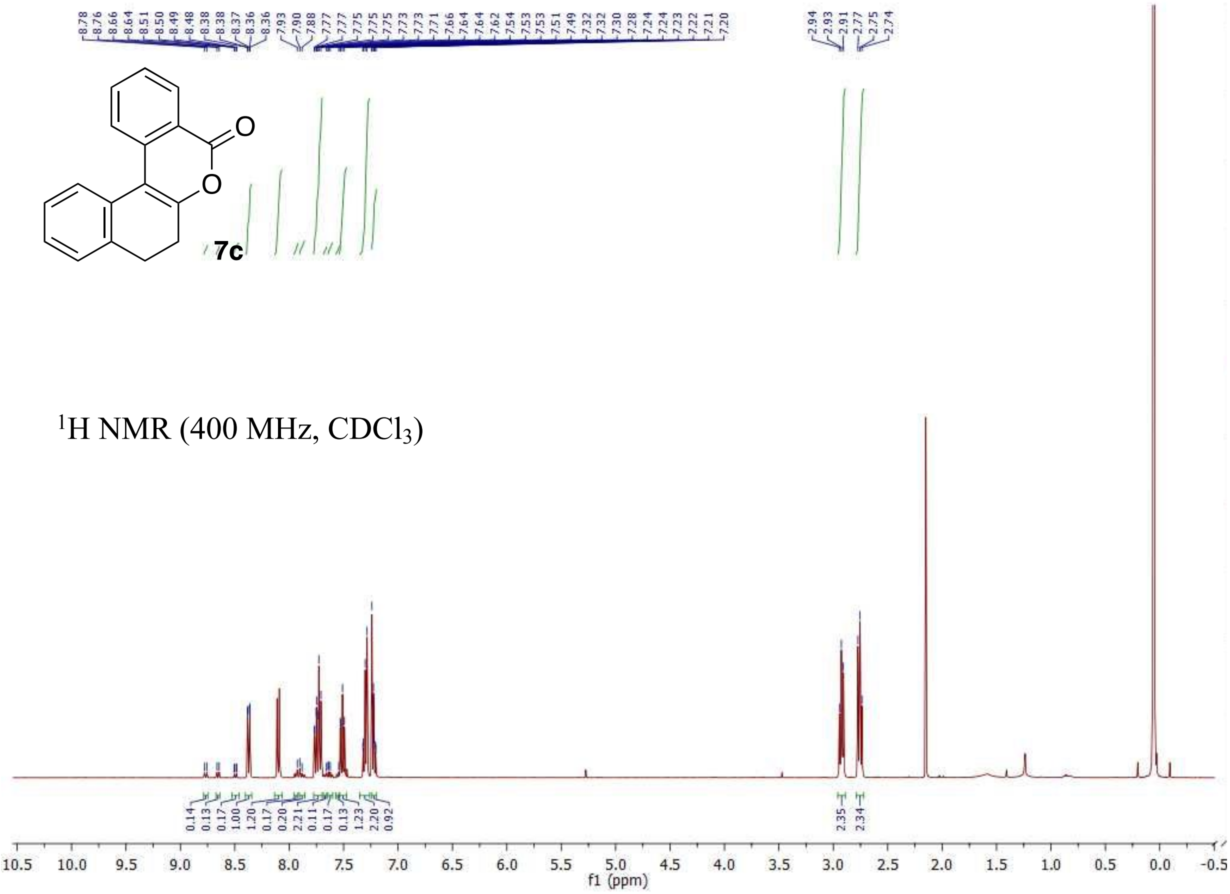

^{1}H NMR (400 MHz, $CDCl_3$)

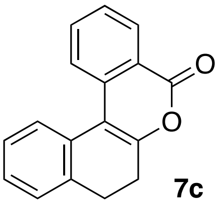

7c

^{13}C NMR (100 MHz, $CDCl_3$)

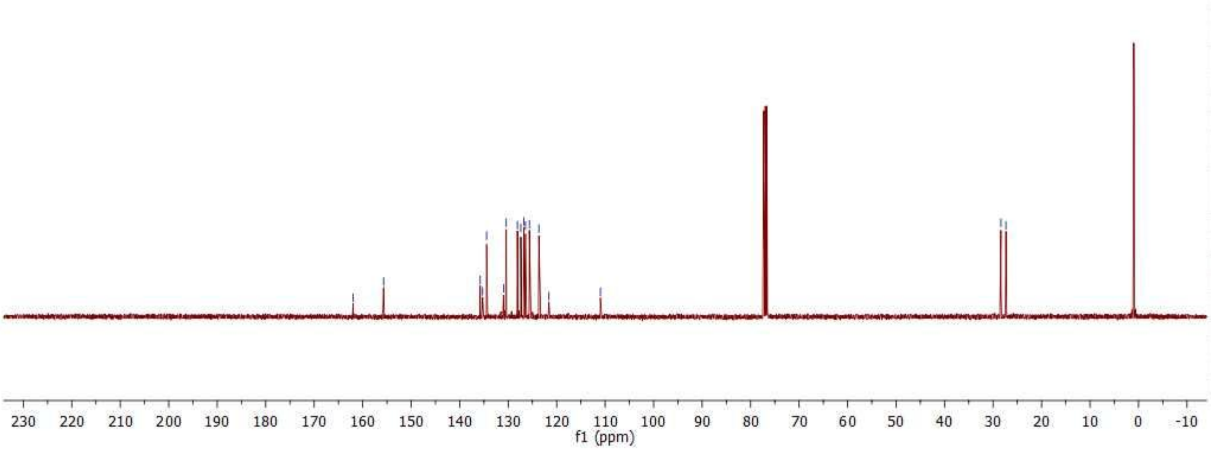

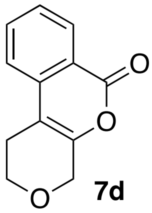

7d

^{1}H NMR (400 MHz, $CDCl_3$)

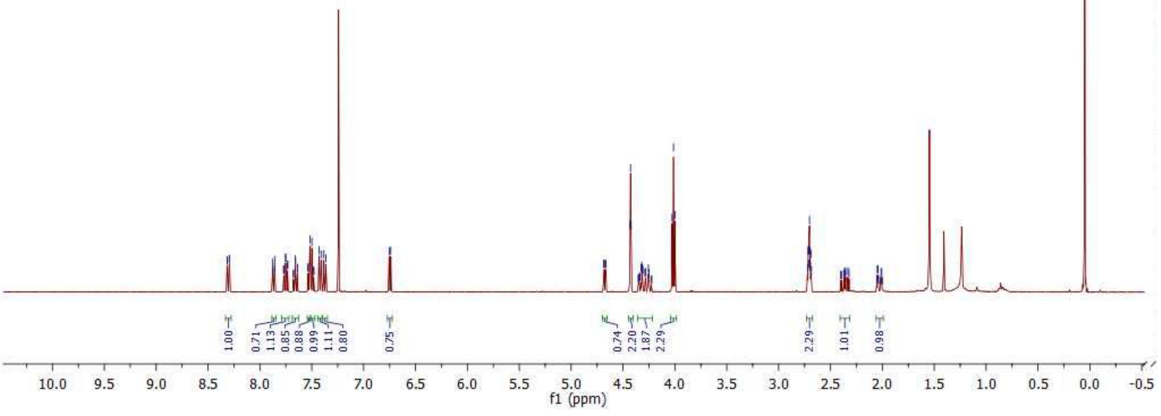

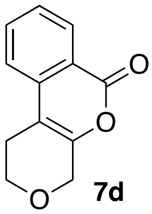

7d

^{13}C NMR (100 MHz, $CDCl_3$)

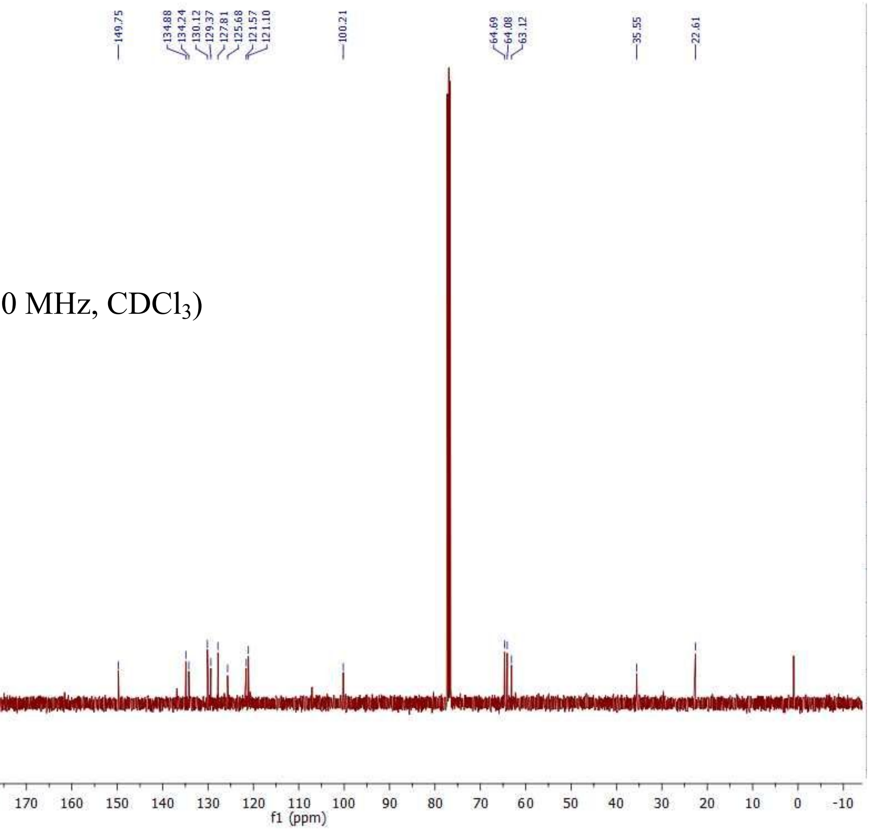

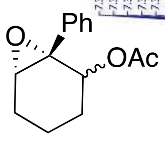

8ab

^{1}H NMR (400 MHz, $CDCl_3$)

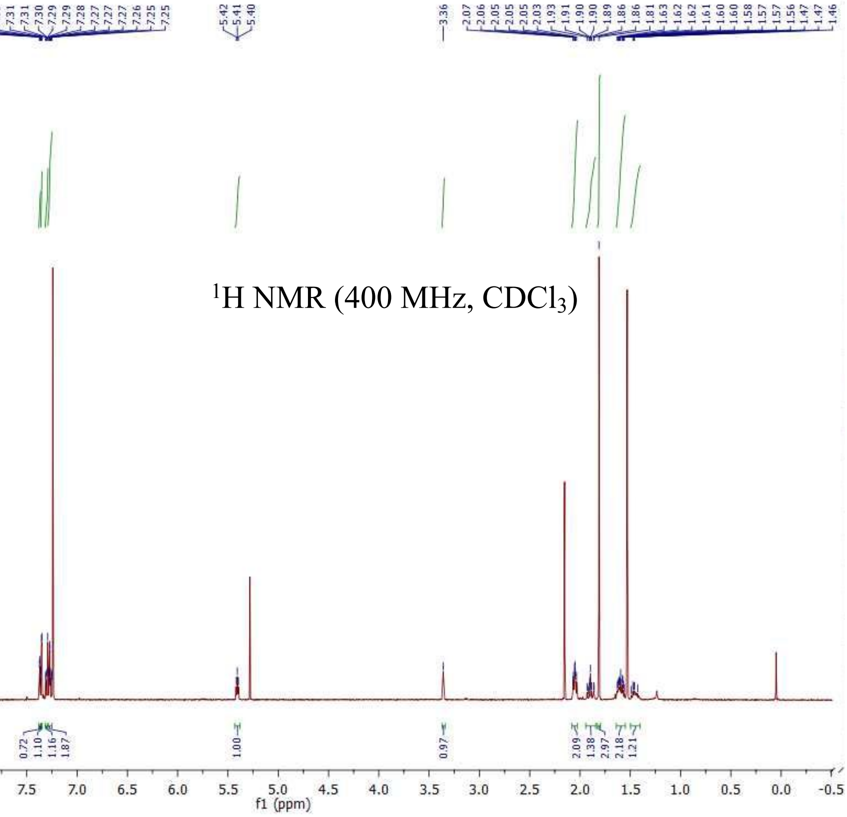

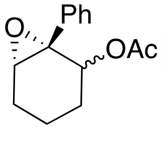

8ab

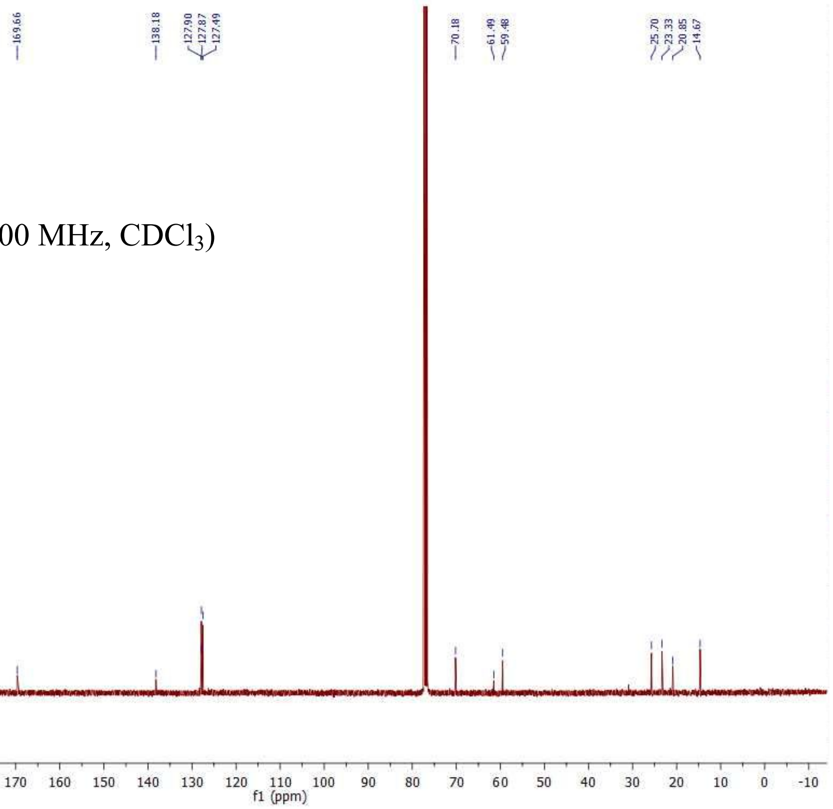

^{13}C NMR (100 MHz, $CDCl_3$)

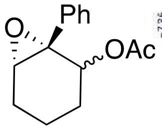

8ab'

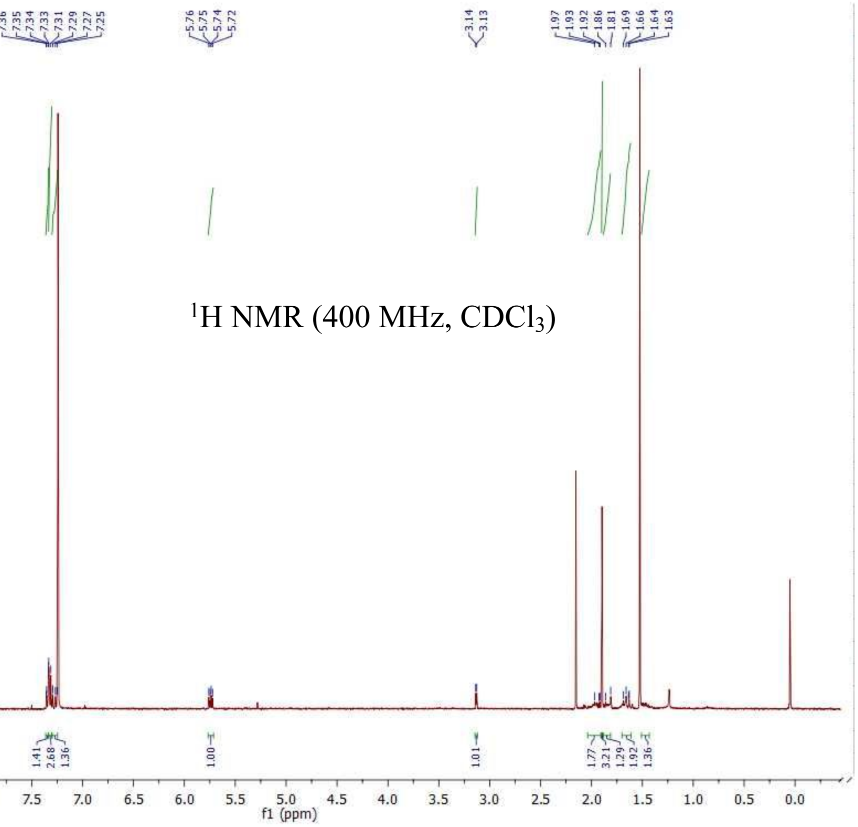

^{1}H NMR (400 MHz, $CDCl_3$)

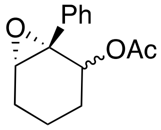

8ab'

^{13}C NMR (100 MHz, $CDCl_3$)

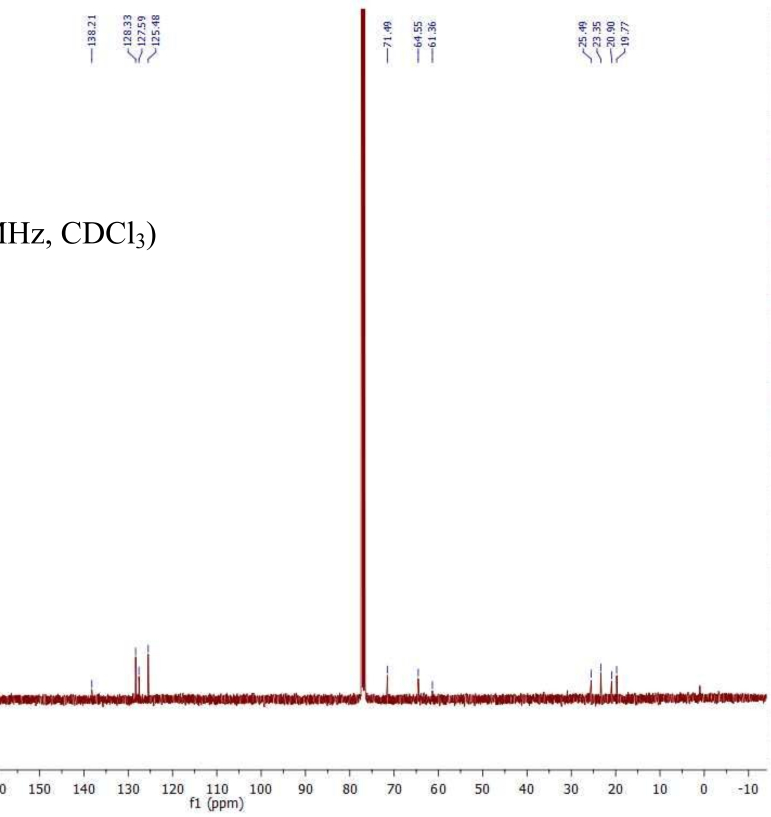

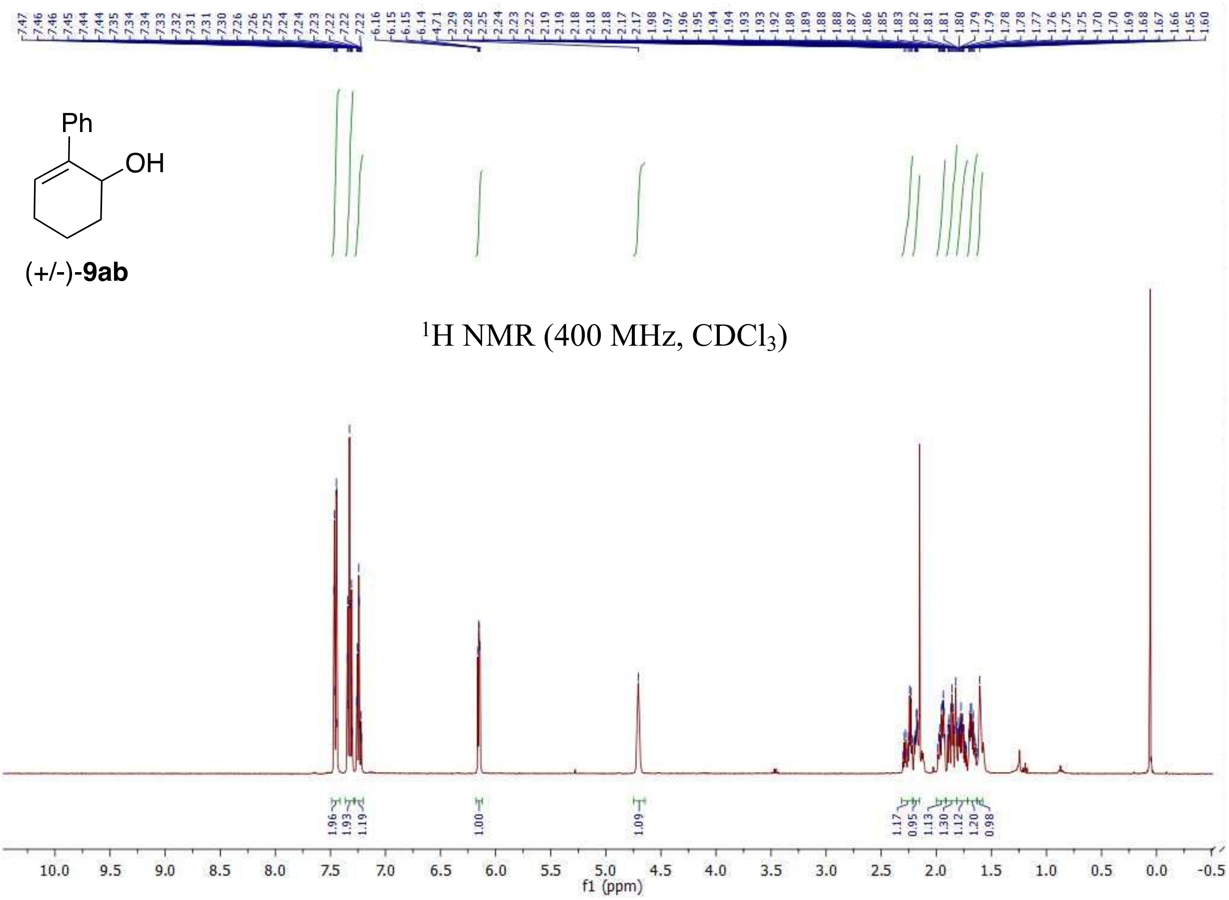

^{1}H NMR (400 MHz, $CDCl_3$)

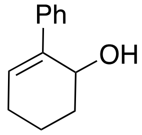

(+/-)-**9ab**

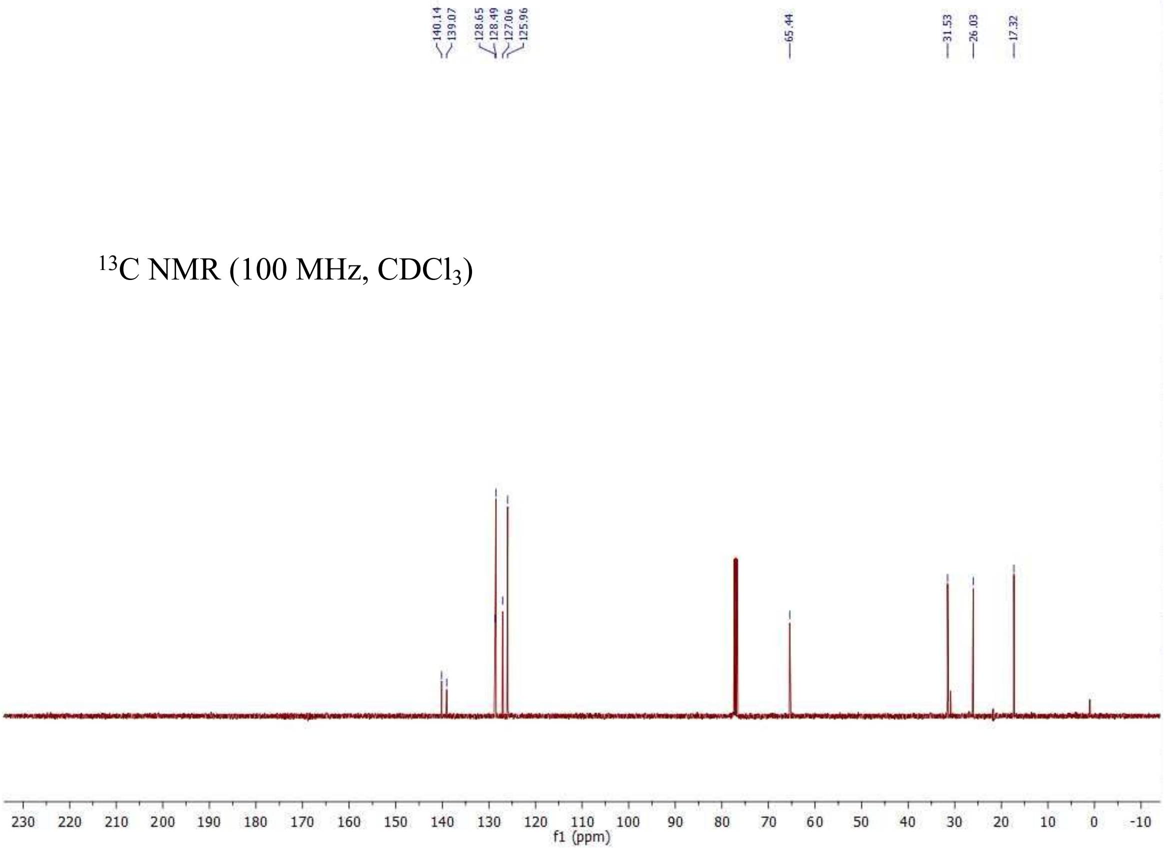

^{13}C NMR (100 MHz, $CDCl_3$)

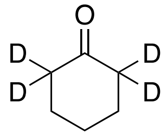

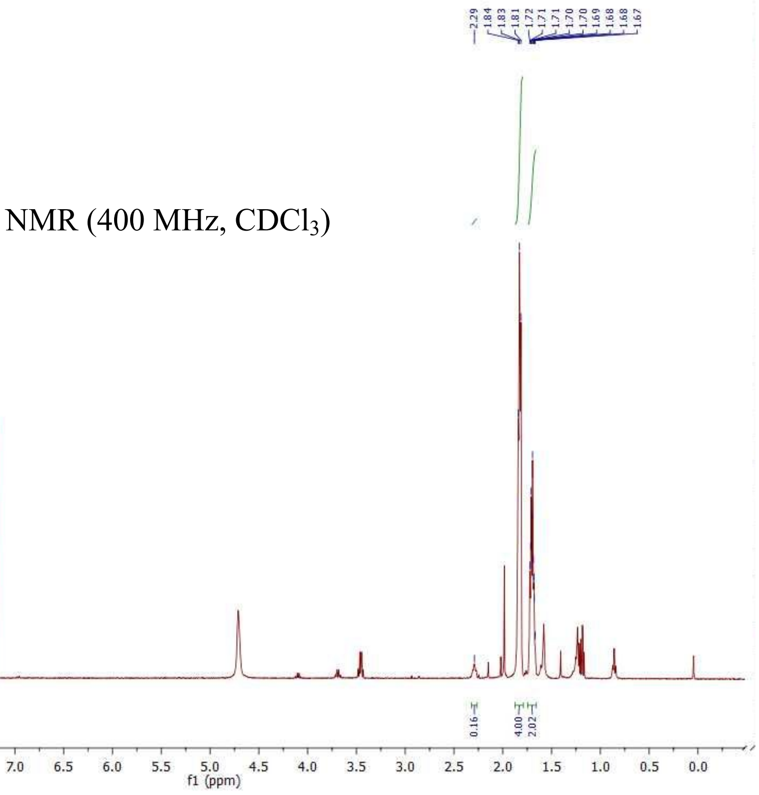

^{1}H NMR (400 MHz, $CDCl_3$)

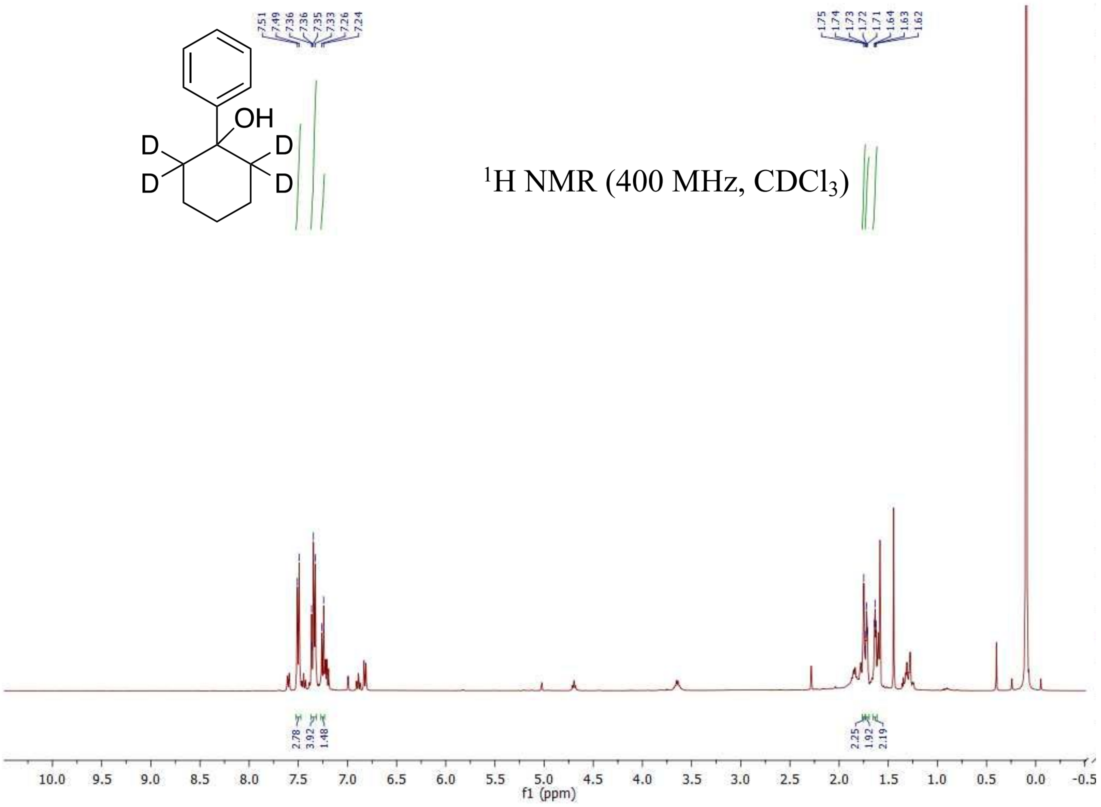
OH
D
D
D
D
1H NMR (400 MHz, CDCl3)
f1 (ppm)

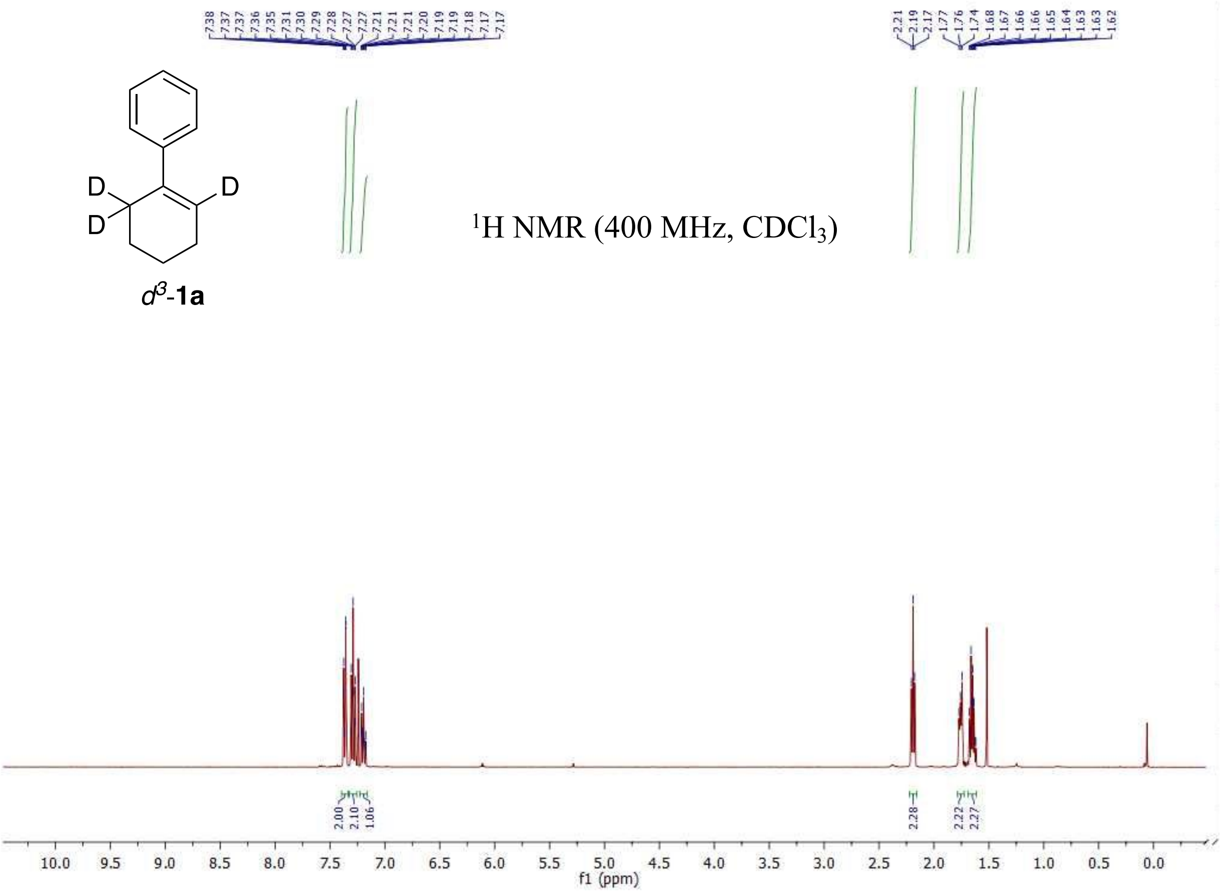
D
D
D
1H NMR (400 MHz, CDCl3)
f1 (ppm)

*d*3-**1a**

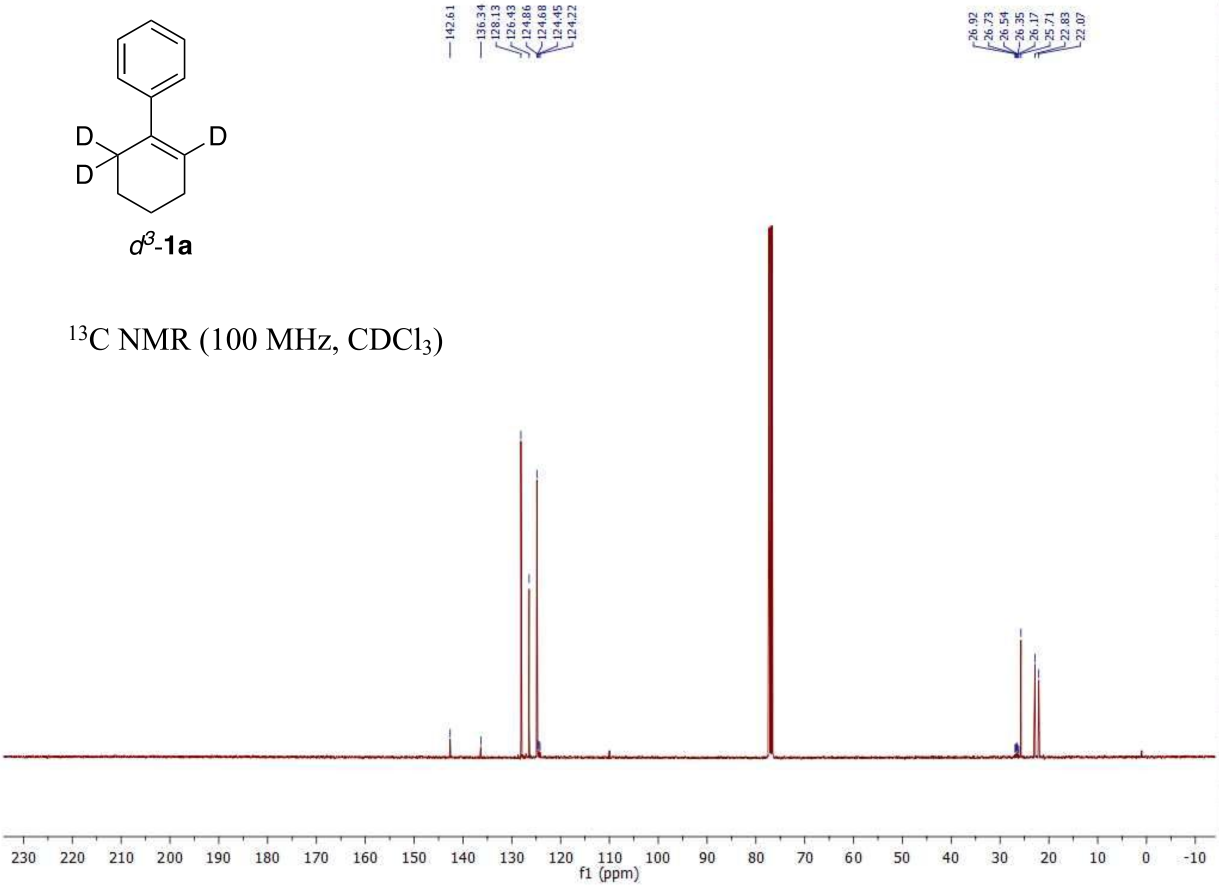

*d*3-**1a**

^{13}C NMR (100 MHz, CDCl$_3$)

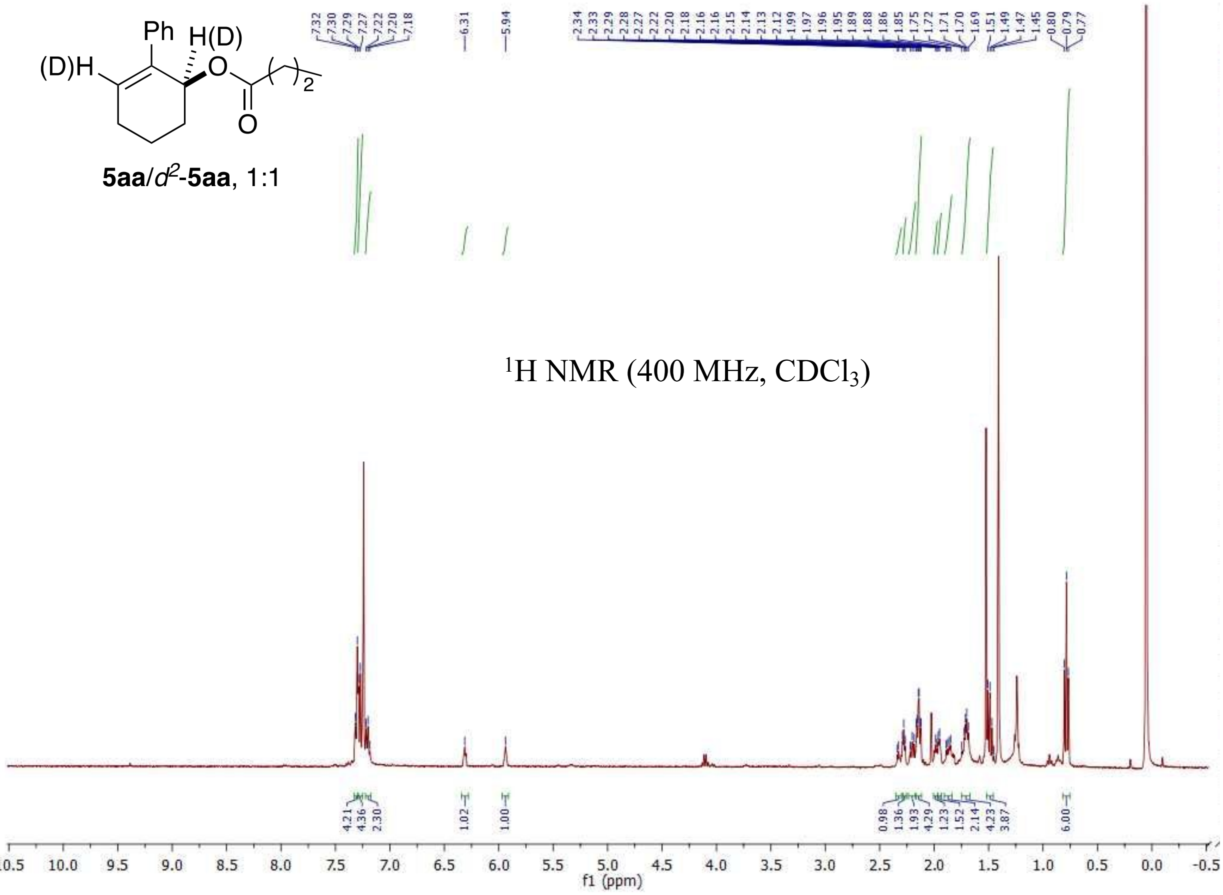

5aa/*d*2-**5aa**, 1:1

^{1}H NMR (400 MHz, CDCl$_3$)

References

[1] Montalti, M.; Credi, C.; Prodi, L.; Gandolfi, M. T. *Handbook of Photochemistry*; CRC Press: Boca Raton, FL, **2006**.

[2] Lim, B.-Y.; Jung, B.-E.; Cho, C.-G. Ene-hydrazide from Enol Triflate for the Regioselective Fischer Indole Synthesis. *Org. Lett.* 2014, **16**, 4492-4495.

[3] (a) Song, C.; Ma, Y.; Chai, Q.; Ma, C.; Jiangb, W.; Andrusa, M.B. Palladium catalyzed Suzuki–Miyaura coupling with aryl chlorides using a bulky phenanthryl *N*-heterocyclic carbene ligand. *Tetrahedron* 2005, **61**, 7438-7446. (b) Eliel, E.L.; Mancharan, M. Conformational analysis. 40. Conformation of 1-methyl-1-phenylcyclohexane and conformational energies of the phenyl and vinyl groups. *J. Org. Chem.* 1981, **46**, 1959-1962. (c) Rono, L.J.; Yayla, H.G.; Wang, D.Y.; Armstrong, M.F.; Knowles, R.R. Enantioselective Photoredox Catalysis Enabled by Proton-Coupled Electron Transfer: Development of an Asymmetric Aza-Pinacol Cyclization. *J. Am. Chem. Soc.* 2013, **135**, 17735-17738.

[4] (a) Li, Z.; Gandon, V.; Bour, C. Bimolecular vinylation of arenes by vinyl cations. *Chem. Commun.*, **2020**, *56*, 6507-6510. (b) Flores-Gaspara, A., Martin, R. Mechanistic Switch *via* Subtle Ligand Modulation: Palladium-Catalyzed Synthesis of α,β-Substituted Styrenes *via* C-H Bond Functionalization. *Adv. Synth. Cat.* 2011, **353**, 1223-1228. (c) Yanagisawa, A.; Nezu, T.; Mohri, S.-I. Brønsted acid-promoted hydrocyanation of arylalkenes. *Org. Lett.* 2009, **11**, 5286-5289. (d) Wu, X.; Zhou, J. Selective arylation at the vinylic site of cyclic olefins. *Chem. Commun.*, 2013, **49**, 4794-4796.

[5] Mahecha-Mahecha, C.; Lecornué, F; Akinari, S.; Charote, T.; Gamba-Sánchez, D.; Ohwada, T.; Thibaudeau, S. Sequential Suzuki–Miyaura Coupling/Lewis Acid-Catalyzed Cyclization: An Entry to Functionalized Cycloalkane-Fused Naphthalenes. *Org. Lett.* 2020, **22**, 6267-6271.

[6] Tian, Z.-X.; Qiao, J.-B.; Xu, G.-L.; Pang, X.; Qi, L.; Ma, W.-Y.; Zhao, Z.-Z.; Duan, J.; Du, Y.-F.; Su, P.; Liu, X.-Y.; Shu, X.-Z. Highly Enantioselective Cross-Electrophile Aryl-Alkenylation of Unactivated Alkenes. *J. Am. Chem. Soc.* 2019, **141**, 7637-7643.

[7] Imazaki, Y.; Shirakawa, E.; Ueno, R.; Hayashi, T. Ruthenium-Catalyzed Transformation of Aryl and Alkenyl Triflates to Halides. *J. Am. Chem. Soc.* 2012, **134**, 14760-14763.

[8] Olsson, V. J.; Szabó, K. J. Functionalization of Unactivated Alkenes through Iridium-Catalyzed Borylation of Carbon−Hydrogen Bonds. Mechanism and Synthetic Applications. *J. Org. Chem.* 2009, **74**, 7715-7723

[9] Elhalem, E.; Comin, M.J.; Rodriguez, J. B. Synthesis of Conformationally Locked Carbocyclic Nucleosides Built on a Thiabicyclo[3.1.0]hexane System as a Pseudosugar Surrogate. *Eur. J. Org. Chem.* 2006, 4473-4482.

[10] Pellicciari, R.; Natalini, B.; Sadeghpour, B. M.; Marinozzi, M.; Snyder, J. P.; Milliamson, B. L.; Keuthe, J. T.; Pawda, A. The Reaction of α-Diazo-β-hydroxy Esters with Boron Trifluoride Etherate: Generation and Rearrangement of Destabilized Vinyl Cations. A Detailed Experimental and Theoretical Study. *J. Am. Chem. Soc.* 1996, **118**, 1-12.

[11] White, A. R.; Wang, L.; Nicewicz, D. A. Synthesis and Characterization of Acridinium Dyes for Photoredox Catalysis. *Synlett*, 2019, **30**, 827-832.
